# Iridium-Catalyzed Arylative Cyclization of Alkynones by 1,4-Iridium Migration**

**DOI:** 10.1002/anie.201403271

**Published:** 2014-05-19

**Authors:** Benjamin M Partridge, Jorge Solana González, Hon Wai Lam

**Affiliations:** EaStCHEM, School of Chemistry, University of EdinburghJoseph Black Building, The King's Buildings, West Mains Road, Edinburgh, EH9 3JJ (UK)

**Keywords:** alkynes, boron, catalysis, C—H activation, iridium

## Abstract

1,4-Metal migrations enable the remote functionalization of C—H bonds, and have been utilized in a wide variety of valuable synthetic methods. The vast majority of existing examples involve the 1,4-migration of palladium or rhodium. Herein, the stereoselective synthesis of complex polycycles by the iridium-catalyzed arylative cyclization of alkynones with arylboronic acids is described. To our knowledge, these reactions involve the first reported examples of 1,4-iridium migration.

Since the early reports of 1,4-palladium migration[1a–d] and 1,4-rhodium migration,[2a,b] numerous catalytic reactions involving 1,4-metal migration have been developed.[1–5] Such processes enable the remote functionalization of C—H bonds, allowing the introduction of metal centers at positions that would otherwise be difficult to metalate. To date, reactions involving the 1,4-migration of palladium,[1] rhodium,[2] platinum,1q nickel,[4] and cobalt[5] have been achieved. The demonstration of the ability of other metals to undergo 1,4-migration would be valuable, as their distinct properties may offer new opportunities for the development of useful synthetic methods. Herein, we describe the preparation of highly functionalized polycycles by the iridium-catalyzed arylative cyclization of alkynones. One of the key steps in this transformation is a 1,4-iridium migration, which, to our knowledge, has not been described previously.

During a program aimed at the stereoselective synthesis of complex polycycles by the desymmetrization of cyclic 1,3-diketones,[6, 7] we became interested in developing an arylative cyclization of substrates such as **1 a** (Scheme 1). We envisaged that in the presence of a suitable metal complex, an arylboron reagent could be employed in an arylmetalation of the alkyne moiety of **1 a** to give alkenylmetal species **3**. This intermediate could then undergo an alkenyl-to-aryl 1,4-migration to provide intermediate **4**, which could then participate in the nucleophilic attack of one of the ketones to give tertiary-alcohol-containing tricycle **2 a**.

**Scheme 1:**
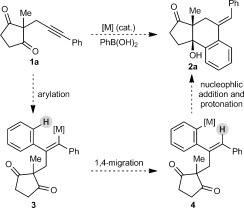
Proposed arylative cyclization of alkynones.

In view of the success of rhodium catalysis in related transformations,[2d–g,i,k–q] the reaction of **1 a** with PhB(OH)_2_ in the presence of [{Rh(cod)Cl}_2_] (1.5 mol %), KF (1.5 equiv) as a mild base, and *t*BuOH (1.5 equiv) as a proton source was examined [Eq. (1)]. Heating the reaction in toluene at 65 °C for 16 hours did indeed provide tricycle **2 a** in 41 % yield. However, **2 a** was accompanied by the simple alkyne hydroarylation product **5** (18 % yield) and the ring-expansion product **6** (17 % yield), which is formed by initial arylation of the alkyne with the opposite regioselectivity, followed by a cyclization–fragmentation process, as described by Murakami and co-workers.[8]

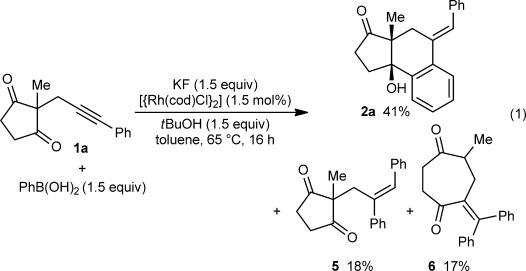


In an effort to increase the yield of **2 a**, catalyst systems based upon other metals known to undergo 1,4-migrations (Pd,[1] Pt,1q Ni,[4] and Co[5]) were surveyed. However, no reaction was observed in these experiments. Fortunately, [{Ir(cod)Cl}_2_] (1.5 mol %) was effective, and provided **2 a** in 72 % yield [Eq. (2)]. Interestingly, this experiment also gave product **7** in 27 % yield, the structure of which was determined by X-ray crystallography.[9] Compound **7** is a 2:1 adduct of **1 a** and PhB(OH)_2_, respectively, resulting from a complex sequence beginning with the arylmetalation of the alkyne of **1 a** with the regioselectivity opposite to that seen in the formation of **2 a**.[10, 11] To our knowledge, this reaction involves the first reported examples of 1,4-iridium migration. Given that the yield of **2 a** was higher using an iridium- rather than a rhodium-based precatalyst, [{Ir(cod)Cl}_2_] was selected for further studies.

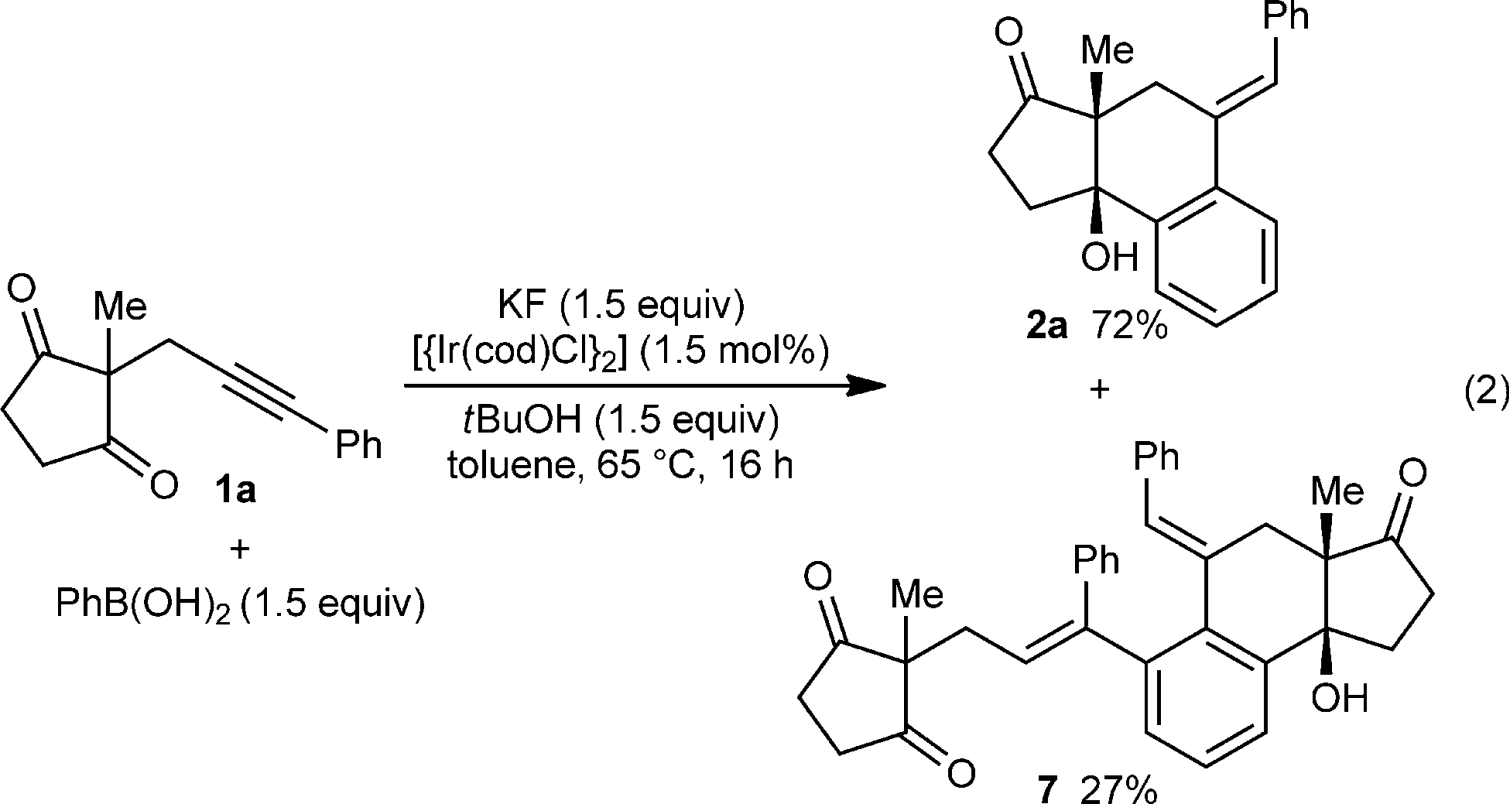


The iridium-catalyzed arylative cyclization of various substrates with PhB(OH)_2_ was then explored (Scheme 2). In all reactions, the 2:1 adduct was observed in approximately 10–25 % yield by ^1^H NMR analysis of the reaction mixtures, but these products were not isolated. Substituents at the *para*, *meta*, *or ortho* positions of the aryl group on the alkyne were tolerated (**2 b**–**f**), though in the case where an *ortho*-cyano group was present, a higher loading of [{Ir(cod)Cl}_2_] (2.5 mol %) was required for full conversion (**2 f**). With *para*-substituted phenyl groups, electron-poor rather than electron-rich arenes led to higher yields of the products (compare **2 b**–**d**), which is likely due to a more regioselective initial arylmetalation of more polarized alkynes. The relative configurations of the stereogenic centers and the *E* geometry of the alkenes in the products were assigned by analogy with **2 d**, the structure of which was determined by X-ray crystallography.[9] Substrates containing a terminal alkyne or an alkyne lacking an aryl substituent did not undergo the reaction and returned only unreacted starting material (**2 g** and **2 h**).

**Scheme 2:**
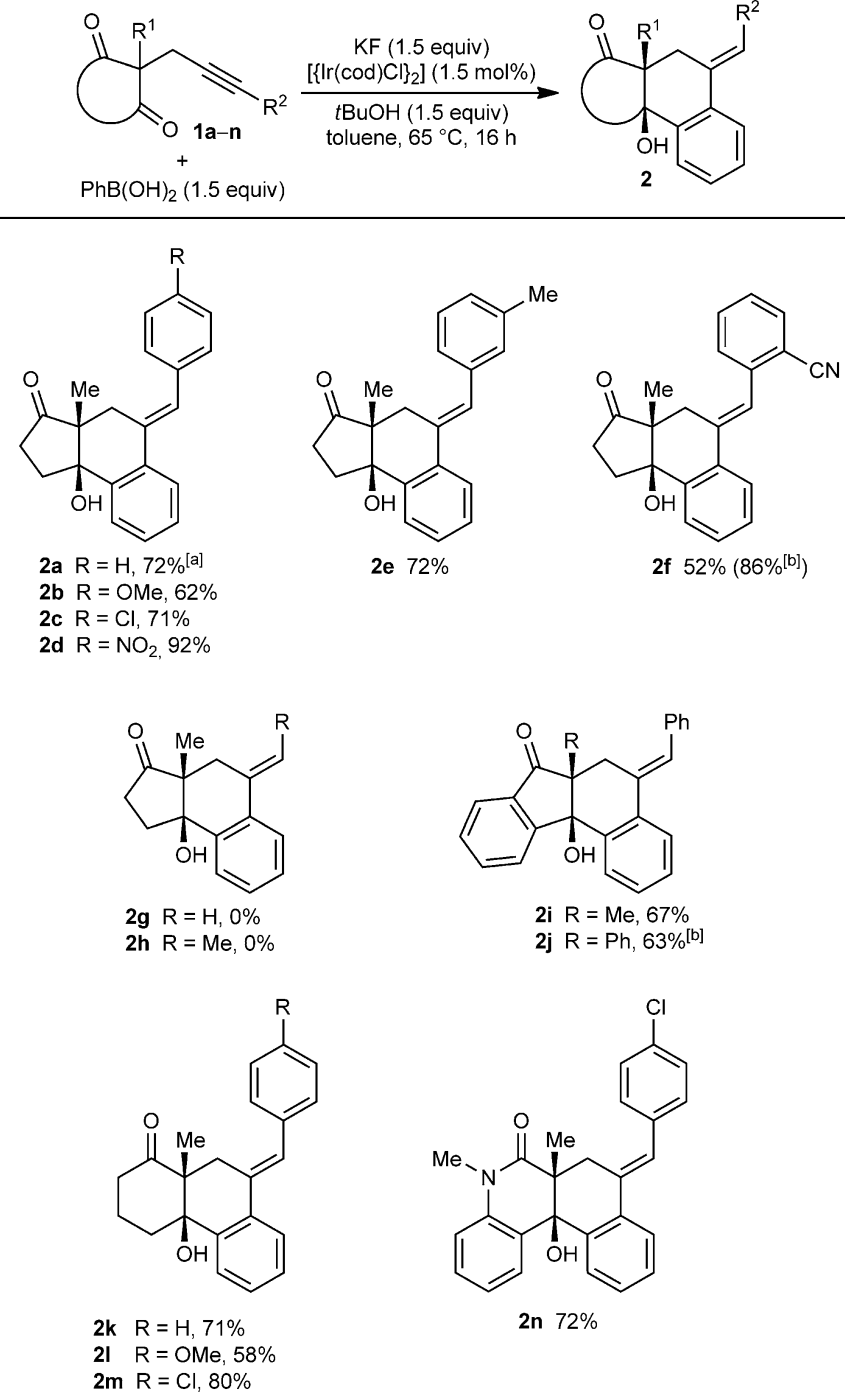
Arylative cyclization of various alkynones. Reactions were conducted using 0.40 mmol of 1 a–n in toluene (4 mL). Cited yields are of isolated products. [a] Compound 7 was also isolated in 27 % yield. See Equation (2). [b] 2.5 mol % of [{Ir(cod)Cl}_2_] was used.

Next, variations of the pendant ketone were examined. An indane-1,3-dione reacted well to give **2 i** in 67 % yield. Changing the substituent at C2 (between the ketones) from a methyl to a phenyl group was tolerated, and **2 j** was obtained in 63 % yield using 2.5 mol % of [{Ir(cod)Cl}_2_]. Switching from five- to six-membered ring diketones was also possible (**2 k**–**m**[9]). In these cases, and in a similar fashion to the five-membered ring substrates, the reactions of substrates containing more electron-deficient arenes on the alkyne led to higher yields than those with electron-rich arenes (compare **2 k**–**m**). A cyclic β-ketoamide was also tolerated, providing **2 n** in 72 % yield.

The process is not limited to cyclic 1,3-dicarbonyl substrates in which both carbonyl groups are part of the ring; the β-ketoester **8** also underwent arylative cyclization to give **9** in 72 % yield [Eq. (3)]. However, substrate **8** was less reactive than those employed in the experiments shown in Scheme 1, and higher loadings of [{Ir(cod)Cl}_2_] and the reagents were required for an acceptable yield of **9**.

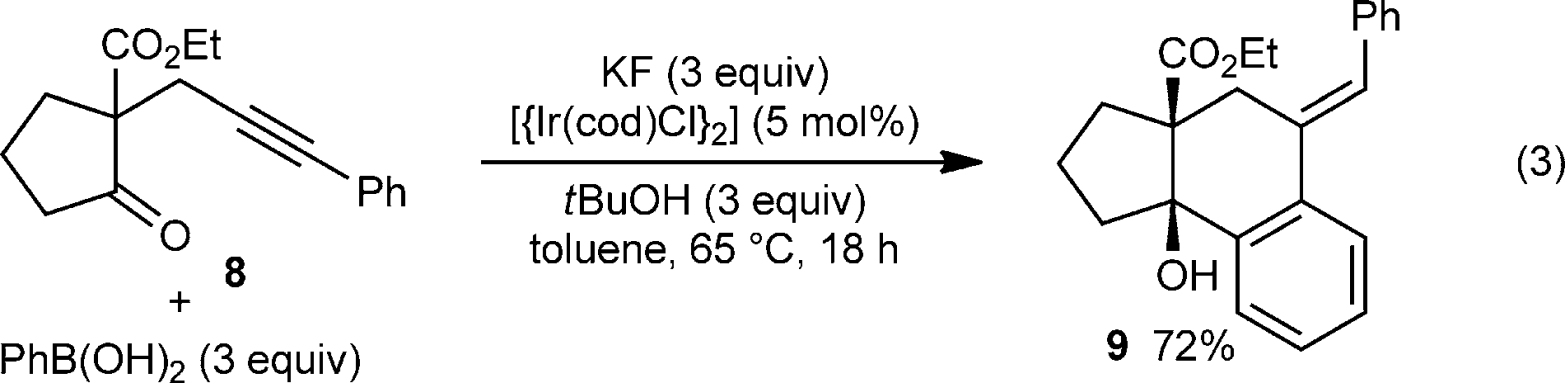


Table 1 presents the results of arylative cyclization of **1 a** with various arylboronic acids. The reaction was compatible with methyl (Table 1, entry 5), methoxy (Table 1, entry 1), halide (Table 1, entries 1 and 5), or ester groups (Table 1, entry 3) at either the *para* or *meta* positions of the arylboronic acid. However, with electron-withdrawing substituents, a higher catalyst loading (5 mol % of Ir) was required for acceptable yields (Table 1, entries 2, 3, and 5). With a 4-carboethoxy group, the yield was lower (35 %), and unreacted **1 a** was recovered in 41 % yield (Table 1, entry 3). 2-Naphthylboronic acid also reacted smoothly to give **10 f** in 59 % yield (Table 1, entry 6). Importantly, the reactions of *meta*-substituted arylboronic acids were highly regioselective (≥10:1 regioisomeric ratio, determined by ^1^H NMR analysis of the unpurified reaction mixtures) and provided **10 d**–**f** as the major products (Table 1, entries 4–6). These results demonstrate that there is a strong preference for iridium to undergo 1,4-migration to the sterically least hindered site of the arene.[12]

**Table 1:** Arylative cyclization of 1 a with various arylboronic acids.[a] 
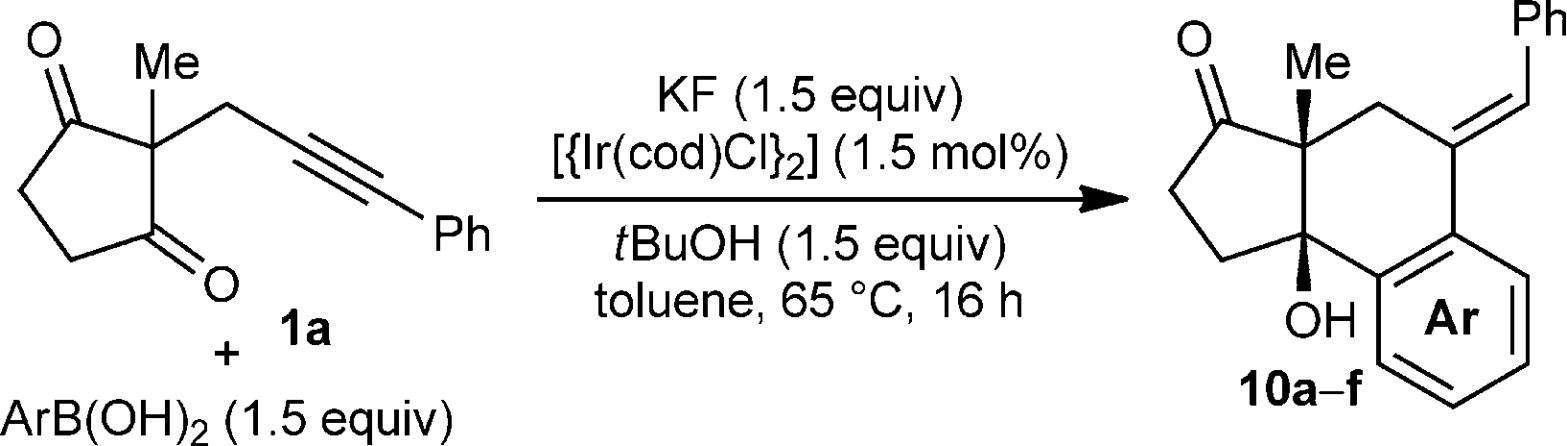

Entry	Ar	Product		Yield [%][b]
1 2 3	4-MeOC_6_H_4_ 4-ClC_6_H_4_ 4-EtO_2_CC_6_H_4_	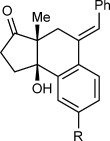	**10 a** R=OMe **10 b** R=Cl **10 c** R=CO_2_Et	69 62[c] 35[c,^d^,e]
				
4 5	3-MeC_6_H_4_ 3-BrC_6_H_4_	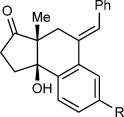	**10 d** R=Me **10 e** R=Br	68[f] 58[c,f]
				
6	2-naphthyl	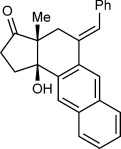	**10 f**	59[c,^f^,g]

[a] Reactions were conducted with 0.40 mmol of **1 a** in toluene (4 mL).

[b] Yields of isolated products.

[c] 2.5 mol % of [{Ir(cod)Cl}_2_] was used.

[d] 3.0 equiv each of ArB(OH)_2_, KF, and *t*BuOH were used.

[e] Substrate **1 a** was recovered in 41 % yield.

[f] Single regioisomer observed.

[g] Reaction conducted at 90 °C.

Next, the arylative cyclization of **1 a** with pentadeuteriophenylboronic acid was conducted [Eq. (4)]. The product [D_5_]-**2 a** was deuterated on the alkene (>95 % deuterium incorporation by ^1^H NMR analysis), a result that is consistent with the proposed mechanism involving alkenyl-to-aryl 1,4-iridium migration (Scheme 1).

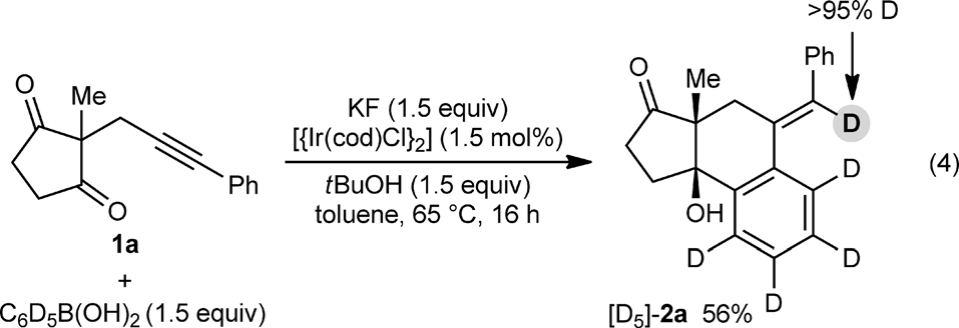


A possible catalytic cycle for these transformations, using **1 a** and PhB(OH)_2_ for illustrative purposes, is shown in Scheme 3. First, an aryliridium species **12** is generated by transmetalation from the arylboronic acid to the iridium butoxide **11** (or alternatively, an iridium fluoride). Migratory insertion of the alkyne into **12** then occurs to give alkenyliridium species **13**,[13, 14] which then undergoes 1,4-migration. The resulting aryliridium intermediate **14** then undergoes nucleophilic attack onto one of the ketones to give iridium alkoxide **15**. Protonation of **15** with *t*BuOH releases the product **2 a** and regenerates the iridium butoxide **11**.

**Scheme 3:**
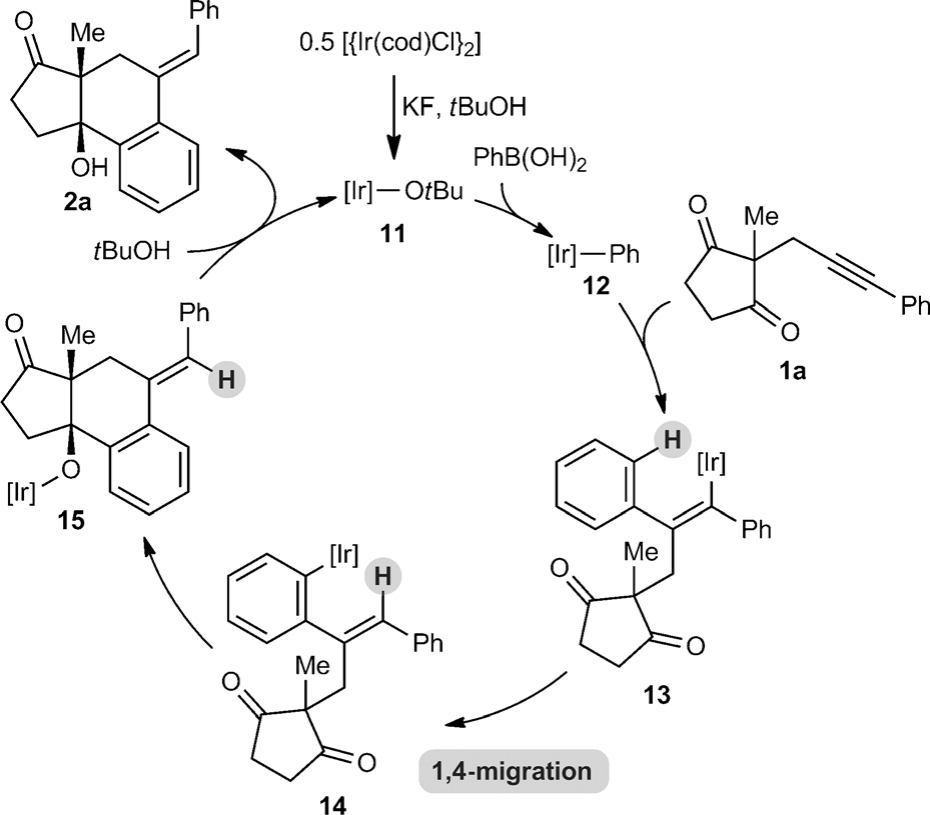
Proposed catalytic cycle for the arylative cyclization.

Preliminary attempts at developing an enantioselective variant of this process revealed that (*R*)-Difluorphos (**L1**) gave high enantioselectivities. For example, the arylative cyclization of alkynones **1 c** and **1 i** provided (+)-**2 c** and (−)-**2 i** in 90 % *ee* and 91 % *ee*, respectively, using 10 mol % of the iridium–bisphosphine complex under slightly modified reaction conditions compared with those used in the racemic reactions [Eqs. (5) and (6)].[9, 15] However, the activity of this iridium–bisphosphine complex was modest, and significant quantitites of the starting materials were returned. Interestingly, 2:1 adducts analogous to **7** were not observed in these reactions.

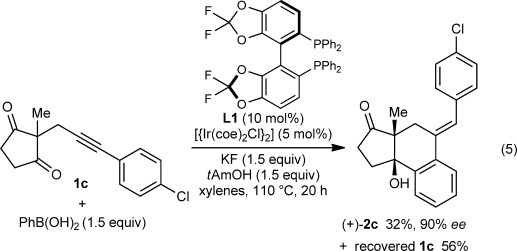


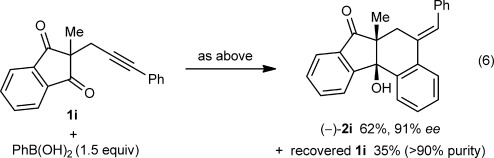


In summary, we have reported the iridium-catalyzed arylative cyclization of alkynones with arylboronic acids.[16] These reactions involve 1,4-iridium migration as a key step, a mode of reactivity for iridium that, to our knowledge, has not been reported previously.[17] Efforts to exploit the 1,4-migration of iridium and other metals in new catalytic transformations are ongoing in our group.
