# Supplementary material for: Iridium-Catalyzed Arylative Cyclization of Alkynones by 1,4-Iridium Migration
Source: Angew Chem Int Ed Engl. 2014 May 19;53(25):6523–7. doi: 10.1002/anie.201403271 (PMC4464530; doi:10.1002/anie.201403271)
Supplement: Supplementary file 1 [file anie0053-6523-sd1.pdf]

Angewandte Chemie
Eine Zeitschrift der Gesellschaft Deutscher Chemiker

Supporting Information
© Wiley-VCH 2014
69451 Weinheim, Germany

# Iridium-Catalyzed Arylative Cyclization of Alkynones by 1,4-Iridium Migration**

*Benjamin M. Partridge, Jorge Solana González, and Hon Wai Lam**

anie_201403271_sm_miscellaneous_information.pdf

1. General Information ........ 2
2. Synthesis of Substrates ........ 3
3. Rhodium-Catalyzed Arylative Cyclization of 1a ........ 13
4. Racemic Iridium Catalyzed Arylative Cyclizations ........ 15
5. Enantioselective Iridium-Catalyzed Arylative Cyclizations ........ 28
6. Semi-Quantitative ICP-MS Analysis ........ 31
7. NMR Spectra ........ 32

## 1. General Information

All air-sensitive reactions were carried out under a nitrogen atmosphere using oven-dried apparatus. Anhydrous toluene and MeCN were dried and purified by passage through activated alumina columns using a solvent purification system. Xylenes were degassed by freeze-pump-thaw and stored in a Young's flask under nitrogen prior to use. All commercially available reagents were used as received unless otherwise stated. Arylboronic acids were used as received unless the sample contained >10% boroxine as determined by $^{1}H$ NMR analysis. In this case, the boronic acid was stirred in a mixture of $Et_2O$ and water for 30 minutes. The organic phase was separated, dried ($Na_2SO_4$), filtered, and concentrated *in vacuo* to give the corresponding boronic acid which was used without further purification. All petroleum ether used was 40-60 °C petroleum ether.

Thin layer chromatography (TLC) was performed on Merck DF-Alufoilien $60F_{254}$ 0.2 mm precoated plates. Compounds were visualized by exposure to UV light or by dipping the plates into solutions of potassium permanganate or vanillin followed by heating. Flash column chromatography was carried out using silica gel (Fisher Scientific 60Å particle size 35-70 micron). Melting points were recorded on a Gallenkamp melting point apparatus and are uncorrected. The solvent of recrystallization is reported in parentheses. Infra-red spectra were recorded on either a Shimadzu IRAffinity-1 or a Nicolet Avatar 360 FT instrument on the neat compound. NMR spectra were acquired on either a Bruker AVA500 or a Bruker AVA400 spectrometer. $^{1}H$ and $^{13}C$ NMR spectra were referenced to external tetramethylsilane via the residual protonated solvent ($^{1}H$) or the solvent itself ($^{13}C$). All chemical shifts are reported in parts per million (ppm). For $CDCl_3$, the shifts are referenced to 7.27 ppm for $^{1}H$ NMR spectroscopy and 77.0 ppm for $^{13}C$ NMR spectroscopy. For $D_6$-DMSO, the shifts are referenced to 2.50 ppm for $^{1}H$ NMR spectroscopy and 39.52 ppm for $^{13}C$ NMR spectroscopy. High-resolution mass spectra were recorded using electrospray ionization (ESI) or electron impact (EI) techniques at the EPSRC National Mass Spectrometry Facility, at the School of Chemistry, University of Edinburgh, or at the School of Chemistry, University of Nottingham. X-ray diffraction data were collected at 120 K on either an Agilent SuperNova diffractometer using Mo Kα radiation at 0.71 Å or on an Agilent GV1000 using CuKα radiation, and refined in SHELXTL. Optical rotations were performed on a Bellingham and Stanley ADP 400 polarimeter. Chiral HPLC analysis was performed on an Agilent Technologies 1260 Infinity instrument using a CHIRALPAK® IA-3 column (4.6 × 250 mm).

## 2. Synthesis of Substrates

**2-Methyl-2-(3-phenylprop-2-yn-1-yl)cyclopentane-1,3-dione (1a)**

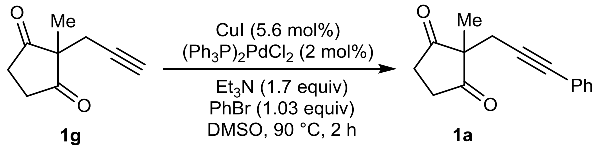


2-Methyl-2-propargyl-1,3-cyclopentanedione (**1g**)[1] (1.50 g, 10.0 mmol) was added to a solution of $Pd(PPh_3)_2Cl_2$ (140 mg, 0.199 mmol), CuI (106 mg, 0.557 mmol), and $Et_3N$ (2.4 mL, 17.2 mmol) in anhydrous DMSO (20 mL). Bromobenzene (1.08 mL, 10.3 mmol) was added and the mixture was stirred at 90 °C for 2 h. The reaction was cooled to room temperature, water (50 mL) was added, and the mixture was extracted with $Et_2O$ (50 mL). The combined organic phases were washed with 10% aqueous HCl (3 × 20 mL), dried ($Na_2SO_4$), filtered, and concentrated *in vacuo*. The mixture was purified by column chromatography (10% EtOAc/hexane) to give *alkynone* **1a** (1.90 g, 84%) as a pale yellow solid. $R_f$ = 0.35 (30% EtOAc/hexane); m.p. 64-65 °C ($Et_2O$/hexane); IR 2970, 1721 (C=O), 1412, 1065, 760 $cm^{-1}$; $^1H$ NMR (400 MHz, $CDCl_3$) δ 7.37-7.27 (5H, m, Ar**H**), 2.90-2.80 (4H, m, C**H**$_2$C**H**$_2$), 2.70 (2H, s, C**H**$_2$C≡C), 1.19 (3H, s, C**H**$_3$); $^{13}C$ NMR (126 MHz, $CDCl_3$) δ 215.6 (2 × C), 131.6 (2 × CH), 128.3 (CH), 128.3 (2 × CH), 122.5 (C), 83.9 (C), 82.9 (C), 55.5 (C), 36.0 (2 × $CH_2$), 25.9 ($CH_2$), 18.9 ($CH_3$); HRMS (ESI) Exact mass calcd for $C_{15}H_{18}NO_2$ $[M+NH_4]^+$: 244.1338, found: 244.1332.

**2-[3-(4-Methoxyphenyl)prop-2-yn-1-yl]-2-methylcyclopentane-1,3-dione (1b)**

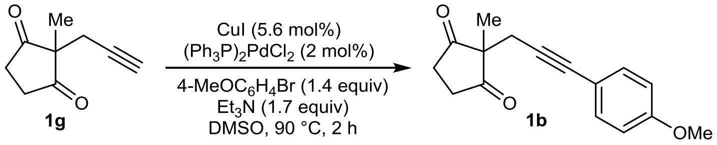


2-Methyl-2-propargyl-1,3-cyclopentanedione (**1g**)[1] (1.50 g, 10.0 mmol) was added to a solution of $Pd(PPh_3)_2Cl_2$ (140 mg, 0.199 mmol), CuI (106 mg, 0.557 mmol), and $Et_3N$ (2.4 mL, 17.2 mmol) in anhydrous DMSO (20 mL). 4-Bromoanisole (1.72 mL, 13.7 mmol) was added and the mixture was stirred at 90 °C for 2 h. The reaction was cooled to room temperature, water (50 mL) was added, and the mixture was extracted with $Et_2O$ (50 mL). The organic layer was washed with 10% aqueous HCl (3 × 20 mL), dried ($Na_2SO_4$), filtered, and concentrated *in vacuo*. The mixture was purified by column chromatography (10% EtOAc/hexane) to give *alkynone* **1b** (1.82 g, 71%) as a brown solid. $R_f$ = 0.33 (30% EtOAc/hexane); m.p. 80-85 °C (hexane); IR 2965, 1722 (C=O), 1508, 1242, 1032,

1. Preparted according to: P. T. Lansbury, A. K. Serelis, J. E. Hengeveld, D. G. Hangauer Jr, *Tetrahedron* **1980**, *36*, 2701-2710.

833 cm$^{-1}$; $^{1}$H NMR (500 MHz, $CDCl_3$) δ 7.26 (2H, d, *J* = 8.9 Hz, Ar**H**), 6.80 (2H, d, *J* = 8.9 Hz, Ar**H**), 3.80 (3H, s, OC**H**$_3$), 2.89-2.78 (4H, m, C**H**$_2$C**H**$_2$), 2.68 (2H, s, C**H**$_2$C≡C), 1.17 (3H, s, CC**H**$_3$); $^{13}$C NMR (126 MHz, $CDCl_3$) δ 215.8 (2 × C), 159.6 (C), 133.0 (2 × CH), 114.6 (C), 113.9 (2 × CH), 82.7 (C), 82.4 (C), 55.5 (C), 55.3 ($CH_3$), 36.0 (2 × $CH_2$), 26.2 ($CH_2$), 18.8 ($CH_3$); HRMS (ESI) Exact mass calcd for $C_{16}H_{17}O_3$ $[M+H]^+$: 257.1178, found: 257.1173.

**2-[3-(4-Chlorophenyl)-2-propynyl]-2-methylcyclopentane-1,3-dione (1c)**

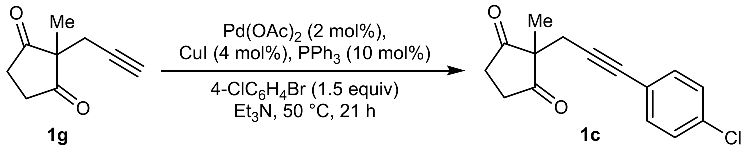


$Et_3N$ (21 mL) was added to a flask containing 2-methyl-2-propargyl-1,3-cyclopentanedione (**1g**)[1] (594 mg, 3.96 mmol), 1-bromo-4-chlorobenzene (1.14 g, 5.94 mmol), $PPh_3$ (41.7 mg, 0.159 mmol), $Pd(OAc)_2$ (17.8 mg, 0.080 mmol), and CuI (30.2 mg, 0.159 mmol) and the mixture was stirred at 50 °C for 21 h. The reaction was cooled to room temperature, water (20 mL) and saturated aqueous $NH_4Cl$ (5 ml) were added, and the mixture was extracted with EtOAc (3 × 25 mL). The combined organic phases were washed with brine (40 mL), dried ($MgSO_4$), filtered, and concentrated *in vacuo*. The mixture was purified by column chromatography (30% EtOAc/hexane) to give *alkynone* **1c** (382 mg, 37%) as a pale yellow solid. $R_f$ = 0.39 (30% EtOAc/hexane); m.p. 85-90 °C (hexane); IR 2968, 1719 (C=O), 1491, 1412, 1094, 835 cm$^{-1}$; $^{1}$H NMR (500 MHz, $CDCl_3$) δ 7.25 (4H, s, Ar**H**), 2.90-2.77 (4H, m, C**H**$_2$C**H**$_2$), 2.69 (2H, s, C**H**$_2$C≡C), 1.19 (3H, s, C**H**$_3$); $^{13}$C NMR (126 MHz, $CDCl_3$) δ 215.3 (2 × C), 134.4 (C), 132.8 (2 × CH), 128.6 (2 × CH), 121.0 (C), 85.1 (C), 81.8 (C), 55.4 (C), 35.9 (2 × $CH_2$), 25.6 ($CH_2$), 19.1 ($CH_3$); HRMS (ESI) Exact mass calcd for $C_{15}H_{14}{}^{35}ClO_2$ $[M+H]^+$: 261.0676, found: 261.0677.

**2-Methyl-2-[3-(4-nitrophenyl)prop-2-yn-1-yl]cyclopentane-1,3-dione (1d)**

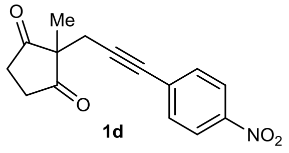


Prepared according to a previously reported procedure.[2]

2. J. D. Dooley, S. Reddy Chidipudi, H. W. Lam, *J. Am. Chem. Soc.* **2013**, *135*, 10829-10836.

**2-Methyl-2-[3-(methylphenyl)prop-2-yn-1-yl]cyclopentane-1,3-dione (1e)**

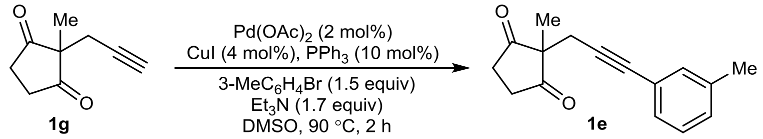


2-Methyl-2-propargyl-1,3-cyclopentanedione (**1g**)[1] (1.50 g, 10.0 mmol) was added to a solution of $Pd(OAc)_2$ (56 mg, 0.25 mmol), $PPh_3$ (262 mg, 1.00 mmol), CuI (106 mg, 0.557 mmol), and $Et_3N$ (2.4 mL, 17.2 mmol) in anhydrous DMSO (20 mL). A solution of 3-bromotoluene (1.33 mL, 11.0 mmol) in anhydrous DMSO (10 mL) was added and the mixture was stirred at 90 °C for 2 h. The reaction was cooled to room temperature, water (50 mL) was added, and the mixture was extracted with $Et_2O$ (50 mL). The organic phase was washed with 10% aqueous HCl (3 × 20 mL), dried ($Na_2SO_4$), filtered, and concentrated *in vacuo*. The mixture was purified by column chromatography (10% EtOAc/hexane) to give *alkynone* **1e** (2.10 g, 88%) as a brown oil. $R_f$ = 0.48 (30% EtOAc/hexane); IR 2924, 1724 (C=O), 1317, 1070, 783, 691 $cm^{-1}$; $^1H$ NMR (500 MHz, $CDCl_3$) δ 7.17-7.09 (4H, m, Ar**H**), 2.92-2.77 (4H, m, C$\mathbf{H}_2$C$\mathbf{H}_2$), 2.69 (2H, s, C$\mathbf{H}_2$C≡C), 2.31 (3H, s, ArC$\mathbf{H}_3$), 1.18 (3H, s, O=CCC$\mathbf{H}_3$); $^{13}C$ NMR (126 MHz, $CDCl_3$) δ 215.6 (2 × C), 138.0 (C), 132.1 (CH), 129.2 (CH), 128.6 (CH), 128.1 (CH), 122.3 (C), 83.5 (C), 83.0 (C), 55.4 (C), 35.9 (2 × $CH_2$), 26.0 ($CH_2$), 21.1 ($CH_3$), 18.8 ($CH_3$); HRMS (ESI) Exact mass calcd for $C_{16}H_{17}O_2$ $[M+H]^+$: 241.1229, found: 241.1224.

**2-[3-(1-Methyl-2,5-dioxo-3-cyclopenten-1-yl)-1-propynyl]benzonitrile (1f)**

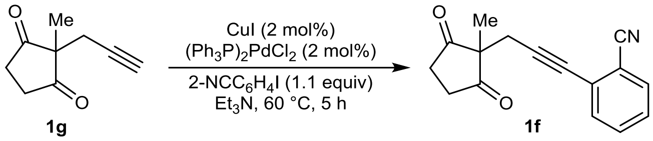


$Et_3N$ (26 mL) was added to a flask containing 2-methyl-2-propargyl-1,3-cyclopentanedione (**1g**)[1] (790 mg, 5.28 mmol), 2-iodobenzonitrile (1.33 g, 5.81 mmol), $Pd(PPh_3)_2Cl_2$ (74.4 mg, 0.106 mmol), and CuI (20.1 mg, 0.106 mmol) and the mixture was stirred at 60 °C for 5 h. The reaction was cooled to room temperature, water (20 mL) and saturated aqueous $NH_4Cl$ (5 ml) were added, and the mixture was extracted with EtOAc (3 × 25 mL). The combined organic phases were washed with brine (40 mL), dried ($MgSO_4$), filtered, and concentrated *in vacuo*. The mixture was purified by column chromatography (30%→40% EtOAc, 70%→60% hexane) to give *alkynone* **1f** (970 mg, 73%) as a pale yellow solid. $R_f$ = 0.23 (30% EtOAc/hexane); m.p. 100-105 °C (hexane); IR 2974, 2224 (C≡N), 1717 (C=O), 1485, 1263, 1069, 758 $cm^{-1}$; $^1H$ NMR (400 MHz, $CDCl_3$) δ 7.62-7.57 (1H, m, Ar**H**), 7.50 (1H, td, $J$ = 7.7, 1.3 Hz, Ar**H**), 7.47-7.43 (1H, m, Ar**H**), 7.37 (1H, td, $J$ = 7.6, 1.3 Hz, Ar**H**), 3.07-2.83 (4H, m, C$\mathbf{H}_2$C$\mathbf{H}_2$), 2.81 (2H, s, C$\mathbf{H}_2$C≡C), 1.22 (3H, s, C$\mathbf{H}_3$); $^{13}C$ NMR

(126 MHz, $CDCl_3$) δ 215.1 (2 × C), 132.5 (CH), 132.5 (CH), 132.3 (CH), 128.3 (CH), 126.6 (C), 117.4 (C), 115.0 (C), 91.5 (C), 78.8 (C), 55.6 (C), 35.7 (2 × $CH_2$), 24.5 ($CH_2$), 20.2 ($CH_3$); HRMS (ESI) Exact mass calcd for $C_{16}H_{14}NO_2$ $[M+H]^+$: 252.1019, found: 252.1007.

**2-Methyl-2-(2-propynyl)-1,3-indandione (S1)**

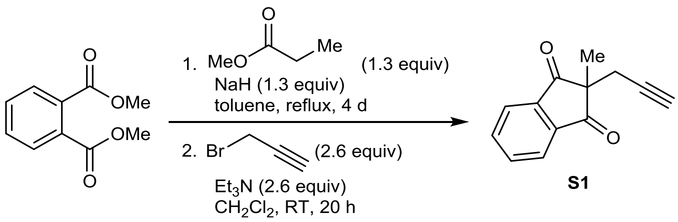


Dimethyl phthalate (4.9 mL, 30 mmol) was added to a two-necked flask under nitrogen containing NaH (60% in mineral oil, 1.56 g, 39.0 mmol) in anhydrous toluene (90 mL). Methyl propionate (3.7 mL, 39 mmol) was added and the mixture was heated at reflux for 2 h. The mixture was cooled to room temperature, NaH (60% in mineral oil, 1.56 g, 39.0 mmol) was added, and the mixture was heated at reflux at 4 days. The mixture was cooled to room temperature and $H_2O$ (10 ml) was added carefully under nitrogen. The mixture was diluted with $H_2O$ (200 mL), and the aqueous layer was separated and washed with hexanes (100 mL). The aqueous layer was acidified (pH 2) with conc. HCl. The mixture was extracted with $CH_2Cl_2$ (3 × 75 mL) and the combined organic phases were dried ($MgSO_4$), filtered, and concentrated *in vacuo* to a volume of ~100 mL. The mixture was cooled to 0 °C, and $Et_3N$ (10.8 mL, 78.0 mmol) and propargyl bromide (80% solution in toluene, 8.2 mL, 78 mmol) were added. The mixture was stirred at room temperature for 20 h. Water (50 mL) and 2 M aqueous HCl (40 ml) were added, and the mixture was extracted with $CH_2Cl_2$ (3 × 50 mL). The combined organic phases were dried ($MgSO_4$), filtered, and concentrated *in vacuo*. The mixture was purified by column chromatography (20% EtOAc/hexane) followed by recrystallization from hexane to give *alkyne* **S1** as a pale yellow solid (948 mg, 16%). $R_f$ = 0.18 (20% EtOAc/hexane); m.p. 87-88 °C (hexane); IR 3256 (C≡**C-H**), 2972, 1705 (C=O), 1595, 1258, 694 $cm^{-1}$; $^1H$ NMR (500 MHz, $CDCl_3$) δ 8.05-8.00 (2H, m, Ar**H**), 7.91-7.86 (2H, m, Ar**H**), 2.69 (2H, d, $J$ = 2.7 Hz, C**H**$_2$), 1.76 (1H, t, $J$ = 2.7 Hz, ≡C**H**), 1.32 (3H, s, C**H**$_3$); $^{13}C$ NMR (126 MHz, $CDCl_3$) δ 202.5 (2 × C), 141.4 (2 × C), 135.9 (2 × CH), 123.5 (2 × CH), 78.6 (C), 71.1 (CH), 52.7 (C), 23.9 ($CH_2$), 19.1 ($CH_3$); HRMS (ESI) Exact mass calcd for $C_{13}H_{11}O_2$ $[M+H]^+$: 199.0754, found: 199.0743.

**2-Methyl-2-(3-phenyl-2-propynyl)-1,3-indandione (1i)**

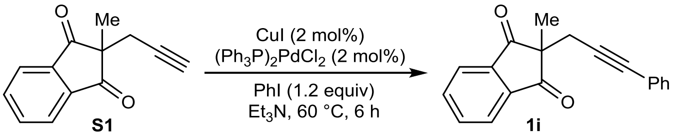


$Et_3N$ (10 mL) was added to a flask containing alkyne **S1** (401 mg, 2.02 mmol), iodobenzene (0.27 mL, 2.4 mmol), $Pd(PPh_3)_2Cl_2$ (28.4 mg, 0.0405 mmol), and CuI (7.7 mg, 0.040 mmol) and the mixture was stirred at 60 °C for 6 h. The reaction was cooled to room temperature, water (20 mL) and saturated aqueous $NH_4Cl$ (5 ml) were added, and the mixture was extracted with EtOAc (3 × 25 mL). The combined organic phases were washed with brine (40 mL), dried ($MgSO_4$), filtered, and concentrated *in vacuo*. The mixture was purified by column chromatography (20% EtOAc/hexane) to give *alkynone* **1i** (402 mg, 72%) as an orange solid. $R_f$ = 0.22 (20% EtOAc/hexane); m.p. 76-78 °C ($Et_2O$/petroleum ether); IR 2907, 1711 (C=O), 1593, 1275, 922, 766, 694 $cm^{-1}$; $^1H$ NMR (500 MHz, $CDCl_3$) δ 8.07-8.01 (2H, m, Ar**H**), 7.91-7.85 (2H, m, Ar**H**), 7.19-7.15 (1H, m, Ar**H**), 7.14-7.09 (2H, m, Ar**H**), 6.92-6.88 (2H, m, Ar**H**), 2.90 (2H, s, C**H**$_2$), 1.37 (3H, s, C**H**$_3$); $^{13}C$ NMR (126 MHz, $CDCl_3$) δ 202.9 (2 × C), 141.7 (2 × C), 135.8 (2 × CH), 131.3 (2 × CH), 127.9 (2 × CH), 127.8 (CH), 123.4 (2 × CH), 122.7 (C), 84.3 (C), 83.7 (C), 53.2 (C), 25.6 ($CH_2$), 18.6 ($CH_3$); HRMS (ESI) Exact mass calcd for $C_{19}H_{15}O_2$ $[M+H]^+$: 275.1067, found: 275.1059.

**2-Phenyl-2-(2-propynyl)-1,3-indandione (S2)**

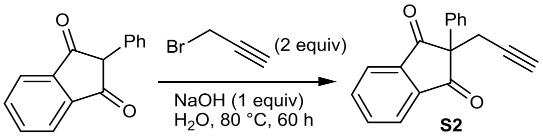


2 M aqueous NaOH (1.1 mL, 2.2 mmol) was added to a slurry of 2-phenyl-1,3-indandione (500 mg, 2.25 mmol) in water (5 mL). When all of the solid had dissolved, forming a bright red solution, propargyl bromide (80% solution in toluene, 0.40 mL, 3.6 mmol) was added. The mixture was stirred at 80 °C for 60 h. The mixture was cooled to room temperature, EtOAc (10 mL) was added, and the mixture was extracted with EtOAc (3 × 10 mL). The combined organic phases were dried ($MgSO_4$), filtered, and concentrated *in vacuo*. The mixture was purified by column chromatography (20% EtOAc/petroleum ether) to give *alkyne* **S2** (543 mg, 93%) as a white solid. $R_f$ = 0.33 (20% EtOAc/petroleum ether); m.p. 136-138 °C (MeOH); IR 3289 (C≡**C-H**), 1706 (C=O), 1592, 1252, 783 $cm^{-1}$; $^1H$ NMR (400 MHz, $CDCl_3$) δ 8.10-8.05 (2H, m, Ar**H**), 7.92-7.86 (2H, m, Ar**H**), 7.43-7.38 (2H, m, Ar**H**), 7.35-7.25 (3H, m, Ar**H**), 3.15 (2H, d, *J* = 2.6 Hz, C**H**$_2$), 1.77 (1H, t, *J* = 2.6 Hz, ≡C**H**); $^{13}C$ NMR (101 MHz, $CDCl_3$) δ 199.8 (C), 142.2 (C), 136.0 (2 × CH), 135.4 (C), 129.0 (2 × CH), 128.2 (CH), 126.7 (2 × CH), 123.7 (2 × CH), 78.8 (C), 70.9 (CH), 60.9 (C), 24.6 ($CH_2$);

HRMS (ESI) Exact mass calcd for $C_{18}H_{12}NaO_2$ $[M+Na]^+$: 283.0730, found: 283.0730.

**2-Phenyl-2-(3-phenyl-2-propynyl)-1,3-indandione (1j)**

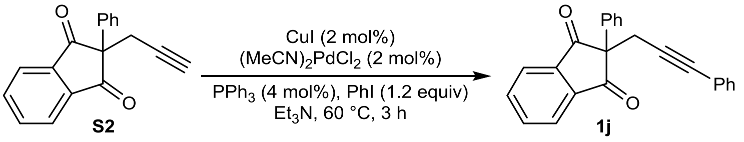


$Et_3N$ (5 mL) was added to a flask containing $(MeCN)_2PdCl_2$ (6.0 mg, 0.023 mmol), $PPh_3$ (12.1 mg, 0.0461 mmol), and CuI (4.4 mg, 0.023 mmol). The mixture was stirred at room temperature for 5 min, alkyne **S2** (300 mg, 1.15 mmol) and iodobenzene (0.15 mL, 1.3 mmol) were added, and the mixture was stirred at 60 °C for 3 h. The reaction was cooled to room temperature, water (10 mL) and saturated aqueous $NH_4Cl$ (5 ml) were added, and the mixture was extracted with EtOAc (3 × 15 mL). The combined organic phases were washed with brine (20 mL), dried ($MgSO_4$), filtered, and concentrated *in vacuo*. The mixture was purified by column chromatography (20% EtOAc/hexane) to give *alkynone* **1j** (232 mg, 60%) as an orange solid. $R_f$ = 0.36 (20% EtOAc/petroleum ether); m.p. 110-112 °C (MeOH); IR 1710 (C=O), 1593, 1489, 1440, 1264 $cm^{-1}$; $^1H$ NMR (400 MHz, $CDCl_3$) 8.12-8.06 (2H, m, Ar**H**), 7.91-7.85 (2H, m, Ar**H**), 7.49-7.45 (2H, m, Ar**H**), 7.38-7.27 (3H, m, Ar**H**), 7.20-7.09 (3H, m, Ar**H**), 6.95-6.91 (2H, m, Ar**H**), 3.38 (2H, s, C**H**$_2$); $^{13}C$ NMR (101 MHz, $CDCl_3$) 200.3 (2 × C), 142.3 (2 × C), 136.0 (2 × CH), 135.5 (C), 131.3 (2 × CH), 128.9 (2 × CH), 128.1 (CH), 127.9 (2 × CH), 127.9 (CH), 126.8 (2 × CH), 123.6 (2 × CH), 122.6 (C), 84.4 (C), 83.5 (C), 61.0 (C), 25.9 ($CH_2$); HRMS (ESI) Exact mass calcd for $C_{24}H_{16}NaO_2$ $[M+Na]^+$: 359.1043, found: 359.1041.

**2-Methyl-2-(3-phenylprop-2-yn-1-yl)cyclohexane-1,3-dione (1k)**

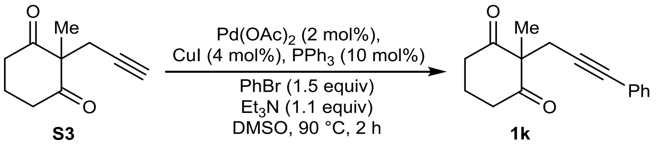


2-Methyl-2-(1-propyn-3-yl)cyclohexane-1,3-dione (**S3**)[3] (1.48 g, 9.01 mmol) was added to a solution of $Pd(OAc)_2$ (40.4 mg, 0.180 mmol), $PPh_3$ (189 mg, 0.721 mmol), CuI (68.6 mg, 0.360 mmol), and $Et_3N$ (1.38 mL, 9.90 mmol) in anhydrous DMSO (20 mL). A solution of 3-bromobenzene (1.41 mL, 13.5 mmol) in anhydrous DMSO (10 mL) was added and the mixture was stirred at 90 °C for 2 h. The reaction was cooled to room temperature, water (50 mL) was added, and the mixture was extracted with $Et_2O$ (50 mL). The organic phase was washed with 10% aqueous HCl (3 × 20 mL), dried ($Na_2SO_4$), filtered, and concentrated *in vacuo*. The mixture was

3. Prepared according to: K. Kato, C. Matsuba, T. Kusakabe, H. Takayama, S. Yamamura, T. Mochida, H. Akita, T. y. A. Peganova, N. V. Vologdin, O. V. Gusev, *Tetrahedron* **2006**, *62*, 9988-9999.

purified by column chromatography (10% EtOAc/hexane) to give *alkynone* **1k** (1.63 g, 75%) as a pale yellow solid. $R_f$ = 0.33 (30% EtOAc/hexane); m.p. 60-65 °C (hexane); IR 2967, 1694 (C=O), 1410, 1315, 1022, 766 cm$^{-1}$; $^1$H NMR (500 MHz, $CDCl_3$) δ 7.35-7.33 (2H, m, Ar**H**), 7.28-7.26 (3H, m, Ar**H**), 2.87 (2H, s, C**H**$_2$C≡C), 2.73 (4H, td, *J* = 7.2, 2.1 Hz, C**H**$_2$CH$_2$C**H**$_2$), 2.01 (2H, qd, *J* = 7.2, 2.1 Hz, CH$_2$C**H**$_2$CH$_2$), 1.36 (3H, s, C**H**$_3$); $^{13}$C NMR (126 MHz, $CDCl_3$) δ 209.3 (2 × C), 131.6 (2 × CH), 128.2 (2 × CH), 128.0 (CH), 123.1 (C), 85.6 (C), 83.0 (C), 64.0 (C), 38.5 (2 × CH$_2$), 26.4 (CH$_2$), 21.7 (CH$_3$), 17.3 (CH$_2$); HRMS (ESI) Exact mass calcd for $C_{16}H_{17}O_2$ [M+H]$^+$: 241.1223, found: 241.1211.

**2-[3-(4-Methoxyphenyl)prop-2-yn-1-yl]-2-methylcyclopentane-1,3-dione (1l)**

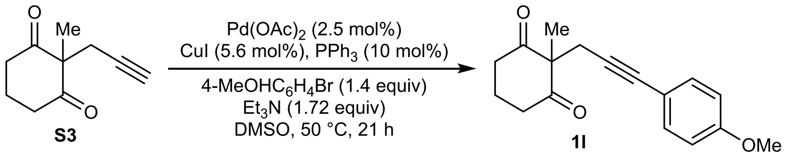


2-Methyl-2-(1-propyn-3-yl)cyclohexane-1,3-dione (**S3**)[3] (1.64 g, 10.0 mmol) was added to a solution of Pd(OAc)$_2$ (56 mg, 0.25 mmol), PPh$_3$ (262 mg, 1.00 mmol), CuI (106 mg, 0.557 mmol), and Et$_3$N (2.4 mL, 17.2 mmol) in anhydrous DMSO (20 mL). A solution of 4-bromoanisole (1.72 mL, 13.7 mmol) in anhydrous DMSO (10 mL) was added and the mixture was stirred at 90 °C for 2 h. The reaction was cooled to room temperature, water (50 mL) was added, and the mixture was extracted with Et$_2$O (50 mL). The organic phase was washed with 10% aqueous HCl (3 × 20 mL), dried (Na$_2$SO$_4$), filtered, and concentrated *in vacuo*. The mixture was purified by column chromatography (10% EtOAc/hexane) to give *alkynone* **1l** (2.21 g, 82%) as a pale yellow solid. $R_f$ = 0.28 (30% EtOAc/hexane); m.p. 70-75 °C (hexane); IR 2695, 1721 (C=O), 1508, 1244, 1032, 841 cm$^{-1}$; 7.30-7.24 (2H, m, Ar**H**), 6.82-6.76 (2H, m, Ar**H**), 3.79 (3H, s, OC**H**$_3$), 2.84 (2H, s, C**H**$_2$C≡C), 2.73 (4H, t, *J* = 3.8 Hz, C**H**$_2$CH$_2$C**H**$_2$), 2.08-1.92 (2H, m, CH$_2$C**H**$_2$CH$_2$), 1.34 (3H, s, CC**H**$_3$); $^{13}$C NMR (126 MHz, $CDCl_3$) δ 209.4 (2 × C), 159.4 (C), 133.0 (2 × CH), 115.2 (C), 113.8 (2 × CH), 83.9 (C), 82.9 (C), 64.0 (C), 55.3 (CH$_3$), 38.6 (2 × CH$_2$), 26.8 (CH$_2$), 21.5 (CH$_3$), 17.3 (CH$_2$); HRMS (ESI) Exact mass calcd for $C_{17}H_{19}O_3$ [M+H]$^+$: 271.1334, found: 271.1329.

**2-[3-(4-Chlorophenyl)prop-2-yn-1-yl]-2-methylcyclopentane-1,3-dione (1m)**

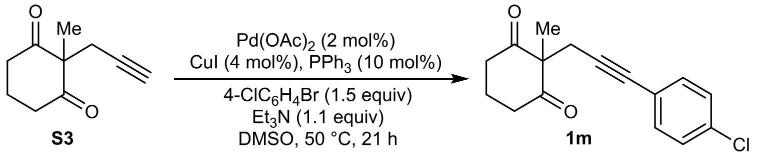


Et$_3$N (21 mL) was added to a flask containing 2-methyl-2-(1-propyn-3-yl)cyclohexane-1,3-dione (**S3**)[3] (650 mg, 3.96 mmol), 4-bromochlorobenzene (1.14 g, 5.94 mmol), PPh$_3$ (41.7 mg, 0.159),

$Pd(OAc)_2$ (17.8 mg, 0.0793 mmol), and CuI (30.2 mg, 0.159 mmol), and the mixture was stirred at 50 °C for 21 h. The reaction was cooled to room temperature, water (20 mL) and saturated aqueous $NH_4Cl$ (5 ml) were added, and the mixture was extracted with EtOAc (3 × 25 mL). The combined organic phases were washed with brine (40 mL), dried ($MgSO_4$), filtered, and concentrated *in vacuo*. The mixture was purified by column chromatography (25% EtOAc/hexane) to give *alkynone* **1m** (512 mg, 63%) as a pale yellow solid. $R_f$ = 0.36 (30% EtOAc/hexane); m.p. 80-85 °C (hexane); IR 2965, 1690 (C=O), 1489, 1317, 1088, 1022, 839 $cm^{-1}$; $^1H$ NMR (500 MHz, $CDCl_3$) δ 7.27-7.23 (4H, m, Ar**H**), 2.86 (2H, s, C**H**$_2$C≡C), 2.79-2.67 (4H, m, C**H**$_2$CH$_2$C**H**$_2$), 2.05-1.97 (2H, m, CH$_2$C**H**$_2$CH$_2$), 1.37 (3H, s, C**H**$_3$); $^{13}C$ NMR (126 MHz, $CDCl_3$) δ 209.0 (2 × C), 134.0 (C), 132.8 (2 × CH), 128.5 (2 × CH), 121.6 (C), 86.8 (C), 81.7 (C), 64.1 (C), 38.3 (2 × $CH_2$), 25.9 ($CH_2$), 22.0 ($CH_3$), 17.3 ($CH_2$); HRMS (ESI) Exact mass calcd for $C_{16}H_{16}{}^{35}ClO_2$ $[M+H]^+$: 275.0839, found: 275.0836.

**1-Methyl-3-methyl-3-(2-propynyl)-1,3-dihydroquinoline-2,4-dione (S5)**

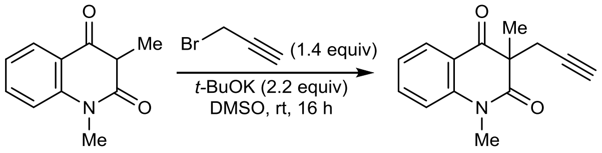


**S4** **S5**

Propargyl bromide (80% solution in toluene, 858 µL, 7.70 mmol) was added to a solution of 1,3-dimethyl-1,2,3,4-tetrahydroquinoline-2,4-dione (**S4**)[4] (1.04 g, 5.50 mmol) and *t*-BuOK (678 mg, 6.04 mmol) in DMSO (50 mL). The mixture was stirred at room temperature for 16 h, diluted with water (50 mL), and extracted with EtOAc (3 × 25 mL). The combined organic phases were dried ($Na_2SO_4$), filtered and concentrated *in vacuo*. The mixture was purified by column chromatography (20% EtOAc/hexane) to give *alkyne* **S5** (650 mg, 52%) as a yellow oil. $R_f$ = 0.33 (25% EtOAc/petroleum ether); IR 3272 (C≡**C-H**), 1698 (C=O), 1656 (C=O), 1375, 1102, 758 $cm^{-1}$; $^1H$ NMR (500 MHz, $CDCl_3$) δ 8.06-8.03 (1H, m, Ar**H**), 7.68-7.63 (1H, m, Ar**H**), 7.27-7.18 (2H, m, Ar**H**), 3.51 (3H, s, NC**H**$_3$), 2.90 (2H, d, $J$ = 2.6 Hz, C**H**$_2$), 1.84 (1H, t, $J$ = 2.6 Hz, ≡C**H**), 1.47 (3H, s, CC**H**$_3$); $^{13}C$ NMR (126 MHz, $CDCl_3$) δ 195.8 (C), 172.4 (C), 143.2 (CH), 136.2 (CH), 128.3 (CH), 123.2 (CH), 120.0 (C), 114.8 (CH), 80.0 (C), 70.3 (CH), 56.8 (C), 29.9 ($CH_3$), 26.2 ($CH_2$), 24.4 ($CH_3$); HRMS (ESI) Exact mass calcd for $C_{14}H_{14}NO_2$ $[M+H]^+$: 228.1025, found: 228.1026.

4. Prepared according to: Dittmer, D. C.; Li, Q.; Avilov, D. V. *J. Org. Chem.* **2005**, *70*, 4682–4686.

**3-[3-(4-Chlorophenyl)-2-propynyl]-1-methyl-3-methyl-1,3-dihydroquinoline-2,4-dione (1n)**

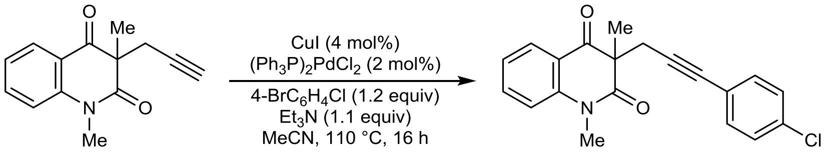


**S5** **1n**

Alkyne **S5** (1.02 g, 4.49 mmol) was added to a solution of $Pd(PPh_3)_2Cl_2$ (63.2 mg, 0.0900 mmol), CuI (34.3 mg, 0.180 mmol), and $Et_3N$ (691 µL, 4.96 mmol) in anhydrous MeCN (15 mL) in a sealed tube. A solution of 4-chlorobromobenzene (1.29 g, 5.41 mmol) in anhydrous MeCN (5 mL) was added, the tube was sealed, and the mixture was stirred at 110 °C for 16 h. The reaction was cooled to room temperature, water (50 mL) was added, and the mixture was extracted with EtOAc (50 mL). The organic phase was washed with aqueous 10% HCl (3 × 20 mL), dried ($Na_2SO_4$), filtered, and concentrated *in vacuo*. The mixture was purified by column chromatography (10% EtOAc/hexane) to give *alkynone* **1n** (0.95 g, 63%) as a yellow solid. $R_f$ = 0.46 (25% EtOAc/petroleum ether); m.p. 96-98 °C (MeOH/hexane); IR 2943, 1687 (C=O), 1651 (C=O), 1472, 1375, 1088, 758 cm$^{-1}$; $^1$H NMR (500 MHz, $CDCl_3$) δ 8.08 (1H, dd, *J* = 7.7, 1.5 Hz, Ar**H**), 7.67-7.62 (1H, m, Ar**H**), 7.23-7.18 (2H, m, Ar**H**), 7.16-7.12 (2H, m, Ar**H**), 7.00-6.96 (2H, m, Ar**H**), 3.50 (3H, s, NC**H**$_3$), 3.08 (1H, d, *J* = 16.2 Hz, C**H**$_2$), 3.02 (1H, d, *J* = 16.2 Hz, C**H**$_2$), 1.55 (3H, s, CC**H**$_3$); $^{13}$C NMR (126 MHz, $CDCl_3$) δ 196.1 (C), 172.6 (C), 143.4 (C), 136.2 (CH), 133.8 (C), 132.7 (2 × CH), 128.3 (2 × CH), 128.2 (CH), 123.2 (CH), 121.5 (C), 120.4 (C), 114.8 (CH), 86.1 (C), 81.8 (C), 56.6 (C), 29.9 ($CH_3$), 29.0 ($CH_2$), 23.3 ($CH_3$); HRMS (ESI) Exact mass calcd for $C_{20}H_{17}{}^{35}ClNO_2$ $[M+H]^+$: 338.0948, found: 338.0940.

**Ethyl 2-oxo-1-(3-phenyl-2-propynyl)cyclopentanecarboxylate (8)**

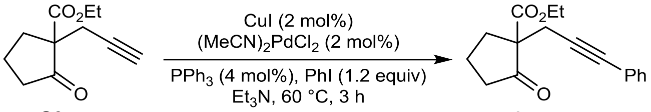


**S6** **8**

$Et_3N$ (40 mL) was added to a flask containing $(MeCN)_2PdCl_2$ (41.5 mg, 0.160 mmol), $PPh_3$ (83.9 mg, 0.320 mmol), and CuI (30.5 mg, 0.160 mmol). The mixture was stirred at room temperature for 5 min, ethyl 2-oxo-1-(2-propynyl)cyclopentanecarboxylate (**S6**)[5] (1.56 g, 8.03 mmol) and iodobenzene (1.1 mL, 9.8 mmol) were added, and the mixture was stirred at 60 °C for 8 h. The reaction was cooled to room temperature, water (50 mL) and saturated aqueous $NH_4Cl$ (25 ml) were added, and the mixture was extracted with EtOAc (3 × 50 mL). The combined organic phases were washed with brine (75 mL), dried ($MgSO_4$), filtered, and concentrated *in vacuo*. The mixture was purified by column chromatography (20% EtOAc/hexane) to give *alkynone* **8** (1.74 g,

5. Prepared according to: F. Barabé, G. v. Bétournay, G. Bellavance, L. Barriault, *Org. Lett.* **2009**, *11*, 4236-4238.

80%) as a yellow oil. $R_f$ = 0.23 (10% EtOAc/petroleum ether); IR 2978, 1751 (C=O), 1726 (C=O), 1227, 1148, 1028 cm$^{-1}$; $^1$H NMR (500 MHz, $CDCl_3$) δ 7.38-7.34 (2H, m, Ar**H**), 7.31-7.27 (3H, m, Ar**H**), 4.20 (2H, q, *J* = 7.1 Hz, OC**H**$_2$), 3.00-2.91 (2H, m, C**H**$_2$C≡C), 2.60-2.47 (2H, m, C**H**$_2$C=O and C**H**$_2$CH$_2$CH$_2$C=O), 2.43-2.28 (2H, m, C**H**$_2$C=O and C**H**$_2$CH$_2$CH$_2$C=O), 2.18-2.04 (2H, m, C**H**$_2$CH$_2$C=O), 1.27 (3H, t, *J* = 7.1 Hz, C**H**$_3$); $^{13}$C NMR (126 MHz, $CDCl_3$) δ 213.9 (C), 170.5 (C), 131.6 (2 × CH), 128.2 (2 × CH), 128.0 (CH), 123.2 (C), 85.3 (C), 82.8 (C), 61.7 ($CH_2$), 59.1 (C), 38.4 ($CH_2$), 32.8 ($CH_2$), 24.2 ($CH_2$), 19.9 ($CH_2$), 14.1 ($CH_3$); HRMS (ESI) Exact mass calcd for $C_{17}H_{19}O_3$ [M+H]$^+$: 271.1329, found: 271.1321.

## 3. Rhodium-Catalyzed Arylative Cyclization of 1a

**(±)-(3a*R*,9b*R*)-5-[(*E*)-Benzylidene)-9b-hydroxy-3a-methyl-1,2,3a,4,5,9b-hexahydro-3*H*-cyclopenta[*a*]naphthalen-3-one (2a), 2-[(*E*)-2,3-diphenylprop-2-en-1-yl]-2-methylcyclopentane-1,3-dione (5), and (±)-5-(diphenylmethylidene)-7-methylcycloheptane-1,4-dione (6)**

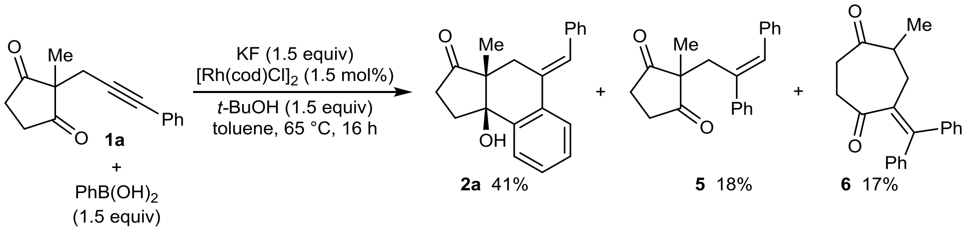


Alkynone **1a** (90.5 mg, 0.400 mmol), phenylboronic acid (73.2 mg, 0.600 mmol), $[Rh(cod)Cl]_2$ (3.0 mg, 0.006 mmol), and KF (34.8 mg, 0.600 mmol) were added to an oven-dried microwave vial. The vial was sealed with a septum-lined cap and purged with nitrogen for 1 h. Anhydrous toluene (4.0 mL) and *t*-BuOH (57 µL, 0.60 mmol) were added, and the mixture was stirred at 65 °C for 16 h. The reaction was cooled to room temperature, water (5 mL) and saturated aqueous $NH_4Cl$ (5 mL) were added, and the mixture was extracted with EtOAc (3 × 10 mL). The combined organic phases were washed with brine (20 mL), dried ($MgSO_4$), filtered, and concentrated *in vacuo*. The mixture was subjected to column chromatography (10% EtOAc/petroleum ether) leading to isolation of *ketone* **6** (20.5 mg, 17%) as a white solid, *ketone* **5** (22.0 mg, 18%) as a pale yellow solid, and *alcohol* **2a** (49.5 mg, 41%) as a white solid.

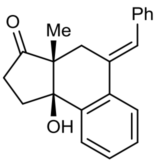


**(±)-(3a*R*,9b*R*)-5-[(*E*)-Benzylidene)-9b-hydroxy-3a-methyl-1,2,3a,4,5,9b-hexahydro-3*H*-cyclopenta[*a*]naphthalen-3-one (2a).** $R_f$ = 0.42 (40% EtOAc/hexane); m.p. 144-145 °C (hexane); IR 3468 (OH), 2936, 1717 (C=O), 1207, 1076, 758, 694 $cm^{-1}$; $^1H$ NMR (500 MHz, $CDCl_3$) δ 7.72 (1H, dd, *J* = 7.7, 1.2 Hz, Ar**H**), 7.68 (1H, dd, *J* = 7.9, 1.2 Hz, Ar**H**), 7.43-7.32 (4H, m, Ar**H**), 7.30-7.25 (3H, m, Ar**H**), 7.24 (1H, d, *J* = 1.4 Hz, C=C**H**), 2.79 (1H, d, *J* = 14.1 Hz, C**H**$_2$C=C), 2.68-2.58 (2H, m, C**H**$_2$C=C and C**H**$_2$C=O), 2.53-2.29 (3H, m, C**H**$_2$C=O and C**H**$_2$COH), 1.83 (1H, d, *J* = 1.6 Hz, O**H**), 1.01 (3H, s, C**H**$_3$); $^{13}C$ NMR (126 MHz, $CDCl_3$) δ 219.9 (C), 140.1 (C), 137.1 (C), 134.3 (C), 132.1 (C), 129.1 (2 × CH), 128.8 (CH), 128.4 (2 × CH), 128.2 (CH), 127.9 (CH), 127.1 (CH), 126.5 (CH), 124.2 (CH), 80.1 (C), 54.1 (C), 35.9 ($CH_2$), 35.2 ($CH_2$), 34.3 ($CH_2$), 13.9 ($CH_3$); HRMS (ESI) Exact mass calcd for $C_{21}H_{21}O_2$ $[M+H]^+$: 305.1542, found: 305.1538.

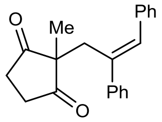


**2-[(*E*)-2,3-Diphenylprop-2-en-1-yl]-2-methylcyclopentane-1,3-dione (5).** $R_f$ = 0.53 (40% EtOAc/hexane); m.p. 85-90 °C ($CH_2Cl_2$/hexane) IR 1742 (C=O), 1210, 929, 730, 702 $cm^{-1}$; $^1H$ NMR (500 MHz, $CDCl_3$) δ 7.54-7.52 (2H, m, Ar**H**), 7.47-7.43 (2H, m, Ar**H**), 7.37-7.33 (3H, m, Ar**H**), 7.32-7.27 (3H, m, Ar**H**), 6.67 (1H, s, C=C**H**), 3.26 (2H, s, C**H**$_2$C=C), 2.48-2.41 (2H, m, C**H**$_2$C**H**$_2$), 1.86-1.80 (2H, m, C**H**$_2$C**H**$_2$), 1.10 (3H, s, C**H**$_3$); $^{13}C$ NMR (126 MHz, $CDCl_3$) δ 216.7 (2 × C), 141.5 (C), 138.3 (C), 137.4 (C), 132.5 (CH), 128.9 (2 × CH), 128.6 (2 × CH), 128.3 (CH), 128.2 (2 × CH), 128.0 (CH), 127.0 (CH), 55.5 (C), 36.7 ($CH_2$), 34.9 (2 × $CH_2$), 22.1 ($CH_3$); HRMS (ESI) Exact mass calcd for $C_{21}H_{20}NaO_2$ $[M+Na]^+$: 327.1356, found: 327.1354.

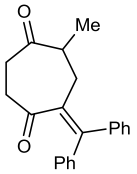


**(±)-5-(Diphenylmethylidene)-7-methylcycloheptane-1,4-dione (6).** $R_f$ = 0.62 (40% EtOAc/hexane); m.p. 110-120 °C ($CH_2Cl_2$/hexane); IR 1708 (C=O), 1215, 760, 702, 699 $cm^{-1}$; $^1H$ NMR (500 MHz, $CDCl_3$) δ 7.39-7.23 (6H, m, Ar**H**), 7.20-7.17 (2H, m, Ar**H**), 7.11-7.08 (2H, m, Ar**H**), 2.85-2.64 (6H, m, C**H**$_2$C**H**$_2$ and C**H**$_2$CH), 2.29-2.20 (1H, m, C**H**CH$_3$), 0.99 (3H, d, $J$ = 6.4 Hz, CHC**H**$_3$); $^{13}C$ NMR (126 MHz, $CDCl_3$) δ 211.5 (C), 207.5 (C), 144.6 (C), 140.8 (C), 139.2 (C), 138.3 (C), 128.9 (2 × CH), 128.6 (2 × CH), 128.5 (2 × CH), 128.3 (2 × CH), 127.8 (CH), 127.8 (CH), 45.2 (CH), 38.9 ($CH_2$), 37.6 ($CH_2$), 34.4 ($CH_2$), 15.6 ($CH_3$); HRMS (ESI) Exact mass calcd for $C_{21}H_{21}O_2$ $[M+H]^+$: 305.1536, found: 305.1527.

# 4. Racemic Iridium Catalyzed Arylative Cyclizations

## General Procedure

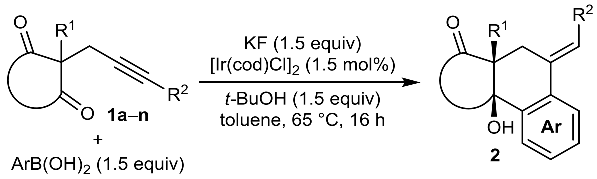


The appropriate alkynone (0.40 mmol), the appropriate arylboronic acid (0.60 mmol), [Ir(cod)Cl]$_2$ (4.0 mg, 0.0060 mmol), and KF (34.8 mg, 0.600 mmol) were added to an oven-dried microwave vial. The vial was sealed with a septum-lined cap and purged with nitrogen for 1 h. Anhydrous toluene (4.0 mL) and *t*-BuOH (57 µL, 0.60 mmol) were added and the mixture was stirred at 65 °C for 16 h. The reaction was cooled to room temperature, water (5 mL) and saturated aqueous $NH_4Cl$ (5 mL) were added, and the mixture was extracted with EtOAc (3 × 10 mL). The combined organic phases were washed with brine (20 mL), dried ($MgSO_4$), filtered, and concentrated *in vacuo*. The mixture was purified by column chromatography to give the arylative cyclization product.

**(±)-(3a*R*,9b*R*)-5-[(*E*)-Benzylidene)-9b-hydroxy-3a-methyl-1,2,3a,4,5,9b-hexahydro-3*H*-cyclopenta[*a*]naphthalen-3-one (2a)** and **(±)-2-[(*E*)-3-{(3a*S*,9b*S*)-5-[(*E*)-benzylidene]-9b-hydroxy-3a-methyl-3-oxo-2,3,3a,4,5,9b-hexahydro-1*H*-cyclopenta[*a*]naphthalen-6-yl}-3-phenylallyl]-2-methylcyclopentane-1,3-dione (7)**

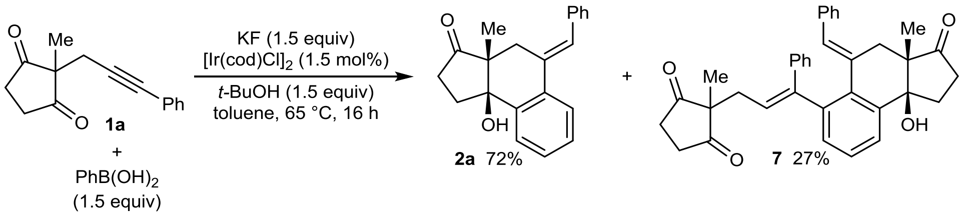


Alkynone **1a** (90.5 mg, 0.400 mmol), phenylboronic acid (73.2 mg, 0.600 mmol), [Ir(cod)Cl]$_2$ (4.0 mg, 0.0060 mmol), and KF (34.8 mg, 0.600 mmol) were added to an oven-dried microwave vial. The vial was sealed with a septum-lined cap and purged with nitrogen for 1 h. Anhydrous toluene (4.0 mL) and *t*-BuOH (57 µL, 0.60 mmol) were added, and the mixture was stirred at 65 °C for 16 h. The reaction was cooled to room temperature, water (5 mL) and saturated aqueous $NH_4Cl$ (5 mL) were added, and the mixture was extracted with EtOAc (3 × 10 mL). The combined organic phases were washed with brine (20 mL), dried ($MgSO_4$), filtered, and concentrated *in vacuo*. The mixture was subjected to column chromatography (40% EtOAc/petroleum ether) leading to the isolation of *arylative cyclization product* **2a** (88.0 mg, 72%) as a white solid and *2:1 adduct* **7** (28.4 mg, 27%) as a white solid.

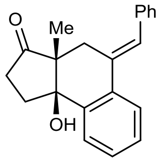


**(±)-(3a*R*,9b*R*)-5-[(*E*)-Benzylidene)-9b-hydroxy-3a-methyl-1,2,3a,4,5,9b-hexahydro-3*H*-cyclopenta[*a*]naphthalen-3-one (2a).** Data as described above.

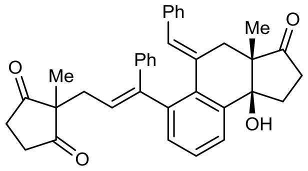


**(±)-2-[(*E*)-3-{(3a*S*,9b*S*)-5-[(*E*)-Benzylidene]-9b-hydroxy-3a-methyl-3-oxo-2,3,3a,4,5,9b-hexahydro-1*H*-cyclopenta[*a*]naphthalen-6-yl}-3-phenylallyl]-2-methylcyclopentane-1,3-dione (7).** $R_f$ = 0.13 (40% EtOAc/hexane); m.p. 206-208 °C ($CH_2Cl_2/Et_2O$); IR 3438 (OH), 2926, 1736 (C=O), 1721 (C=O), 1445, 1205, 1070 $cm^{-1}$; $^1$H NMR (400 MHz, $CDCl_3$) δ 7.68 (1H, dd, *J* = 7.6, 1.3 Hz, Ar**H**), 7.47 (1H, dd, *J* = 7.5, 1.4 Hz, Ar**H**), 7.41 (1H, t, *J* = 7.6 Hz, Ar**H**), 7.30-7.13 (7H, m, Ar**H**), 6.94 (1H, s, C=C**H**Ph), 6.78-6.84 (4H, m, Ar**H**), 5.86 (1H, t, *J* = 7.5 Hz, $CH_2$C**H**=CPh), 2.72-2.42 (7H, m, O=CC**H**$_2$C**H**$_2$C=O and C**H**$_2$CH=C and C**H**$_2$CH$_2$COH), 2.33-1.99 (5H, m, C**H**$_2$CH$_2$COH and C**H**$_2$COH and C**H**$_2$C=CH), 1.82 (1H, d, *J* = 1.4 Hz, O**H**), 1.14 (3H, s, C**H**$_3$C(C=O)$_2$), 0.83 (3H, s, C**H**$_3$CCOH); $^{13}$C NMR (101 MHz, $CDCl_3$) δ 219.7 (C), 215.1 (2 × C), 147.8 (C), 142.4 (C), 142.1 (C), 139.4 (C), 136.9 (C), 134.8 (C), 133.9 (CH), 132.3 (CH), 132.1 (C), 129.7 (2 × CH), 129.1 (2 × CH), 128.1 (2 × CH), 127.8 (CH), 127.6 (2 × CH), 127.0 (CH), 126.9 (CH), 125.2 (CH), 123.1 (CH), 80.7 (C), 57.1 (C), 54.6 (C), 35.8 ($CH_2$), 35.1 ($CH_2$), 34.9 ($CH_2$), 34.8 ($CH_2$), 34.73 ($CH_2$), 34.69 ($CH_2$), 16.7 ($CH_3$), 15.1 ($CH_3$); HRMS (ESI) Exact mass calcd for $C_{36}H_{34}NaO_4$ $[M+Na]^+$: 554.2349, found: 553.2325.

Slow diffusion of $Et_2O$ into a solution of **7** in $CH_2Cl_2$ provided crystals that were suitable for X-ray diffraction:

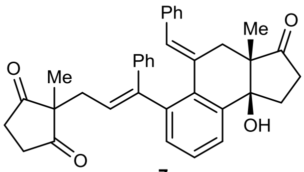


**7**
CCDC 979585

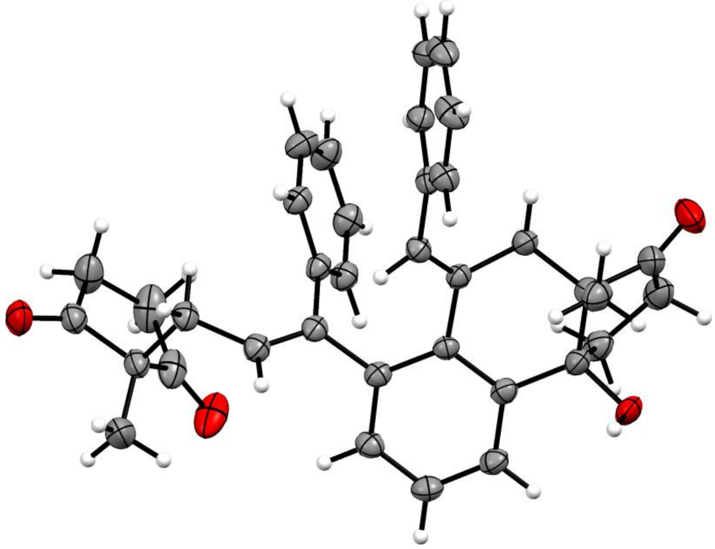

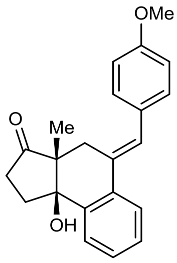

**(±)-(3a*R*,9b*R*)-9b-Hydroxy-5-[(*E*)-4-methoxybenzylidene]-3a-methyl-1,2,3a,4,5,9b-hexahydro-3*H*-cyclopenta[*a*]naphthalen-3-one (2b).** According to the general procedure, reaction of alkynone **1b** (102 mg, 0.400 mmol) and phenylboronic acid (73.2 mg, 0.600 mmol) gave, after purification by column chromatography (40% EtOAc/petroleum ether), *alcohol* **2b** (82.4 mg, 62%) as a white solid. $R_f$ = 0.31 (30% EtOAc/hexane); m.p. 130-140 °C (*i*-PrOH); IR 3289 (OH), 2931, 1719 (C=O), 1508, 1250, 1022, 756 cm$^{-1}$; $^1$H NMR (500 MHz, $CDCl_3$) δ 7.71 (1H, dd, *J* = 7.7, 1.5 Hz, Ar**H**), 7.66 (1H, dd, *J* = 7.8, 1.4 Hz, Ar**H**), 7.38 (1H, ddd, *J* = 7.7, 7.5, 1.4 Hz, Ar**H**) 7.33 (1H, ddd, *J* = 7.8, 7.5, 1.5 Hz, Ar**H**), 7.24-7.19 (2H, m, Ar**H**), 7.17 (1H, d, *J* = 1.6 Hz, C=C**H**), 6.93-6.88 (2H, m, Ar**H**), 3.85 (3H, s, OC**H**$_3$), 2.80 (1H, d, *J* = 14.1 Hz, C**H**$_2$C=C), 2.66-2.58 (2H, m, C**H**$_2$C=C and C**H**$_2$C=O), 2.52-2.29 (3H, m, C**H**$_2$C=O and C**H**$_2$COH), 1.83 (1H, d, *J* = 2.1 Hz, O**H**), 1.01 (3H, s, C**H**$_3$); $^{13}$C NMR (126 MHz, $CDCl_3$) δ 220.0 (C), 158.7 (C), 139.9 (C), 134.5 (C), 130.7 (C), 130.4 (2 × CH), 129.7 (C), 128.5 (CH), 128.1 (CH), 127.6 (CH), 126.5 (CH), 124.1 (CH), 113.9 (2 × CH), 80.1 (C), 55.3 ($CH_3$), 54.1 (C), 35.9 ($CH_2$), 35.2 ($CH_2$), 34.3 ($CH_2$), 13.9 ($CH_3$); HRMS (ESI) Exact mass calcd for $C_{22}H_{26}NO_3$ [M+NH$_4$]$^+$: 352.1913, found: 352.1907.

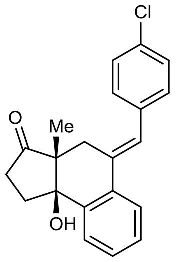

**(±)-(3a*R*,9b*R*)-9b-Hydroxy-5-[(*E*)-4-chlorobenzylidene]-3a-methyl-1,2,3a,4,5,9b-hexahydro-3*H*-cyclopenta[*a*]naphthalen-3-one (2c).** According to the general procedure, reaction of alkynone **1c** (104 mg, 0.400 mmol) and phenylboronic acid (73.2 mg, 0.600 mmol) gave, after purification by column chromatography (30% EtOAc/petroleum ether), *alcohol* **2c** (96.2 mg, 71%) as a pale yellow solid. $R_f$ = 0.31 (30% EtOAc/hexane); m.p. 187-188 °C ($Et_2O$/hexane); IR 3451 (OH), 2957, 1717 (C=O), 1231, 1082, 858, 770 cm$^{-1}$; $^1$H NMR (500 MHz, $CDCl_3$) δ 7.73 (1H, dd, *J* = 7.8, 1.3 Hz, Ar**H**), 7.66 (1H, dd, *J* = 7.9, 1.2 Hz, Ar**H**), 7.41 (1H, ddd, *J* = 7.9, 7.4, 1.3 Hz, Ar**H**), 7.37-7.32 (3H, m, Ar**H**), 7.23-7.19 (2H, m, Ar**H**), 7.15 (1H, d, *J* = 1.1 Hz, C=C**H**), 2.70 (1H, d, *J* = 14.1 Hz, C**H**$_2$C=C), 2.67-2.57 (2H, m, C**H**$_2$C=C and C**H**$_2$C=O), 2.50-2.39 (2H, m, C**H**$_2$C=O and C**H**$_2$COH), 2.33 (1H, dt, *J* = 13.6, 9.7 Hz, C**H**$_2$COH), 1.84 (1H, s, O**H**), 1.01 (3H, s, C**H**$_3$); $^{13}$C NMR (126 MHz, $CDCl_3$) δ 219.6 (C), 140.2 (C), 135.5 (C), 134.0 (C), 133.0 (C), 132.9 (C), 130.4 (2 × CH), 129.0 (CH), 128.6 (2 × CH), 128.2 (CH), 126.7 (CH), 126.5 (CH), 124.2 (CH), 80.0 (C), 54.1 (C), 35.9 ($CH_2$), 35.2 ($CH_2$), 34.2 ($CH_2$), 14.1 ($CH_3$); HRMS (ESI) Exact mass calcd for $C_{21}H_{19}{}^{35}ClNaO_2$ [M+Na]$^+$: 361.0966, found: 361.0957.

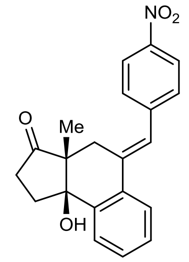


**(±)-(3a*R*,9b*R*)-9b-Hydroxy-5-[(*E*)-4-nitrobenzylidene]-3a-methyl-1,2,3a,4,5,9b-hexahydro-3*H*-cyclopenta[*a*]naphthalen-3-one (2d).** According to the general procedure, reaction of 2-methyl-2-[3-(4-nitrophenyl)prop-2-yn-1-yl]cyclopentane-1,3-dione (**1d**)[2] (108 mg, 0.400 mmol) and phenylboronic acid (73.2 mg, 0.600 mmol) gave, after purification by column chromatography (40% EtOAc/petroleum ether), *alcohol* **2d** (128 mg, 92%) as a yellow solid. $R_f$ = 0.31 (40% EtOAc/hexane); m.p. 186-187 °C (EtOAc/hexane); IR 3563 (OH), 2984, 1732 (C=O), 1591, 1508, 1339, 758 cm$^{-1}$; $^1$H NMR (500 MHz, $CDCl_3$) δ 8.28-8.22 (2H, m, Ar**H**), 7.75 (1H, dd, *J* = 7.8, 1.3 Hz, Ar**H**), 7.67 (1H, dd, *J* = 8.0, 1.1 Hz, Ar**H**), 7.49-7.41 (3H, m, Ar**H**), 7.40-7.35 (1H, m, Ar**H**), 7.21 (1H, s, C=C**H**), 2.71 (1H, dd, *J* = 14.0, 1.9 Hz, C$\mathbf{H}_2$C=C), 2.68-2.58 (2H, m, C$\mathbf{H}_2$C=C and C$\mathbf{H}_2$C=O), 2.47-2.31 (3H, m, C$\mathbf{H}_2$C=O and C$\mathbf{H}_2$COH), 1.87 (1H, d, *J* = 1.9 Hz, O**H**), 1.02 (3H, s, C$\mathbf{H}_3$); $^{13}$C NMR (126 MHz, $CDCl_3$) δ 219.1 (C), 146.5 (C), 143.9 (C), 140.6 (C), 136.0 (C), 133.6 (C), 129.9 (2 × CH), 129.8 (CH), 128.4 (CH), 126.9 (CH), 125.4 (CH), 124.4 (CH), 123.8 (2 × CH), 79.7 (C), 54.2 (C), 35.9 ($CH_2$), 35.3 ($CH_2$), 34.3 ($CH_2$), 14.5 ($CH_3$); HRMS (ESI) Exact mass calcd for $C_{21}H_{19}NNaO_4$ [M+Na]$^+$: 372.1212, found: 372.1202.

Cooling a solution of **2d** in EtOAc/hexane in a freezer provided crystals that were suitable for X-ray diffraction:

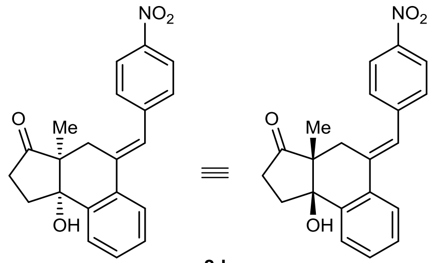


**2d**
CCDC 979586

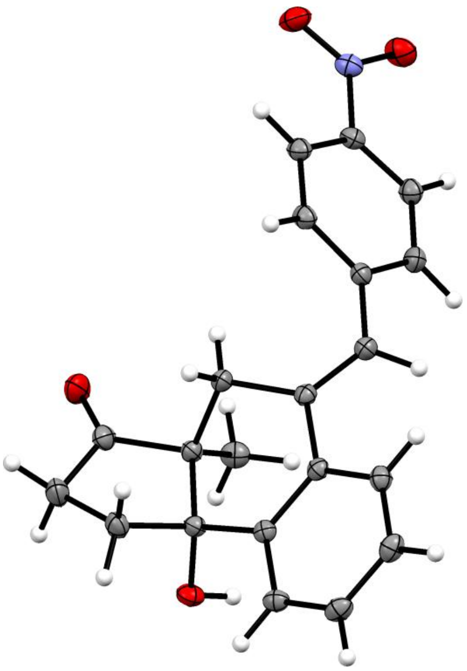

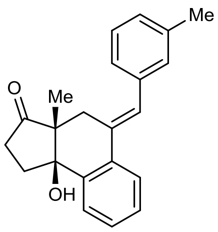


**(±)-(3a*R*,9b*R*)-9b-Hydroxy-5-[(*E*)-3-methylbenzylidene]-3a-methyl-1,2,3a,4,5,9b-hexahydro-3*H*-cyclopenta[*a*]naphthalen-3-one (2e).** According to the general procedure, reaction of alkynone **1e** (96.0 mg, 0.400 mmol) and phenylboronic acid (73.2 mg, 0.600 mmol) gave, after purification by column

chromatography (10% EtOAc/hexane), *alcohol* **2e** (91.1 mg, 72%) as a white solid. $R_f$ = 0.29 (30% EtOAc/hexane); m.p. 110-120 °C (*i*-PrOH); IR 3468 (OH), 2953, 1717 (C=O), 1450, 1076, 959, 694 $cm^{-1}$; $^1$H NMR (500 MHz, $CDCl_3$) δ 7.74 (1H, d, *J* = 1.5 Hz, Ar**H**), 7.69 (1H, dd, *J* = 7.9, 1.2 Hz, Ar**H**), 7.41 (1H, ddd, *J* = 7.7, 7.4, 1.3 Hz, Ar**H**), 7.36 (1H, ddd, *J* = 7.9, 7.4, 1.5 Hz, Ar**H**), 7.29-7.24 (1H, m, Ar**H**), 7.22 (1H, d, *J* = 1.4 Hz, C=C**H**), 7.13-7.07 (3H, m, Ar**H**), 2.81 (1H, d, *J* = 14.1 Hz, C**H**$_2$C=C), 2.69-2.59 (2H, m, C**H**$_2$C=C and C**H**$_2$C=O), 2.51 (1H, ddd, *J* = 19.0, 9.0, 2.4 Hz, C**H**$_2$C=O), 2.43 (1H, ddd, *J* = 13.6, 9.2, 2.4 Hz, C**H**$_2$COH), 2.40-2.26 (1H, m, C**H**$_2$COH), 2.37 (3H, s, ArC**H**$_3$), 1.86 (1H, s, O**H**), 1.03 (3H, s, CC**H**$_3$); $^{13}$C NMR (126 MHz, $CDCl_3$) δ 220.0 (C), 140.1 (C), 137.9 (C), 137.0 (C), 134.3 (C), 131.9 (C), 129.8 (CH), 128.7 (CH), 128.3 (CH), 128.1 (CH), 128.1 (CH), 127.9 (CH), 126.5 (CH), 126.2 (CH), 124.1 (CH), 80.1 (C), 54.2 (C), 35.9 ($CH_2$), 35.2 ($CH_2$), 34.4 ($CH_2$), 21.5 ($CH_3$), 13.8 ($CH_3$); HRMS (ESI) Exact mass calcd for $C_{22}H_{26}NO_2$ $[M+NH_4]^+$: 336.1964, found: 336.1958.

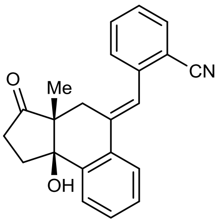


**(±)-2-[(*E*)-{(3a*R*,9b*R*)-9b-Hydroxy-3a-methyl-3-oxo-1,2,3,3a,4,9b-hexahydro-5*H*-cyclopenta[*a*]naphthalen-5-ylidene}methyl]benzonitrile (2f).** In a modification of the general procedure, reaction of alkynone **1f** (101 mg, 0.400 mmol), phenylboronic acid (73.2 mg, 0.600 mmol), and $[Ir(cod)Cl]_2$ (6.7 mg, 0.010 mmol) gave, after purification by column chromatography (10% EtOAc/$CH_2Cl_2$), *alcohol* **2f** (114 mg, 86%) as a pale yellow solid. $R_f$ = 0.38 (10% EtOAc/$CH_2Cl_2$); m.p. 138-139 °C (MeOH); IR 3420 (OH), 2938, 2228 (C≡N), 1717 (C=O), 1086, 966, 760 $cm^{-1}$; $^1$H NMR (500 MHz, $CDCl_3$) δ 7.74 (1H, dd, *J* = 7.8, 1.1 Hz, Ar**H**), 7.72-7.68 (2H, m, Ar**H**), 7.60 (1H, td, *J* = 7.7, 1.2 Hz, Ar**H**), 7.45 (1H, ddd, *J* = 7.8, 7.6, 1.3 Hz, Ar**H**), 7.43-7.35 (3H, m, Ar**H**), 7.28 (1H, d, *J* = 1.5 Hz, C=C**H**), 2.69-2.58 (2H, m, C**H**$_2$C=C and C**H**$_2$C=O), 2.52-2.40 (3H, m, C**H**$_2$C=C and C**H**$_2$C=O and C**H**$_2$COH), 2.39-2.30 (1H, m, C**H**$_2$COH), 1.89 (1H, d, *J* = 2.1 Hz, O**H**), 1.03 (3H, s, C**H**$_3$); $^{13}$C NMR (126 MHz, $CDCl_3$) δ 219.7 (C), 140.9 (C), 140.6 (C), 136.8 (C), 133.3 (C), 133.0 (CH), 132.7 (CH), 129.9 (CH), 129.8 (CH), 128.4 (CH), 127.6 (CH), 126.7 (CH), 124.7 (CH), 123.6 (CH), 118.0 (C), 112.7 (C), 80.0 (C), 54.3 (C), 35.9 ($CH_2$), 35.4 ($CH_2$), 34.7 ($CH_2$), 14.2 ($CH_3$); HRMS (ESI) Exact mass calcd for $C_{22}H_{19}NNaO_2$ $[M+Na]^+$: 352.1308, found: 352.1306.

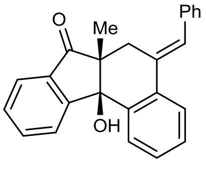


**(±)-(6a*R*,11b*S*)-5-[(*E*)-Benzylidene]-11b-hydroxy-6a-methyl-5,6,6a,11b-tetrahydro-7*H*-benzo[*c*]fluoren-7-one (2i).** According to the general procedure, reaction of alkynone **1i** (110 mg, 0.400 mmol) and phenylboronic acid (73.2 mg, 0.600 mmol) gave, after purification by column chromatography (30% EtOAc/hexane), *alcohol* **2i**

(97.5 mg, 67%) as a pale yellow solid. $R_f$ = 0.31 (30% EtOAc/hexane); m.p. 70-71 °C (hexane); IR 3422 (OH), 2924, 1701 (C=O), 1600, 1449, 1294, 1034, 756, 698 cm$^{-1}$; $^1$H NMR (500 MHz, $CDCl_3$) δ 8.13 (1H, dd, *J* = 7.9, 1.2 Hz, Ar**H**), 7.85-7.82 (1H, m, Ar**H**), 7.73-7.69 (1H, m, Ar**H**), 7.63 (1H, td, *J* = 7.6, 1.2 Hz, Ar**H**), 7.55 (1H, dd, *J* = 7.9, 1.2 Hz, Ar**H**), 7.49-7.27 (8H, m, Ar**H**), 6.98 (1H, s, C=C**H**), 3.20 (1H, dd, *J* = 13.7, 0.9 Hz, C$\mathbf{H}_2$), 2.60 (1H, dd, *J* = 13.7, 1.4 Hz, C$\mathbf{H}_2$), 2.46 (1H, s, O**H**), 1.30 (3H, s, C$\mathbf{H}_3$); $^{13}$C NMR (126 MHz, $CDCl_3$) δ 206.9 (C), 156.0 (C), 139.0 (C), 137.3 (C), 136.6 (C), 135.2 (CH), 133.5 (C), 133.2 (C), 129.3 (CH), 129.1 (2 × CH), 128.5 (CH), 128.3 (2 × CH), 128.2 (CH), 127.7 (CH), 127.3 (CH), 127.0 (CH), 125.10 (CH), 125.05 (CH), 123.9 (CH), 78.4 (C), 56.4 (C), 34.8 ($CH_2$), 19.0 ($CH_3$); HRMS (ESI) Exact mass calcd for $C_{25}H_{21}O_2$ $[M+H]^+$: 353.1536, found: 353.1516.

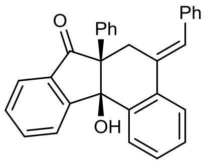


**(±)-(6a*S*,11b*S*)-5-[(*E*)-Benzylidene]-11b-hydroxy-6a-phenyl-5,6,6a,11b-tetrahydro-7*H*-benzo[*c*]fluoren-7-one (2j).** In a modification of the general procedure, reaction of alkynone **1j** (134 mg, 0.400 mmol), phenylboronic acid (73.2 mg, 0.600 mmol), and $[Ir(cod)Cl]_2$ (6.7 mg, 0.010 mmol) gave, after purification by column chromatography (25% EtOAc/petroleum ether), *alcohol* **2j** (104 mg, 63%) as a pale yellow solid. $R_f$ = 0.24 (25% EtOAc/petroleum ether); m.p. 86-88 °C (MeOH); IR 3475 (OH) 1717 (C=O), 1601, 1495, 1288, 1042 cm$^{-1}$; $^1$H NMR (400 MHz, $CDCl_3$) δ 8.12 (1H, dd, *J* = 7.9, 0.9 Hz, Ar**H**), 7.79 (1H, d, *J* = 7.6 Hz, Ar**H**), 7.71 (1H, d, *J* = 7.8 Hz, Ar**H**), 7.65-7.60 (1H, m, Ar**H**), 7.54 (1H, dd, *J* = 7.8, 0.8 Hz, Ar**H**), 7.47-7.41 (2H, m, Ar**H**), 7.40-7.21 (11 H, m, Ar**H**), 6.93 (1H, s, C=C**H**), 3.81 (1H, d, *J* = 13.7 Hz, C$\mathbf{H}_2$), 3.16 (1H, dd, *J* = 13.7, 1.7 Hz, C$\mathbf{H}_2$), 2.08 (1H, s, O**H**); $^{13}$C NMR (101 MHz, $CDCl_3$) δ 205.1 (C), 157.4 (C), 139.6 (C), 139.1 (C), 137.1 (C), 136.9 (C), 135.8 (CH), 134.6 (C), 133.7 (C), 129.3 (CH), 129.0 (2 × CH), 128.7 (2 × CH), 128.5 (CH) 128.5 (2 × CH), 128.3 (2 × CH), 128.1 (CH), 127.7 (CH), 127.4 (CH), 127.0 (2 × CH), 125.5 (CH), 125.3 (CH), 123.4 (CH), 78.4 (C), 64.5 (C), 34.2 ($CH_2$); HRMS (ESI) Exact mass calcd for $C_{30}H_{24}NaO_2$ $[M+Na]^+$: 437.1512, found: 437.1513.

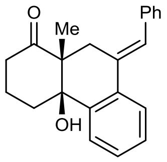


**(±)-(4a*R*,10a*R*)-9-[(*E*)-Benzylidene]-4a-hydroxy-10a-methyl-3,4,4a,9,10,10a-hexahydrophenanthren-1(2*H*)-one (2k).** According to the general procedure, reaction of alkynone **1k** (96.0 mg, 0.400 mmol) and phenylboronic acid (73.2 mg, 0.600 mmol) gave, after purification by column chromatography (10% EtOAc/hexane), *alcohol* **2k** (90.4 mg, 71%) as a white solid. $R_f$ = 0.29 (30% EtOAc/hexane); m.p. 170-171 °C (MeOH); IR 3503 (OH), 2934, 1686 (C=O), 1167, 982, 754, 696 cm$^{-1}$; $^1$H NMR (500 MHz, $CDCl_3$) δ 7.74 (1H, dd, *J* = 7.7, 1.5 Hz, Ar**H**), 7.64 (1H, dd, *J* = 7.6, 1.5 Hz, Ar**H**), 7.40-

7.25 (6H, m, Ar**H**), 7.24 (1H, d, *J* = 1.4 Hz, C=C**H**), 3.20 (1H, dd, *J* = 15.0, 2.0 Hz, C**H**$_2$C=C), 2.81 (1H, dd, *J* = 15.0, 0.9 Hz, C**H**$_2$C=C), 2.70 (1H, ddd, *J* = 14.9, 13.0, 6.6 Hz, C**H**$_2$C=O), 2.46-2.37 (1H, m, C**H**$_2$C=O), 2.34-2.13 (2H, m, C**H**$_2$COH), 2.02-1.94 (2H, m, C**H**$_2$CH$_2$COH and O**H**), 1.93-1.85 (1H, m, C**H**$_2$CH$_2$COH), 1.02 (3H, s, C**H**$_3$); $^{13}$C NMR (126 MHz, CDCl$_3$) δ 213.7 (C), 142.1 (C), 137.2 (C), 133.9 (C), 132.0 (C), 129.2 (2 × CH), 128.5 (CH), 128.4 (2 × CH), 127.8 (CH), 127.08 (CH), 127.06 (CH), 125.3 (CH), 123.7 (CH), 78.4 (C), 53.3 (C), 36.9 (CH$_2$), 36.8 (CH$_2$), 36.7 (CH$_2$), 20.9 (CH$_2$), 15.9 (CH$_3$); HRMS (ESI) Exact mass calcd for $C_{22}H_{23}O_2$ [M+H]$^+$: 319.1698, found: 319.1696.

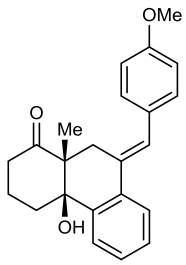


**(±)-(4a*R*,10a*R*)-4a-Hydroxy-9-[(*E*)-4-methoxybenzylidene]-10a-methyl-3,4,4a,9,10,10a-hexahydrophenanthren-1(2*H*)-one (2l).** According to the general procedure, reaction of alkynone **1l** (108 mg, 0.400 mmol) and phenylboronic acid (73.2 mg, 0.600 mmol) gave, after purification by column chromatography (10% EtOAc/hexane), *alcohol* **2l** (80.7 mg, 58%) as a white solid. $R_f$ = 0.25 (30% EtOAc/hexane); m.p. 133-134 °C (Et$_2$O/hexane); IR 3466 (OH), 2936, 1717 (C=O), 1452, 1238, 1034, 758, 694 cm$^{-1}$; $^1$H NMR (400 MHz, CDCl$_3$) δ 7.73-7.70 (1H, m, Ar**H**), 7.64-7.61 (1H, m, Ar**H**), 7.36-7.29 (2H, m, Ar**H**), 7.29-7.24 (2H, m, Ar**H**), 7.17 (1H, d, *J* = 1.3 Hz, C=C**H**), 6.93-6.88 (2H, m, Ar**H**), 3.84 (3H, s, OC**H**$_3$), 3.19 (1H, dd, *J* = 15.1, 1.9 Hz, C**H**$_2$C=C), 2.80 (1H, dd, *J* = 15.1, 1.2 Hz, C**H**$_2$C=C), 2.76-2.66 (1H, m, C**H**$_2$C=O), 2.46-2.40 (1H, m, C**H**$_2$C=O), 2.33-2.14 (2H, m, C**H**$_2$COH), 1.99-1.86 (3H, m, CH$_2$C**H**$_2$CH$_2$ and O**H**), 1.01 (3H, s, CC**H**$_3$); $^{13}$C NMR (126 MHz, CDCl$_3$) δ 213.8 (C), 158.6 (C), 141.9 (C), 134.3 (C), 130.6 (2 × CH), 130.5 (C), 129.8 (C), 128.2 (CH), 127.7 (CH), 126.8 (CH), 125.1 (CH), 123.6 (CH), 113.9 (2 × CH), 78.4 (C), 55.3 (CH$_3$), 53.3 (C), 36.9 (CH$_2$), 36.8 (CH$_2$), 36.6 (CH$_2$), 20.9 (CH$_2$), 15.9 (CH$_3$); HRMS (ESI) Exact mass calcd for $C_{23}H_{25}O_3$ [M+H]$^+$: 366.2069, found: 366.2066.

Slow evaporation of a solution of **2l** in EtOAc/hexane provided crystals that were suitable for X-ray diffraction:

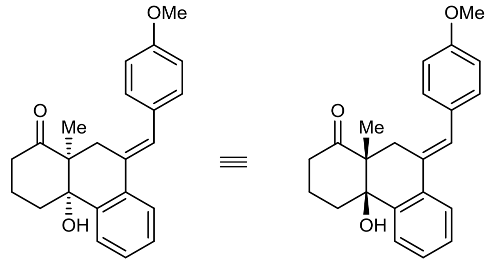


**2l**
CCDC 979587

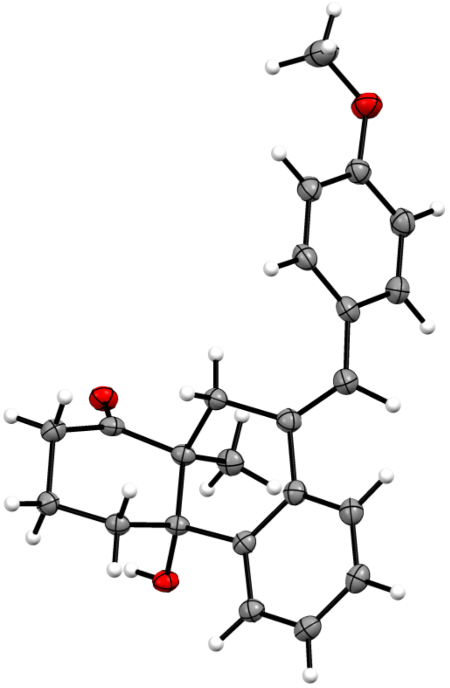

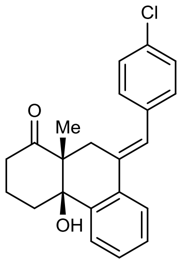


**(±)-(4a*R*,10a*R*)-4a-Hydroxy-9-[(*E*)-4-chlorobenzylidene]-10a-methyl-3,4,4a,9,10,10a-hexahydrophenanthren-1(2*H*)-one (2m).** According to the general procedure, reaction of alkynone **1m** (110 mg, 0.400 mmol) and phenylboronic acid (73.2 mg, 0.600 mmol) gave, after purification by column chromatography (10% EtOAc/cyclohexane), *alcohol* **2m** (112.6 mg, 80%) as a white solid. $R_f$ = 0.36 (30% EtOAc/hexane); m.p. 166-167 °C (MeOH); IR 3497 (OH), 2953, 1682 (C=O), 1489, 1190, 870, 756 cm$^{-1}$; $^1$H NMR (500 MHz, $CDCl_3$) δ 7.72 (1H, dd, *J* = 7.8, 1.3 Hz, Ar**H**), 7.64 (1H, dd, *J* = 7.7, 1.4 Hz, Ar**H**), 7.39-7.30 (4H, m, Ar**H**), 7.26-7.20 (2H, m, Ar**H**), 7.17 (1H, s, C=C**H**), 3.17 (1H, dd, *J* = 15.0, 2.0 Hz, C**H**$_2$C=C), 2.73 (1H, dd, *J* = 15.0, 0.8 Hz, C**H**$_2$C=C), 2.67 (1H, ddd, *J* = 15.0, 12.7, 6.6 Hz, C**H**$_2$C=O), 2.46-2.40 (1H, m, C**H**$_2$C=O), 2.32-2.14 (2H, m, C**H**$_2$COH), 2.02-1.96 (1H, m, $CH_2$C**H**$_2$$CH_2$), 1.94 (1H, d, *J* = 2.0 Hz, O**H**), 1.92-1.84 (1H, m, $CH_2$C**H**$_2$$CH_2$), 1.02 (3H, s, C**H**$_3$);$^{13}$C NMR (126 MHz, $CDCl_3$) δ 213.4 (C), 142.1 (C), 142.1 (C), 135.7 (C), 133.7 (C), 132.9 (C), 130.5 (2 × CH), 128.7 (CH), 128.6 (2 × CH), 127.9 (CH), 125.7 (CH), 125.4 (CH), 123.8 (CH), 78.3 (C), 53.4 (C), 36.9 ($CH_2$), 36.7 ($CH_2$), 36.6 ($CH_2$), 20.8 ($CH_2$), 16.1 ($CH_3$); HRMS (ES) Exact mass calcd for $C_{22}H_{21}{}^{35}ClNaO_2$ $[M+Na]^+$: 375.1128, found: 375.1113.

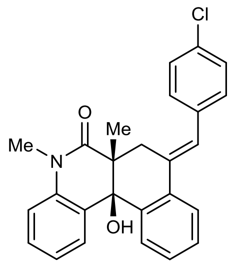

**(±)-(6a*R*,12b*S*)-8-[(*E*)-4-Chlorobenzylidene)-12b-hydroxy-5,6a-dimethyl-6a,7,8,12b-tetrahydrobenzo[*k*]phenanthridin-6(5*H*)-one (2n).** According to the general procedure, reaction of alkynone **1n** (135 mg, 0.400 mmol) and phenylboronic acid (73.2 mg, 0.600 mmol) gave, after purification by column chromatography (10% EtOAc/hexane), *alcohol* **2n** (120 mg, 72%) as a white solid. $R_f$ = 0.38 (30% EtOAc/petroleum ether); m.p. 174-176 °C (MeOH/hexane); IR 3293 (OH), 2936, 1638 (C=O), 1377, 1018, 752 cm$^{-1}$; $^1$H NMR (400 MHz, $D_6$-DMSO, 85 °C) δ 7.88-7.84 (1H, m, Ar**H**), 7.57-7.47 (3H, br m, Ar**H**), 7.46-7.42 (2H, m, Ar**H**), 7.36 (1H, ddd, *J* = 8.1, 7.5, 1.6 Hz, Ar**H**), 7.32-7.26 (1H, m, Ar**H**), 7.20-7.13 (3H, m, Ar**H** and C=C**H**), 7.08 (1H, dd, *J* = 8.1, 1.0 Hz, Ar**H**), 7.00 (1H, br s, Ar**H**), 5.71 (1H, s, O**H**), 3.22 (1H, br d, *J* = 16.1 Hz, C**H**$_2$), 3.09 (3H, s, NC**H**$_3$), 2.82 (1H, dd, *J* = 16.1, 2.4 Hz, C**H**$_2$), 1.00 (3H, s, CC**H**$_3$); $^{13}$C NMR (101 MHz, $D_6$-DMSO, 85 °C) δ 171.7 (C), 138.3 (C), 137.5 (C), 136.2 (C), 134.5 (C), 133.6 (C), 130.8 (C), 130.5 (2 × CH), 130.4 (C), 128.0 (CH), 127.8 (2 × CH), 127.5 (CH), 126.9 (CH), 126.8 (CH), 126.5 (CH), 123.3 (CH), 123.1 (CH), 121.9 (CH), 113.9 (CH), 72.2 (C), 45.8 (C), 32.3 ($CH_2$), 28.9 ($CH_3$), 18.0 ($CH_3$); HRMS (ESI) Exact mass calcd for $C_{26}H_{23}{}^{35}ClNO_3$ [M+H]$^+$: 416.1417, found: 416.1413.

**(±)-Ethyl (3a*S*,9b*R*)-5-[(*E*)-benzylidene]-9b-hydroxy-1,2,3,4,5,9b-hexahydro-3a*H*-cyclopenta[*a*]naphthalene-3a-carboxylate (9)**

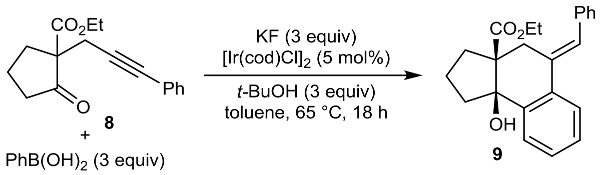


Alkynone **8** (108 mg, 0.400 mmol), phenylboronic acid (146 mg, 1.21 mmol), $[Ir(cod)Cl]_2$ (6.7 mg, 0.010 mmol), and KF (69.7 mg, 1.20 mmol) were added to an oven-dried microwave vial. The vial was sealed with a septum-lined cap and purged with nitrogen for 1 h. Anhydrous toluene (4.0 mL) and *t*-BuOH (126 µL, 1.32 mmol) were added and the mixture was stirred at 65 °C for 4 h. A solution of $[Ir(cod)Cl]_2$ (6.7 mg, 0.010 mmol) in anhydrous toluene (1.0 mL) was added, and the mixture was stirred at 65 °C for 14 h. The reaction was cooled to room temperature, water (5 mL) and saturated aqueous $NH_4Cl$ (5 mL) were added, and the mixture was extracted with EtOAc (3 × 10 mL). The combined organic phases were washed with brine (20 mL), dried ($MgSO_4$), filtered, and concentrated *in vacuo*. The mixture was purified by column chromatography (10% hexane/toluene) to give *alcohol* **9** (100 mg, 72%) as a white solid. $R_f$ = 0.30 (10% EtOAc/hexane); m.p. 100-105 °C ($CH_2Cl_2$/hexane); IR 3482 (OH), 2969, 1693 (C=O), 1313, 1201, 1020 cm$^{-1}$; $^1$H NMR (500 MHz, $CDCl_3$) δ 7.72 (1H, dd, *J* = 7.7, 1.2 Hz, Ar**H**), 7.56 (1H, dd, *J* = 7.9, 1.3 Hz, Ar**H**), 7.40-7.23 (6H, m, Ar**H**), 7.07 (1H, d, *J* = 1.7 Hz, C=C**H**), 4.38 (1H, d, *J* = 2.2 Hz, O**H**), 3.93

(1H, dq, $J$ = 10.7, 7.1 Hz, C**H**$_2$CH$_3$), 3.79 (1H, dq, $J$ = 10.7, 7.1 Hz, C**H**$_2$CH$_3$), 3.50 (1H, d, $J$ = 14.2 Hz, C**H**$_2$C=C), 2.71 (1H, dd, $J$ = 14.2, 1.7 Hz, C**H**$_2$C=C), 2.44-2.36 (1H, m, C**H**$_2$COH), 2.20-2.01 (3H, m, C**H**$_2$C**H**$_2$CH$_2$COH), 1.93-1.81 (2H, m, C**H**$_2$CH$_2$CH$_2$COH and C**H**$_2$COH), 0.85 (3H, t, $J$ = 7.1 Hz, C**H**$_3$); $^{13}$C NMR (126 MHz, CDCl$_3$) δ 176.2 (C), 141.8 (C), 137.5 (C), 134.3 (C), 134.1 (C), 129.1 (2 × CH), 128.4 (CH), 128.3 (2 × CH), 127.2 (CH), 127.0 (CH), 126.8 (CH), 126.2 (CH), 123.7 (CH), 81.9 (C), 60.7 (CH$_2$), 57.7 (C), 41.2 (CH$_2$), 36.1 (CH$_2$), 34.5 (CH$_2$), 20.7 (CH$_2$), 13.5 (CH$_3$); HRMS (ESI) Exact mass calcd for $C_{23}H_{24}NaO_3$ [M+Na]$^+$: 371.1618, found: 371.1622.

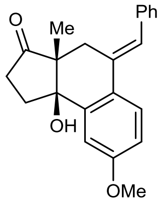


**(±)-(3a*R*,9b*R*)-5-[(*E*)-Benzylidene]-9b-hydroxy-8-methoxy-3a-methyl-1,2,3a,4,5,9b-hexahydro-3*H*-cyclopenta[*a*]naphthalen-3-one (10a).** According to the general procedure, reaction of alkynone **1a** (90.5 mg, 0.400 mmol) and 4-methoxyphenylboronic acid (91.2 mg, 0.600 mmol) gave, after purification by column chromatography (40% EtOAc/hexane), *alcohol* **10a** (91.9 mg, 69%) as a white solid. $R_f$ = 0.35 (40% EtOAc/hexane); m.p. 189-190 °C (MeOH); IR 3478 (OH), 2961, 1736 (C=O), 1491, 1207, 866, 700 cm$^{-1}$; $^1$H NMR (500 MHz, CDCl$_3$) δ 7.64 (1H, d, $J$ = 8.8 Hz, Ar**H**), 7.40-7.34 (2H, m, Ar**H**), 7.28-7.24 (4H, m, Ar**H**), 7.13 (1H, d, $J$ = 1.5 Hz, C=C**H**), 6.92 (1H, dd, $J$ = 8.8, 2.8 Hz, Ar**H**), 3.90 (3H, s, OC**H**$_3$), 2.79 (1H, d, $J$ = 14.2 Hz, C**H**$_2$C=C), 2.68-2.59 (2H, m, C**H**$_2$C=O and C**H**$_2$C=C), 2.51 (1H, ddd, $J$ = 19.0, 9.0, 2.4 Hz, C**H**$_2$C=O), 2.42 (1H, ddd, $J$ = 13.6, 9.2, 2.4 Hz, C**H**$_2$COH), 2.35 (1H, dddd, $J$ = 13.5, 10.9, 9.1, 2.2 Hz, C**H**$_2$COH), 1.84 (1H, d, $J$ = 2.2 Hz, O**H**), 1.02 (3H, s, CC**H**$_3$); $^{13}$C NMR (126 MHz, CDCl$_3$) δ 219.8 (C), 160.2 (C), 141.5 (C), 137.3 (C), 131.6 (C), 129.1 (2 × CH), 128.4 (2 × CH), 126.9 (C), 126.8 (CH), 126.0 (CH), 125.7 (CH), 114.8 (CH), 110.6 (CH), 80.3 (C), 55.4 (CH$_3$), 54.2 (C), 35.8 (CH$_2$), 35.1 (CH$_2$), 34.4 (CH$_2$), 13.7 (CH$_3$); HRMS (ESI) Exact mass calcd for $C_{22}H_{22}NaO_3$ [M+Na]$^+$: 357.1467, found: 357.1455.

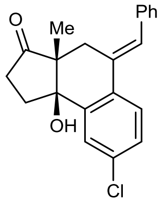


**(±)-(3a*R*,9b*R*)-5-[(*E*)-Benzylidene]-8-chloro-9b-hydroxy-3a-methyl-1,2,3a,4,5,9b-hexahydro-3*H*-cyclopenta[*a*]naphthalen-3-one (10b).** In a modification of the general procedure, reaction of alkynone **1a** (90.5 mg, 0.400 mmol), 4-chlorophenylboronic acid (93.8 mg, 0.600 mmol), and [Ir(cod)Cl]$_2$ (6.7 mg, 0.01 mmol) gave, after purification by column chromatography (30% EtOAc/hexane), *alcohol* **10b** (83.6 mg, 62%) as a pale yellow solid. $R_f$ = 0.54 (40% EtOAc/hexane); m.p. 178-179 °C (Et$_2$O/hexane); IR 3468 (OH), 2962, 1724 (C=O), 1192, 1076, 947, 810, 694 cm$^{-1}$; $^1$H NMR (400 MHz, CDCl$_3$) δ 7.71 (1H, d, $J$ = 2.3 Hz, Ar**H**), 7.61 (1H, d, $J$ = 8.6 Hz, Ar**H**), 7.40-7.34 (2H, m, Ar**H**), 7.33-7.23 (4H, m, Ar**H**), 7.20 (1H, d, $J$ = 1.4 Hz, C=C**H**), 2.78 (1H, d, $J$ = 14.3 Hz, C**H**$_2$C=C), 2.69-2.56 (2H, m, C**H**$_2$C=C and C**H**$_2$C=O), 2.55-2.25 (3H, m, C**H**$_2$C=O and C**H**$_2$COH),

1.82 (1H, d, $J$ = 2.2 Hz, O**H**), 0.99 (3H, s, C**H**$_3$); $^{13}$C NMR (126 MHz, $CDCl_3$) δ 219.2 (C), 141.8 (C), 136.8 (C), 134.5 (C), 132.7 (C), 131.1 (C), 129.1 (2 × CH), 128.5 (2 × CH), 128.40 (CH), 128.39 (CH), 127.3 (CH), 126.7 (CH), 125.7 (CH), 79.9 (C), 53.9 (C), 35.8 ($CH_2$), 35.0 ($CH_2$), 34.2 ($CH_2$), 13.7 ($CH_3$); HRMS (ESI) $C_{21}H_{19}{}^{35}ClNaO_3$ [M+Na]$^+$: 361.0966, found: 361.0959.

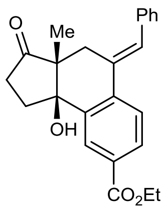


**(±)-Ethyl (3a*R*,9b*R*)-5-[(*E*)-benzylidene]-9b-hydroxy-3a-methyl-3-oxo-2,3,3a,4,5,9b-hexahydro-1*H*-cyclopenta[*a*]naphthalene-8-carboxylate (10c).** In a modification of the general procedure, reaction of alkynone **1a** (90.5 mg, 0.400 mmol), 4-(ethoxycarbonyl)phenylboronic acid (232 mg, 1.20 mmol), $[Ir(cod)Cl]_2$ (6.7 mg, 0.010 mmol), KF (69.7 mg, 1.20 mmol), and *t*-BuOH (0.11 mL, 1.2 mmol) at 90 °C for 16 h gave, after purification by column chromatography (10% $Et_2O/CH_2Cl_2$), *alcohol* **10c** (52.8 mg, 35%) as a white solid. $R_f$ = 0.30 (10% $Et_2O/CH_2Cl_2$); m.p. 174-175 °C (EtOH); IR 3449 (OH), 2976, 1738 (C=O), 1678 (C=O), 1292, 1256, 1030, 770, 698 cm$^{-1}$; $^1$H NMR (500 MHz, $CDCl_3$) δ 8.40 (1H, d, $J$ = 1.7 Hz, Ar**H**), 7.99 (1H, dd, $J$ = 8.3, 1.9 Hz, Ar**H**), 7.74 (1H, d, $J$ = 8.3 Hz, Ar**H**), 7.41-7.36 (2H, m, Ar**H**), 7.34-7.27 (4H, m, Ar**H** and C=C**H**), 4.42 (2H, qd, $J$ = 7.1, 0.8 Hz, OC**H**$_2$), 2.81 (1H, d, $J$ = 14.3 Hz, C**H**$_2$C=C), 2.70-2.60 (2H, m, C**H**$_2$C=C and C**H**$_2$C=O), 2.54-2.43 (2H, m, C**H**$_2$C=O and C**H**$_2$COH), 2.38-2.28 (1H, m, C**H**$_2$COH), 1.90 (1H, d, $J$ = 2.1 Hz, O**H**), 1.44 (1H, t, $J$ = 7.1 Hz, $CH_2$C**H**$_3$), 1.01 (3H, s, O=CCC**H**$_3$); $^{13}$C NMR (126 MHz, $CDCl_3$) δ 219.3 (C), 166.2 (C), 140.2 (C), 138.6 (C), 136.6 (C), 131.5 (C), 130.6 (C), 130.2 (CH), 129.1 (2 × CH), 129.0 (CH), 128.5 (2 × CH), 128.1 (CH), 127.5 (CH), 124.3 (CH), 79.9 (C), 61.2 ($CH_2$), 54.0 (C), 36.0 ($CH_2$), 35.0 ($CH_2$), 34.2 ($CH_2$), 14.4 ($CH_3$), 13.8 ($CH_3$); HRMS (ESI) Exact mass calcd for $C_{24}H_{24}NaO_4$ [M+Na]$^+$: 399.1572, found: 399.1556.

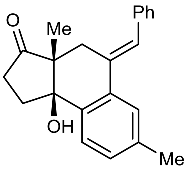


**(±)-(3a*R*,9b*R*)-5-[(*E*)-Benzylidene]-9b-hydroxy-3a,7-dimethyl-1,2,3a,4,5,9b-hexahydro-3*H*-cyclopenta[*a*]naphthalen-3-one (10d).** According to the general procedure, reaction of alkynone **1a** (90.5 mg, 0.400 mmol) and 3-methylphenylboronic acid (81.6 mg, 0.600 mmol) gave, after purification by column chromatography (40% EtOAc/hexane), *alcohol* **10d** (87.0 mg, 68%) as a white solid. $R_f$ = 0.44 (40% EtOAc/hexane); m.p. 154-155 °C (MeOH); IR 3445 (OH), 2928, 1715 (C=O), 1443, 1206, 1067, 835, 702 cm$^{-1}$; $^1$H NMR (400 MHz, $CDCl_3$) δ 7.61 (1H, d, $J$ = 7.9 Hz, Ar**H**), 7.49 (1H, s, Ar**H**), 7.40-7.34 (2H, m, Ar**H**), 7.30-7.19 (5H, m, Ar**H** and C=C**H**), 2.77 (1H, d, $J$ = 14.1 Hz, C**H**$_2$C=C), 2.67-2.55 (2H, m, C**H**$_2$C=C and C**H**$_2$C=O), 2.52-2.25 (3H, m, C**H**$_2$C=O and C**H**$_2$COH), 2.41 (3H, s, ArC**H**$_3$), 1.77 (1H, d, $J$ = 2.1 Hz, O**H**), 1.00 (3H, s, O=CCC**H**$_3$); $^{13}$C NMR (126 MHz, $CDCl_3$) δ 220.0 (C), 137.8 (C), 137.4 (C), 137.2 (C), 134.1 (C), 132.2 (C), 129.8 (CH), 129.1 (2 ×

CH), 128.4 (2 × CH), 127.7 (CH), 127.0 (CH), 126.5 (CH), 124.5 (CH), 80.0 (C), 54.2 (C), 35.9 ($CH_2$), 35.2 ($CH_2$), 34.3 ($CH_2$), 21.3 ($CH_3$), 13.9 ($CH_3$); HRMS (ESI) Exact mass calcd for $C_{22}H_{22}NaO_2$ $[M+Na]^+$: 341.1517, found: 341.1507.

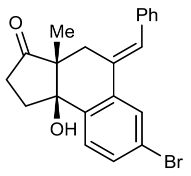


**(±)-(3a*R*,9b*R*)-5-[(*E*)-Benzylidene]-7-bromo-9b-hydroxy-3a-methyl-1,2,3a,4,5,9b-hexahydro-3*H*-cyclopenta[*a*]naphthalen-3-one (10e).** According to the general procedure, reaction of alkynone **1a** (90.5 mg, 0.400 mmol) and 3-bromophenylboronic acid (93.8 mg, 0.600 mmol) gave, after purification by column chromatography (40% EtOAc/hexane), *alcohol* **10e** (89.0 mg, 58%) as a pale yellow solid. $R_f$ = 0.37 (40% EtOAc/hexane); m.p. 159-160 °C (MeOH); IR 3439 (OH), 2968, 1717 (C=O), 1400, 1231, 1078, 962, 839, 696 $cm^{-1}$; $^1H$ NMR (400 MHz, $CDCl_3$) δ 7.81 (1H, d, *J* = 2.0 Hz, Ar**H**), 7.60 (1H, d, *J* = 8.4 Hz, Ar**H**), 7.50 (1H, dd, *J* = 8.4, 2.0 Hz, Ar**H**), 7.41-7.34 (2H, m, Ar**H**), 7.33-7.24 (3H, m, Ar**H**), 7.21 (1H, d, *J* = 1.3 Hz, C=C**H**), 2.77 (1H, d, *J* = 14.2 Hz, C**H**$_2$C=C), 2.68-2.56 (2H, m, C**H**$_2$C=C and C**H**$_2$C=O), 2.53-2.23 (3H, m, C**H**$_2$C=O and C**H**$_2$COH), 1.79 (1H, d, *J* = 2.2 Hz, O**H**), 0.99 (3H, s, CC**H**$_3$); $^{13}C$ NMR (126 MHz, $CDCl_3$) δ 219.3 (C), 139.0 (C), 136.6 (C), 136.4 (C), 131.6 (CH), 130.9 (C), 129.2 (CH), 129.1 (2 × CH), 128.5 (CH), 128.5 (2 × CH), 127.4 (CH), 127.0 (CH), 122.4 (C), 79.9 (C), 53.9 (C), 35.8 ($CH_2$), 35.0 ($CH_2$), 34.2 ($CH_2$), 13.8 ($CH_3$); HRMS (ESI) Exact mass calcd for $C_{21}H_{18}{}^{79}BrO$ $[M-H_2O]^+$: 365.0541, found: 365.0531.

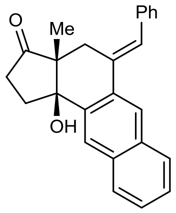


**(±)-(3a*R*,11b*R*)-5-[(*E*)-Benzylidene]-11b-hydroxy-3a-methyl-1,2,3a,4,5,11b-hexahydro-3*H*-cyclopenta[*a*]anthracen-3-one (10f).** In a modification of the general procedure, reaction of alkynone **1a** (90.5 mg, 0.400 mmol), 2-naphthylboronic acid (103 mg, 0.600 mmol), and $[Ir(cod)Cl]_2$ (6.7 mg, 0.010 mmol) in toluene (8 mL) at 90 °C for 16 h gave, after purification by column chromatography (30% EtOAc/hexane), *alcohol* **10f** (83.8 mg, 59%) as a white solid. $R_f$ = 0.31 (30% EtOAc/hexane); m.p. 146-147 °C (EtOAc/hexane); IR 3426 (OH), 2961, 1726 (C=O), 1269, 1024, 899, 743, 698 $cm^{-1}$; $^1H$ NMR (500 MHz, $CDCl_3$) δ 8.21 (1H, s, Ar**H**), 8.15 (1H, s, Ar**H**), 7.91-7.84 (2H, m, Ar**H**), 7.53-7.46 (2H, m, Ar**H**), 7.42-7.36 (3H, m, Ar**H** and C=C**H**), 7.34-7.27 (3H, m, Ar**H**), 2.88 (1H, d, *J* = 14.2 Hz, C**H**$_2$C=C), 2.75-2.62 (2H, m, C**H**$_2$C=C and C**H**$_2$C=O), 2.59-2.34 (3H, m, C**H**$_2$C=O and C**H**$_2$COH), 1.97 (1H, d, *J* = 2.3 Hz, O**H**), 1.04 (3H, s, C**H**$_3$); $^{13}C$ NMR (126 MHz, $CDCl_3$) δ 219.8 (C), 138.9 (C), 137.1 (C), 133.5 (C), 133.1 (C), 133.0 (C), 132.3 (C), 129.2 (2 × CH), 128.6 (CH), 128.5 (2 × CH), 128.0 (CH), 127.6 (CH), 127.2 (CH), 126.5 (CH), 126.4 (CH), 125.5 (CH), 123.2 (CH), 80.5 (C), 54.4 (C), 36.5 ($CH_2$), 35.0 ($CH_2$), 34.6 ($CH_2$), 13.6 ($CH_3$); HRMS (ESI) Exact mass calcd for $C_{25}H_{22}NaO_2$ $[M+Na]^+$: 377.1517, found: 377.1507.

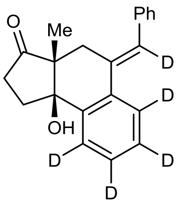


**(±)-(3a*R*,9b*R*,*E*)-9b-hydroxy-3a-methyl-5-(phenylmethylene-d)-1,2,3a,4,5,9b-hexahydro-3*H*-cyclopenta[*a*]naphthalen-3-one-6,7,8,9-d4 ([$D_5$]-2a).** According to the general procedure, reaction of alkynone **1a** (22.8 mg, 0.101 mmol), $d_5$-phenylboronic acid[6] (19.0 mg, 0.150 mmol), $[Ir(COD)Cl]_2$ (1.0 mg, 0.0015 mmol), KF (8.7 mg, 0.15 mmol), and *t*-BuOH (14.5 µL) in toluene (1 mL) gave, after purification by column chromatography (10% $Et_2O/CH_2Cl_2$), *alcohol* [$D_5$]-**2a** (17.6 mg, 56%) as a white solid. $R_f$ = 0.34 (10% $Et_2O/CH_2Cl_2$); $^1$H NMR (500 MHz, $CDCl_3$) δ 7.41-7.33 (2H, m, Ar**H**), 7.31-7.27 (3H, m, Ar**H**), 2.80 (1H, d, $J$ = 14.1 Hz, C**H**$_2$C=C), 2.69-2.58 (2H, m, C=CC**H**$_2$ and C**H**$_2$C=O), 2.53-2.28 (3H, m, C**H**$_2$C=O and C**H**$_2$COH), 1.85 (1H, d, $J$ = 2.1 Hz, O**H**), 1.01 (3H, s, C**H**$_3$); $^{13}$C NMR (126 MHz, $CDCl_3$) δ 219.9 (C), 140.0 (C), 137.0 (C), 134.2 (C), 132.0 (C), 129.1 (2 × CH), 128.5 (2 × CH), 128.3 (t, $J_D$ = 23.6 Hz, CD), 127.7 (t, $J_D$ = 24.5 Hz, CD), 127.6 (t, $J_D$ = 23.2 Hz, CD), 127.1 (CH), 126.1 (t, $J_D$ = 23.6 Hz, CD), 123.8 (t, $J_D$ = 23.8 Hz, CD), 80.1 (C), 54.1 (C), 35.9 ($CH_2$), 35.2 ($CH_2$), 34.3 ($CH_2$), 13.9 ($CH_3$). HRMS (ESI) Exact mass calcd for $C_{21}H_{16}D_5O_2$ $[M+H]^+$: 310.1850, found: 310.1842.

6. Prepared according to: C. Wang, S. Rakshit, F. Glorius, *J. Am. Chem. Soc.* **2010**, *132*, 14006-14008.

## 5. Enantioselective Iridium-Catalyzed Arylative Cyclizations

**(3a*R*,9b*R*)-9b-Hydroxy-5-[(*E*)-4-chlorobenzylidene]-3a-methyl-1,2,3a,4,5,9b-hexahydro-3*H*-cyclopenta[*a*]naphthalen-3-one (2c)**

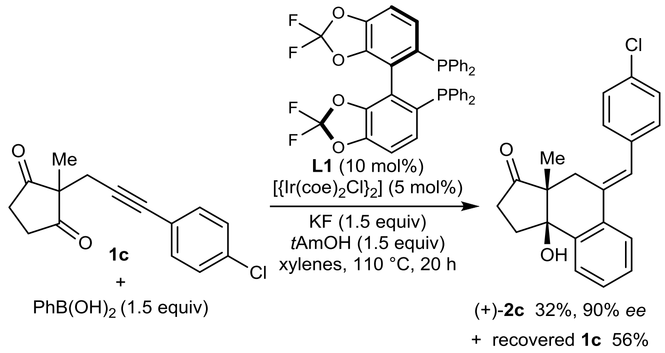


$[Ir(coe)_2Cl]_2$ (9.0 mg, 0.010 mmol) and (*R*)-Difluorphos (**L1**) (13.7 mg, 0.0201 mmol) were added to an oven-dried microwave vial, a septum was fitted, and the vial was purged with nitrogen for 1 h. Degassed xylenes (1 mL) were added and the mixture was stirred at room temperature for 30 min under nitrogen. In a separate microwave vial, alkynone **1c** (52.1 mg, 0.200 mmol), phenylboronic acid (36.3 mg, 0.300 mmol), and KF (34.8 mg, 0.300 mmol) were added. This vial was sealed with a septum-lined cap and purged with nitrogen for 1 h. The solution of catalyst was added to the vial containing the alkynone under nitrogen. *t*-Amyl alcohol (33 µL, 0.30 mmol) was added and the mixture was stirred at 110 °C for 20 h. The reaction was cooled to room temperature, water (5 mL) and saturated aqueous $NH_4Cl$ (5 mL) were added, and the mixture was extracted with EtOAc (3 × 10 mL). The combined organic phases were washed with brine (20 mL), dried ($MgSO_4$), filtered, and concentrated *in vacuo*. The mixture was purified by column chromatography (5% $Et_2O/CH_2Cl_2$) to give *alcohol* **2c** (21.6 mg, 32%) as a white solid. Alkynone **1c** (29.0 mg, 56%) was recovered as a brown solid. The NMR data of **2c** matched those of the racemic product above. $[\alpha]_D^{23.4}$ +170 (*c* 2.21, $CHCl_3$). Enantiomeric excess was determined by HPLC with a Chiralpak IA-3 column (80:20 hexane:*i*-PrOH, 1.0 mL/min, 230 nm, room temperature): $t_r$ (major) = 7.3 min, $t_r$ (minor) = 10.4 min; 90% ee.

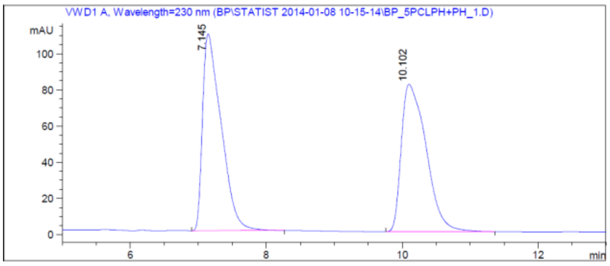


| Meas. R | Area % | Width | Symmetr. |
|---|---|---|---|
| 7.145 | 49.835 | 0.316 | 0.423 |
| 10.102 | 50.165 | 0.425 | 0.441 |

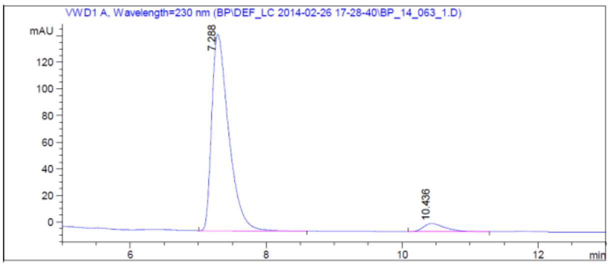


| Meas. R | Area % | Width | Symmetr. |
|---|---|---|---|
| 7.288 | 94.887 | 0.289 | 0.550 |
| 10.436 | 5.113 | 0.373 | 0.599 |

Slow diffusion petroleum ether of into a solution of **2c** in $Et_2O$ provided crystals that were suitable for X-ray diffraction, thus allowing confirmation of the absolute stereochemistry.

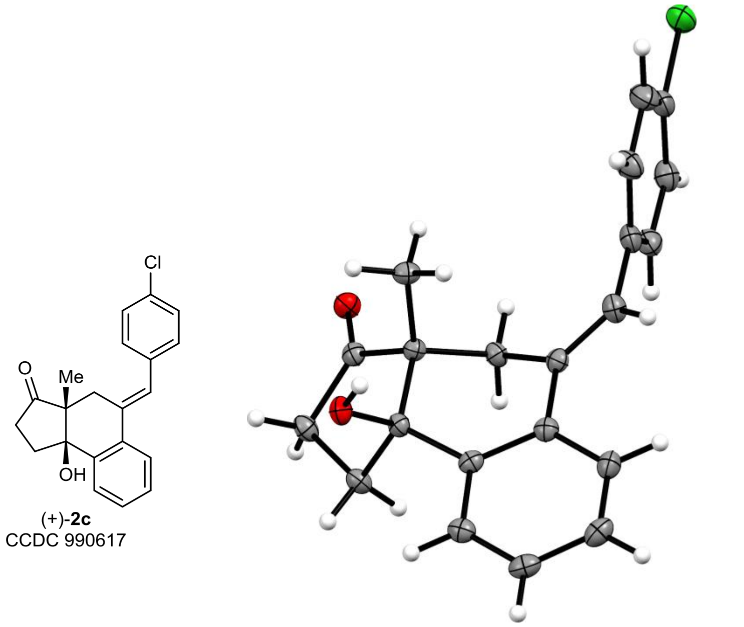


(+)-**2c**
CCDC 990617

**(6a*R*,11b*S*)-5-[(*E*)-Benzylidene]-11b-hydroxy-6a-methyl-5,6,6a,11b-tetrahydro-7*H*-benzo[*c*]fluoren-7-one (2i)**

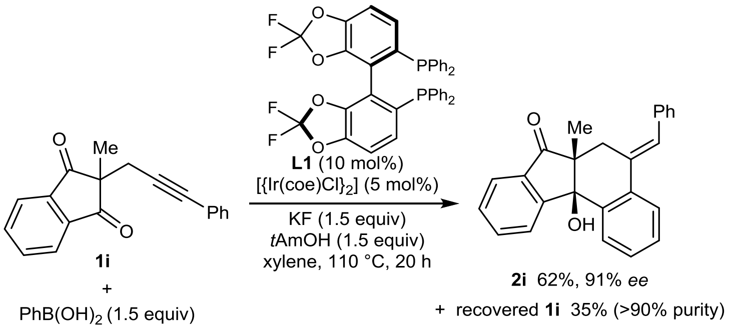


$[Ir(coe)_2Cl]_2$ (9.0 mg, 0.010 mmol) and (*R*)-Difluorphos (**L1**) (13.7 mg, 0.0201 mmol) were added to an oven-dried microwave vial, a septum was fitted, and the vial was purged with nitrogen for 1 h. Degassed xylenes (1 mL) were added and the mixture was stirred at room temperature for 30 min under nitrogen. In a separate microwave vial, alkynone **1i** (54.8 mg, 0.200 mmol), phenylboronic

acid (36.3 mg, 0.300 mmol), and KF (34.8 mg, 0.300 mmol) were added. This vial was sealed with a septum-lined cap and purged with nitrogen for 1 h. The solution of catalyst was added to the vial containing the alkynone under nitrogen. *t*-Amyl alcohol (33 µL, 0.30 mmol) was added and the mixture was stirred at 110 °C for 20 h. The reaction was cooled to room temperature, water (5 mL) and saturated aqueous $NH_4Cl$ (5 mL) were added, and the mixture was extracted with EtOAc (3 × 10 mL). The combined organic phases were washed with brine (20 mL), dried ($MgSO_4$), filtered, and concentrated *in vacuo*. The mixture was purified by column chromatography (5% $Et_2O/CH_2Cl_2$) to give *alcohol* **2o** (43.6 mg, 62%) as an orange solid. Alkynone **1i** (35.0 mg, 35%) was recovered as a brown solid in >90% purity by $^1$H NMR spectroscopy. The NMR data of **2i** matched those of the racemic product above. $[\alpha]_D^{24.2}$ −24.2 (*c* 0.83, MeOH). Enantiomeric excess was determined by HPLC with a Chiralpak IA-3 column (90:10 hexane:*i*-PrOH, 0.8 mL/min, 230 nm, room temperature); $t_r$ (major) = 13.6 min, $t_r$ (minor) = 15.8 min; 91% ee

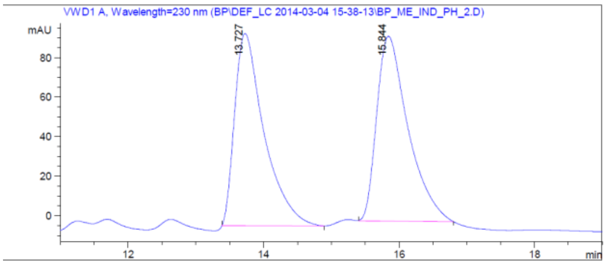


| Meas. R | Area % | Width | Symmetr. |
|---|---|---|---|
| 13.727 | 49.403 | 0.504 | 0.526 |
| 15.844 | 50.597 | 0.537 | 0.572 |

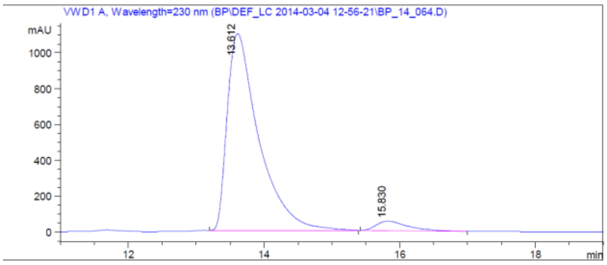


| Meas. R | Area % | Width | Symmetr. |
|---|---|---|---|
| 13.612 | 95.365 | 0.539 | 0.469 |
| 15.830 | 4.635 | 0.541 | 0.554 |

## 6. Semi-Quantitative ICP-MS Analysis

Samples were analyzed by ICP-MS using an Agilent 7500ce (with octopole reaction system), employing an rf forward power of 1540 W and reflected power of 1 W, with argon gas flows of 0.70 L $min^{-1}$ and 0.15 L $min^{-1}$ for carrier and makeup flows (1.5mm ID torch), respectively. Sample solutions were taken up into the Micro mist nebulizer by peripump at a rate of approximately 1.0 mL $min^{-1}$. Skimmer and sample cones were made of nickel.

The instrument was operated in spectrum acquisition mode. Each mass was analyzed in semi quant mode (three points per unit mass). A 1000 fold and 100 fold dilution of the Merck Multielement standard VI were used to calculate a response curve across the mass range from 6-238 amu. Separate cross check standards were analyzed to confirm accuracy of the response curve calibration.

**Sample preparation:** Ketone **X** (22.8 mg, 0.101 mmol), phenylboronic acid (18.3 mg, 0.150 mmol), $[Ir(cod)Cl]_2$ (1.0 mg, 0.0015 mmol), and KF (8.7 mg, 0.15 mmol), anhydrous toluene (1.0 mL) and *t*-BuOH (14.5 µL, 0.150 mmol) were added to an oven-dried microwave vial. The mixture was stirred and the volatiles were removed with a stream of nitrogen. The residue was dissolved in conc. $HNO_3$ (1.5 mL) and the solution was diluted to a volume of 10 mL using deionized water. The organic precipitate was filtered and the filtrate was submitted to ICP-MS analysis.

| Element | Concentration (ppb) |
|---|---|
| Fe | 130.0 |
| Co | 4.5 |
| Ni | 29.0 |
| Cu | 19.0 |
| Ru | 0.2 |
| Rh | 3.0 |
| Pd | 62.0 |
| Ag | 5.6 |
| Ir | 43000.0 |
| Pt | 0.1 |
| Au | 0.2 |

Table 1: ICP-MS analysis data (semi-quantitative).

# 7. NMR Spectra

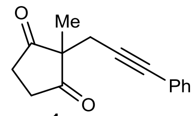


**1a**

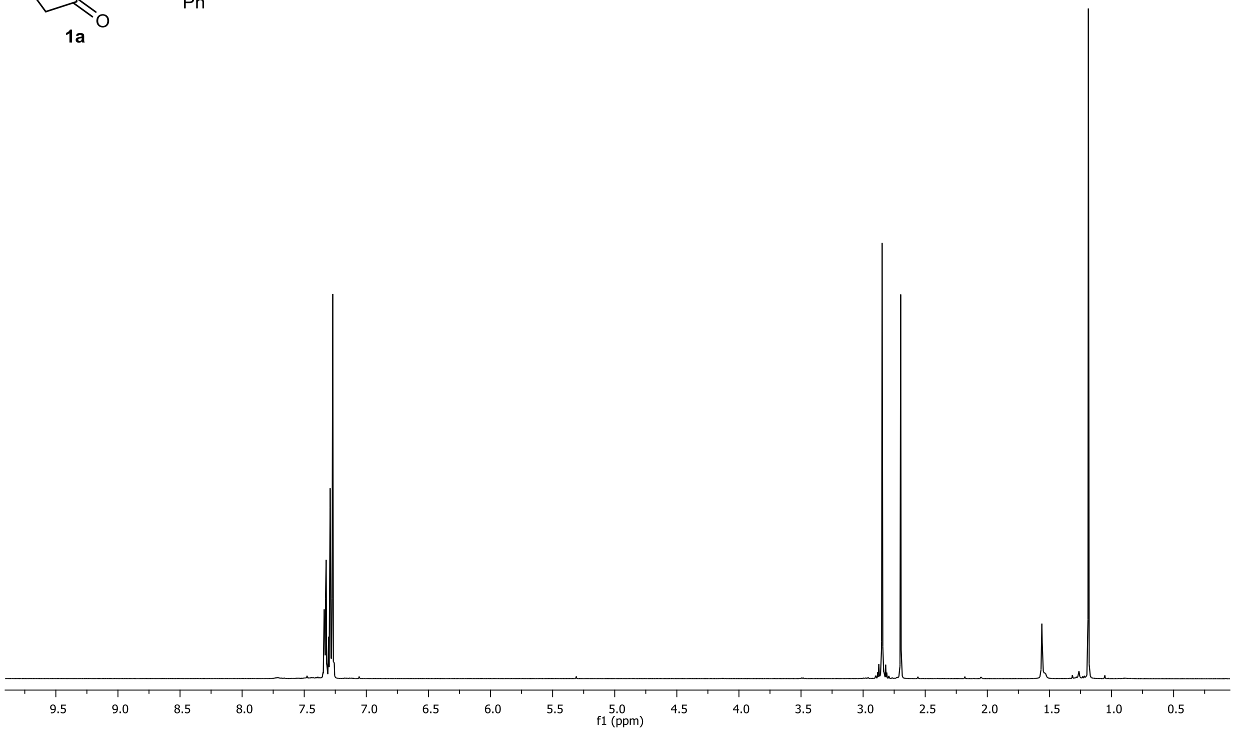


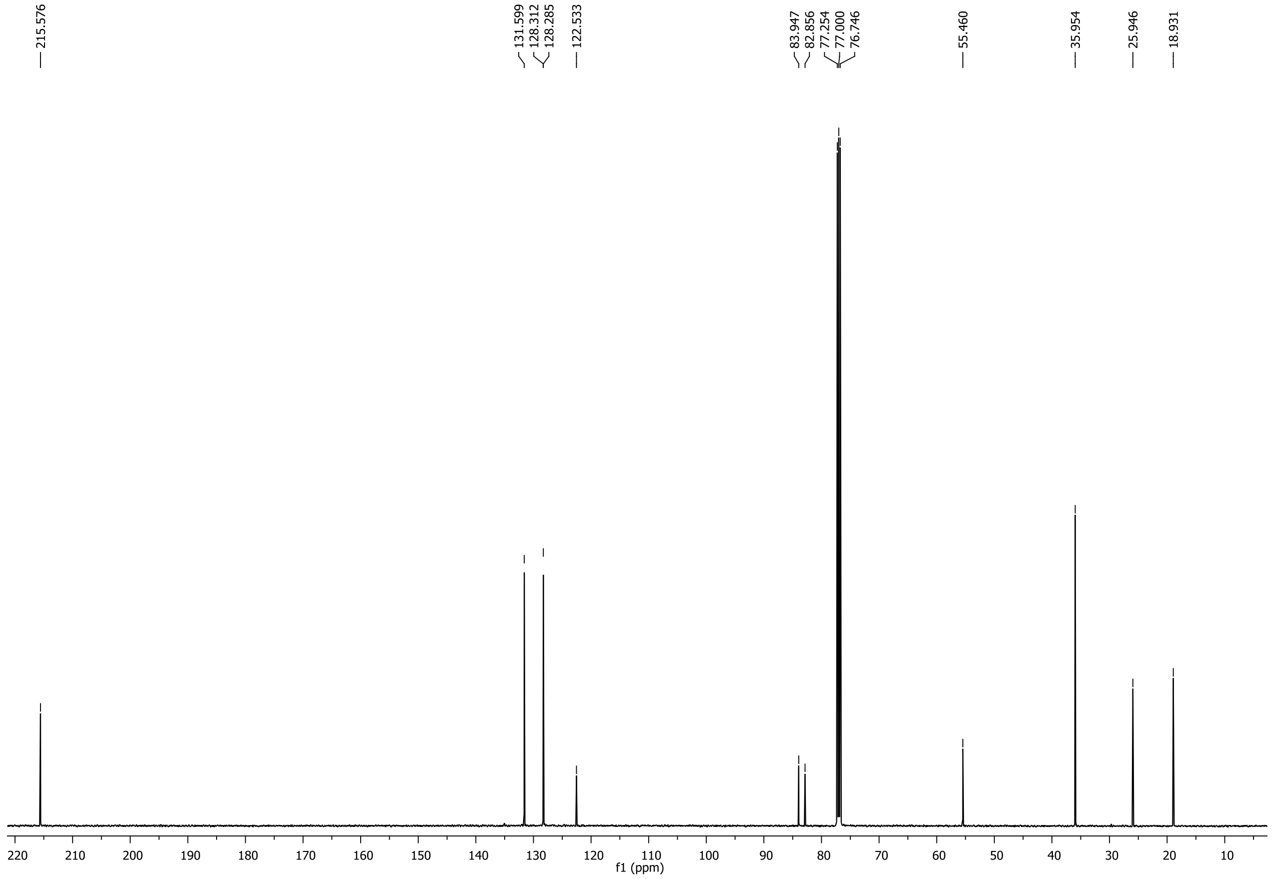

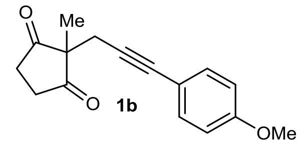
Me
O
O
1b
OMe

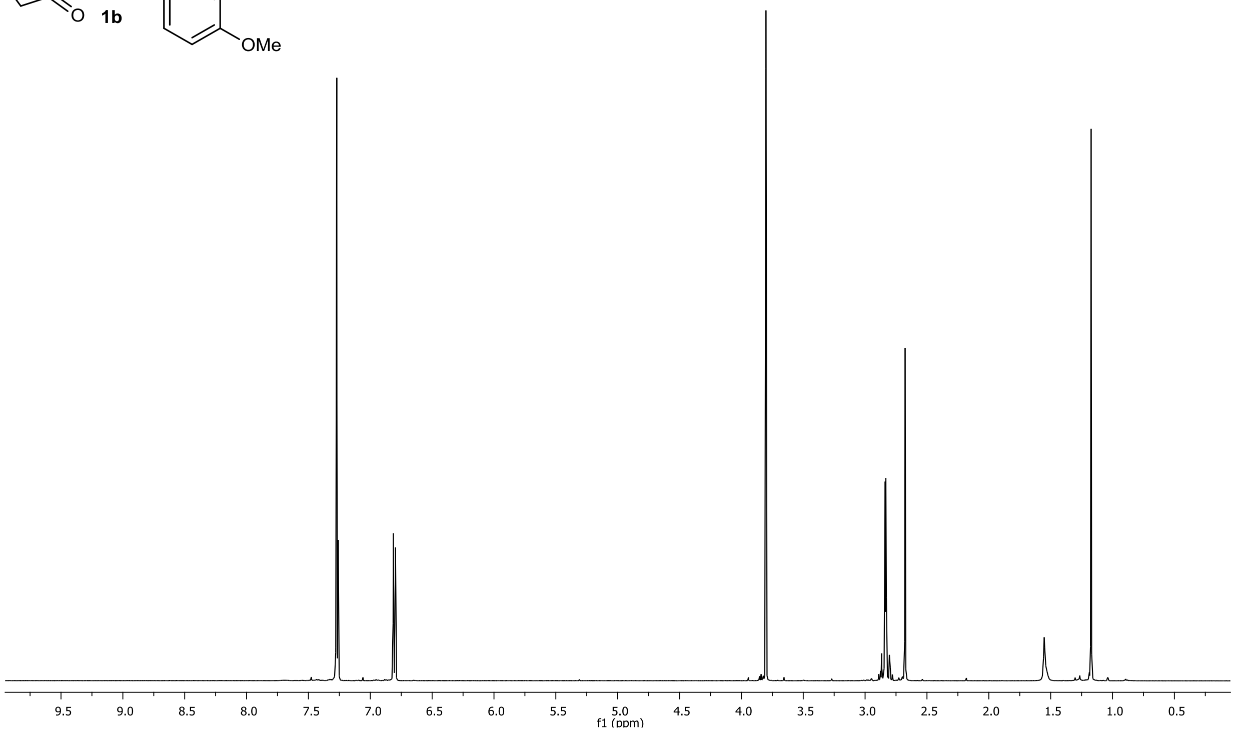
9.5
9.0
8.5
8.0
7.5
7.0
6.5
6.0
5.5
5.0
4.5
4.0
3.5
3.0
2.5
2.0
1.5
1.0
0.5
f1 (ppm)

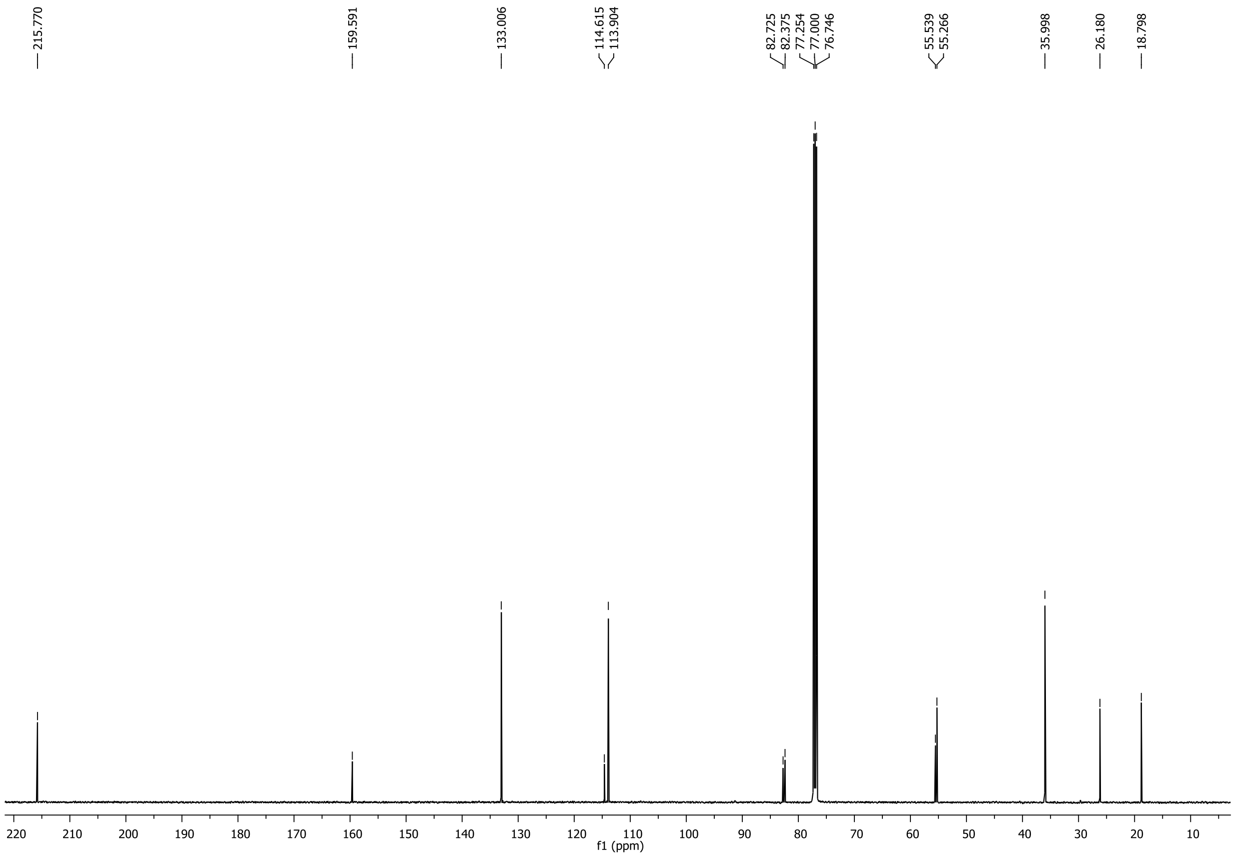
215.770
159.591
133.006
114.615
113.904
82.725
82.375
77.254
77.000
76.746
55.539
55.266
35.998
26.180
18.798
220
210
200
190
180
170
160
150
140
130
120
110
100
90
80
70
60
50
40
30
20
10
f1 (ppm)

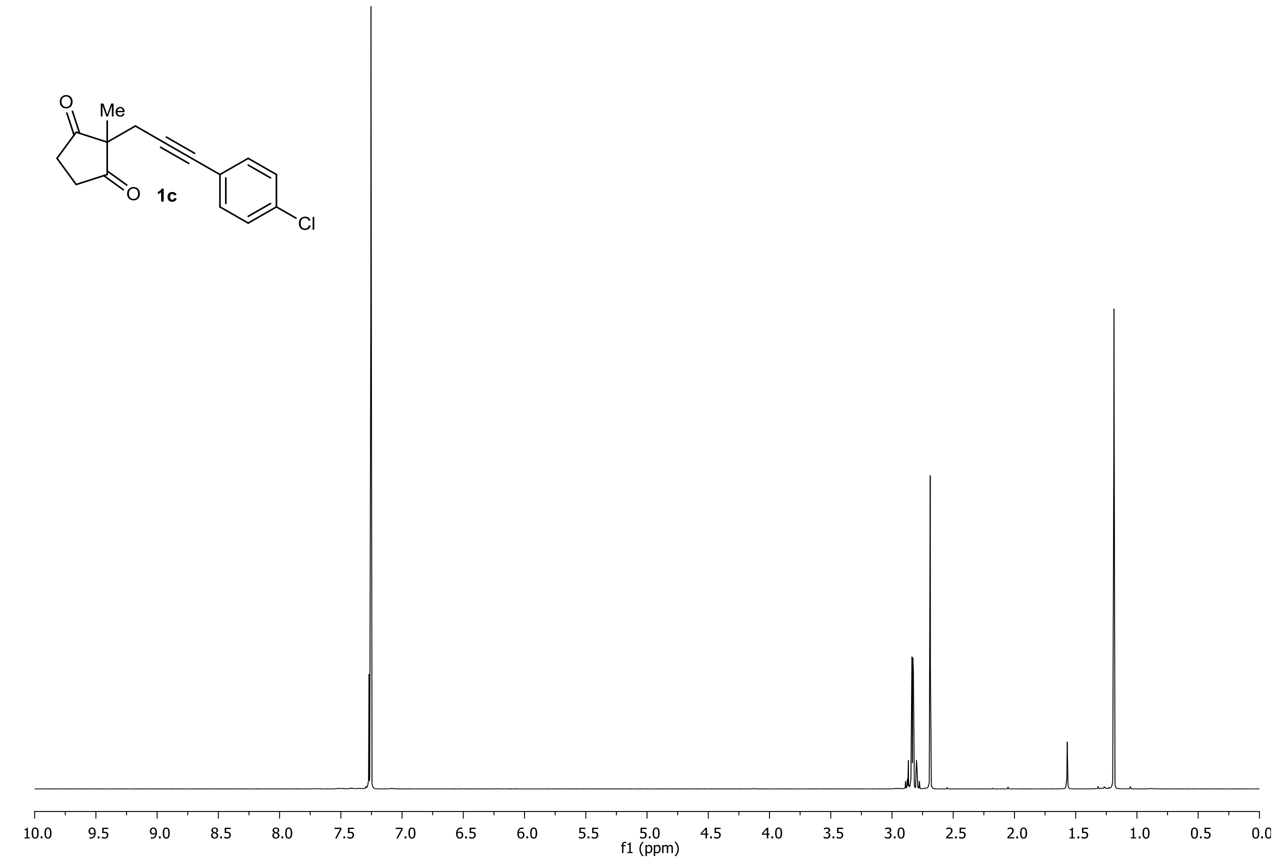
O
Me
O
1c
Cl
10.0 9.5 9.0 8.5 8.0 7.5 7.0 6.5 6.0 5.5 5.0 4.5 4.0 3.5 3.0 2.5 2.0 1.5 1.0 0.5 0.0
f1 (ppm)

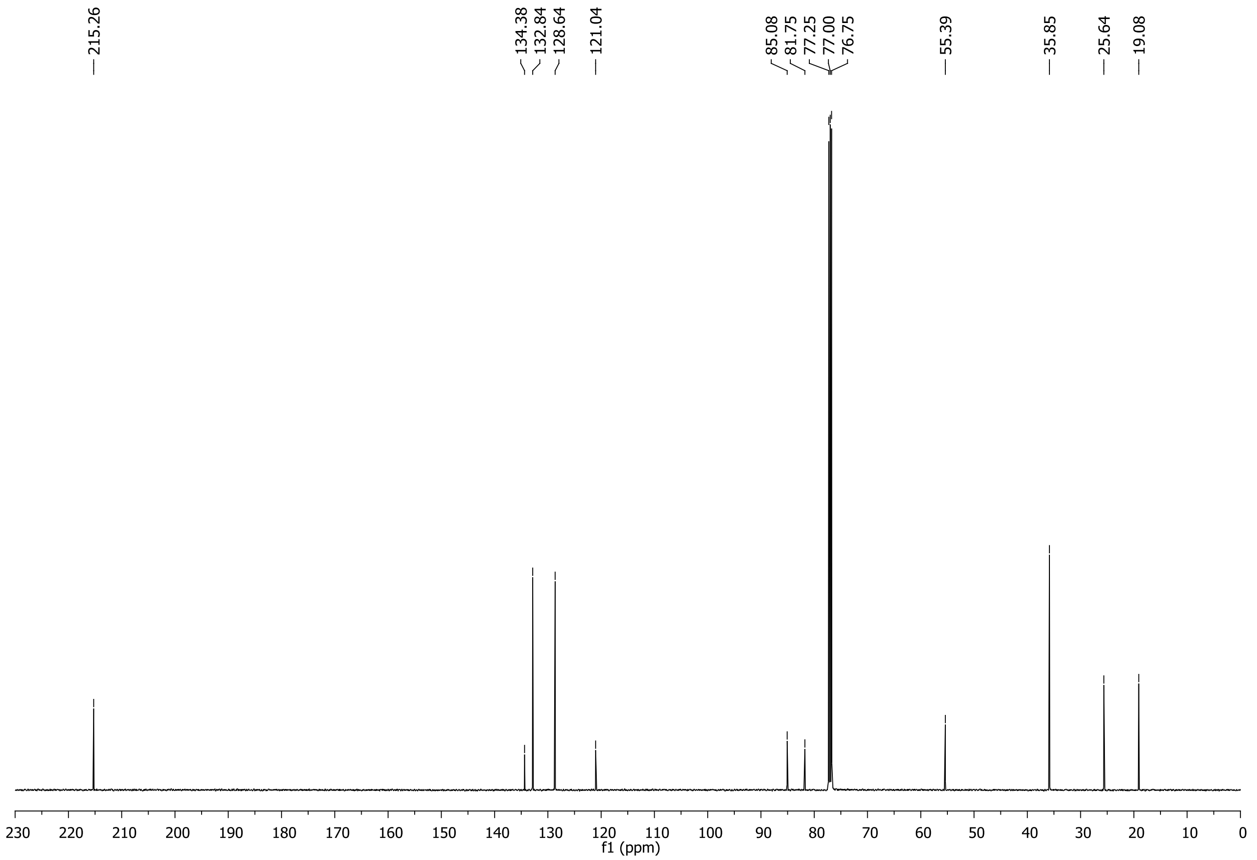
215.26
134.38
132.84
128.64
121.04
85.08
81.75
77.25
77.00
76.75
55.39
35.85
25.64
19.08
230 220 210 200 190 180 170 160 150 140 130 120 110 100 90 80 70 60 50 40 30 20 10 0
f1 (ppm)

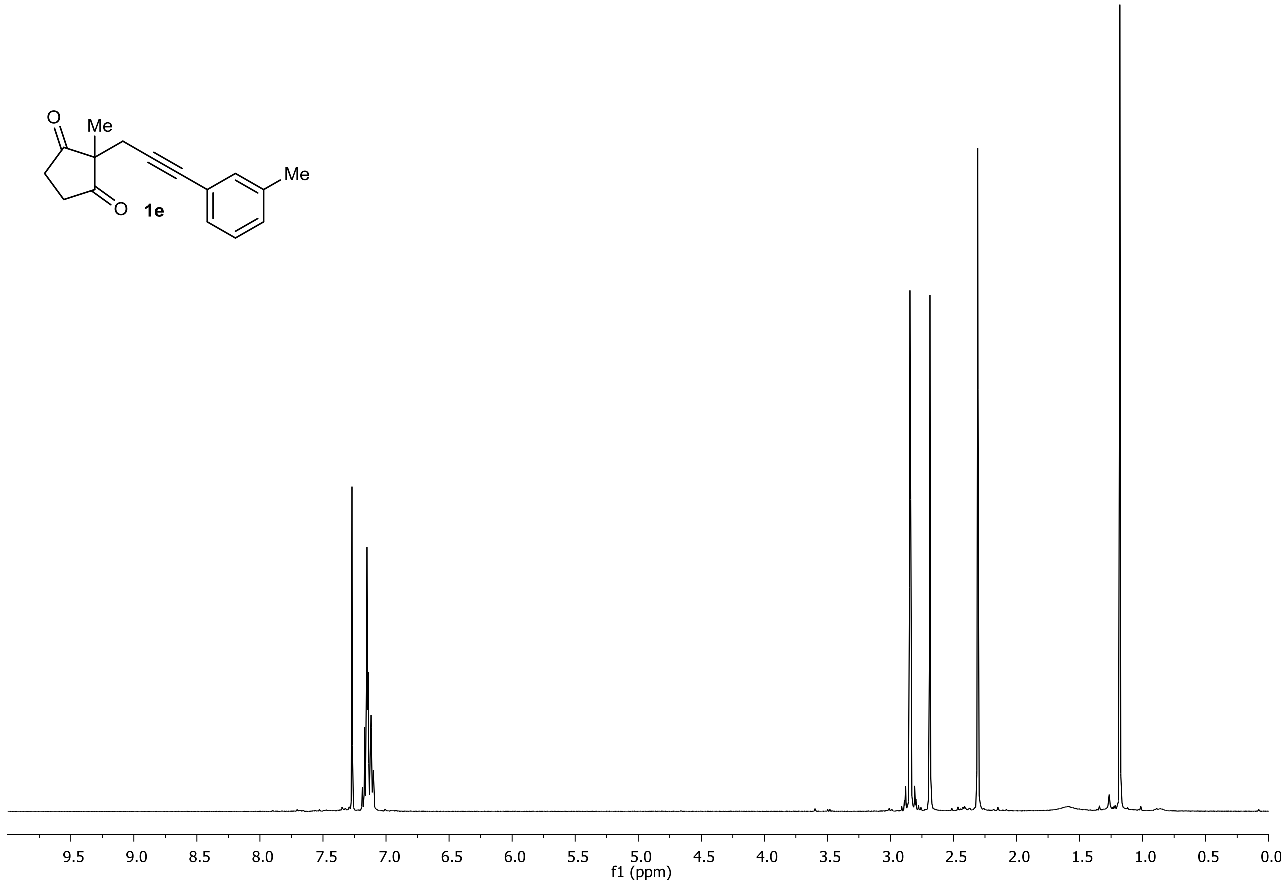
O
Me
Me
O 1e
9.5 9.0 8.5 8.0 7.5 7.0 6.5 6.0 5.5 5.0 4.5 4.0 3.5 3.0 2.5 2.0 1.5 1.0 0.5 0.0
f1 (ppm)

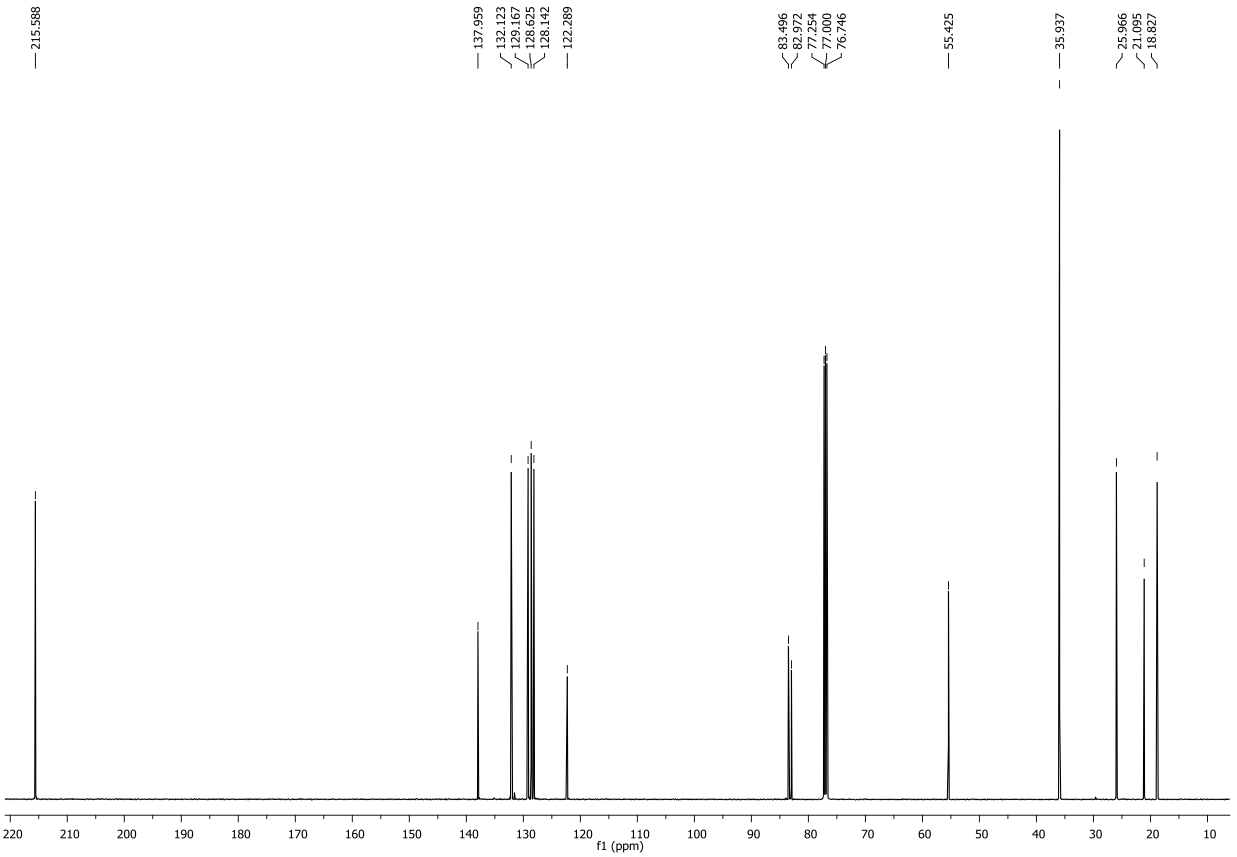
215.588
137.959
132.123
129.167
128.625
128.142
122.289
83.496
82.972
77.254
77.000
76.746
55.425
35.937
25.966
21.095
18.827
220 210 200 190 180 170 160 150 140 130 120 110 100 90 80 70 60 50 40 30 20 10
f1 (ppm)

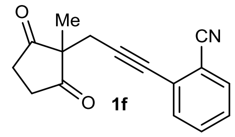
O
Me
CN
O

**1f**

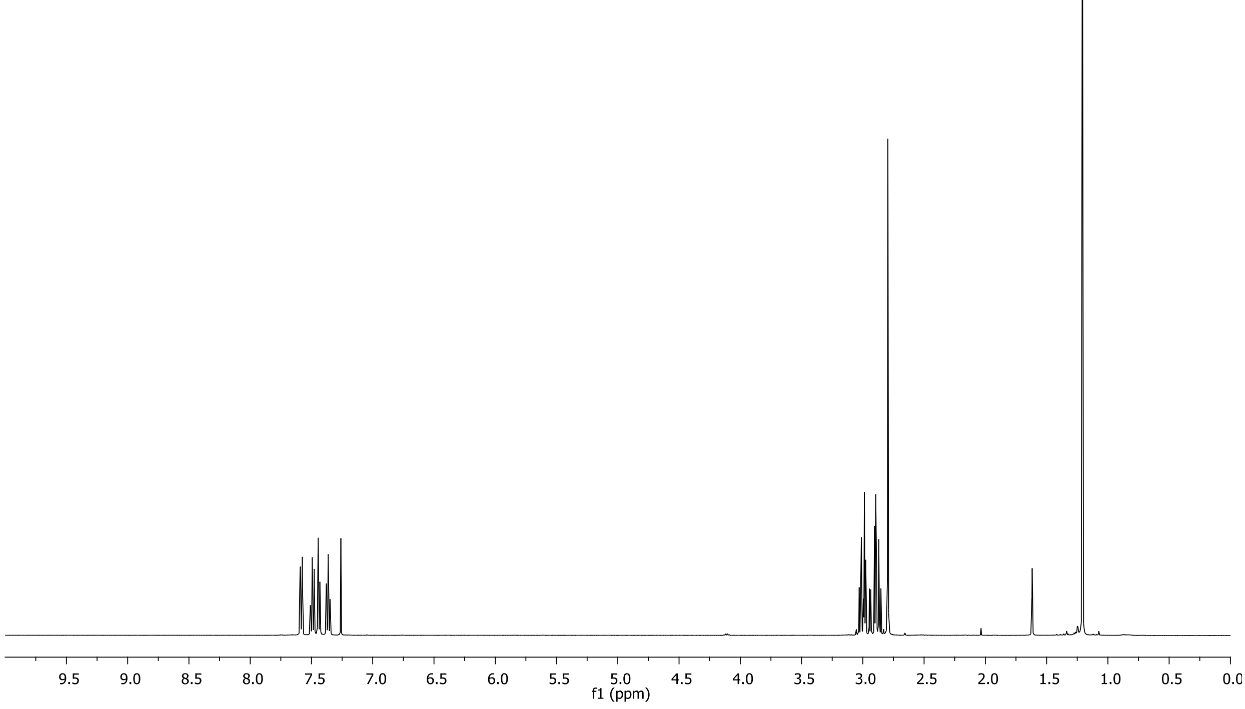
9.5 9.0 8.5 8.0 7.5 7.0 6.5 6.0 5.5 5.0 4.5 4.0 3.5 3.0 2.5 2.0 1.5 1.0 0.5 0.0
f1 (ppm)

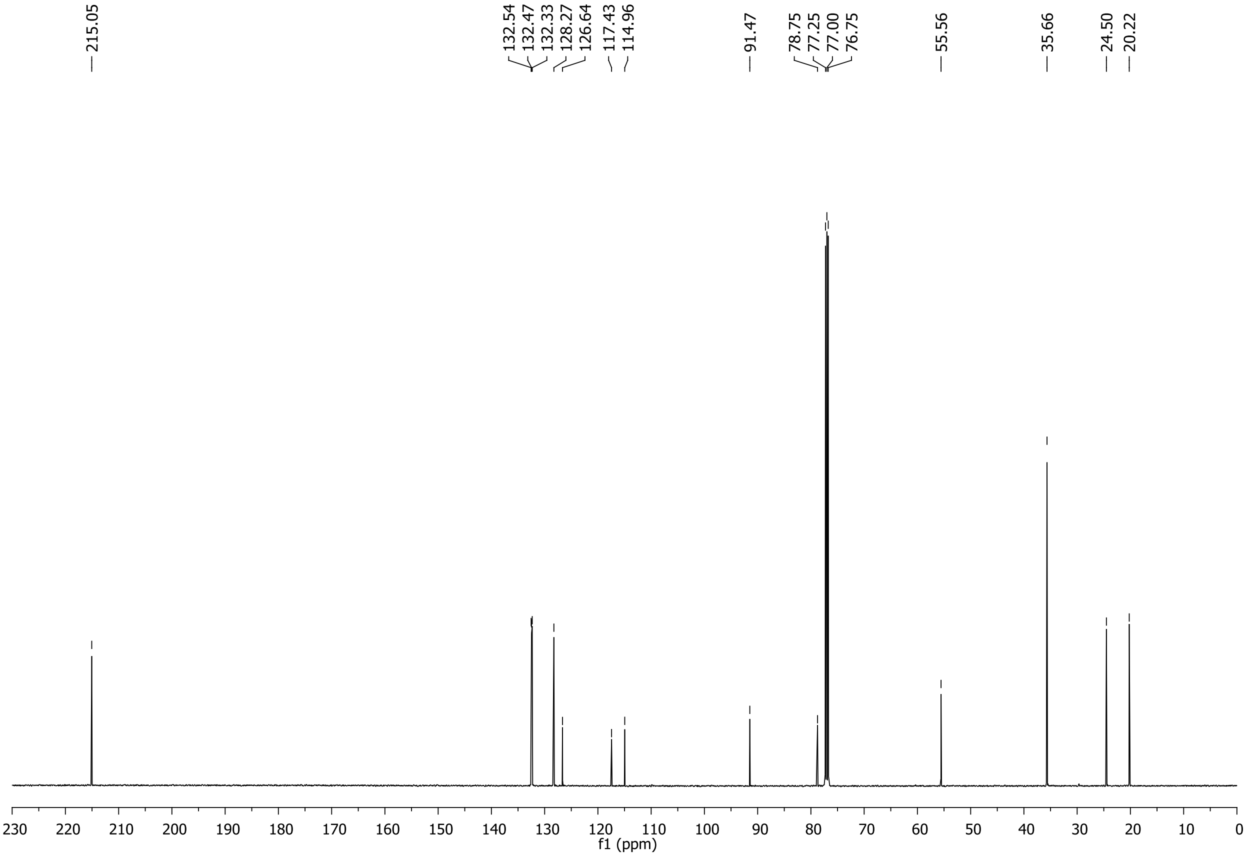
215.05
132.54
132.47
132.33
128.27
126.64
117.43
114.96
91.47
78.75
77.25
77.00
76.75
55.56
35.66
24.50
20.22
230 220 210 200 190 180 170 160 150 140 130 120 110 100 90 80 70 60 50 40 30 20 10 0
f1 (ppm)

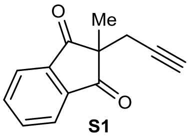

**S1**

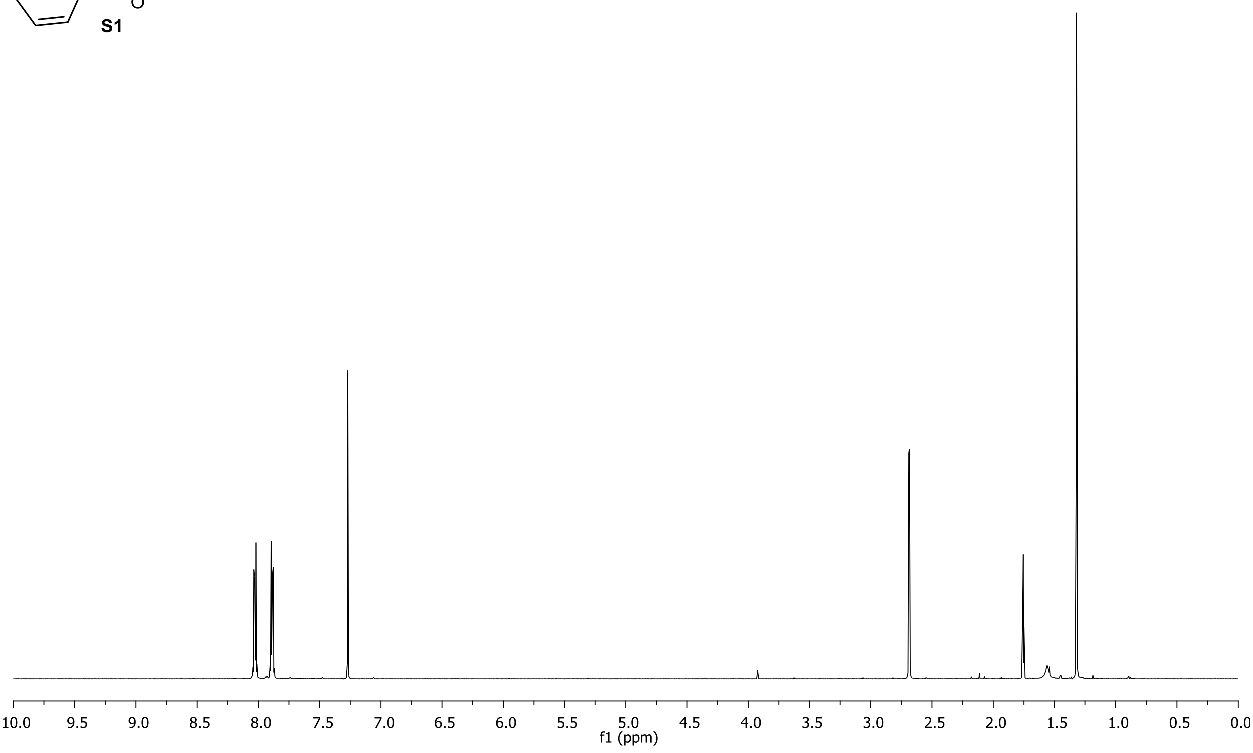
10.0
9.5
9.0
8.5
8.0
7.5
7.0
6.5
6.0
5.5
5.0
4.5
4.0
3.5
3.0
2.5
2.0
1.5
1.0
0.5
f1 (ppm)

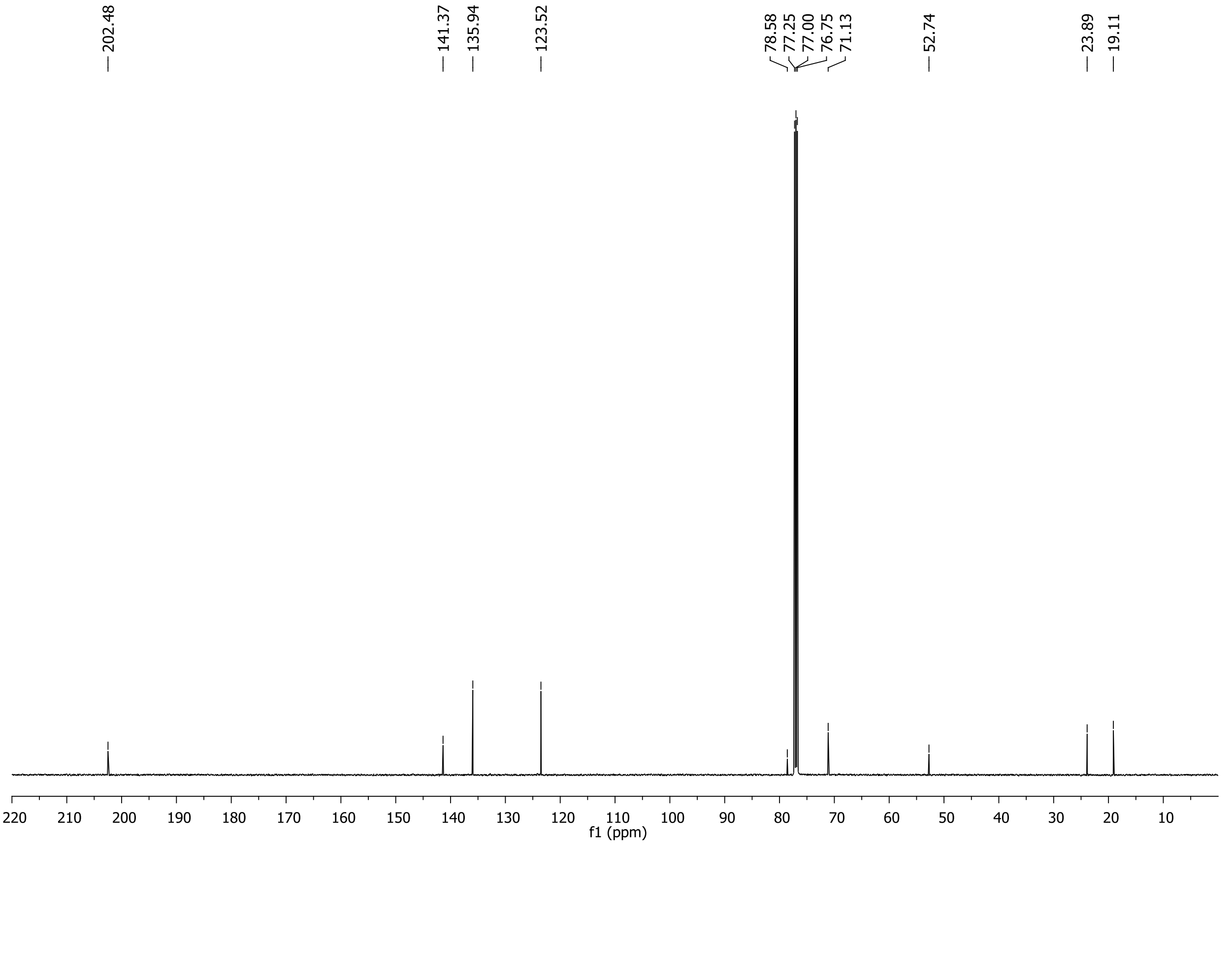
202.48
141.37
135.94
123.52
78.58
77.25
77.00
76.75
71.13
52.74
23.89
19.11
220
210
200
190
180
170
160
150
140
130
120
110
100
90
80
70
60
50
40
30
20
10
f1 (ppm)

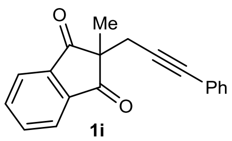
O
Me
Ph
O

**1i**

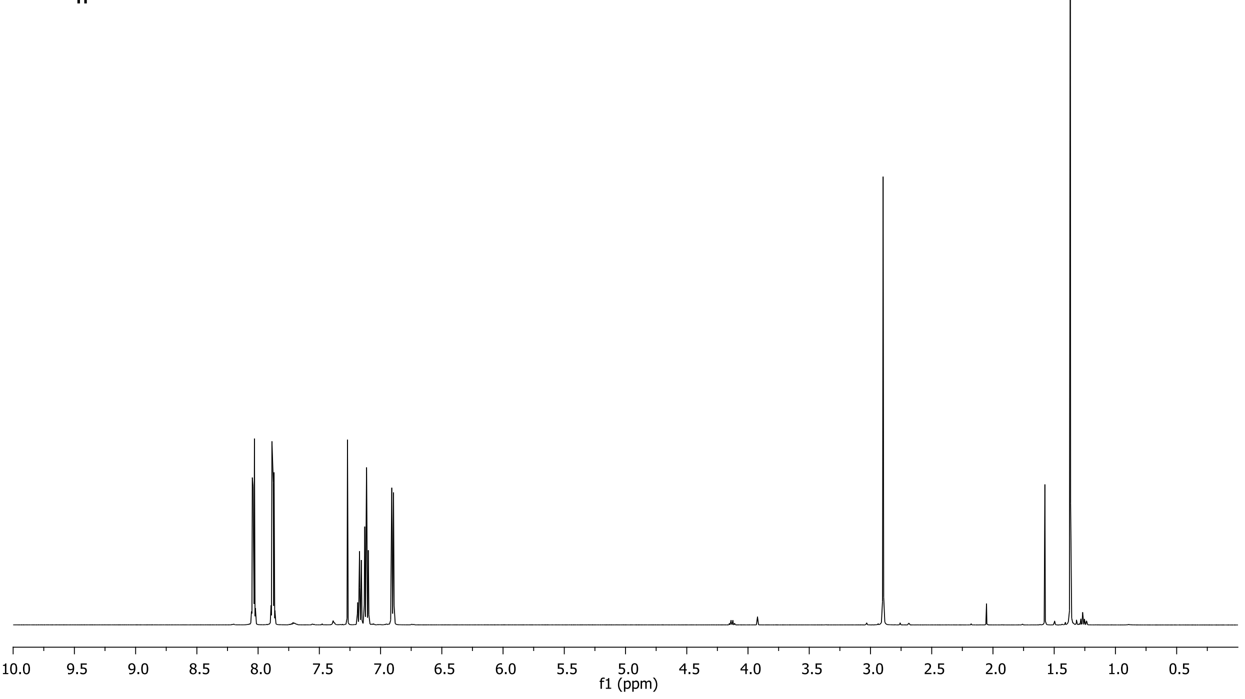
10.0 9.5 9.0 8.5 8.0 7.5 7.0 6.5 6.0 5.5 5.0 4.5 4.0 3.5 3.0 2.5 2.0 1.5 1.0 0.5
f1 (ppm)

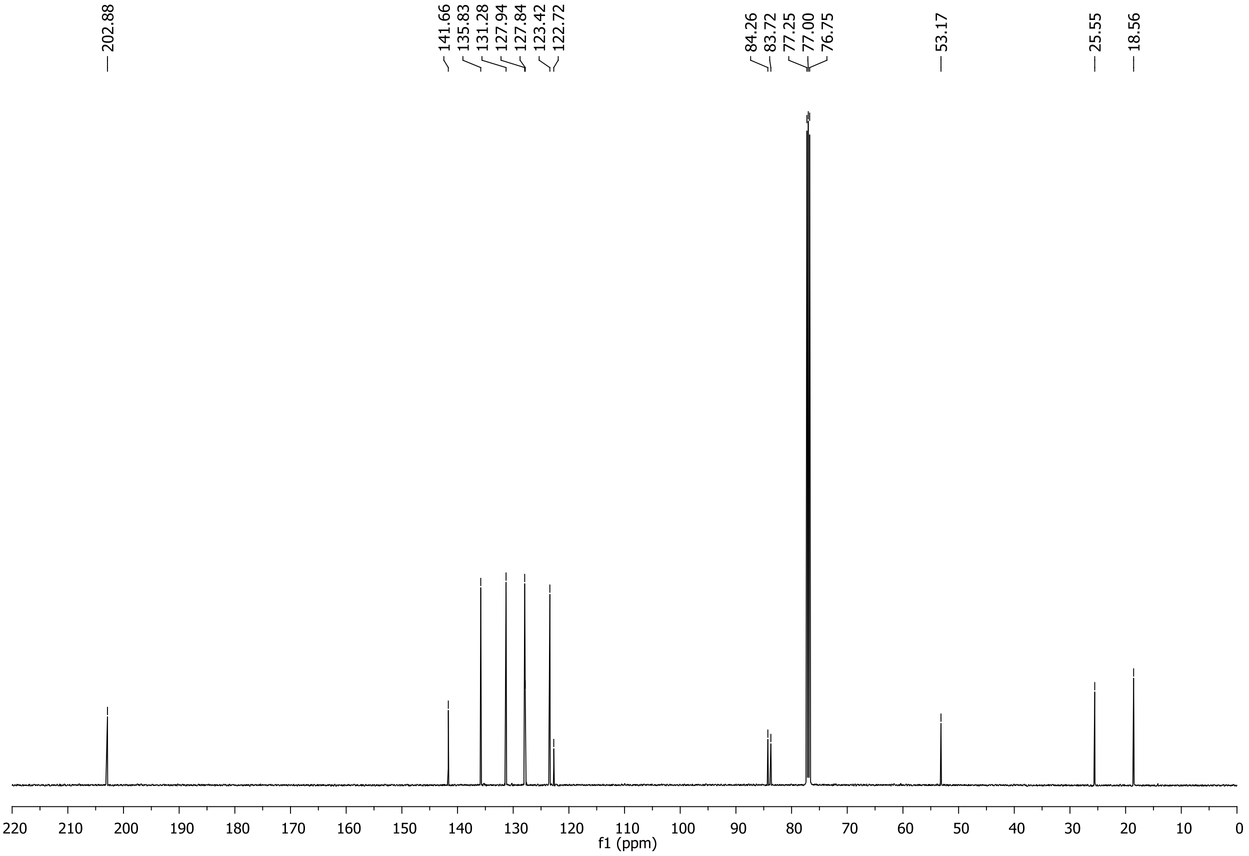
202.88
141.66
135.83
131.28
127.94
127.84
123.42
122.72
84.26
83.72
77.25
77.00
76.75
53.17
25.55
18.56
220 210 200 190 180 170 160 150 140 130 120 110 100 90 80 70 60 50 40 30 20 10 0
f1 (ppm)

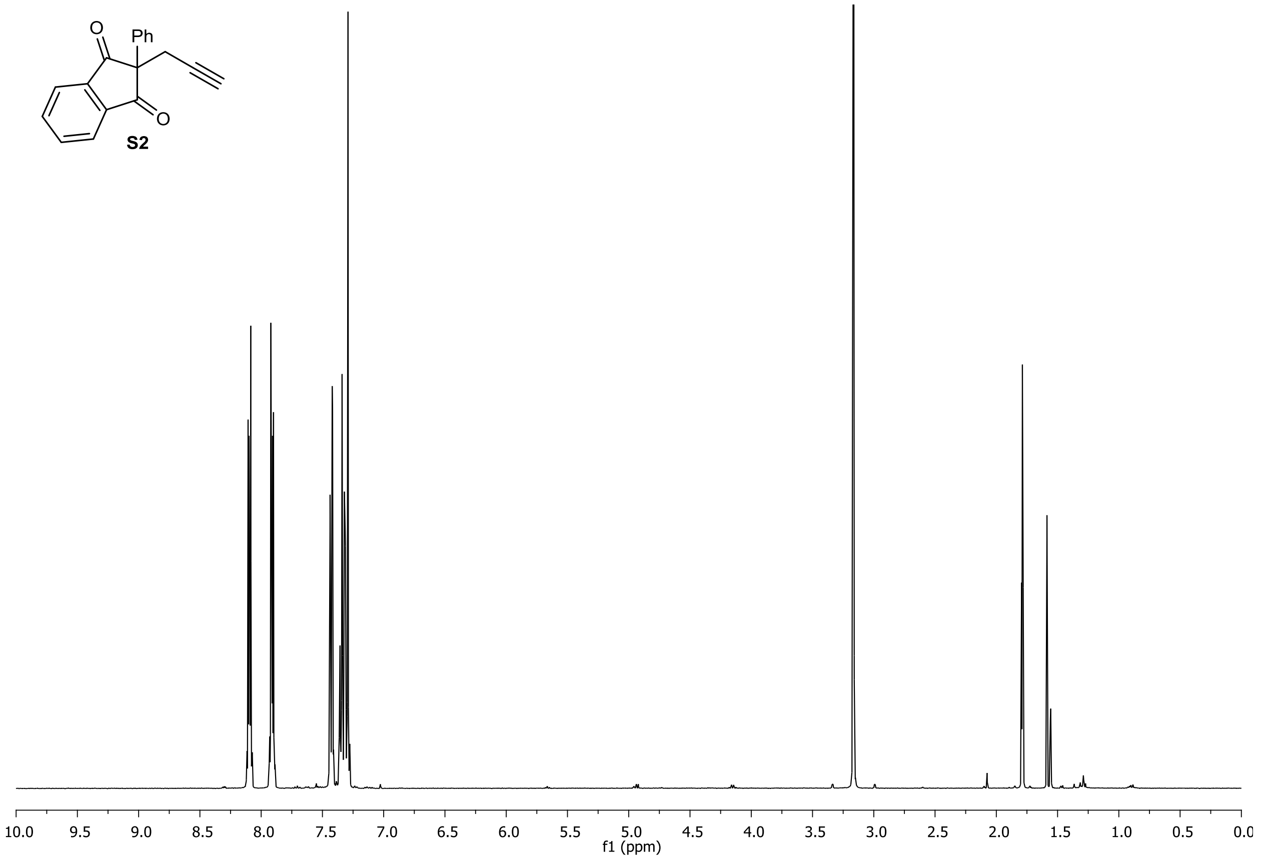
O
Ph
O
S2
10.0 9.5 9.0 8.5 8.0 7.5 7.0 6.5 6.0 5.5 5.0 4.5 4.0 3.5 3.0 2.5 2.0 1.5 1.0 0.5 0.0
f1 (ppm)

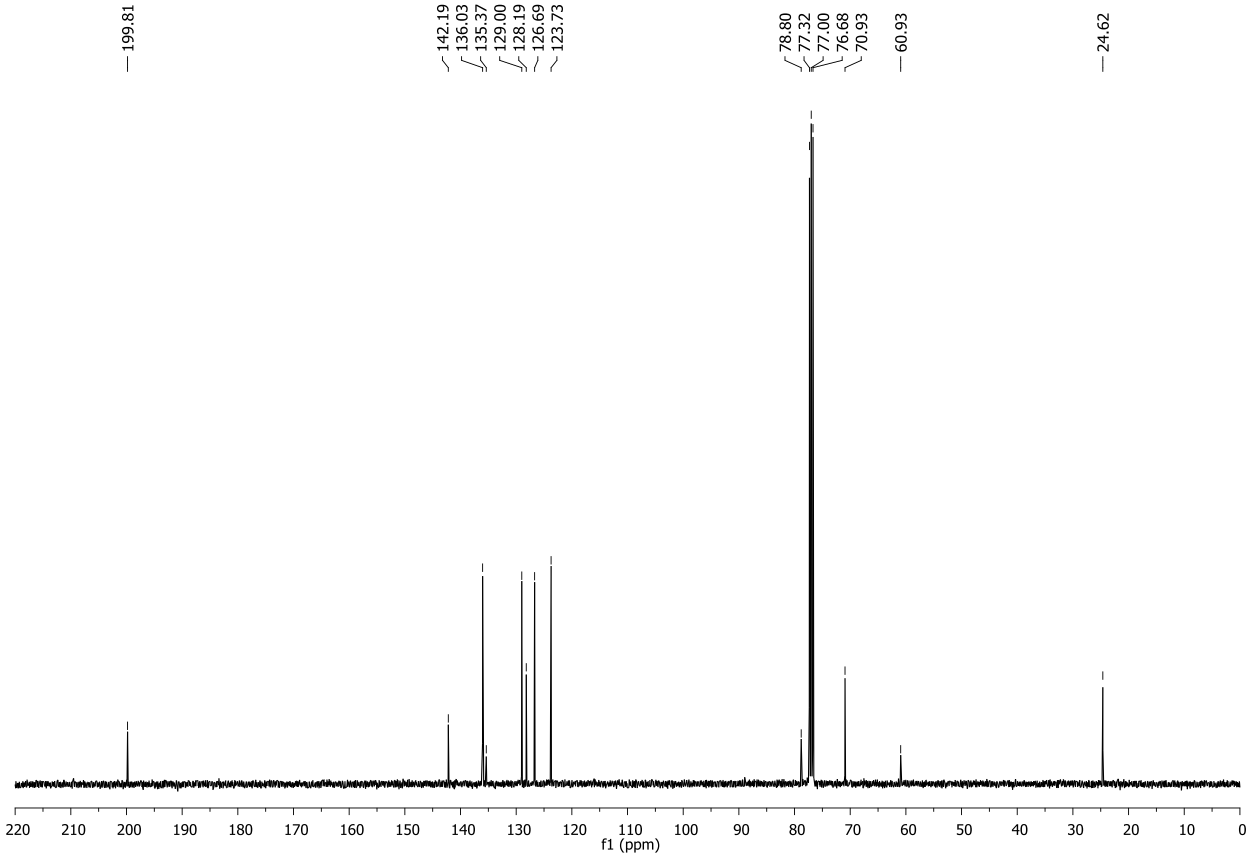
199.81
142.19
136.03
135.37
129.00
128.19
126.69
123.73
78.80
77.32
77.00
76.68
70.93
60.93
24.62
220 210 200 190 180 170 160 150 140 130 120 110 100 90 80 70 60 50 40 30 20 10 0
f1 (ppm)

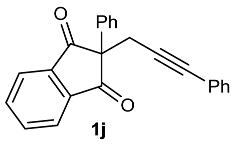
O
Ph
Ph
O

**1j**

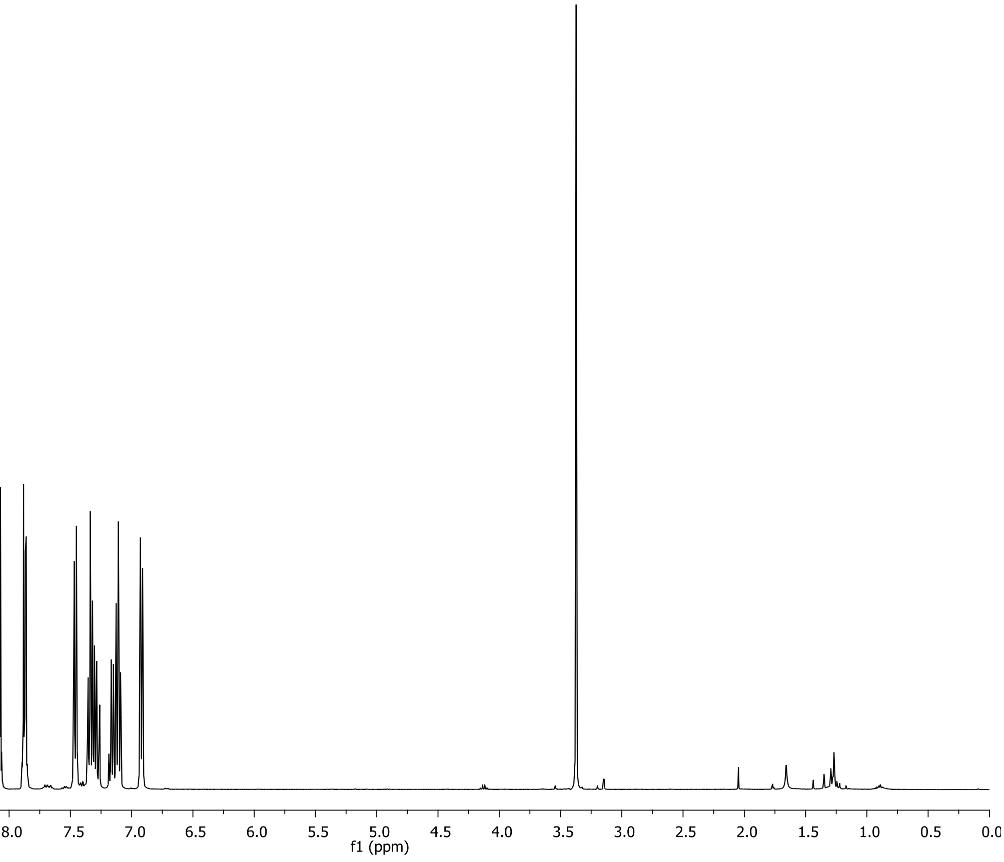
10.0
9.5
9.0
8.5
8.0
7.5
7.0
6.5
6.0
5.5
5.0
4.5
4.0
3.5
3.0
2.5
2.0
1.5
1.0
0.5
0.0
f1 (ppm)

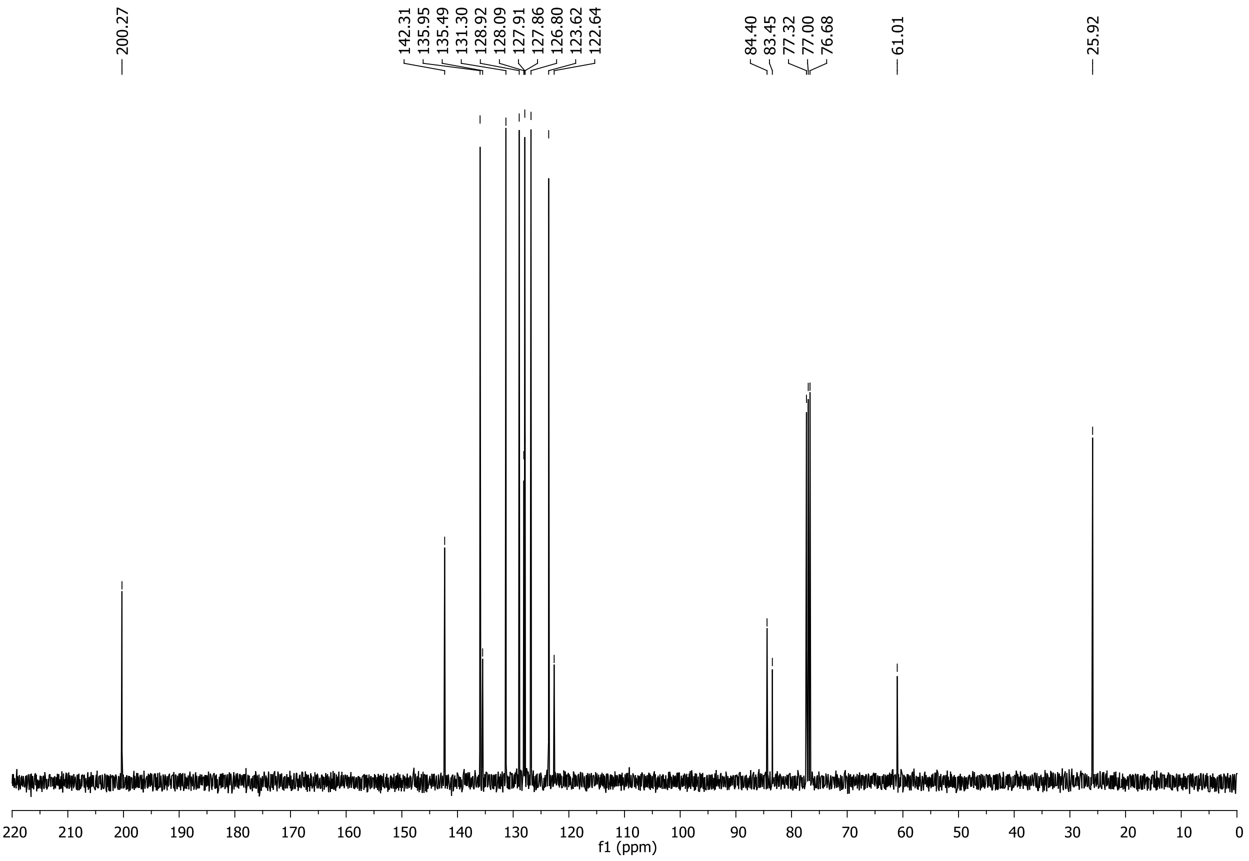
200.27
142.31
135.95
135.49
131.30
128.92
128.09
127.91
127.86
126.80
123.62
122.64
84.40
83.45
77.32
77.00
76.68
61.01
25.92
220
210
200
190
180
170
160
150
140
130
120
110
100
90
80
70
60
50
40
30
20
10
0
f1 (ppm)

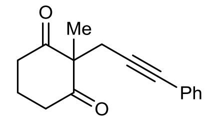
O
Me
Ph
O

**1k**

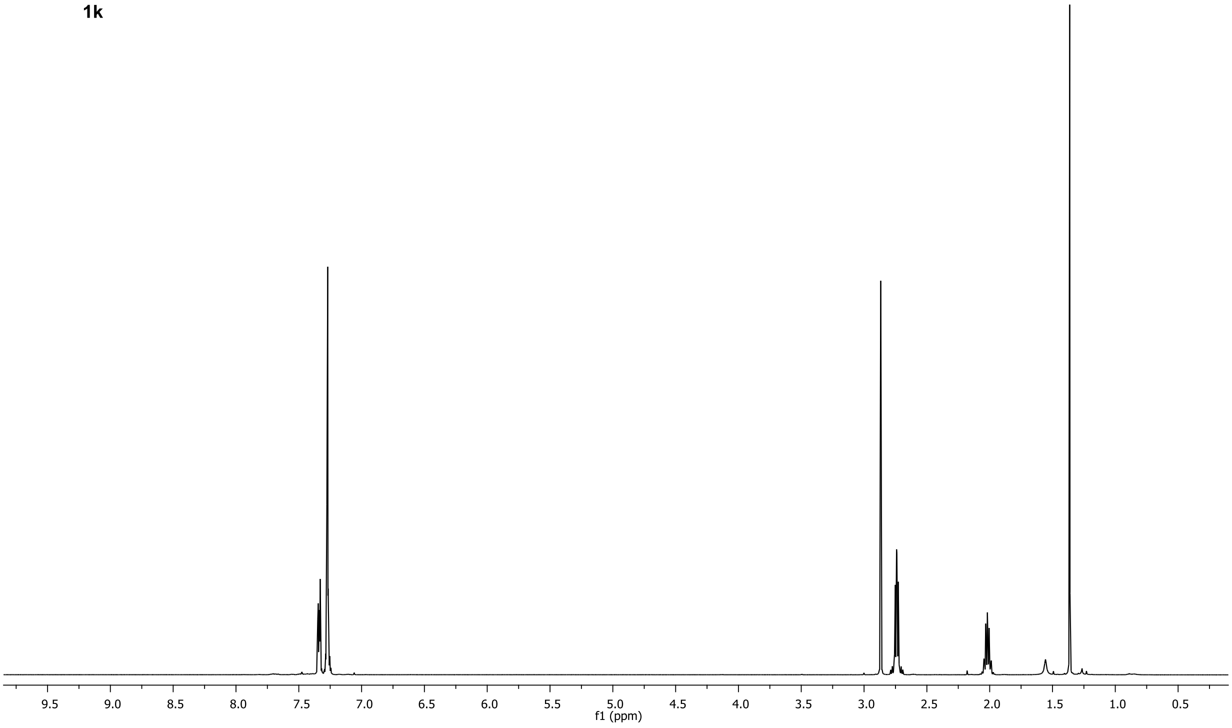
9.5 9.0 8.5 8.0 7.5 7.0 6.5 6.0 5.5 5.0 4.5 4.0 3.5 3.0 2.5 2.0 1.5 1.0 0.5
f1 (ppm)

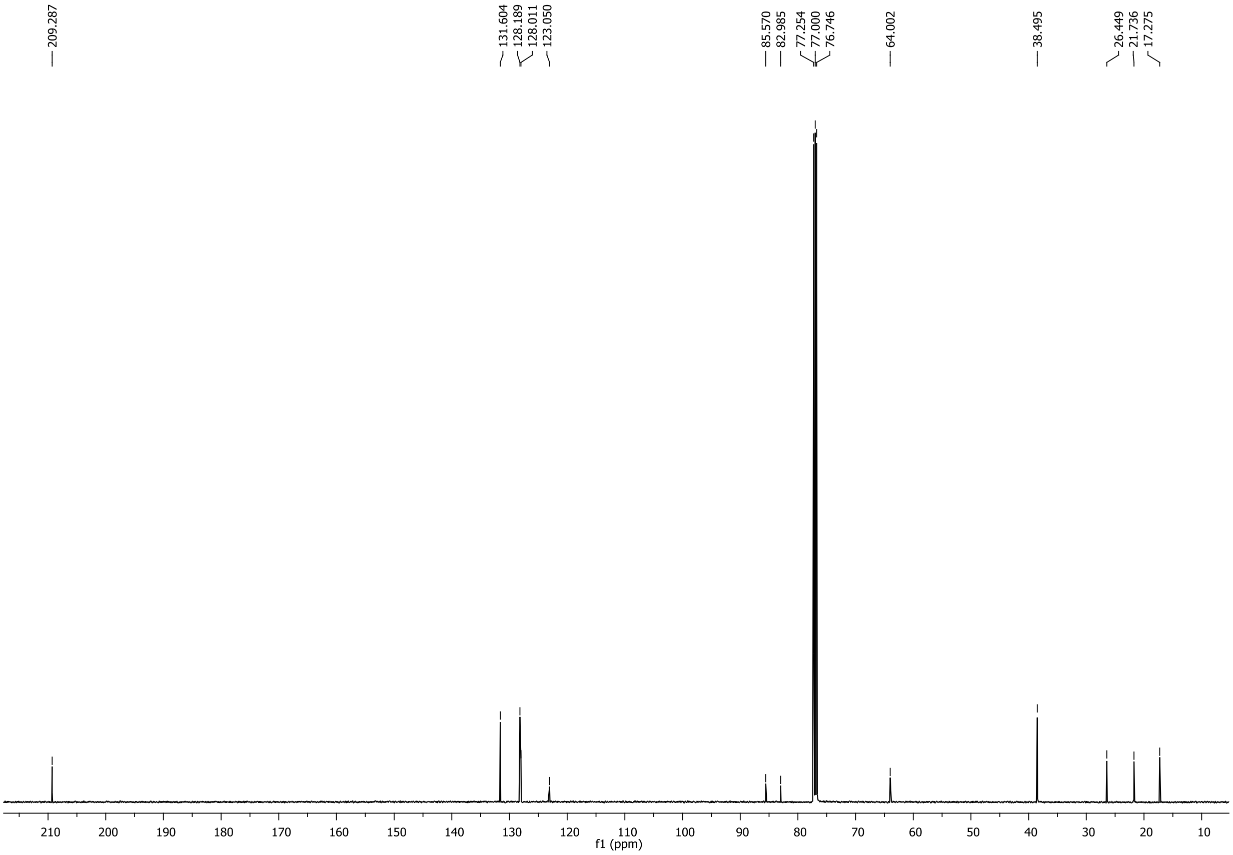
209.287
131.604
128.189
128.011
123.050
85.570
82.985
77.254
77.000
76.746
64.002
38.495
26.449
21.736
17.275
210 200 190 180 170 160 150 140 130 120 110 100 90 80 70 60 50 40 30 20 10
f1 (ppm)

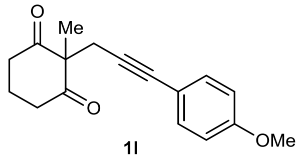
O
Me
O
OMe

**1l**

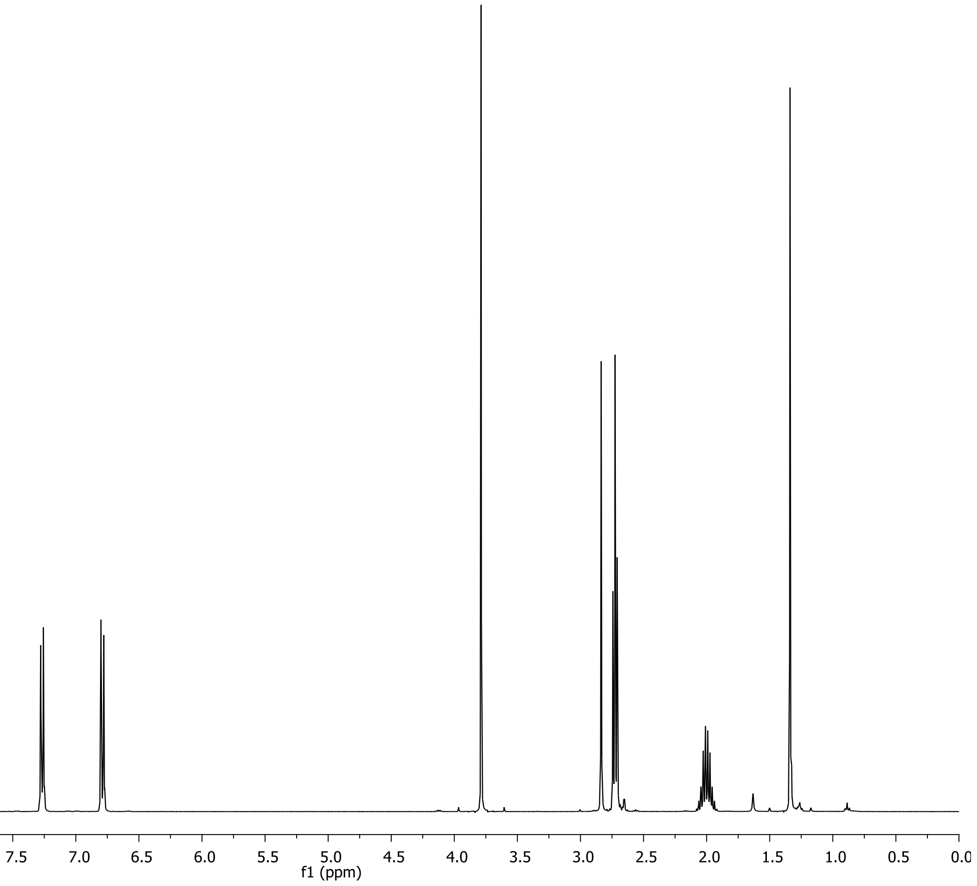
10.0
9.5
9.0
8.5
8.0
7.5
7.0
6.5
6.0
5.5
5.0
4.5
4.0
3.5
3.0
2.5
2.0
1.5
1.0
0.5
0.0
f1 (ppm)

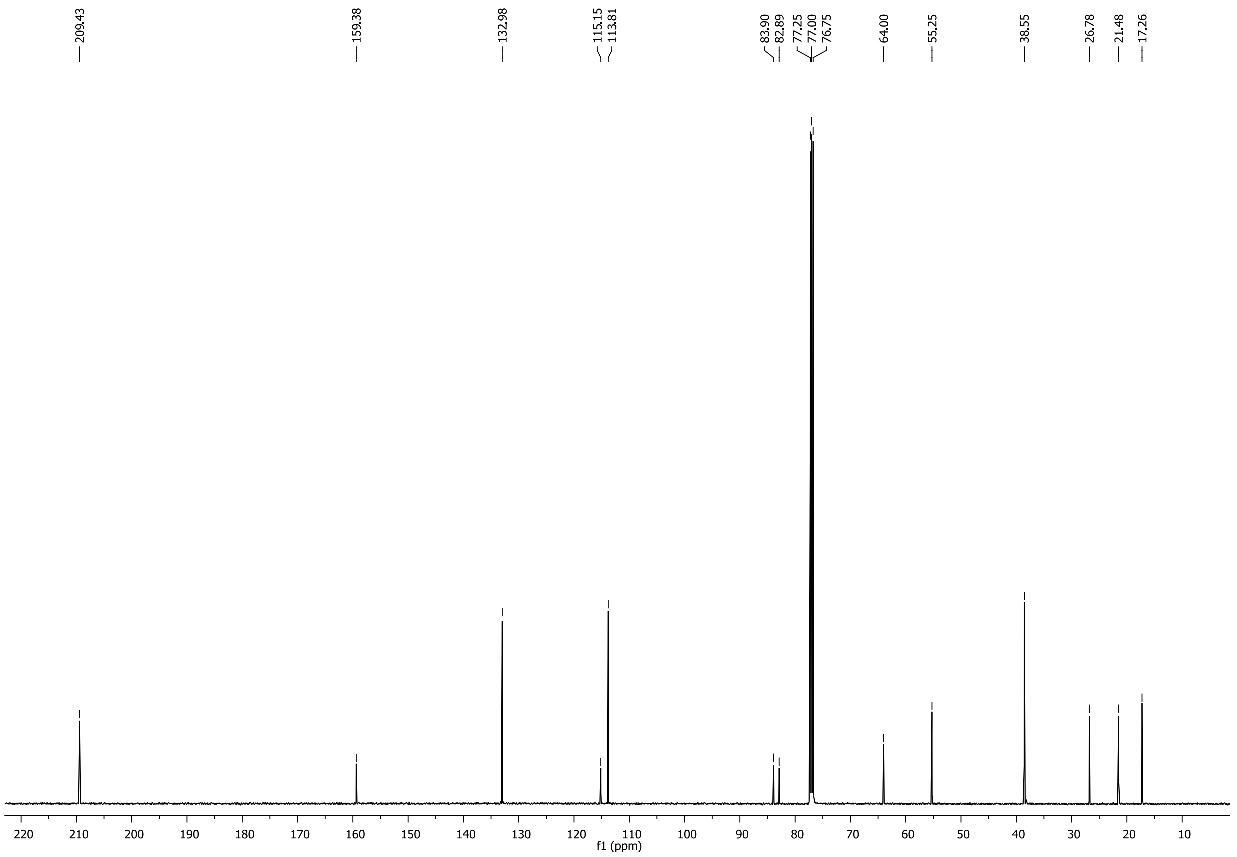
209.43
159.38
132.98
115.15
113.81
83.90
82.89
77.25
77.00
76.75
64.00
55.25
38.55
26.78
21.48
17.26
220
210
200
190
180
170
160
150
140
130
120
110
100
90
80
70
60
50
40
30
20
10
f1 (ppm)

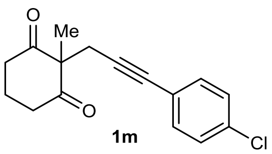
O
Me
O
Cl

**1m**

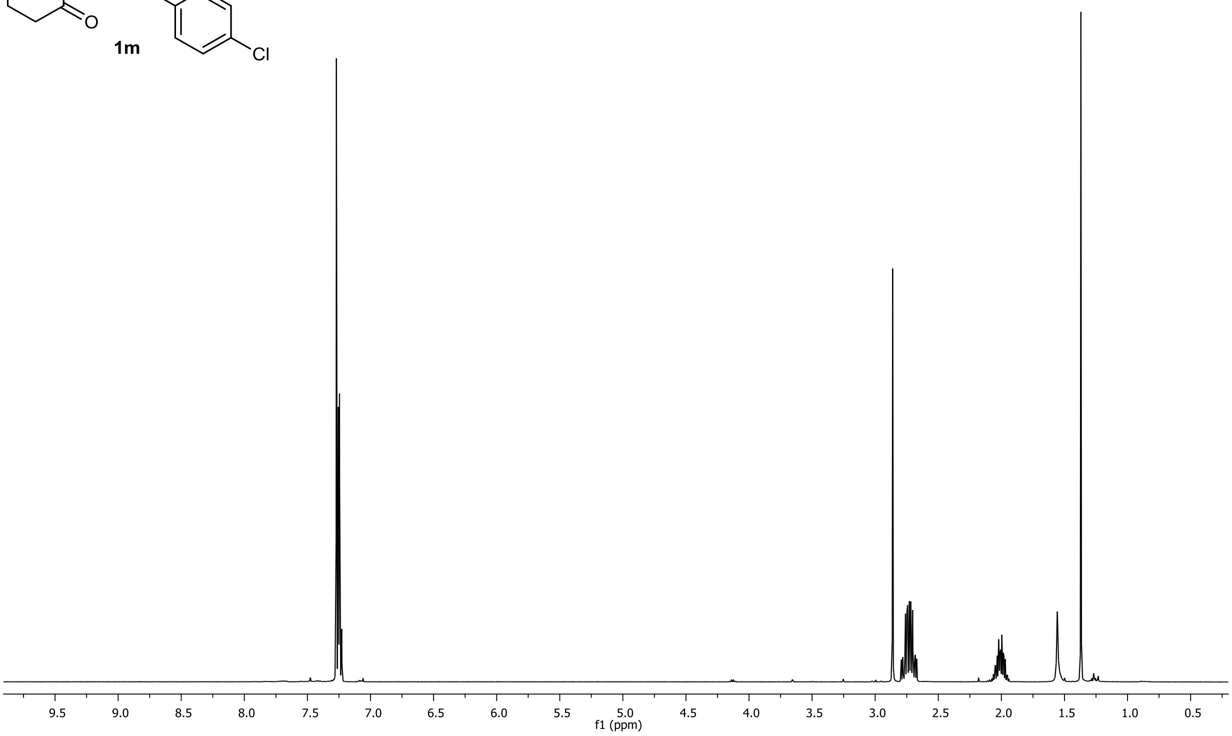
9.5 9.0 8.5 8.0 7.5 7.0 6.5 6.0 5.5 5.0 4.5 4.0 3.5 3.0 2.5 2.0 1.5 1.0 0.5
f1 (ppm)

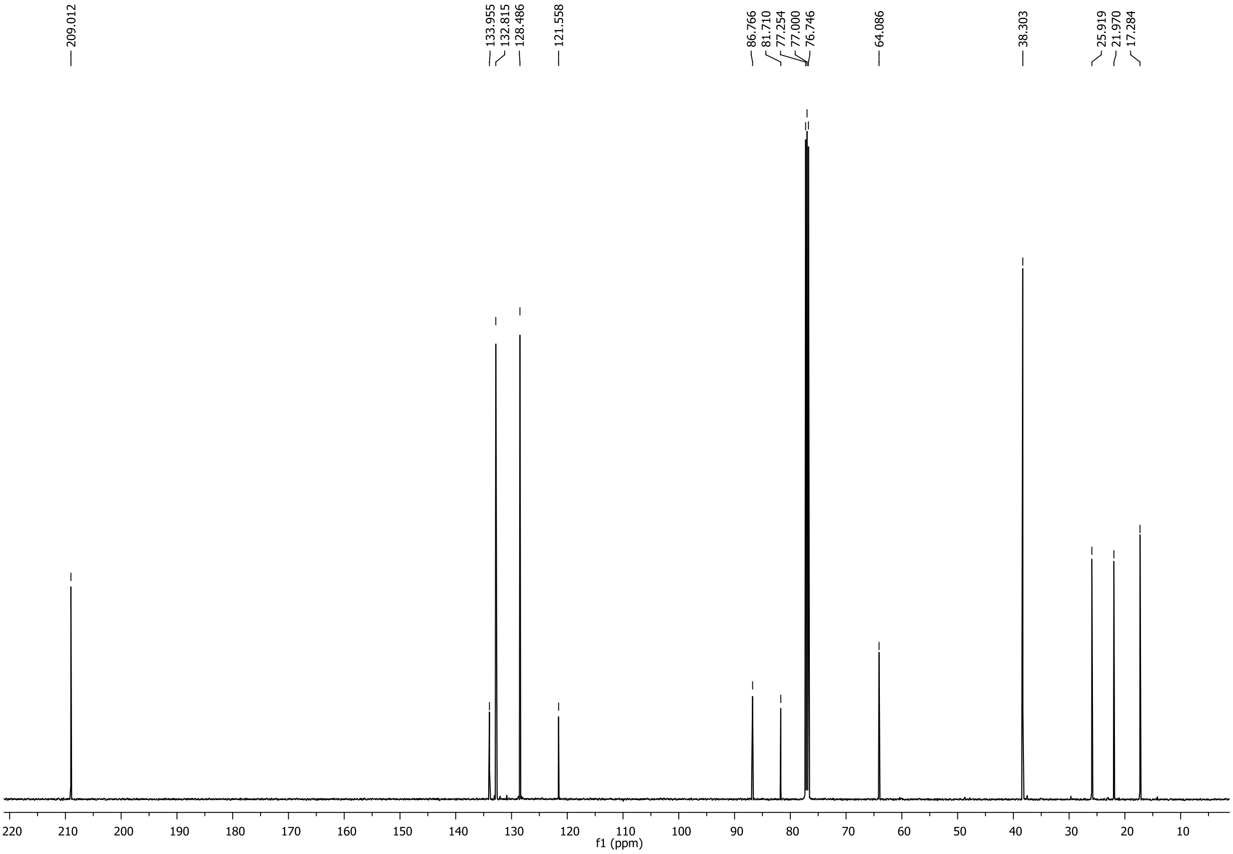
209.012
133.955
132.815
128.486
121.558
86.766
81.710
77.254
77.000
76.746
64.086
38.303
25.919
21.970
17.284
220 210 200 190 180 170 160 150 140 130 120 110 100 90 80 70 60 50 40 30 20 10
f1 (ppm)

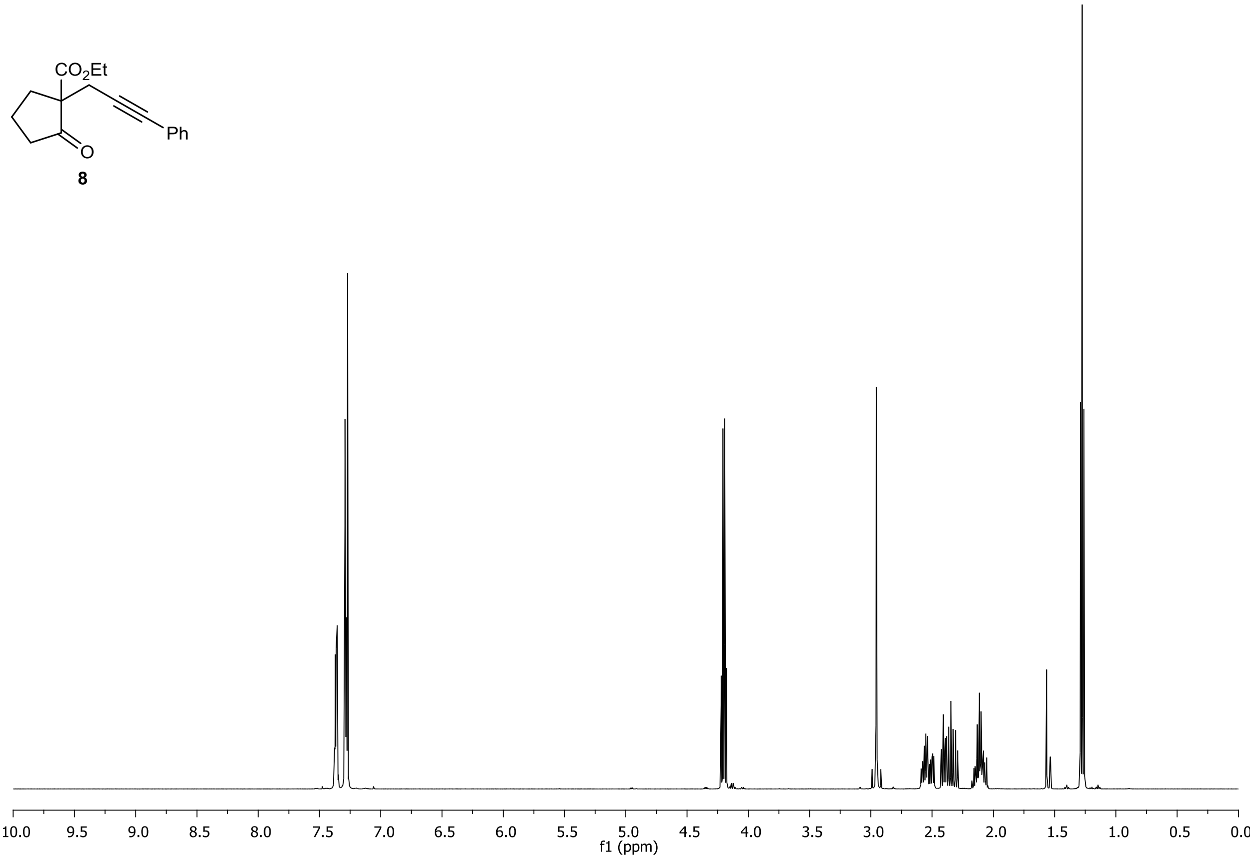
CO2Et
Ph
O
8
10.0 9.5 9.0 8.5 8.0 7.5 7.0 6.5 6.0 5.5 5.0 4.5 4.0 3.5 3.0 2.5 2.0 1.5 1.0 0.5 0.0
f1 (ppm)

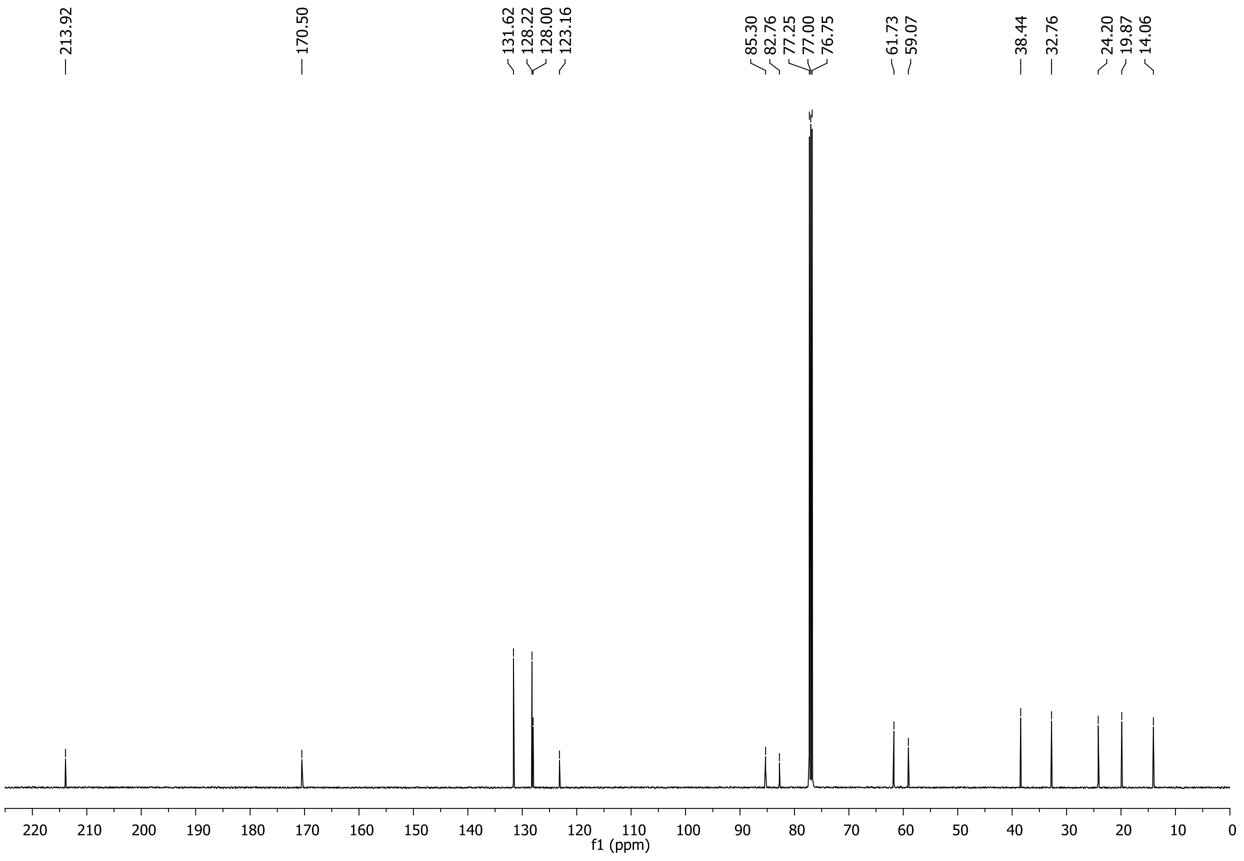
213.92
170.50
131.62
128.22
128.00
123.16
85.30
82.76
77.25
77.00
76.75
61.73
59.07
38.44
32.76
24.20
19.87
14.06
220 210 200 190 180 170 160 150 140 130 120 110 100 90 80 70 60 50 40 30 20 10 0
f1 (ppm)

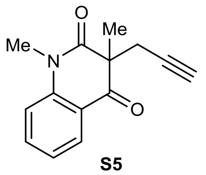
Me
Me
N
O
O

**S5**

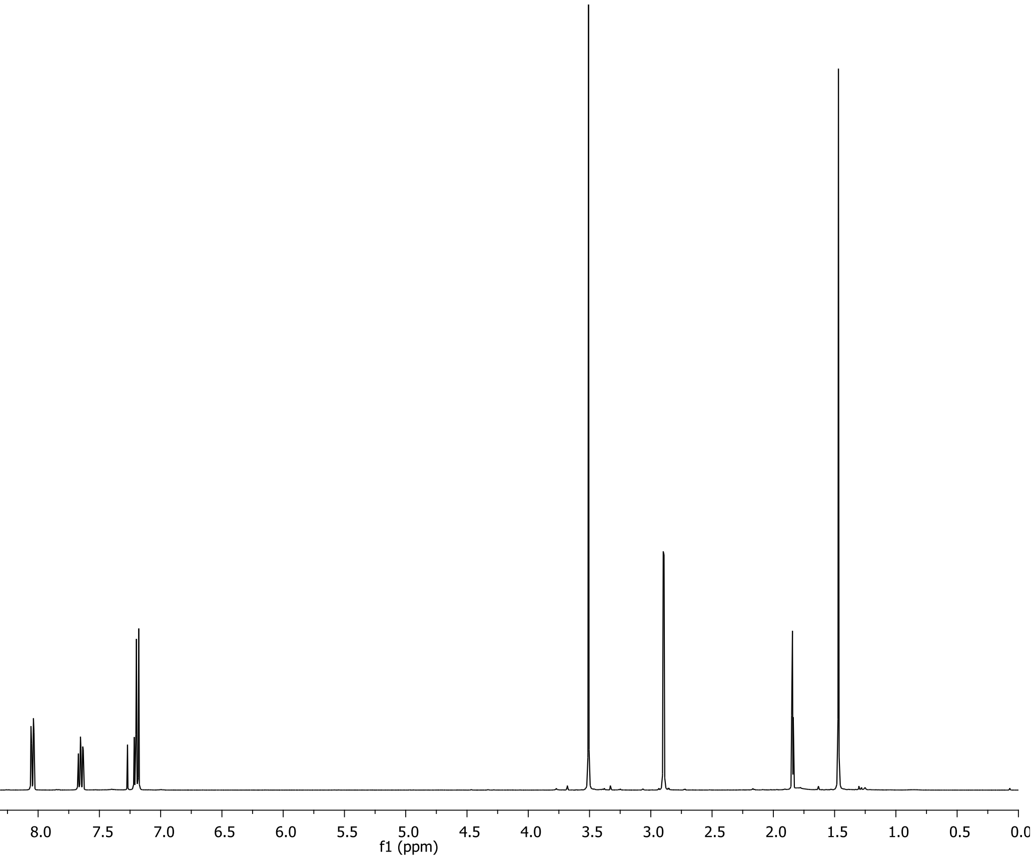
10.0 9.5 9.0 8.5 8.0 7.5 7.0 6.5 6.0 5.5 5.0 4.5 4.0 3.5 3.0 2.5 2.0 1.5 1.0 0.5 0.0
f1 (ppm)

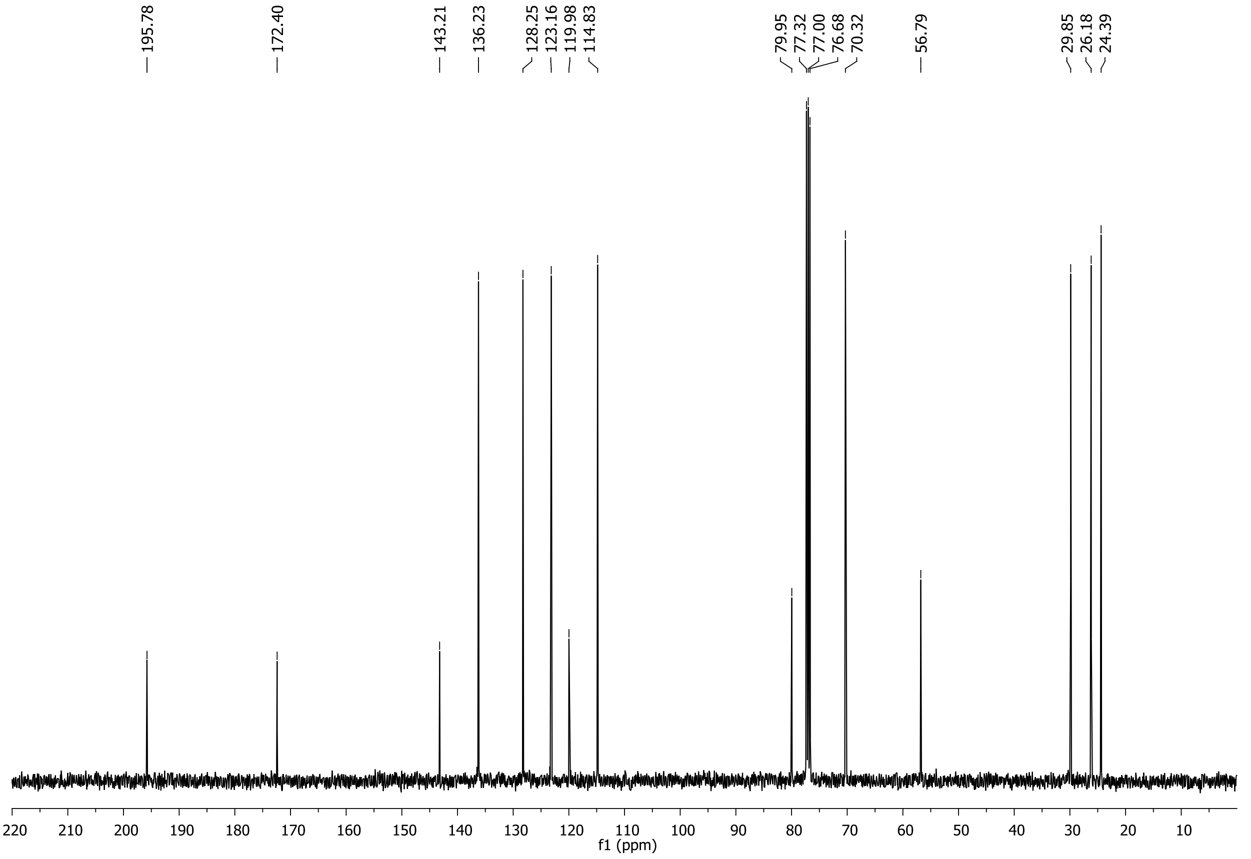
195.78
172.40
143.21
136.23
128.25
123.16
119.98
114.83
79.95
77.32
77.00
76.68
70.32
56.79
29.85
26.18
24.39
220 210 200 190 180 170 160 150 140 130 120 110 100 90 80 70 60 50 40 30 20 10
f1 (ppm)

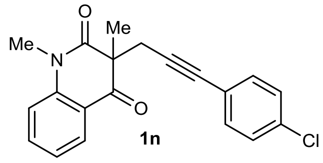
Me
N
O
Me
O
Cl

**1n**

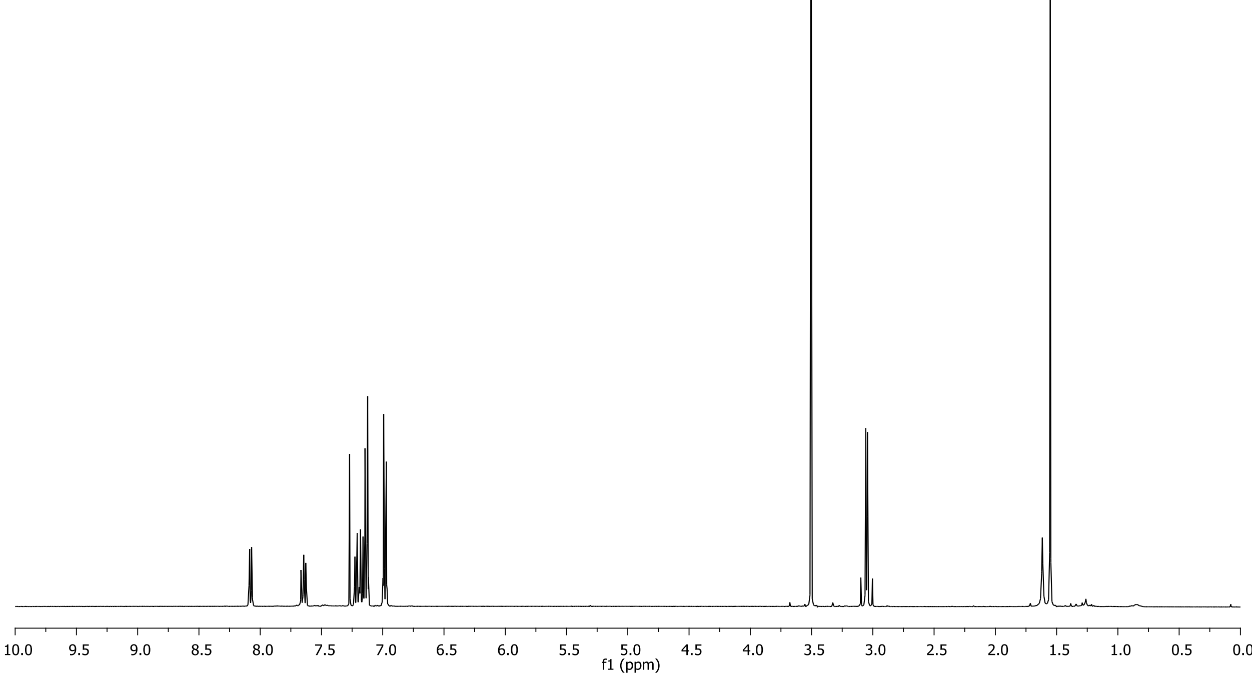
10.0
9.5
9.0
8.5
8.0
7.5
7.0
6.5
6.0
5.5
5.0
4.5
4.0
3.5
3.0
2.5
2.0
1.5
1.0
0.5
0.0
f1 (ppm)

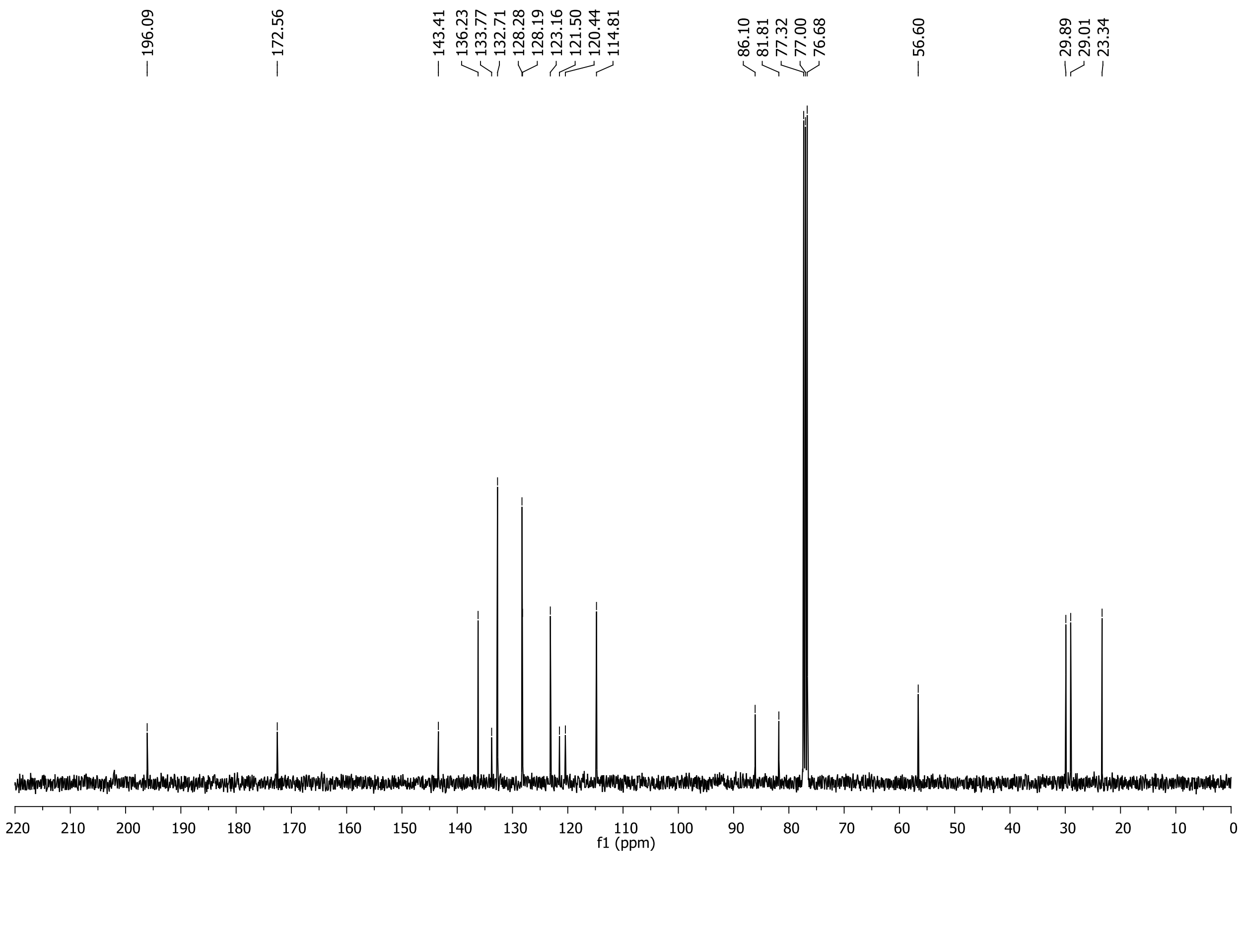
196.09
172.56
143.41
136.23
133.77
132.71
128.28
128.19
123.16
121.50
120.44
114.81
86.10
81.81
77.32
77.00
76.68
56.60
29.89
29.01
23.34
220
210
200
190
180
170
160
150
140
130
120
110
100
90
80
70
60
50
40
30
20
10
0
f1 (ppm)

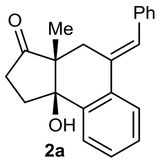
O
Me
Ph
OH

**2a**

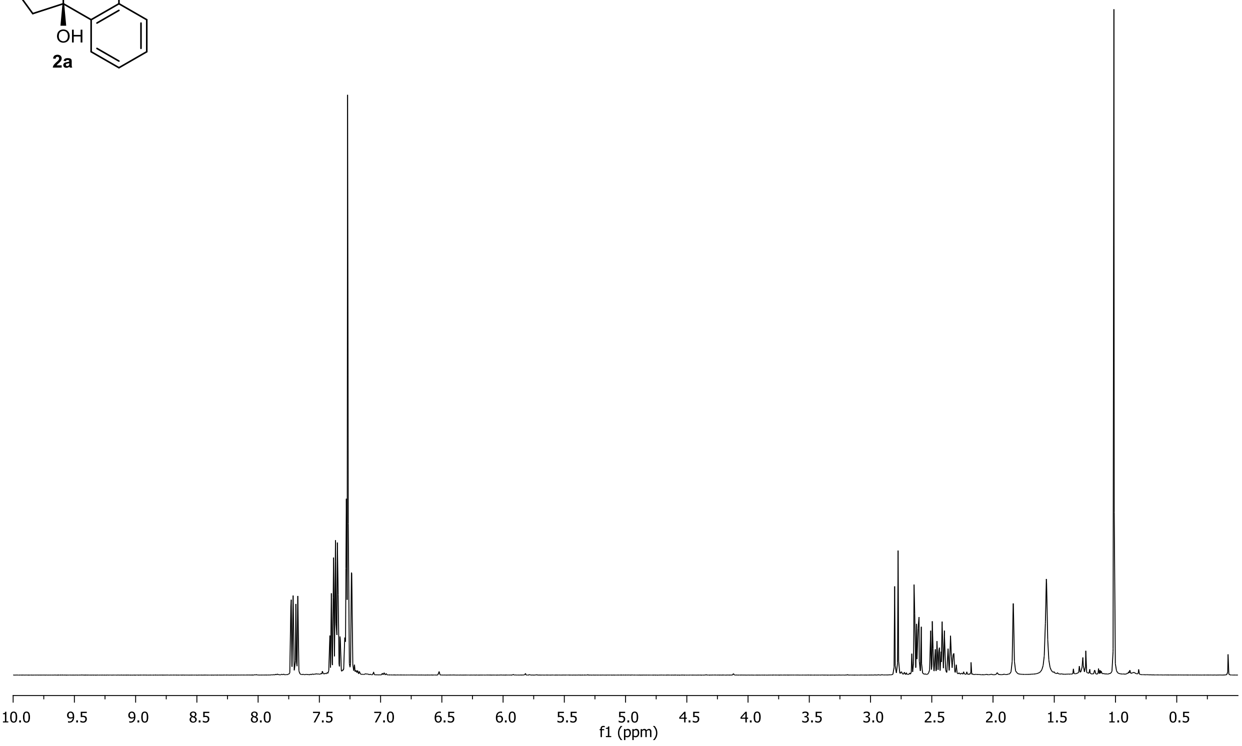
10.0 9.5 9.0 8.5 8.0 7.5 7.0 6.5 6.0 5.5 5.0 4.5 4.0 3.5 3.0 2.5 2.0 1.5 1.0 0.5
f1 (ppm)

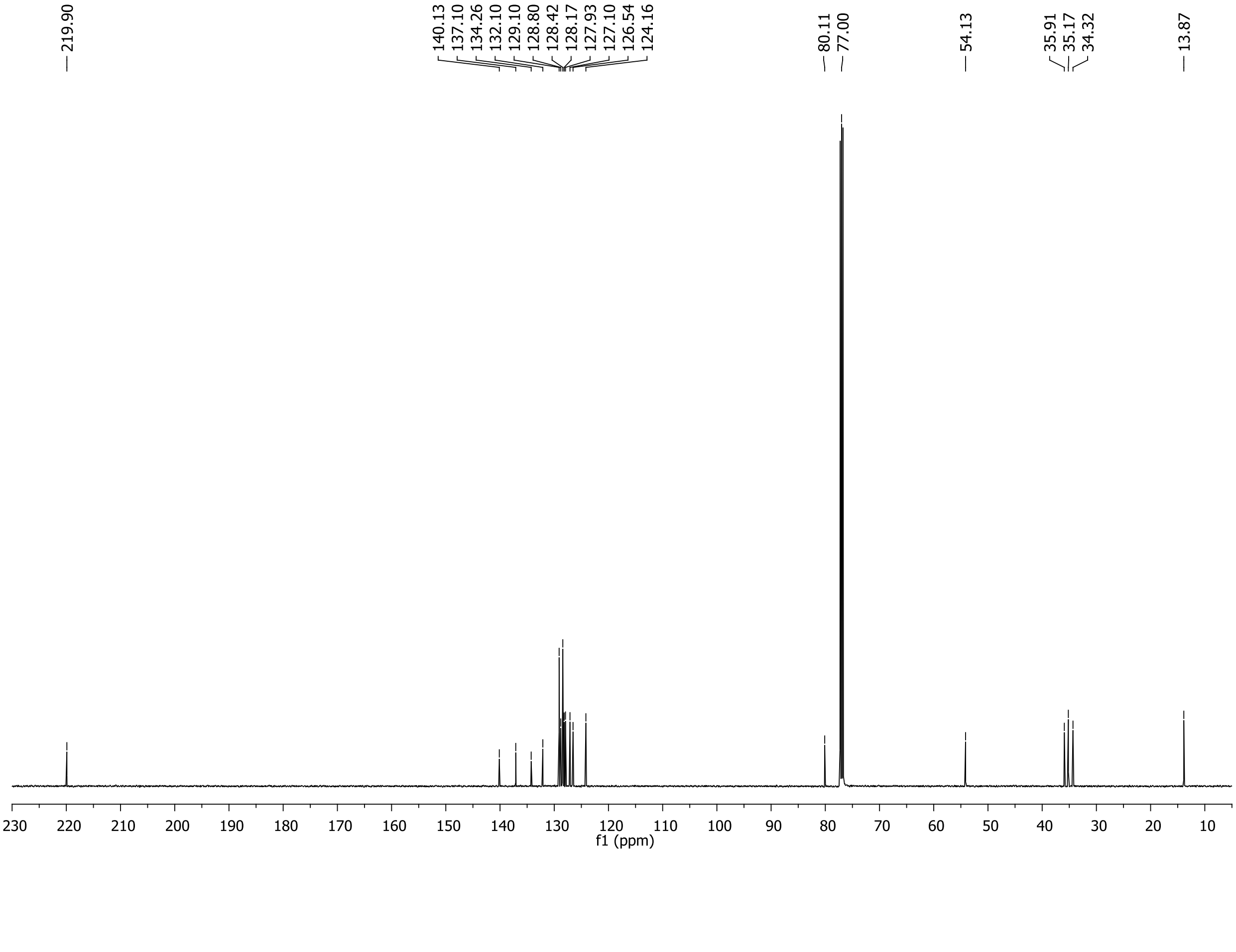
219.90
140.13
137.10
134.26
132.10
129.10
128.80
128.42
128.17
127.93
127.10
126.54
124.16
80.11
77.00
54.13
35.91
35.17
34.32
13.87
230 220 210 200 190 180 170 160 150 140 130 120 110 100 90 80 70 60 50 40 30 20 10
f1 (ppm)

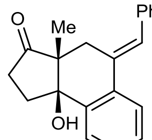
O
Me
Ph
OH
**2a**

HMBC

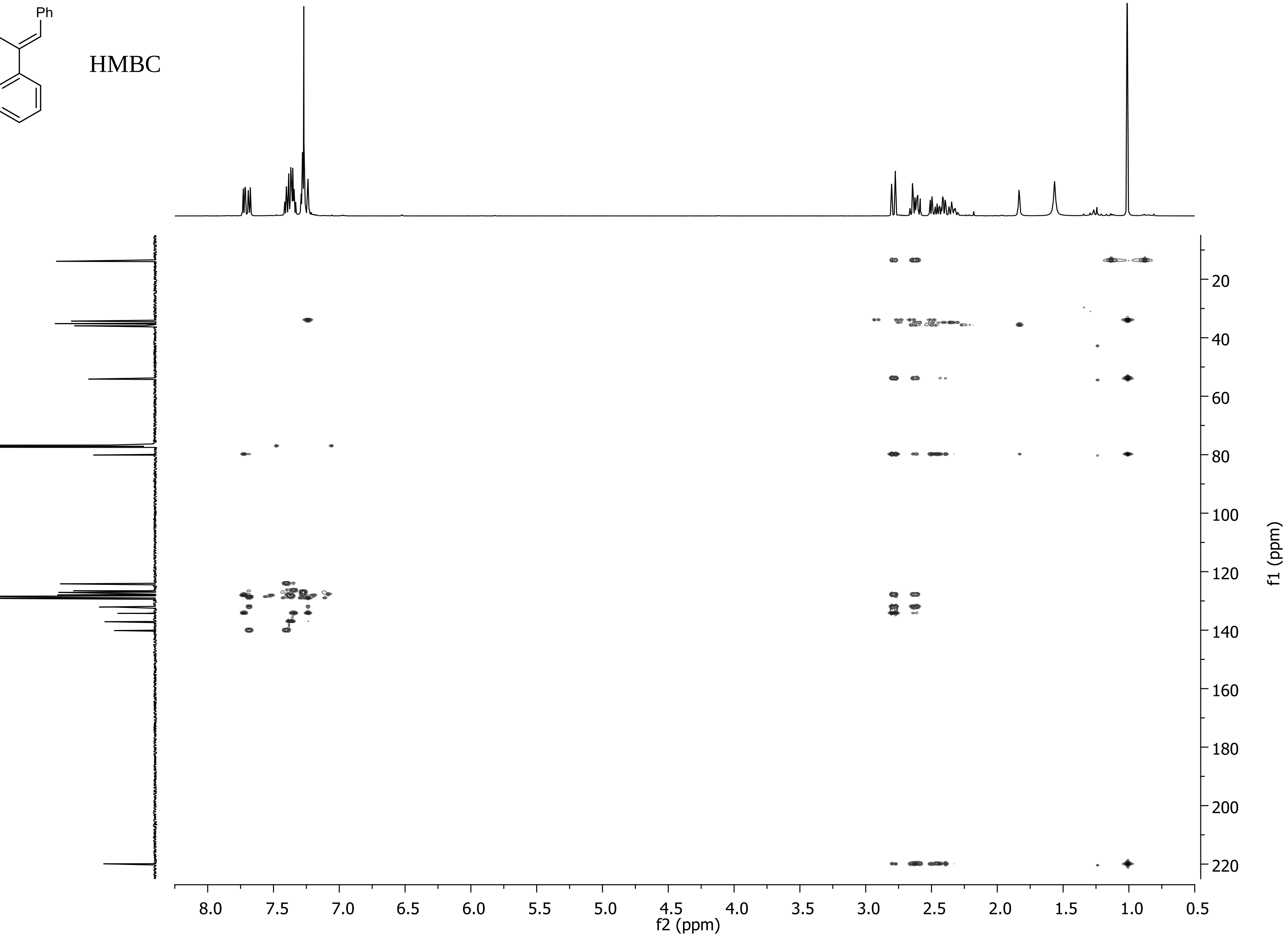
f1 (ppm)
f2 (ppm)
20
40
60
80
100
120
140
160
180
200
220
8.0
7.5
7.0
6.5
6.0
5.5
5.0
4.5
4.0
3.5
3.0
2.5
2.0
1.5
1.0
0.5

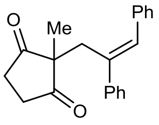
O
Me
Ph
Ph
O

**5**

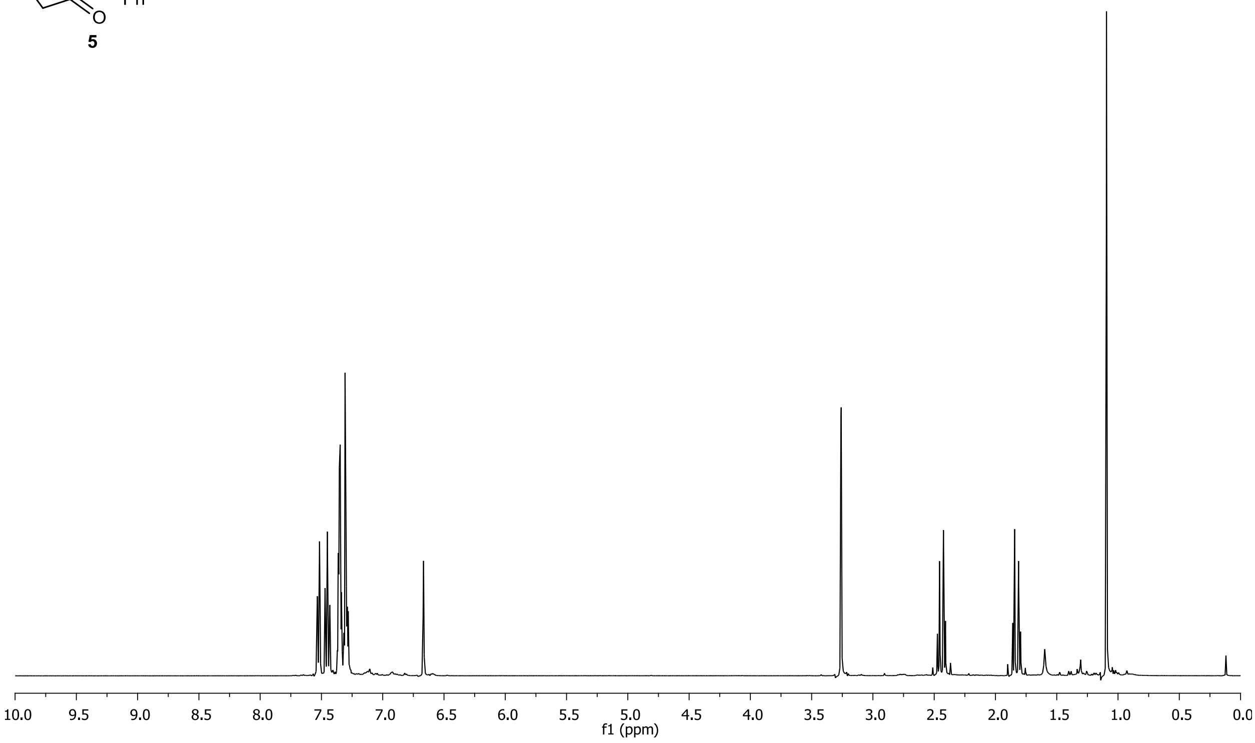
10.0 9.5 9.0 8.5 8.0 7.5 7.0 6.5 6.0 5.5 5.0 4.5 4.0 3.5 3.0 2.5 2.0 1.5 1.0 0.5 0.0
f1 (ppm)

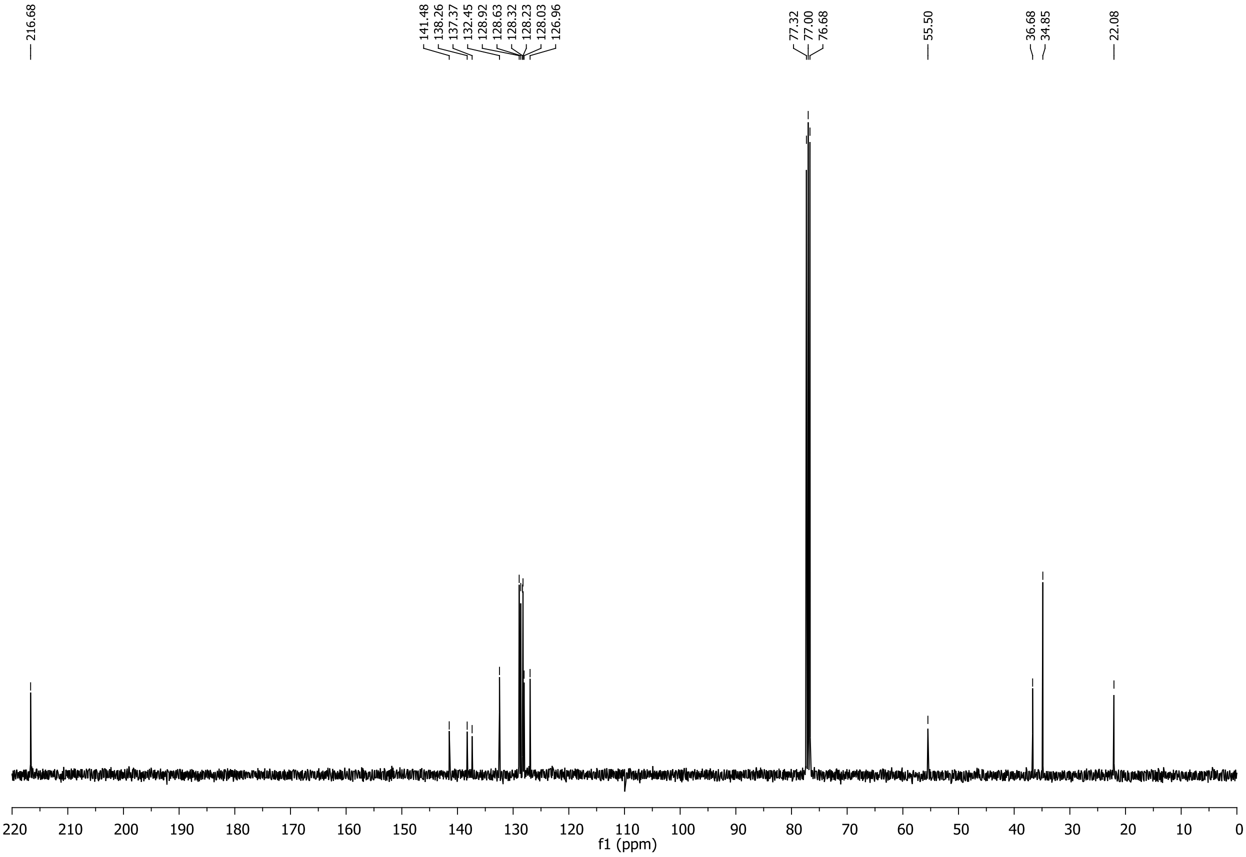
216.68
141.48
138.26
137.37
132.45
128.92
128.63
128.32
128.23
128.03
126.96
77.32
77.00
76.68
55.50
36.68
34.85
22.08
220 210 200 190 180 170 160 150 140 130 120 110 100 90 80 70 60 50 40 30 20 10 0
f1 (ppm)

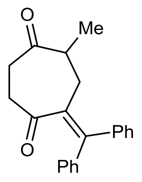
6

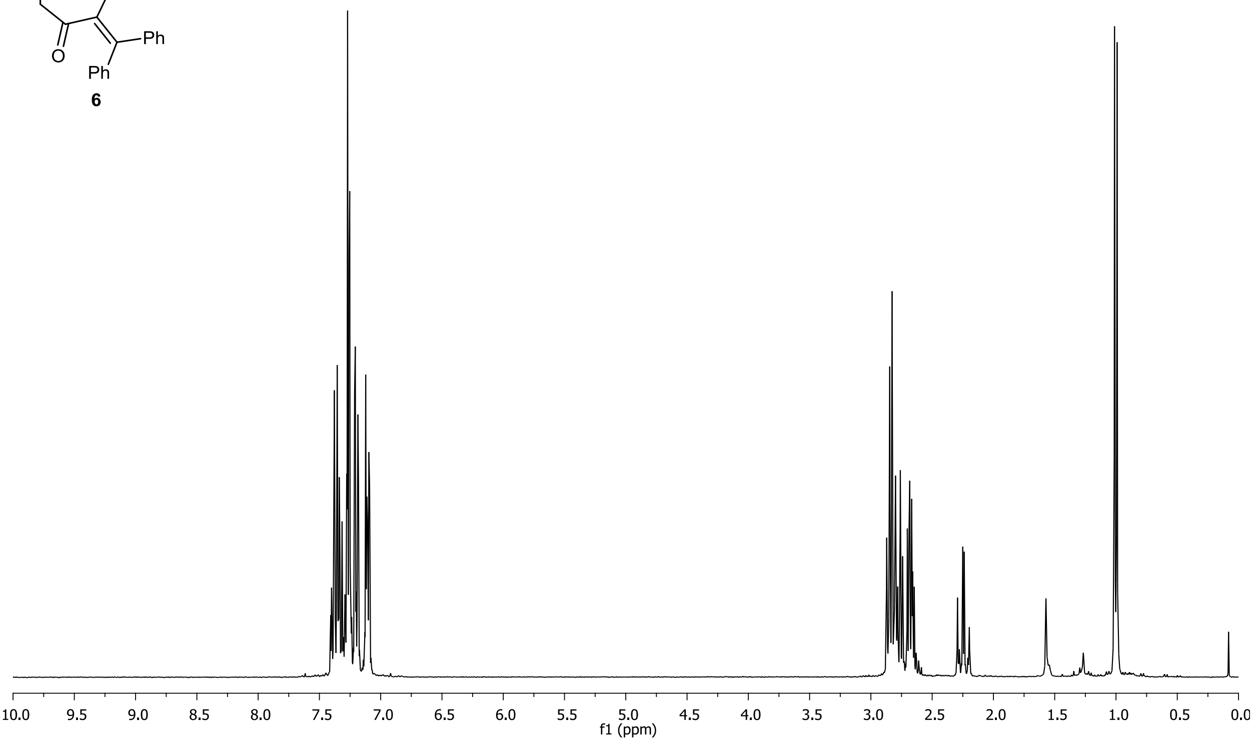
10.0 9.5 9.0 8.5 8.0 7.5 7.0 6.5 6.0 5.5 5.0 4.5 4.0 3.5 3.0 2.5 2.0 1.5 1.0 0.5 0.0
f1 (ppm)

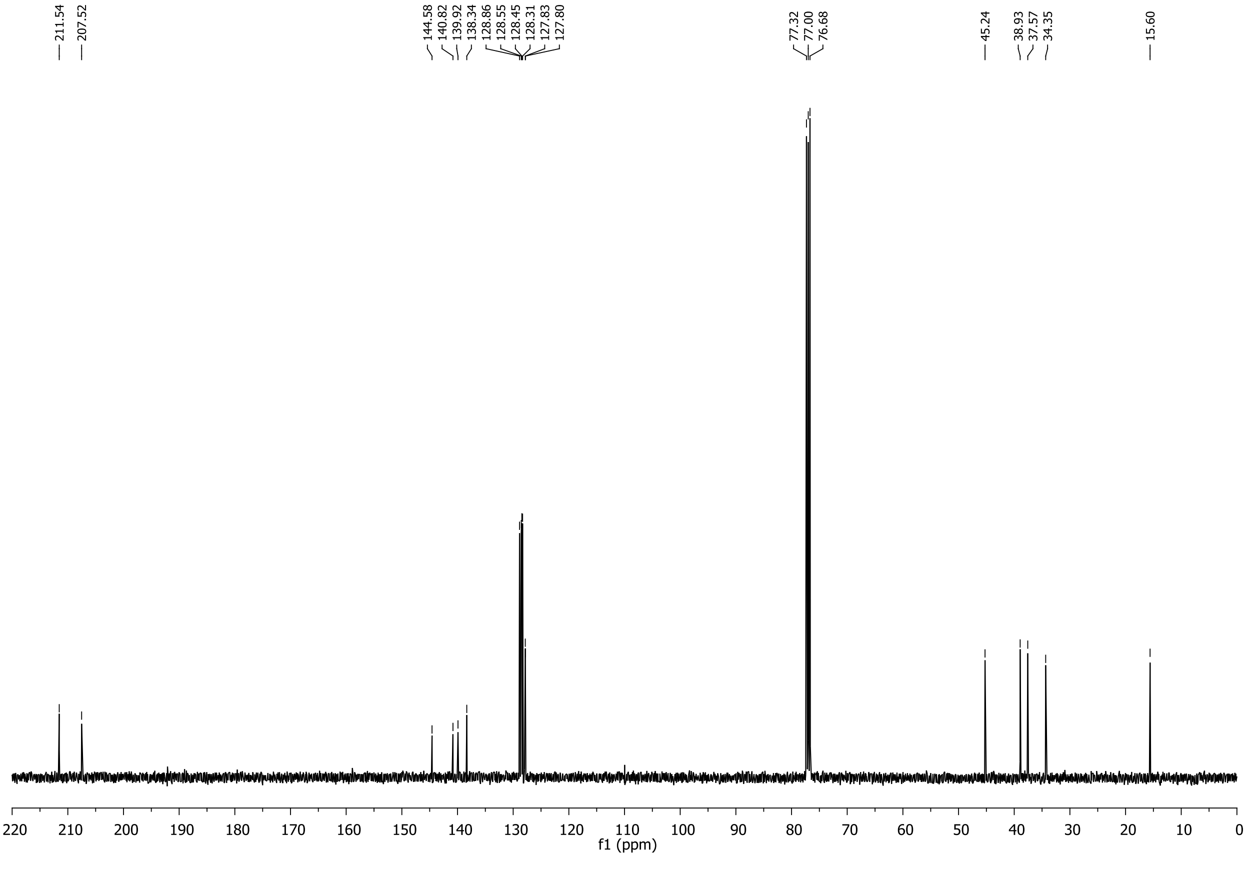
211.54
207.52
144.58
140.82
139.92
138.34
128.86
128.55
128.45
128.31
127.83
127.80
77.32
77.00
76.68
45.24
38.93
37.57
34.35
15.60
220 210 200 190 180 170 160 150 140 130 120 110 100 90 80 70 60 50 40 30 20 10 0
f1 (ppm)

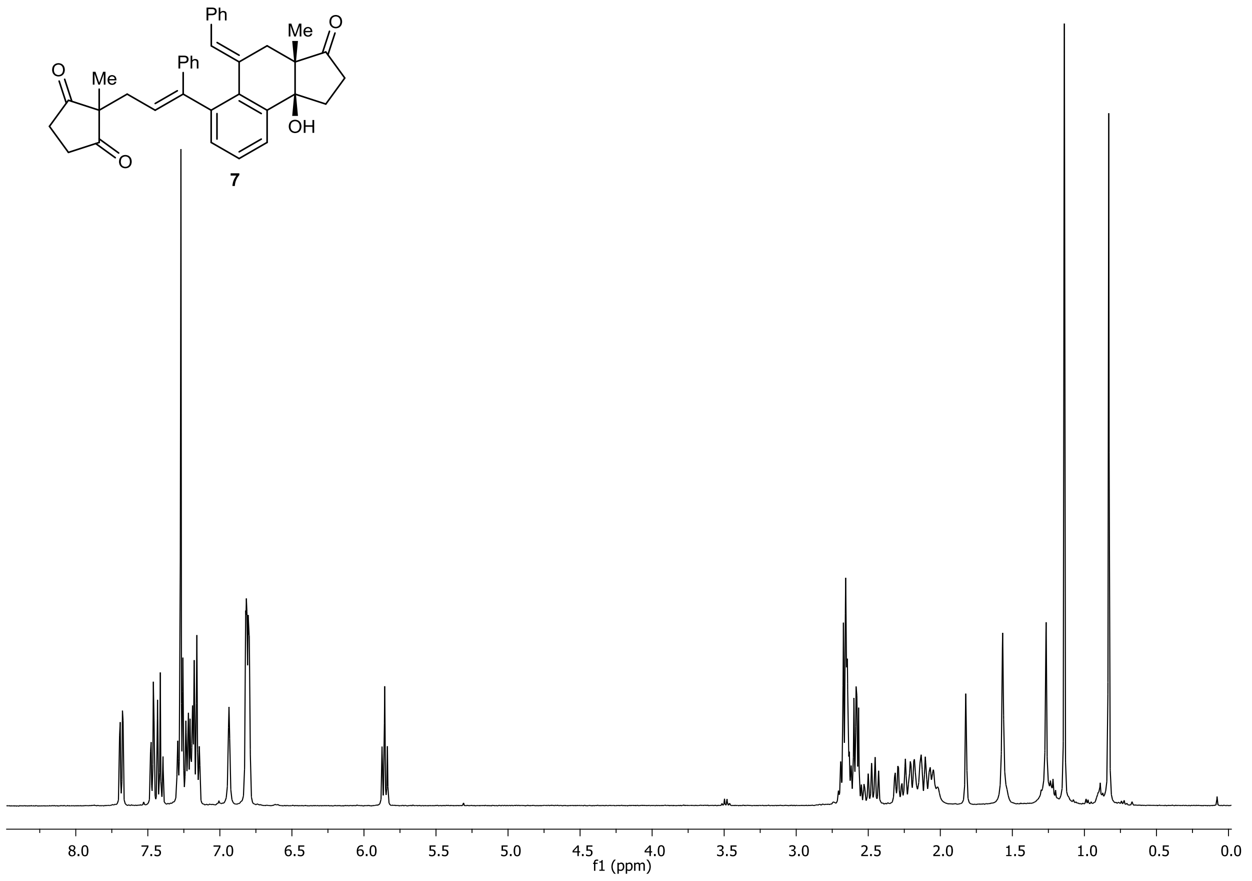
Ph
Me
O
O
Me
Ph
OH
O
7
8.0 7.5 7.0 6.5 6.0 5.5 5.0 4.5 4.0 3.5 3.0 2.5 2.0 1.5 1.0 0.5 0.0
f1 (ppm)

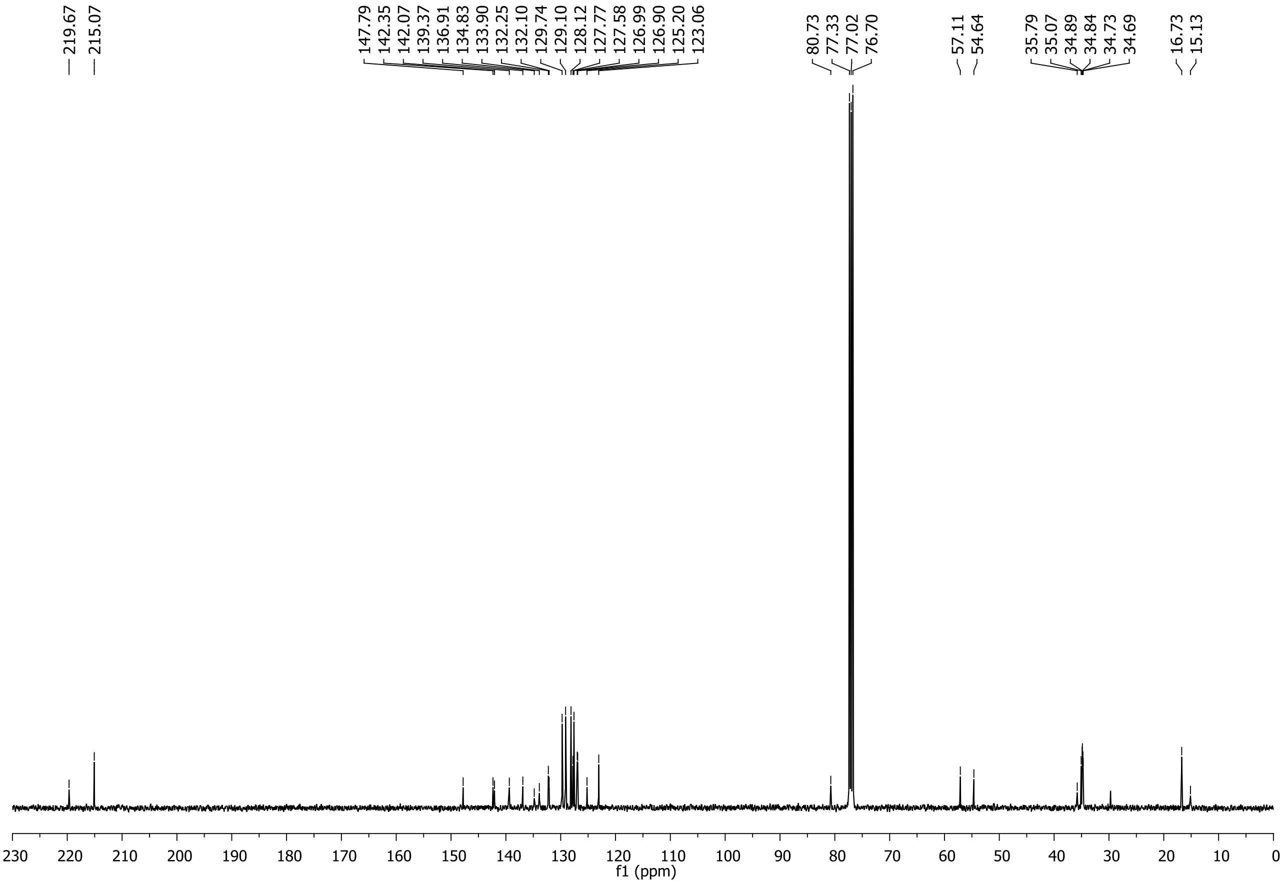
219.67
215.07
147.79
142.35
142.07
139.37
136.91
134.83
133.90
132.25
132.10
129.74
129.10
128.12
127.77
127.58
126.99
126.90
125.20
123.06
80.73
77.33
77.02
76.70
57.11
54.64
35.79
35.07
34.89
34.84
34.73
34.69
16.73
15.13
230 220 210 200 190 180 170 160 150 140 130 120 110 100 90 80 70 60 50 40 30 20 10 0
f1 (ppm)

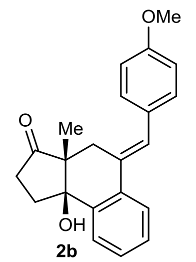

OMe
O
Me
OH


**2b**

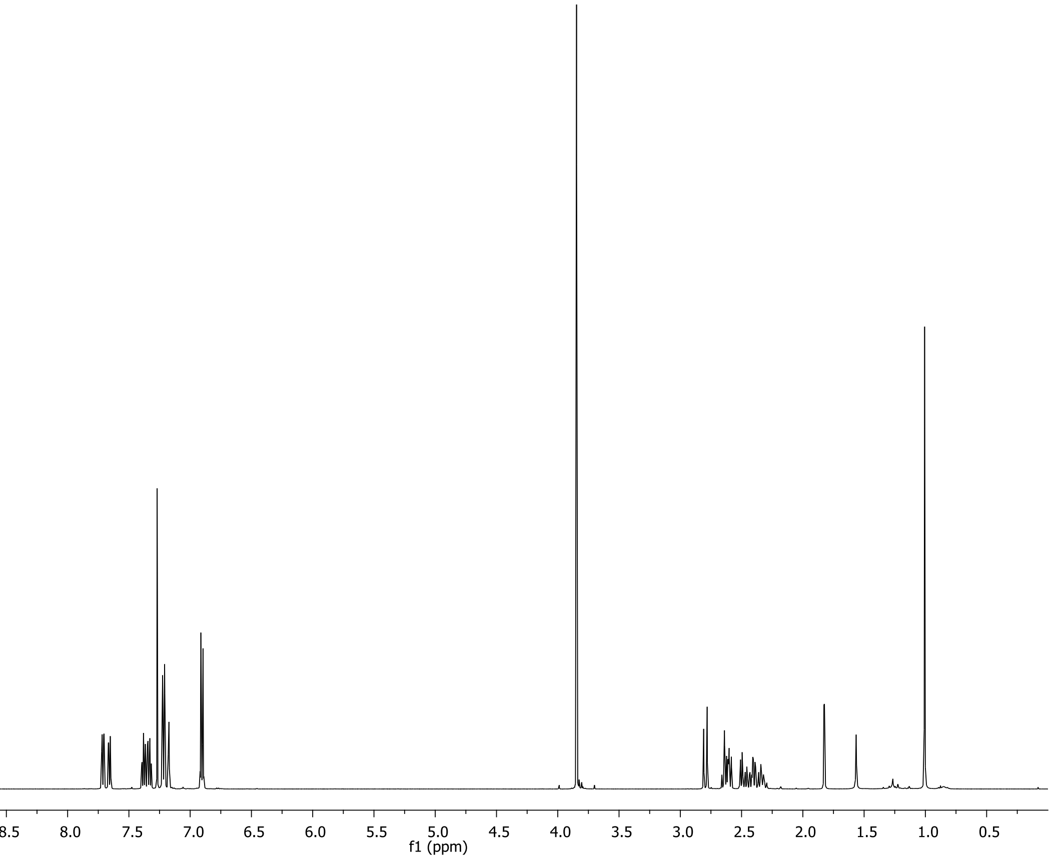

10.0 9.5 9.0 8.5 8.0 7.5 7.0 6.5 6.0 5.5 5.0 4.5 4.0 3.5 3.0 2.5 2.0 1.5 1.0 0.5
f1 (ppm)


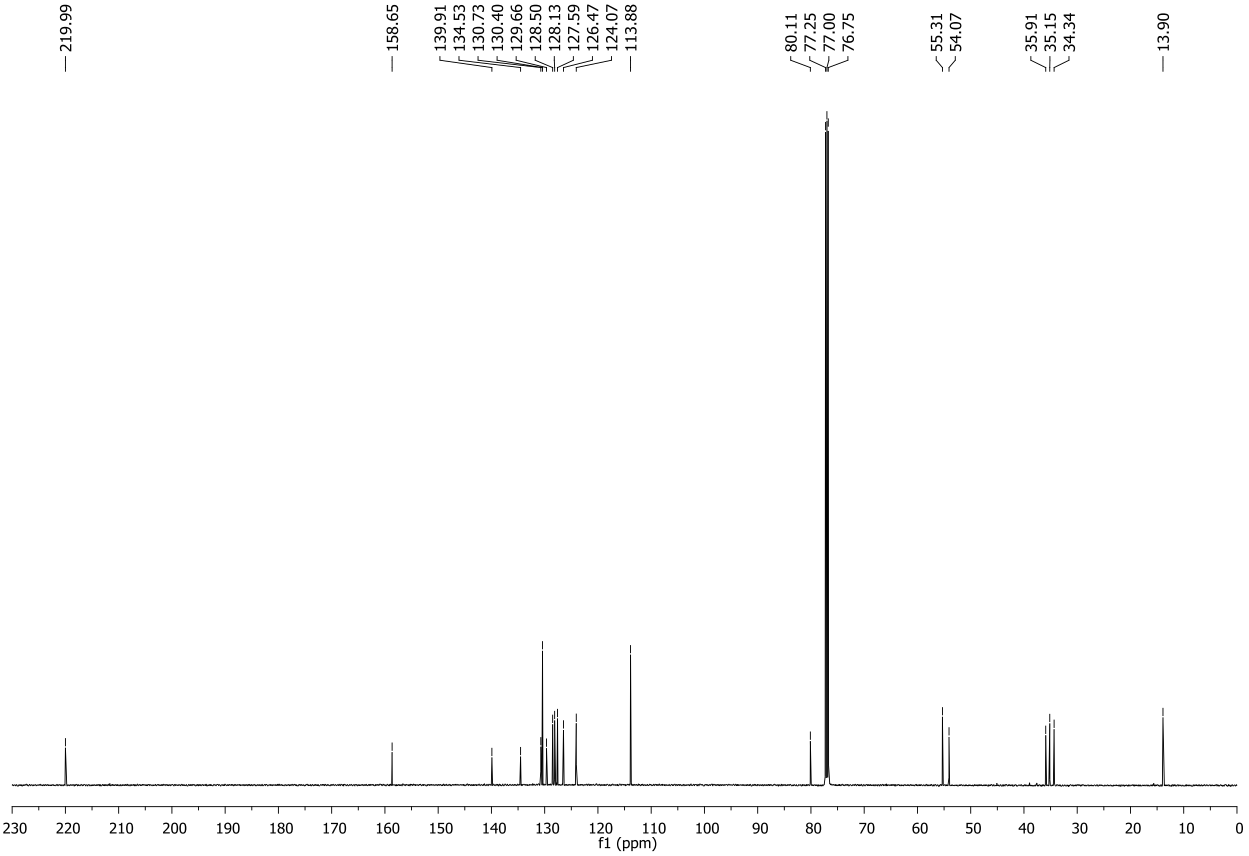

219.99
158.65
139.91
134.53
130.73
130.40
129.66
128.50
128.13
127.59
126.47
124.07
113.88
80.11
77.25
77.00
76.75
55.31
54.07
35.91
35.15
34.34
13.90
230 220 210 200 190 180 170 160 150 140 130 120 110 100 90 80 70 60 50 40 30 20 10 0
f1 (ppm)

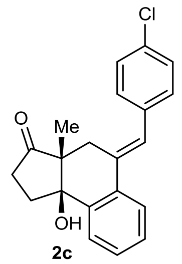
Cl
O
Me
OH

**2c**

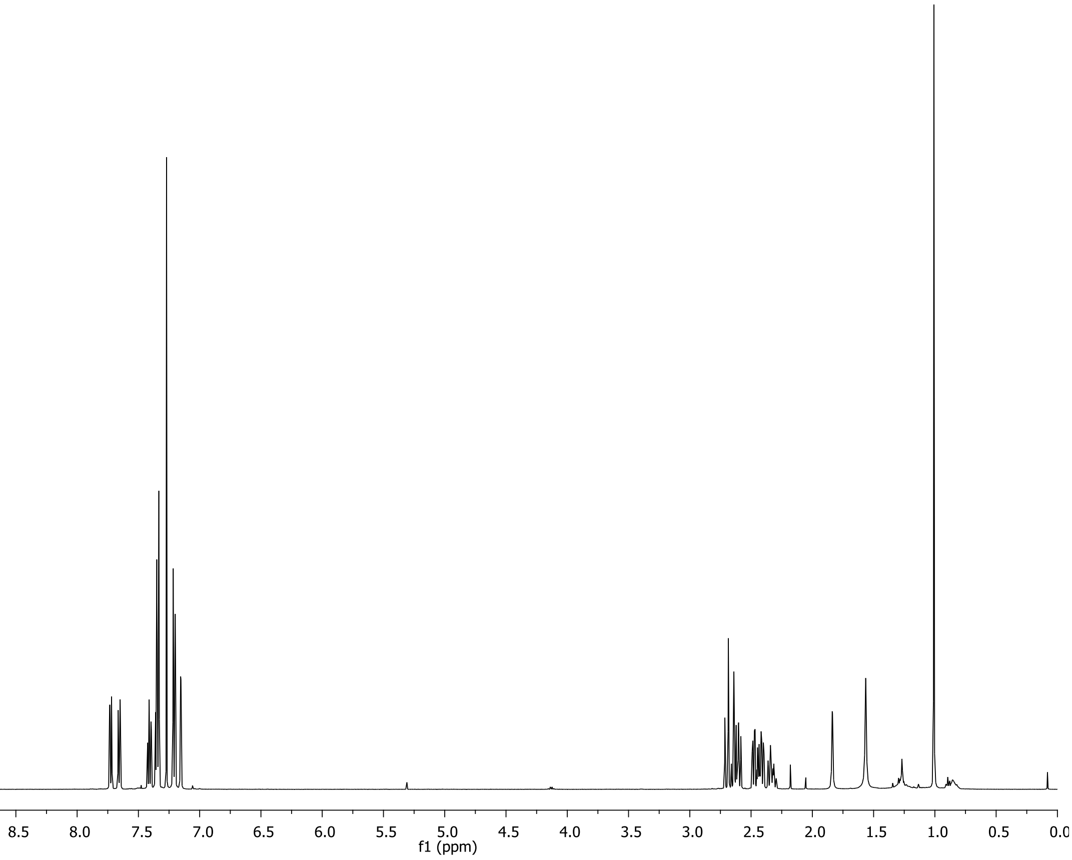
10.0 9.5 9.0 8.5 8.0 7.5 7.0 6.5 6.0 5.5 5.0 4.5 4.0 3.5 3.0 2.5 2.0 1.5 1.0 0.5 0.0
f1 (ppm)

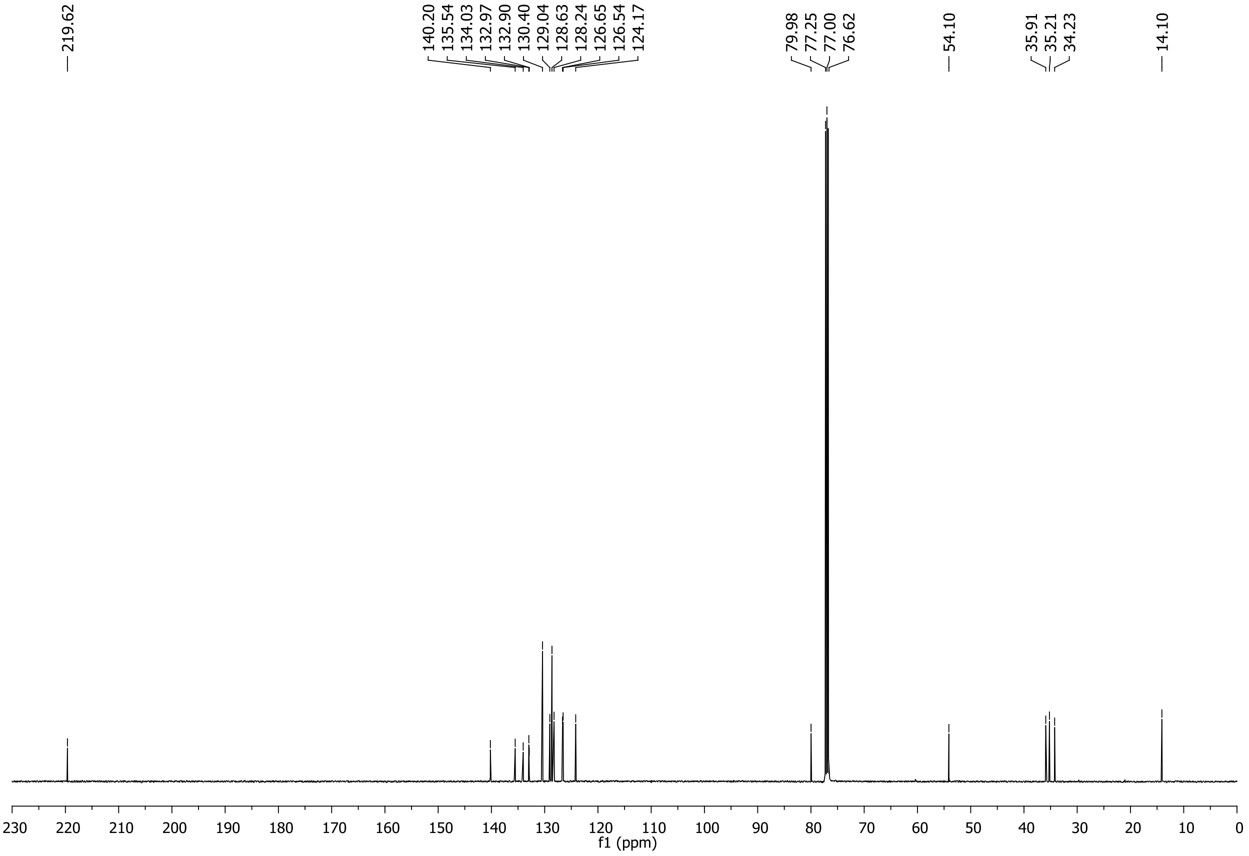
219.62
140.20
135.54
134.03
132.97
132.90
130.40
129.04
128.63
128.24
126.65
126.54
124.17
79.98
77.25
77.00
76.62
54.10
35.91
35.21
34.23
14.10
230 220 210 200 190 180 170 160 150 140 130 120 110 100 90 80 70 60 50 40 30 20 10 0
f1 (ppm)

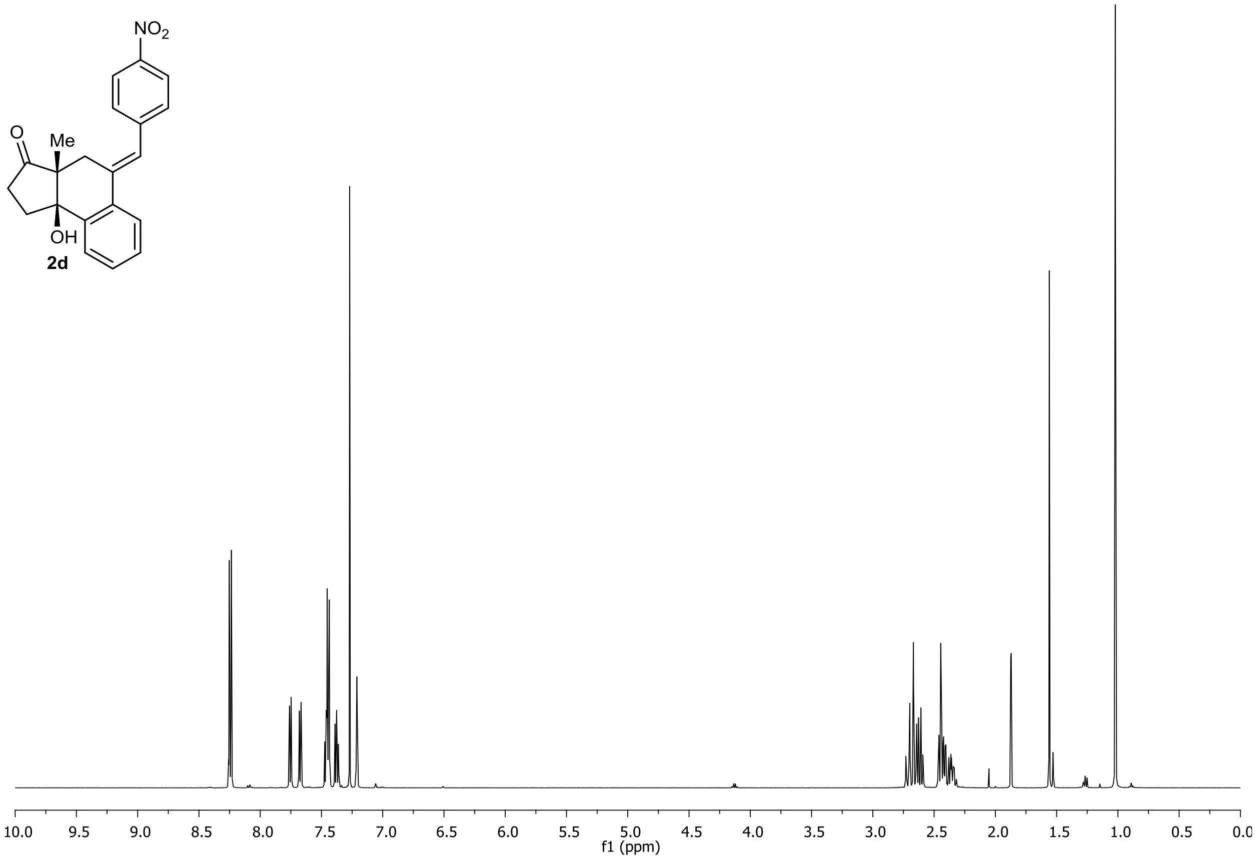
NO2
O
Me
OH
2d
10.0 9.5 9.0 8.5 8.0 7.5 7.0 6.5 6.0 5.5 5.0 4.5 4.0 3.5 3.0 2.5 2.0 1.5 1.0 0.5 0.0
f1 (ppm)

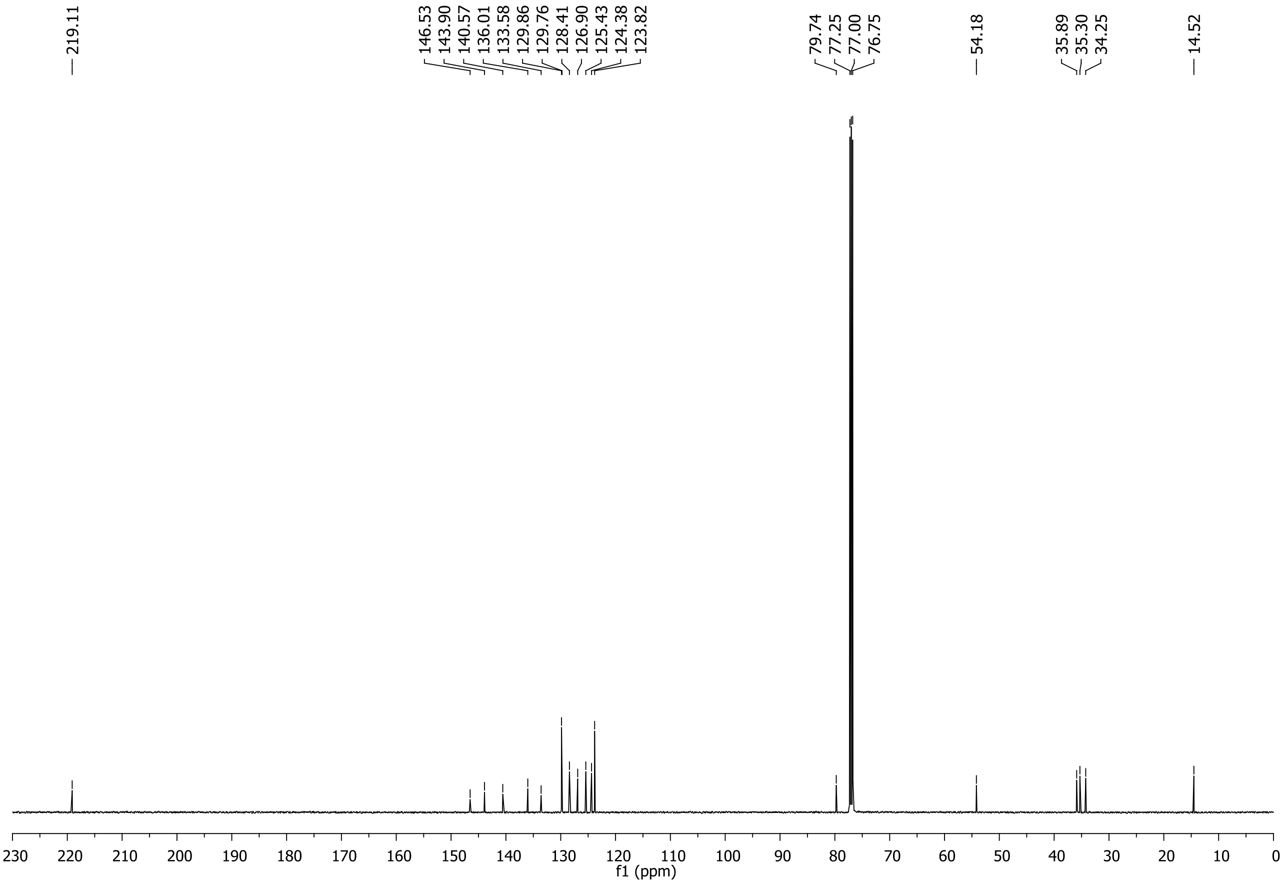
219.11
146.53
143.90
140.57
136.01
133.58
129.86
129.76
128.41
126.90
125.43
124.38
123.82
79.74
77.25
77.00
76.75
54.18
35.89
35.30
34.25
14.52
230 220 210 200 190 180 170 160 150 140 130 120 110 100 90 80 70 60 50 40 30 20 10 0
f1 (ppm)

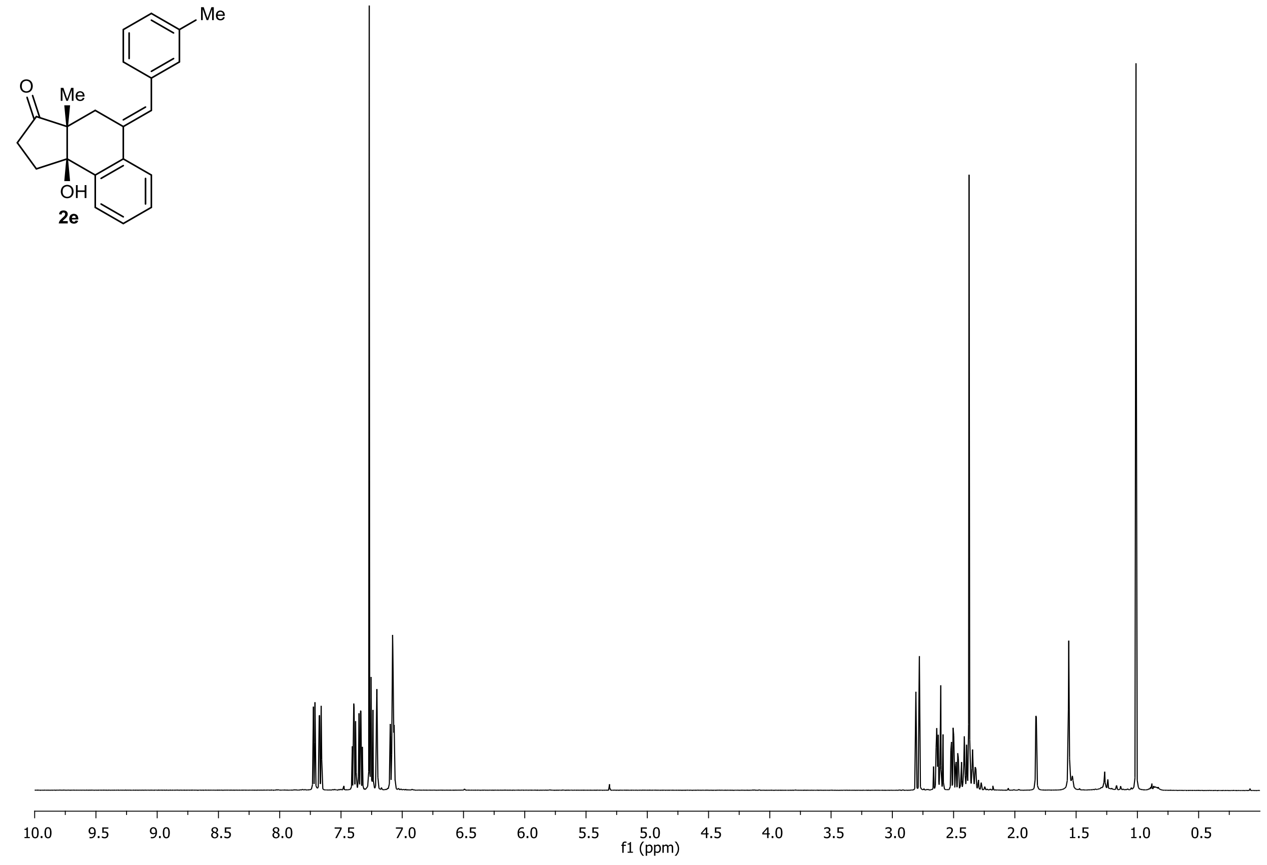
Me
O
Me
OH
2e
10.0 9.5 9.0 8.5 8.0 7.5 7.0 6.5 6.0 5.5 5.0 4.5 4.0 3.5 3.0 2.5 2.0 1.5 1.0 0.5
f1 (ppm)

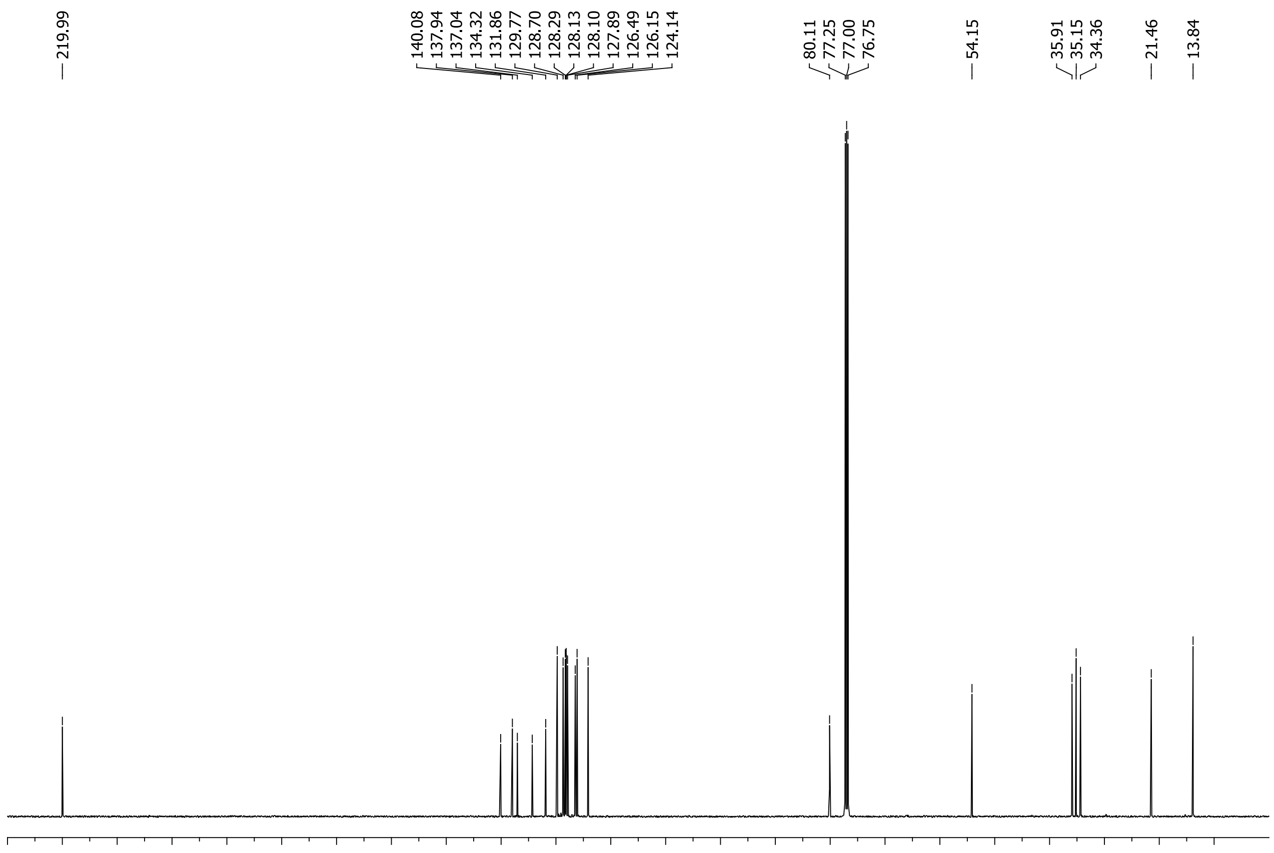
219.99
140.08
137.94
137.04
134.32
131.86
129.77
128.70
128.29
128.13
128.10
127.89
126.49
126.15
124.14
80.11
77.25
77.00
76.75
54.15
35.91
35.15
34.36
21.46
13.84
230 220 210 200 190 180 170 160 150 140 130 120 110 100 90 80 70 60 50 40 30 20 10
f1 (ppm)

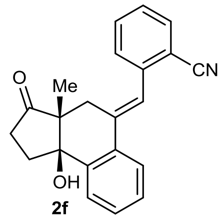
O
Me
CN
OH

**2f**

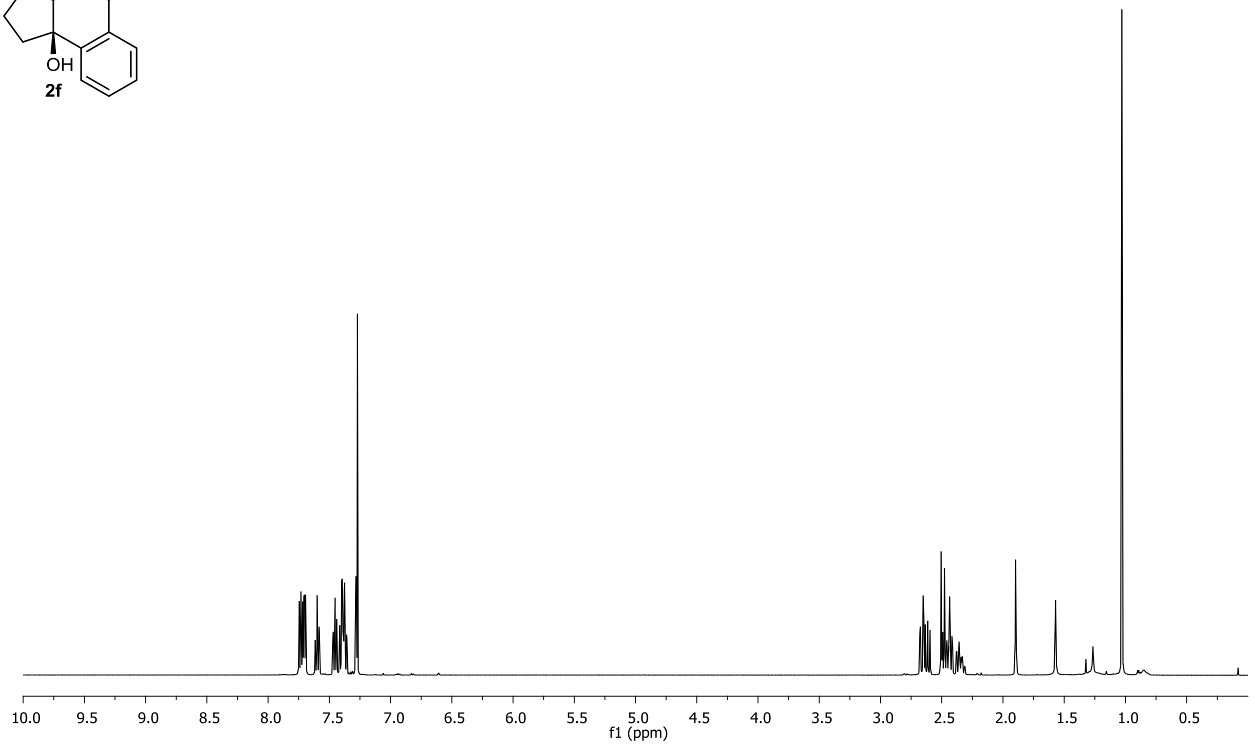
10.0 9.5 9.0 8.5 8.0 7.5 7.0 6.5 6.0 5.5 5.0 4.5 4.0 3.5 3.0 2.5 2.0 1.5 1.0 0.5
f1 (ppm)

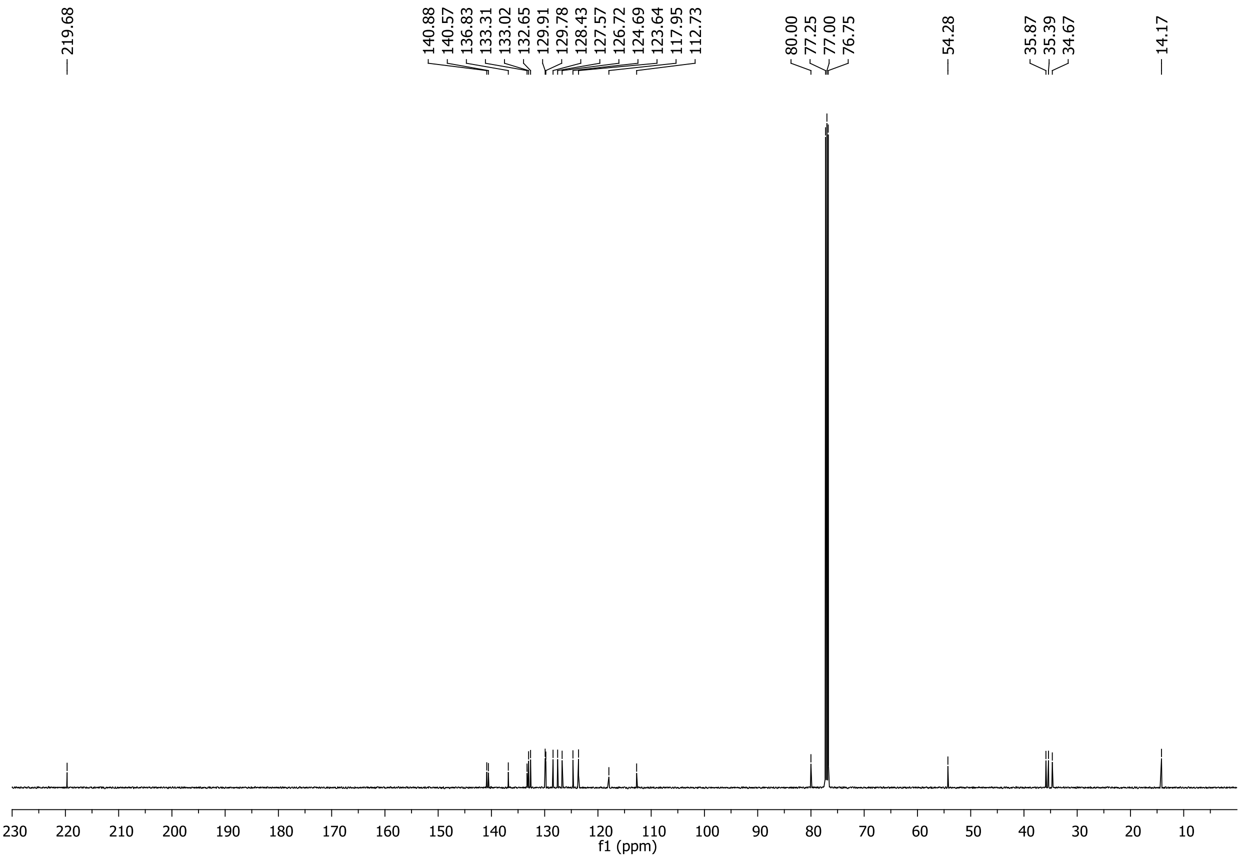
219.68
140.88
140.57
136.83
133.31
133.02
132.65
129.91
129.78
128.43
127.57
126.72
124.69
123.64
117.95
112.73
80.00
77.25
77.00
76.75
54.28
35.87
35.39
34.67
14.17
230 220 210 200 190 180 170 160 150 140 130 120 110 100 90 80 70 60 50 40 30 20 10
f1 (ppm)

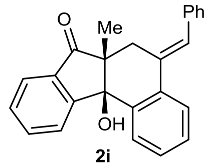
O
Me
Ph
OH

**2i**

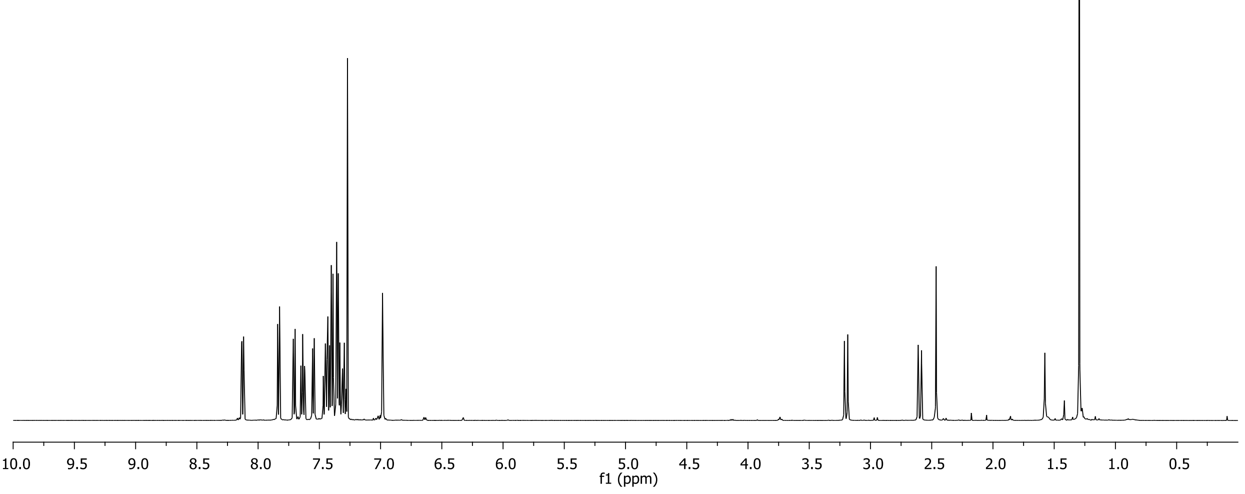
10.0 9.5 9.0 8.5 8.0 7.5 7.0 6.5 6.0 5.5 5.0 4.5 4.0 3.5 3.0 2.5 2.0 1.5 1.0 0.5
f1 (ppm)

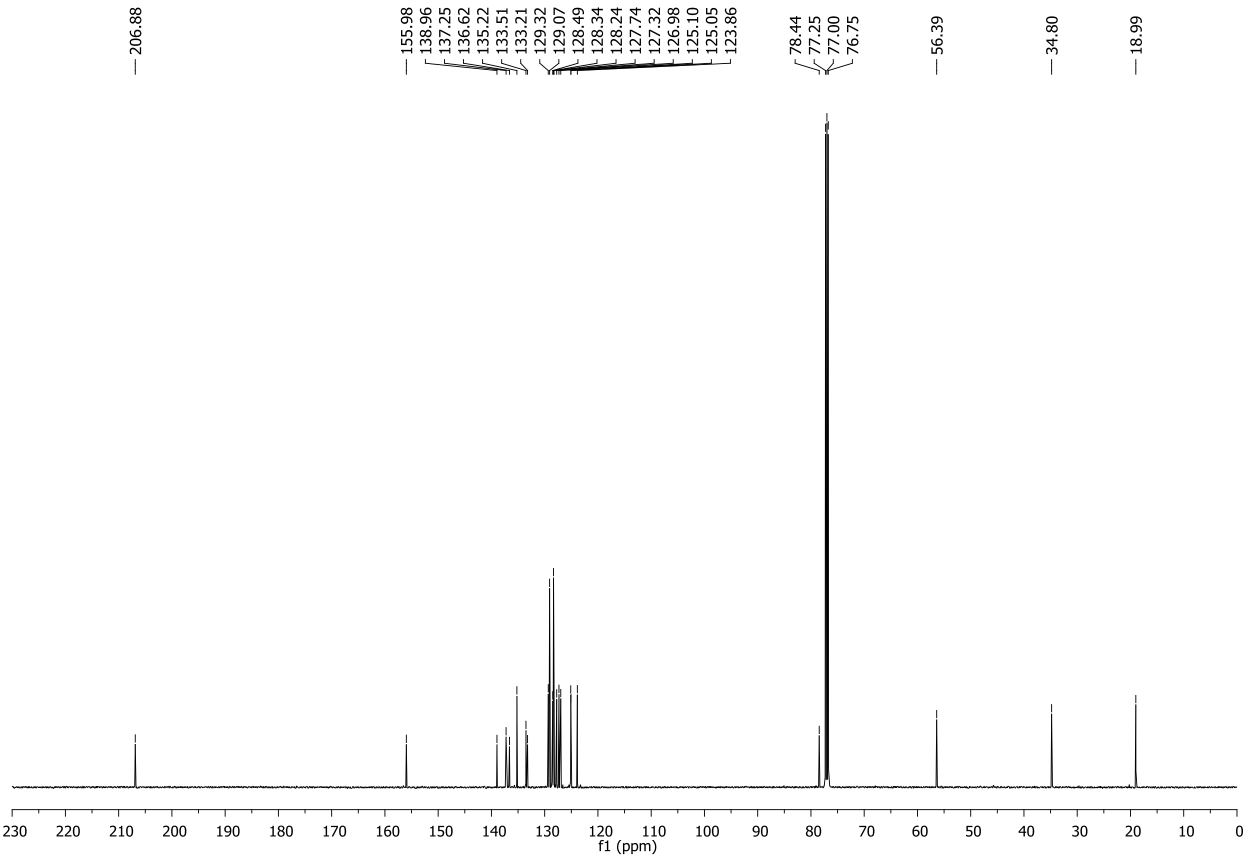
206.88
155.98
138.96
137.25
136.62
135.22
133.51
133.21
129.32
129.07
128.49
128.34
128.24
127.74
127.32
126.98
125.10
125.05
123.86
78.44
77.25
77.00
76.75
56.39
34.80
18.99
230 220 210 200 190 180 170 160 150 140 130 120 110 100 90 80 70 60 50 40 30 20 10 0
f1 (ppm)

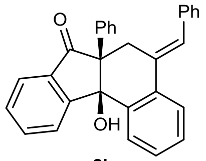
O
Ph
Ph
OH

**2j**

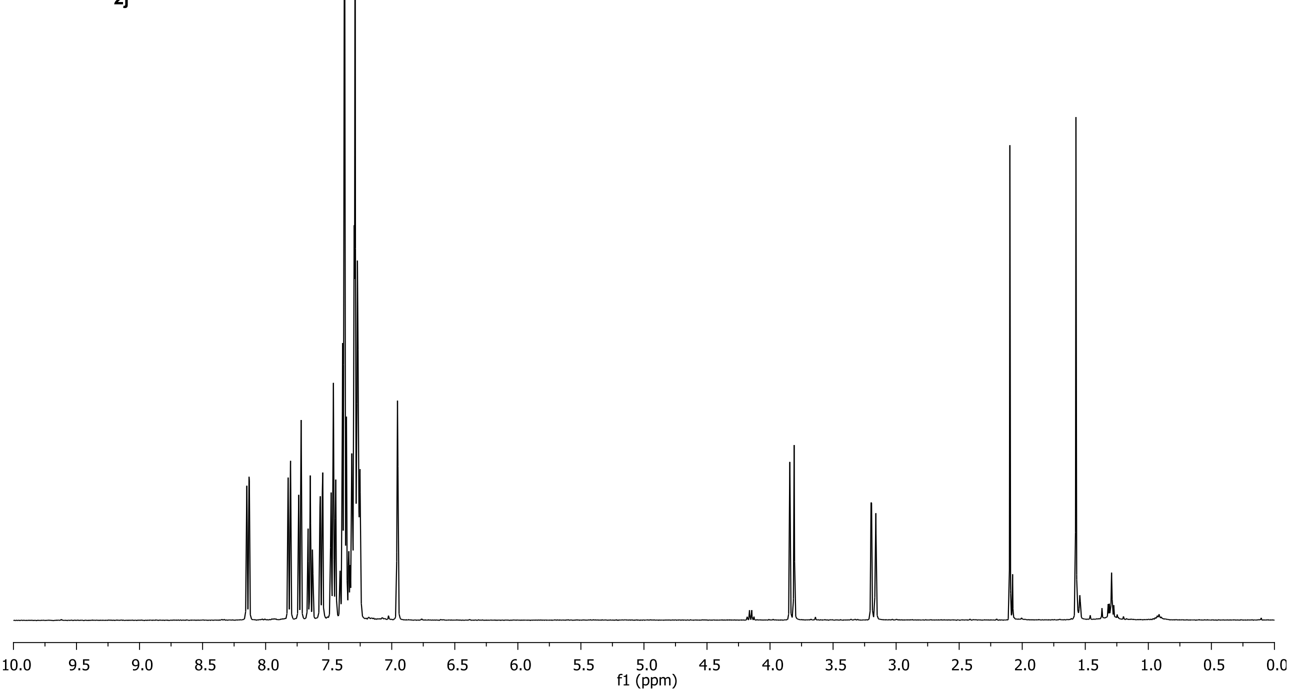
f1 (ppm)

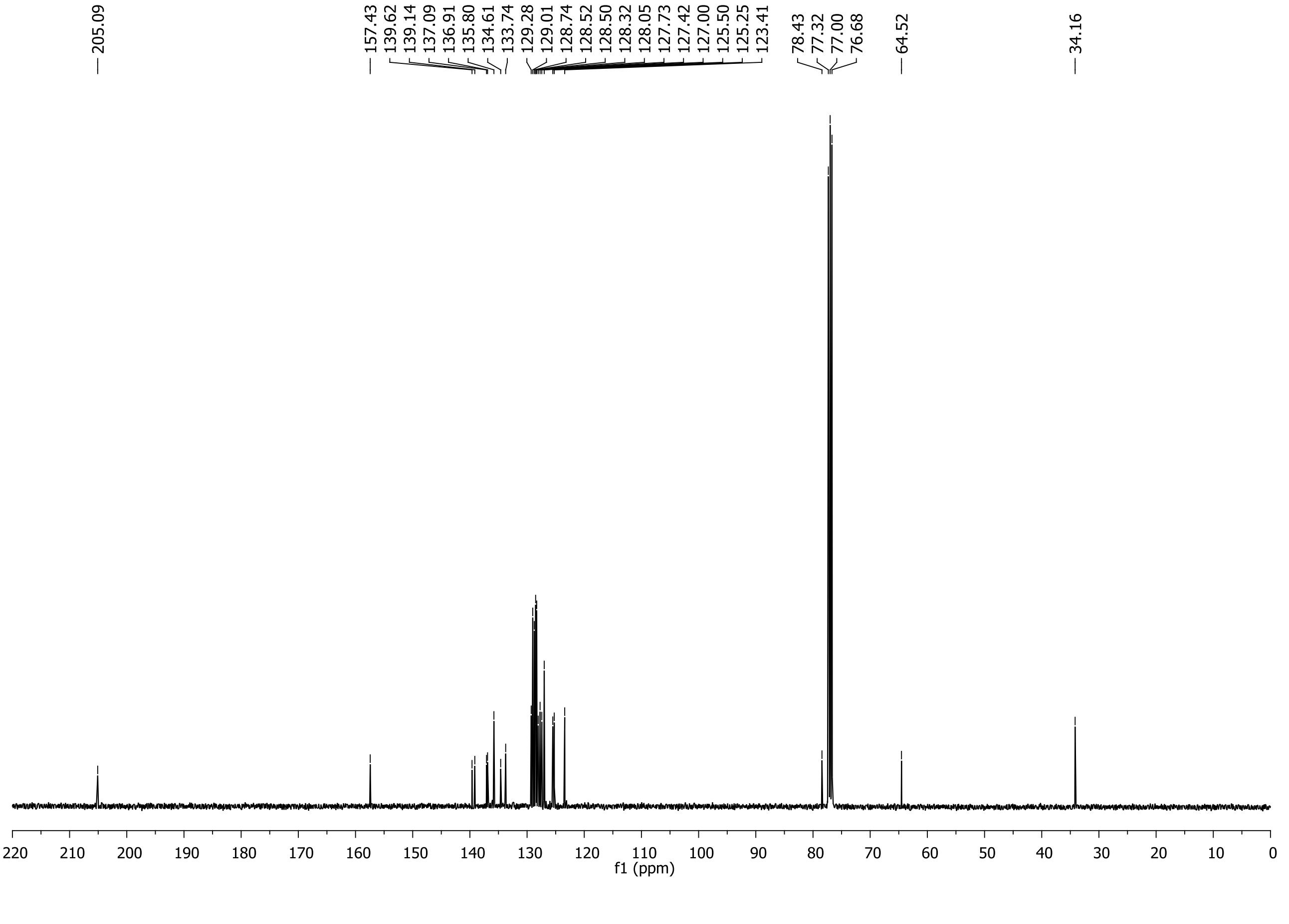
205.09
157.43
139.62
139.14
137.09
136.91
135.80
134.61
133.74
129.28
129.01
128.74
128.52
128.50
128.32
128.05
127.73
127.42
127.00
125.50
125.25
123.41
78.43
77.32
77.00
76.68
64.52
34.16
f1 (ppm)

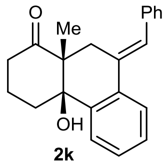
O
Me
Ph
OH

**2k**

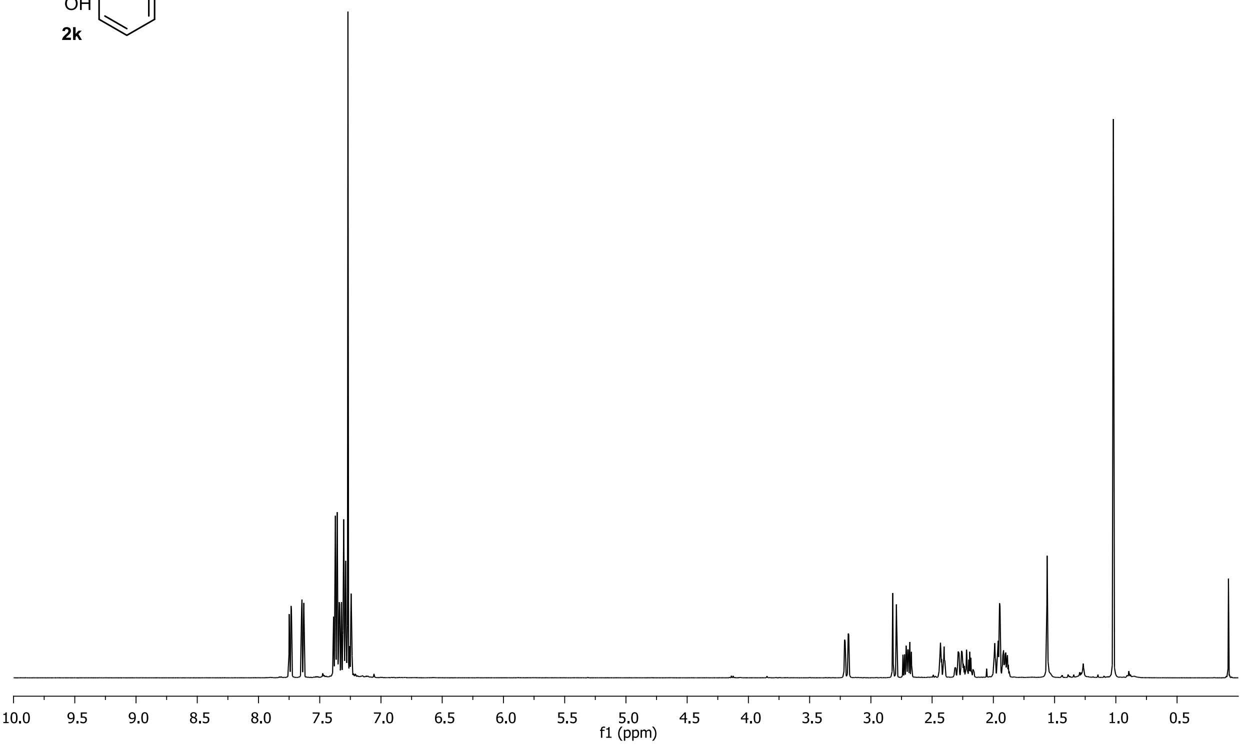
10.0 9.5 9.0 8.5 8.0 7.5 7.0 6.5 6.0 5.5 5.0 4.5 4.0 3.5 3.0 2.5 2.0 1.5 1.0 0.5
f1 (ppm)

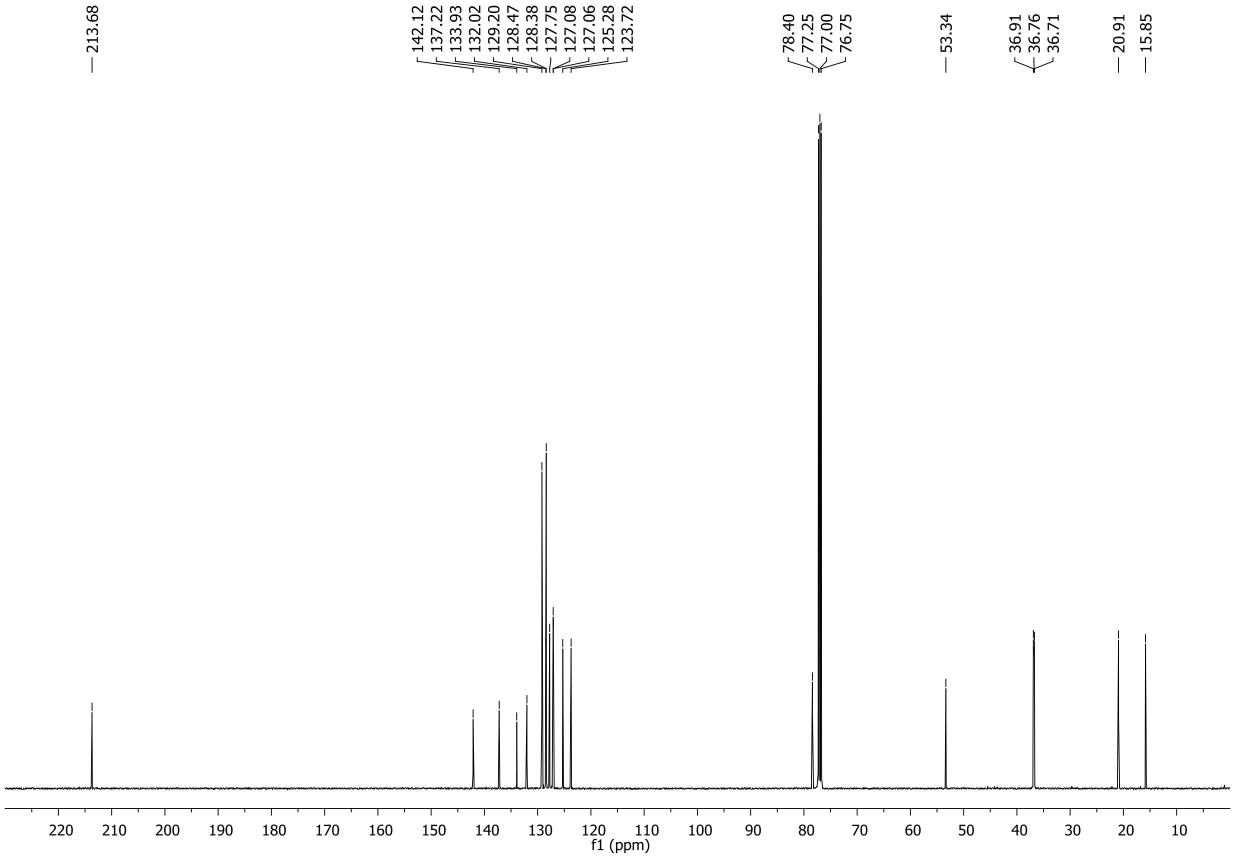
213.68
142.12
137.22
133.93
132.02
129.20
128.47
128.38
127.75
127.08
127.06
125.28
123.72
78.40
77.25
77.00
76.75
53.34
36.91
36.76
36.71
20.91
15.85
220 210 200 190 180 170 160 150 140 130 120 110 100 90 80 70 60 50 40 30 20 10
f1 (ppm)

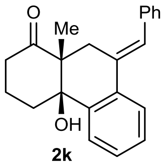
O
Me
Ph
OH

**2k**

HMBC

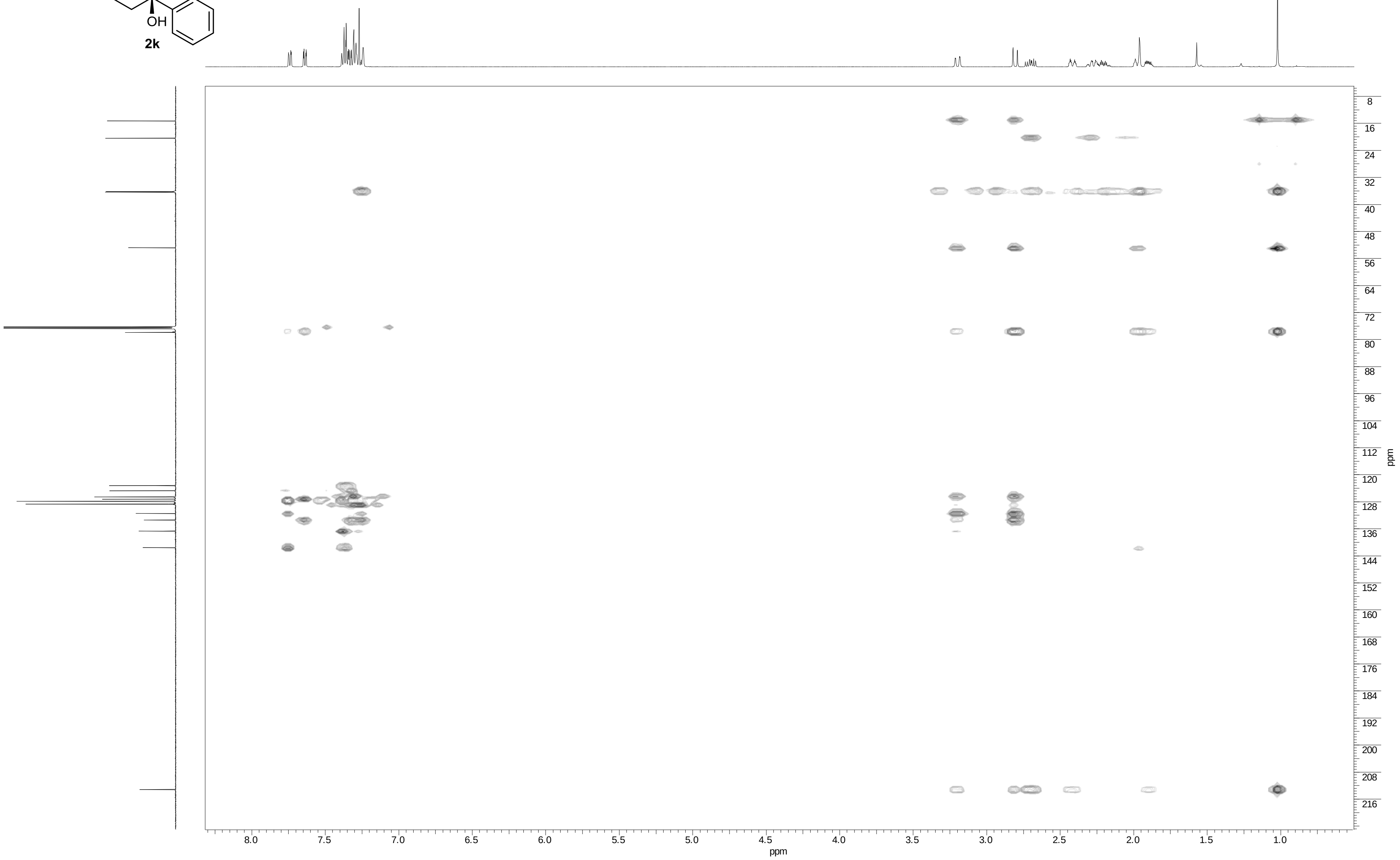
8.0
7.5
7.0
6.5
6.0
5.5
5.0
4.5
4.0
3.5
3.0
2.5
2.0
1.5
1.0
ppm
8
16
24
32
40
48
56
64
72
80
88
96
104
112
120
128
136
144
152
160
168
176
184
192
200
208
216
ppm

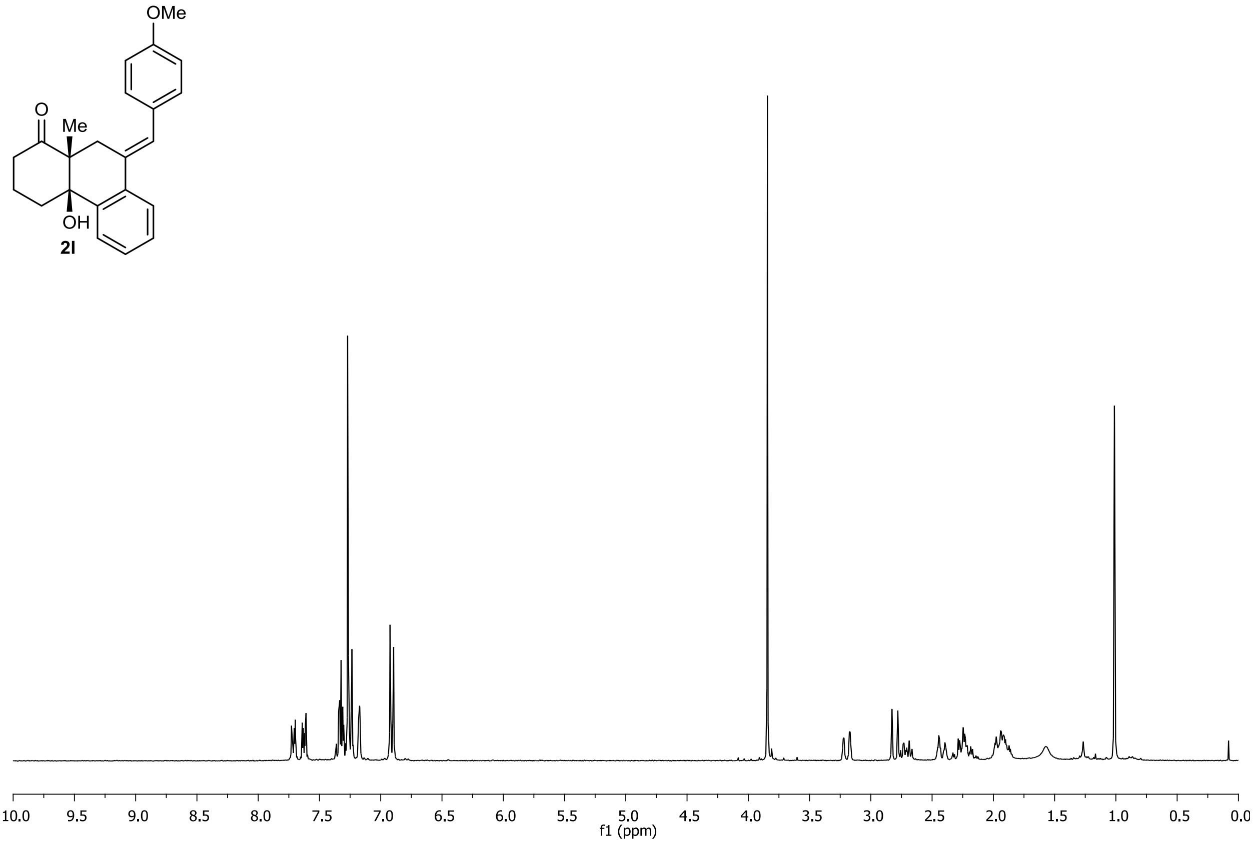
OMe
O
Me
OH
2l
10.0 9.5 9.0 8.5 8.0 7.5 7.0 6.5 6.0 5.5 5.0 4.5 4.0 3.5 3.0 2.5 2.0 1.5 1.0 0.5 0.0
f1 (ppm)

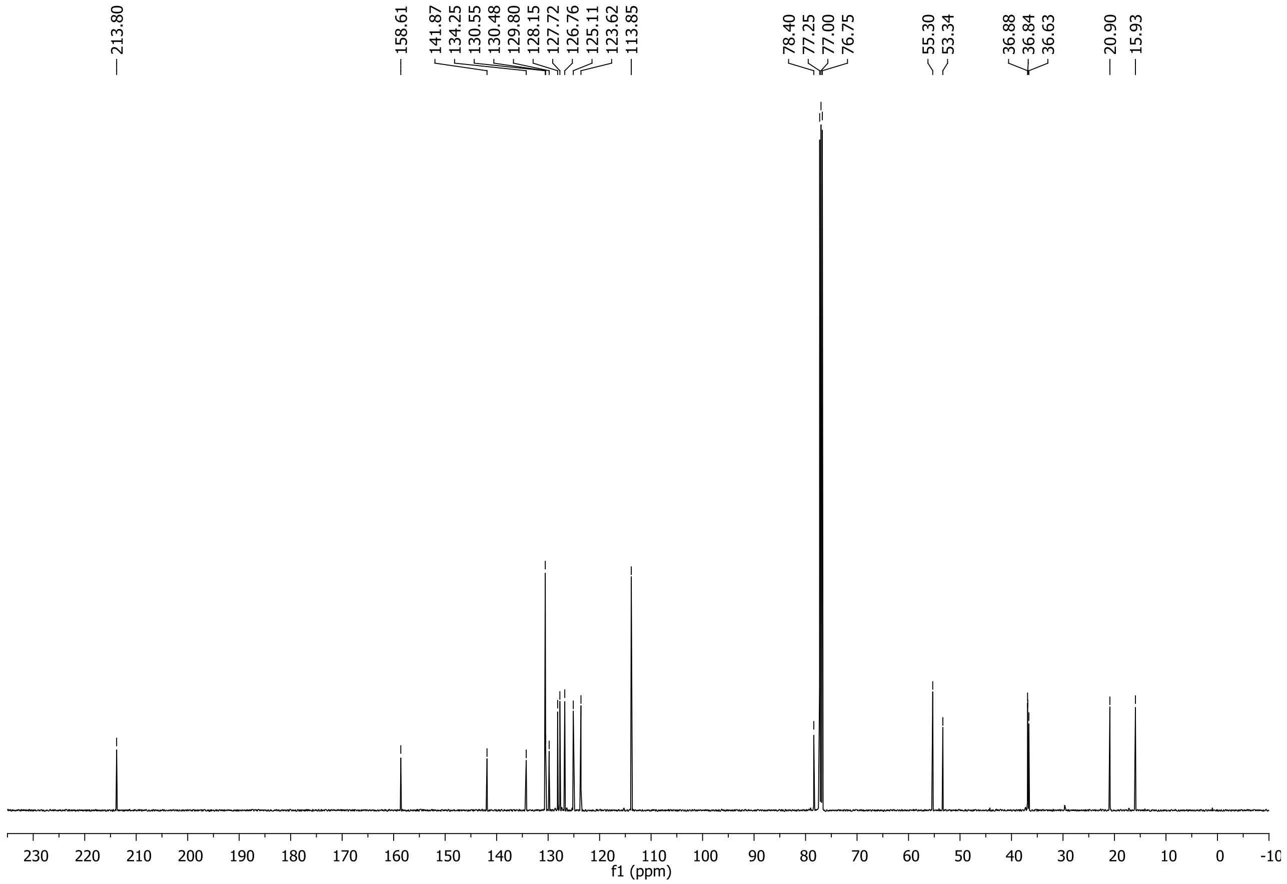
213.80
158.61
141.87
134.25
130.55
130.48
129.80
128.15
127.72
126.76
125.11
123.62
113.85
78.40
77.25
77.00
76.75
55.30
53.34
36.88
36.84
36.63
20.90
15.93
230 220 210 200 190 180 170 160 150 140 130 120 110 100 90 80 70 60 50 40 30 20 10 0 -10
f1 (ppm)

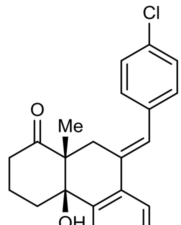
Cl
O
Me
OH

**2m**

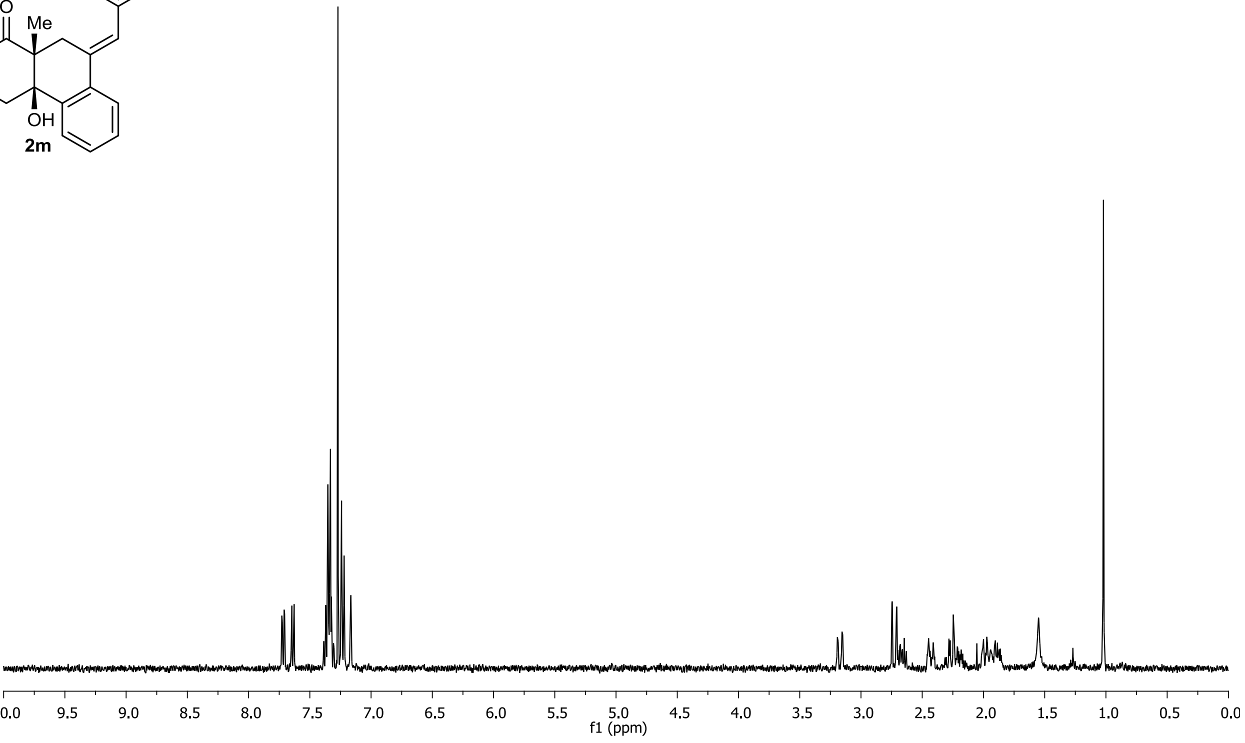
10.0 9.5 9.0 8.5 8.0 7.5 7.0 6.5 6.0 5.5 5.0 4.5 4.0 3.5 3.0 2.5 2.0 1.5 1.0 0.5 0.0
f1 (ppm)

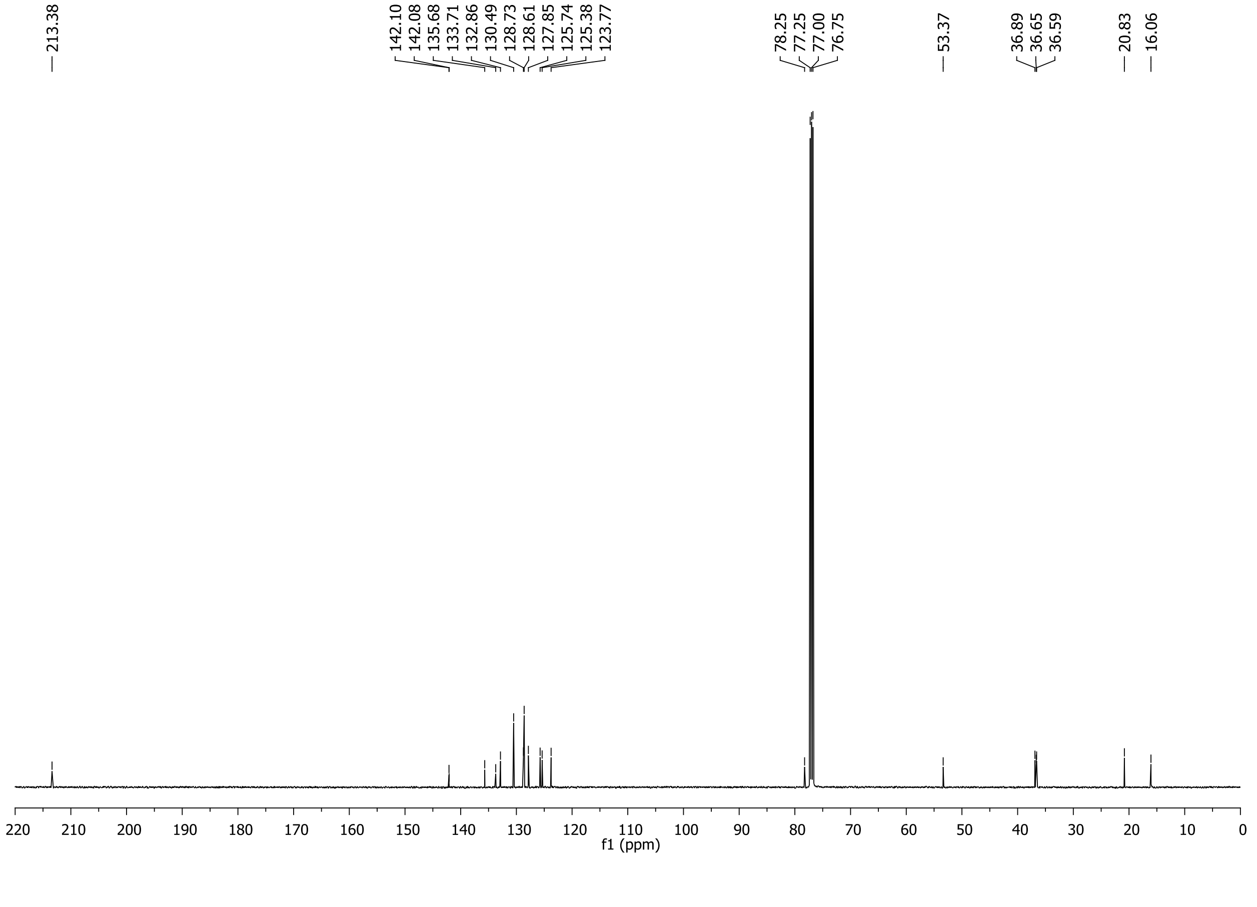
213.38
142.10
142.08
135.68
133.71
132.86
130.49
128.73
128.61
127.85
125.74
125.38
123.77
78.25
77.25
77.00
76.75
53.37
36.89
36.65
36.59
20.83
16.06
220 210 200 190 180 170 160 150 140 130 120 110 100 90 80 70 60 50 40 30 20 10 0
f1 (ppm)

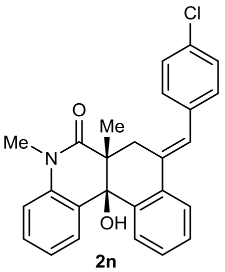
Cl
O
Me
Me
N
OH

**2n**

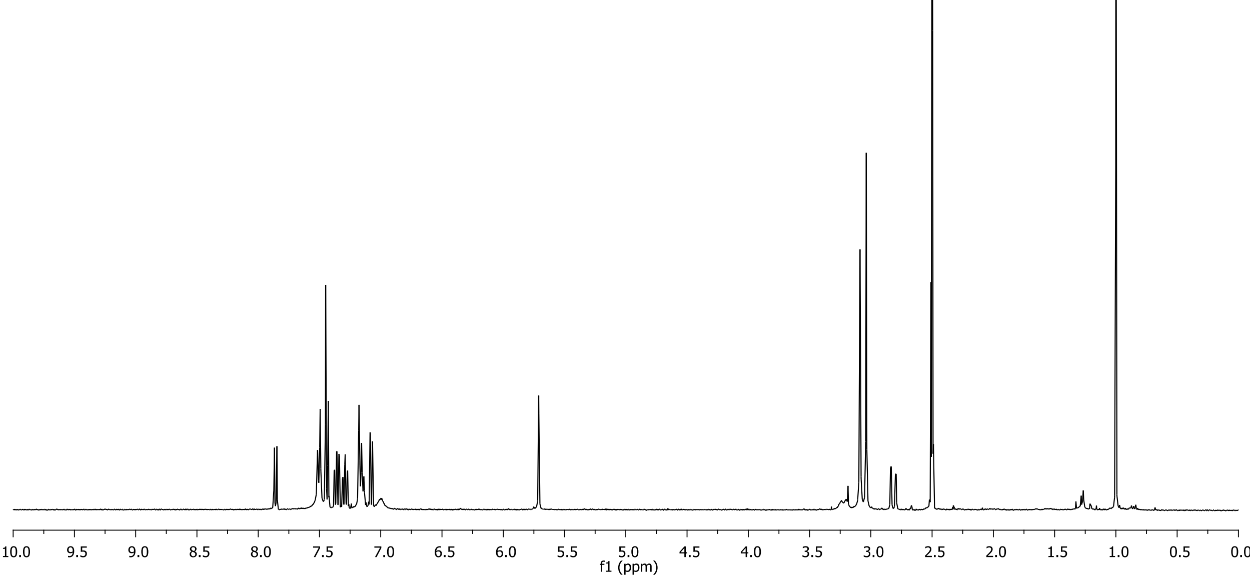
10.0 9.5 9.0 8.5 8.0 7.5 7.0 6.5 6.0 5.5 5.0 4.5 4.0 3.5 3.0 2.5 2.0 1.5 1.0 0.5 0.0
f1 (ppm)

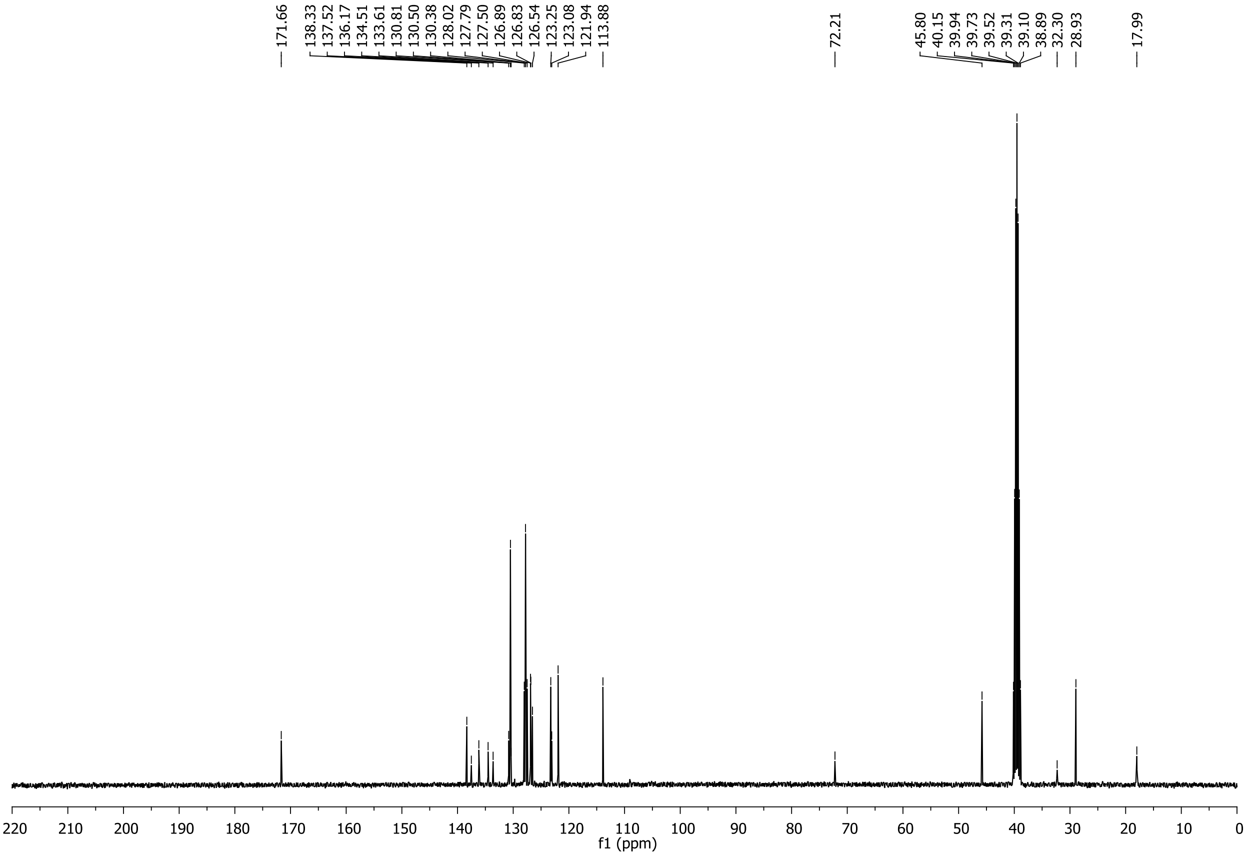
171.66
138.33
137.52
136.17
134.51
133.61
130.81
130.50
130.38
128.02
127.79
127.50
126.89
126.83
126.54
123.25
123.08
121.94
113.88
72.21
45.80
40.15
39.94
39.73
39.52
39.31
39.10
38.89
32.30
28.93
17.99
220 210 200 190 180 170 160 150 140 130 120 110 100 90 80 70 60 50 40 30 20 10 0
f1 (ppm)

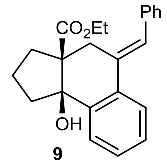
Ph
$CO_2Et$
OH

**9**

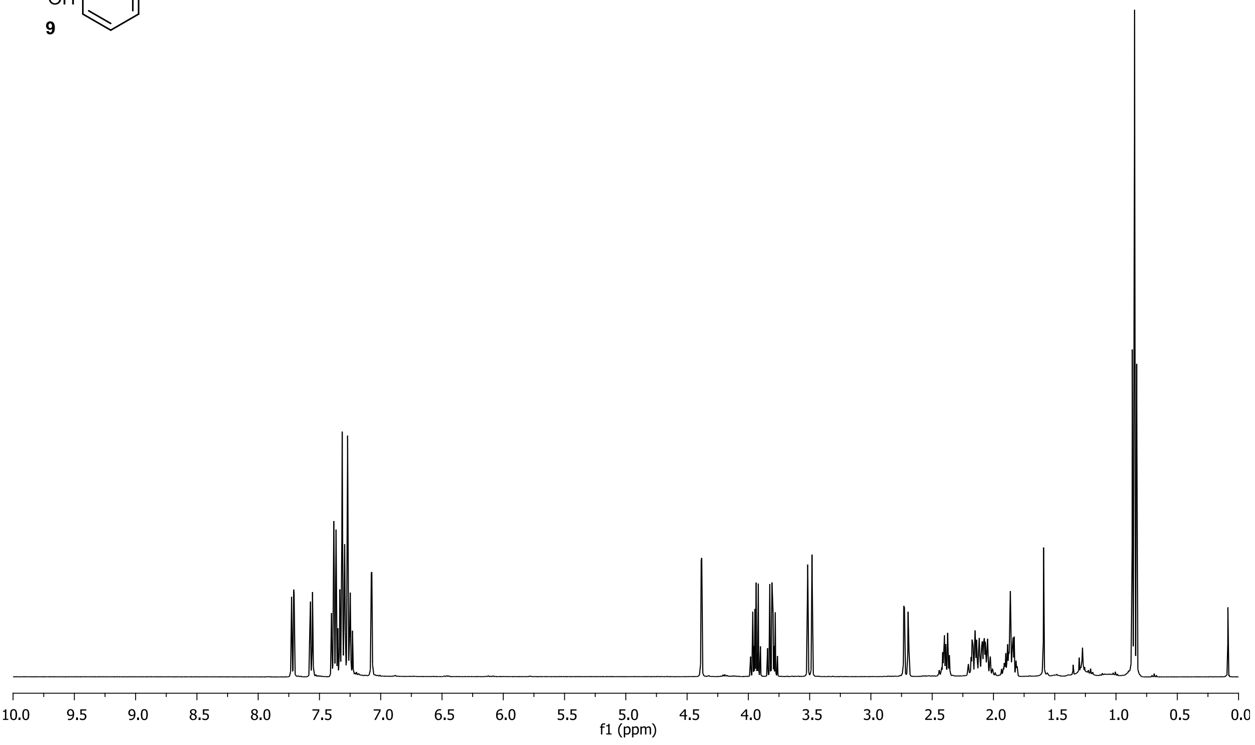
f1 (ppm)

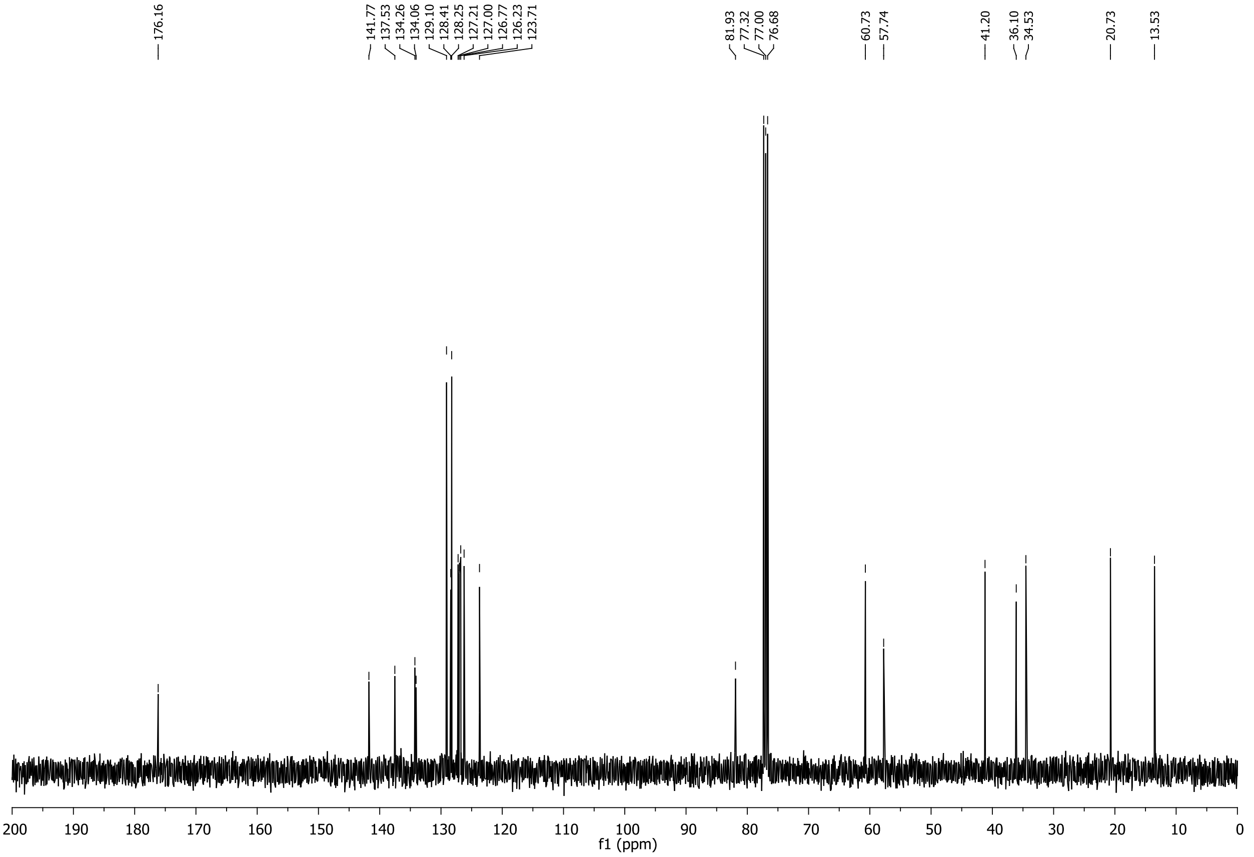
176.16
141.77
137.53
134.26
134.06
129.10
128.41
128.25
127.21
127.00
126.77
126.23
123.71
81.93
77.32
77.00
76.68
60.73
57.74
41.20
36.10
34.53
20.73
13.53
f1 (ppm)

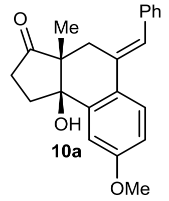
O
Me
Ph
OH
OMe

**10a**

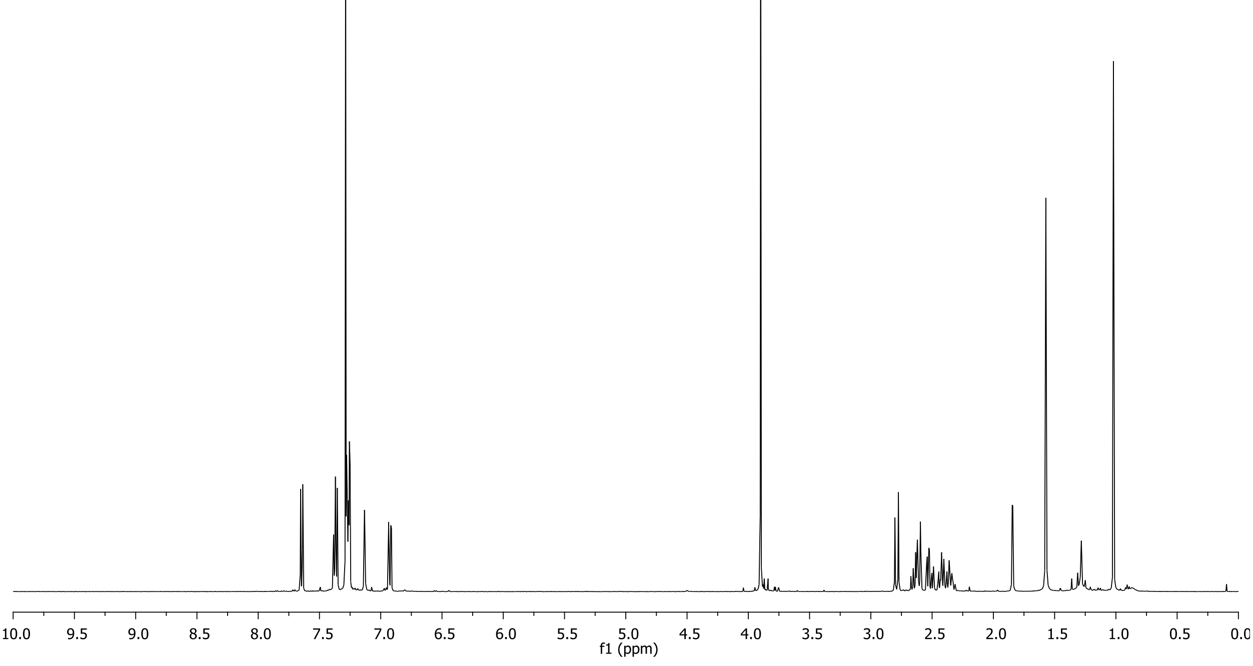
10.0
9.5
9.0
8.5
8.0
7.5
7.0
6.5
6.0
5.5
5.0
4.5
4.0
3.5
3.0
2.5
2.0
1.5
1.0
0.5
0.0
f1 (ppm)

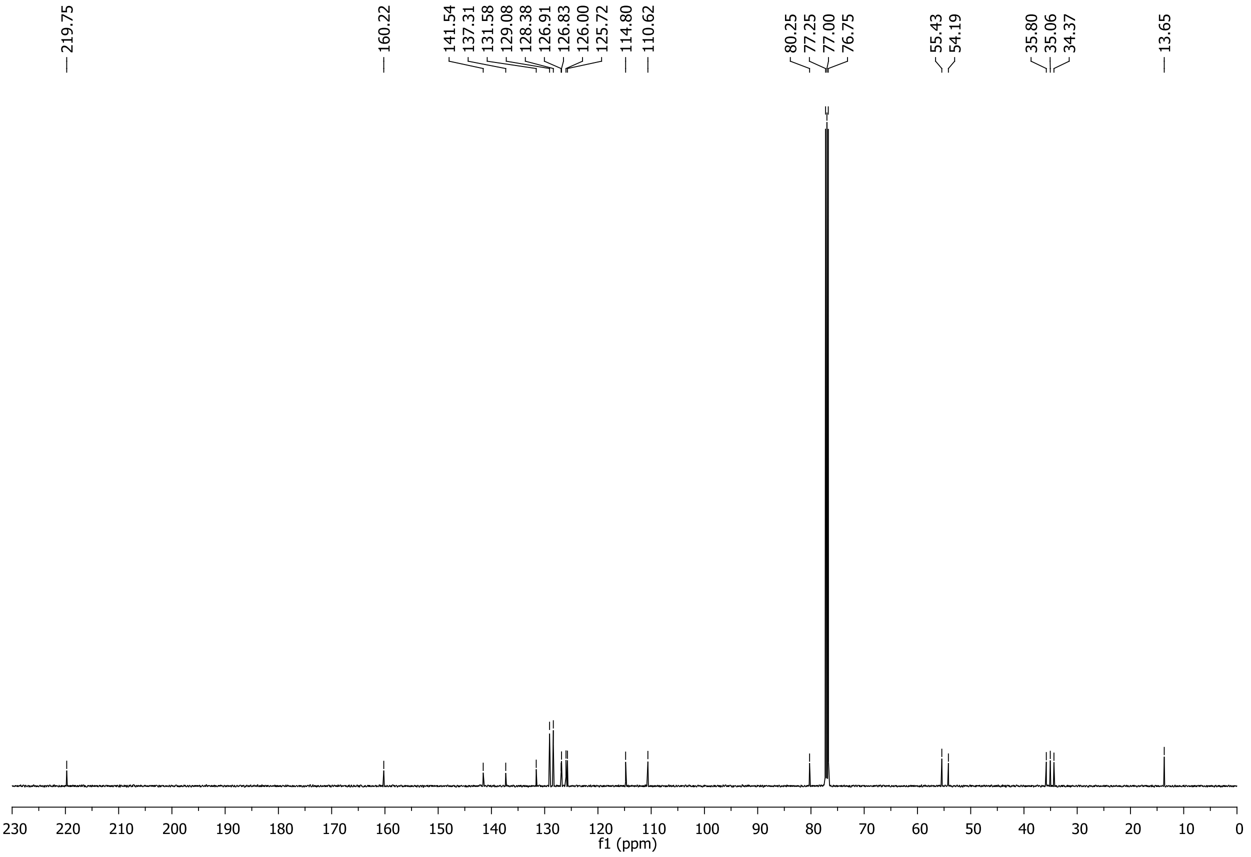
219.75
160.22
141.54
137.31
131.58
129.08
128.38
126.91
126.83
126.00
125.72
114.80
110.62
80.25
77.25
77.00
76.75
55.43
54.19
35.80
35.06
34.37
13.65
230
220
210
200
190
180
170
160
150
140
130
120
110
100
90
80
70
60
50
40
30
20
10
0
f1 (ppm)

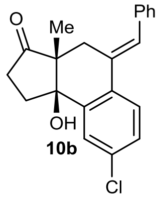
O
Me
Ph
OH
Cl

**10b**

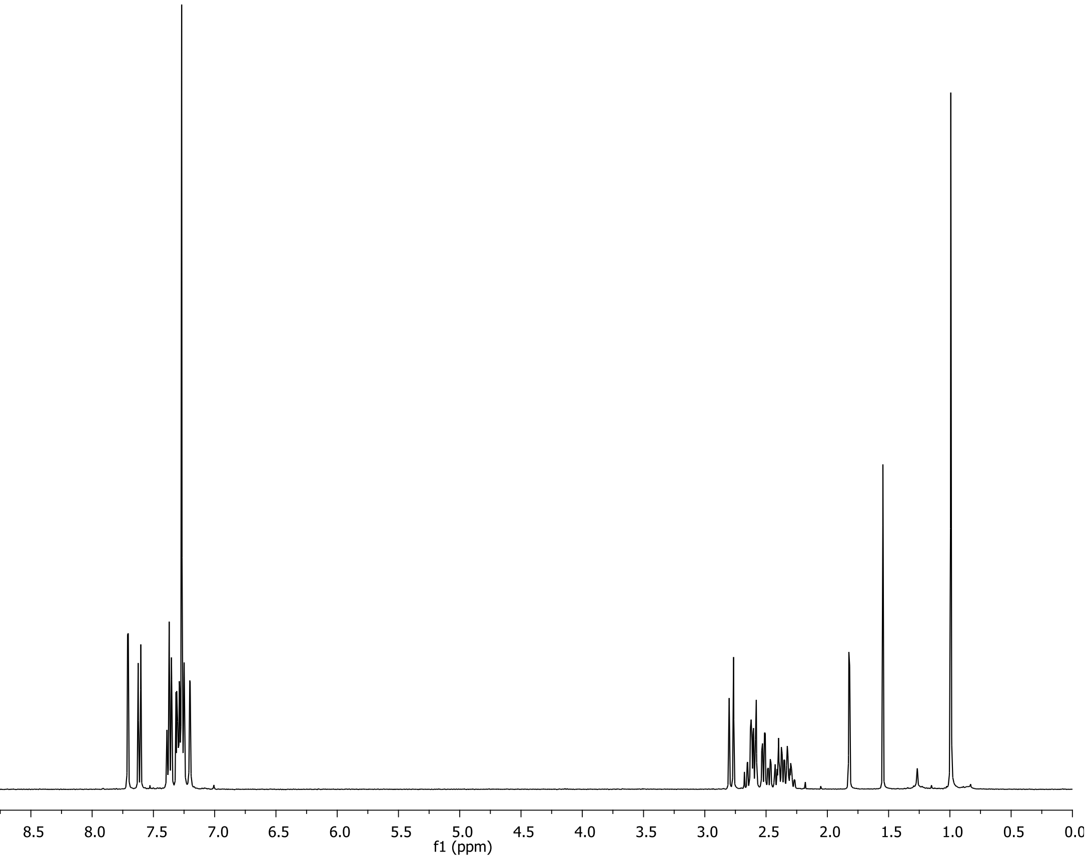
f1 (ppm)

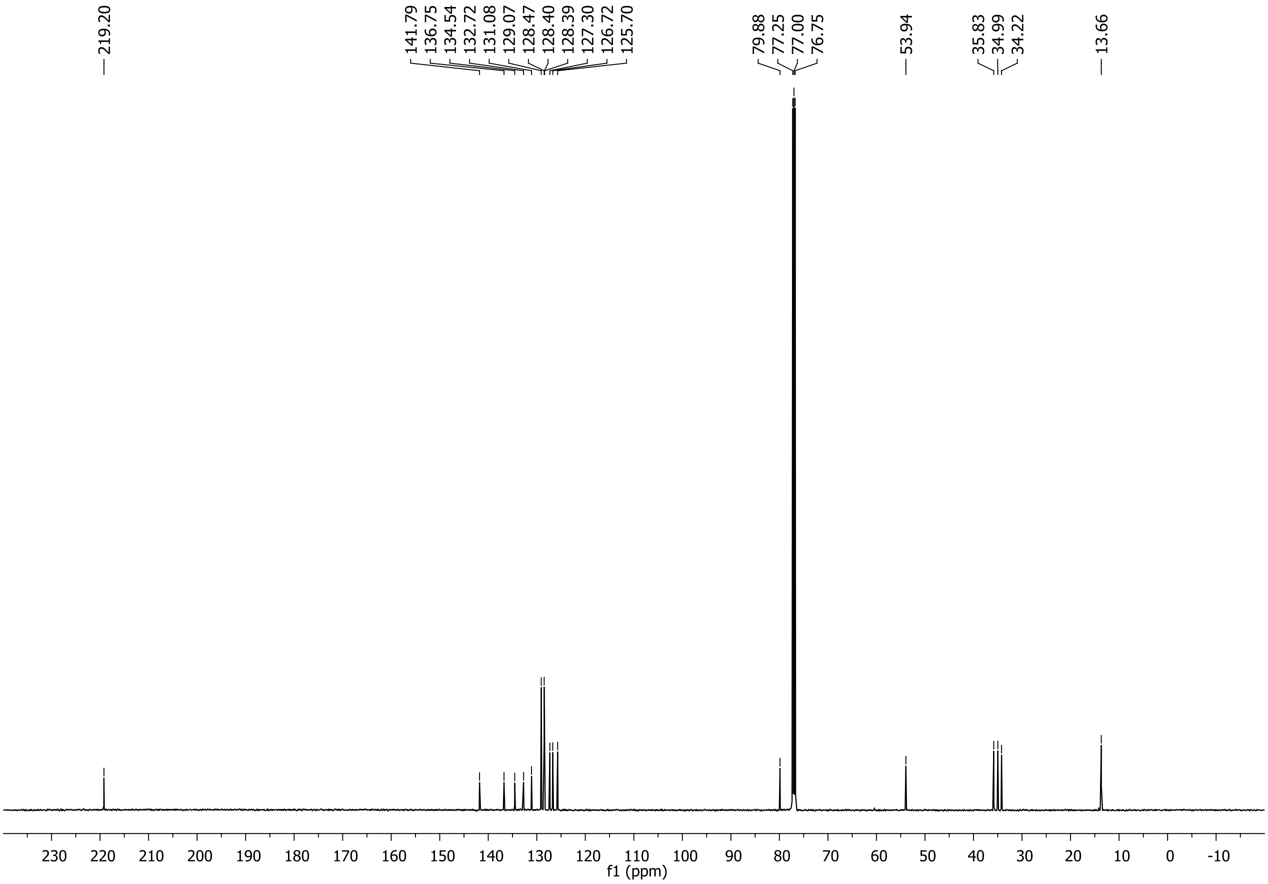
219.20
141.79
136.75
134.54
132.72
131.08
129.07
128.47
128.40
128.39
127.30
126.72
125.70
79.88
77.25
77.00
76.75
53.94
35.83
34.99
34.22
13.66
f1 (ppm)

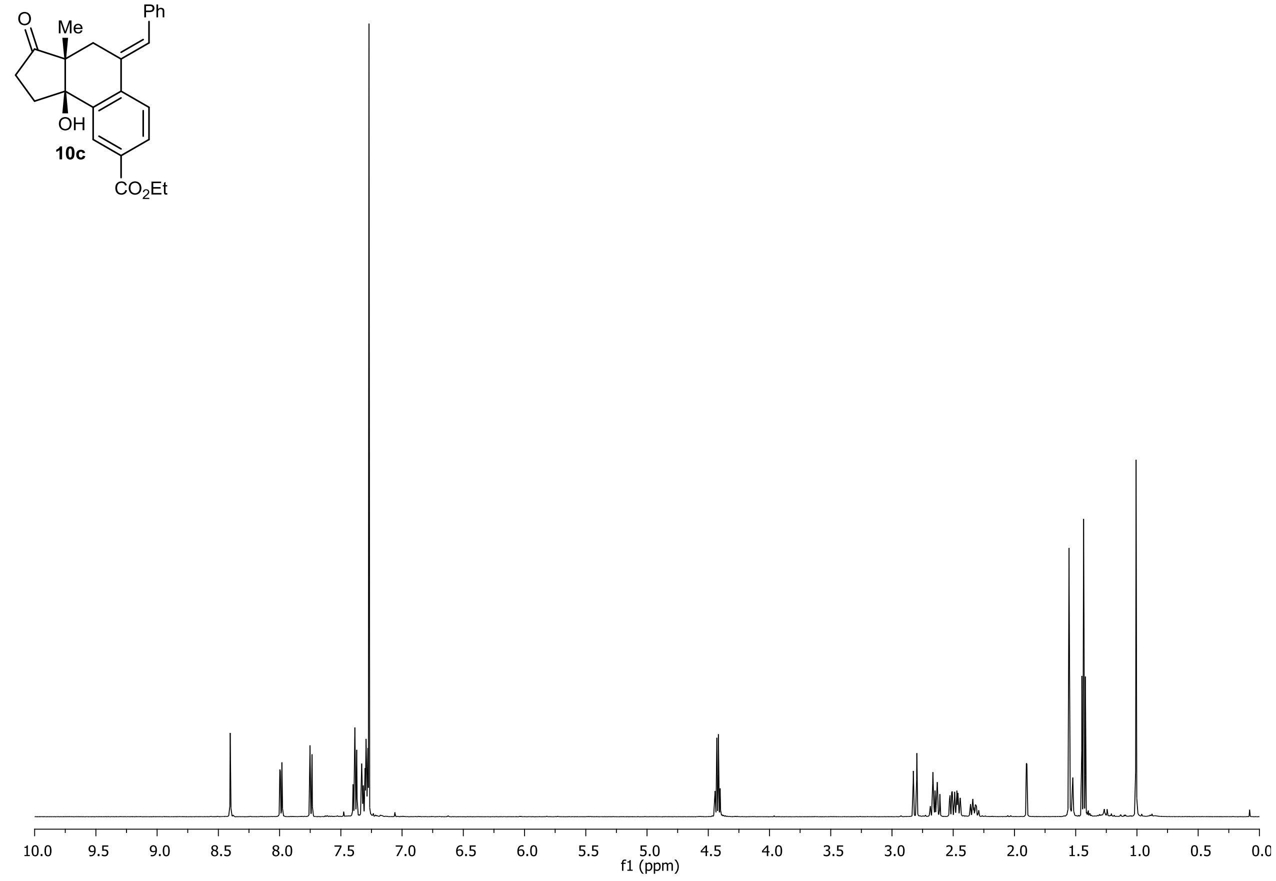
O
Me
Ph
OH
10c
$CO_2Et$
f1 (ppm)

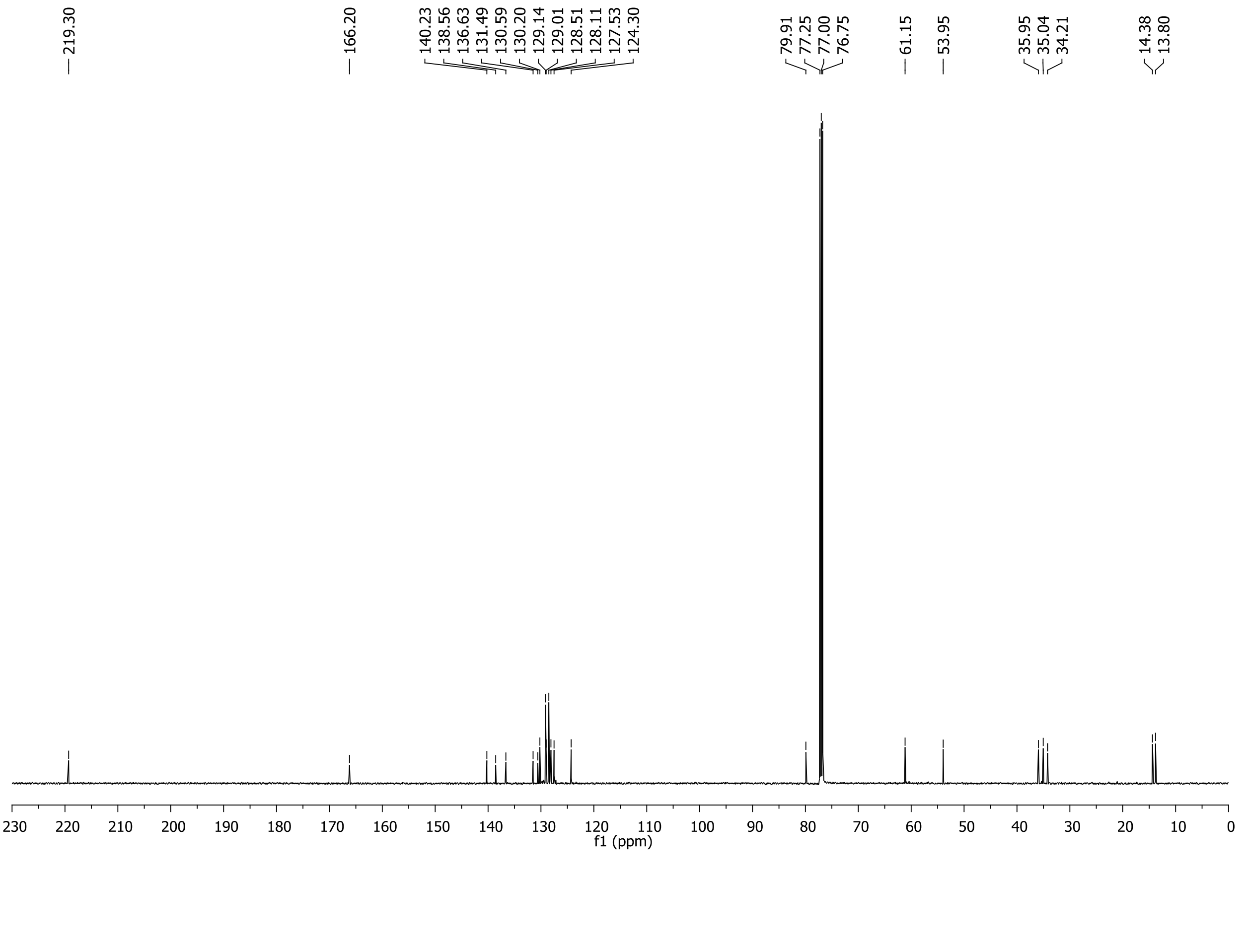
219.30
166.20
140.23
138.56
136.63
131.49
130.59
130.20
129.14
129.01
128.51
128.11
127.53
124.30
79.91
77.25
77.00
76.75
61.15
53.95
35.95
35.04
34.21
14.38
13.80
f1 (ppm)

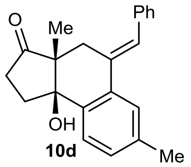
O
Me
Ph
OH
Me

**10d**

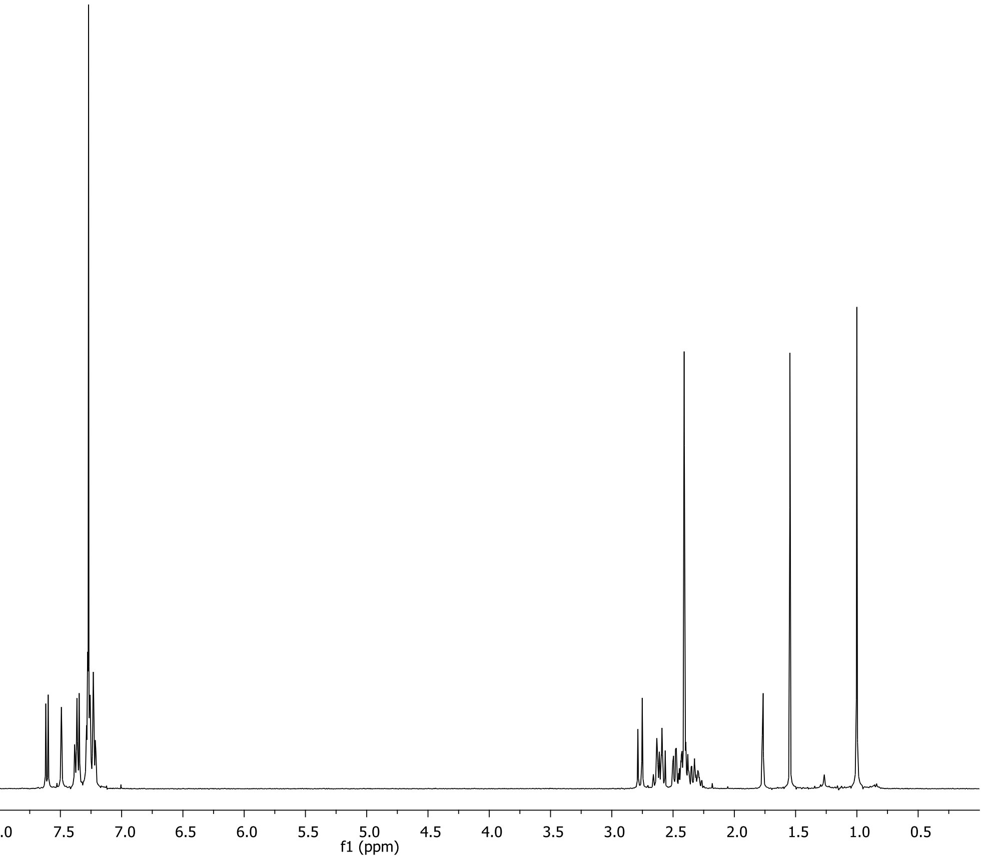
9.5 9.0 8.5 8.0 7.5 7.0 6.5 6.0 5.5 5.0 4.5 4.0 3.5 3.0 2.5 2.0 1.5 1.0 0.5
f1 (ppm)

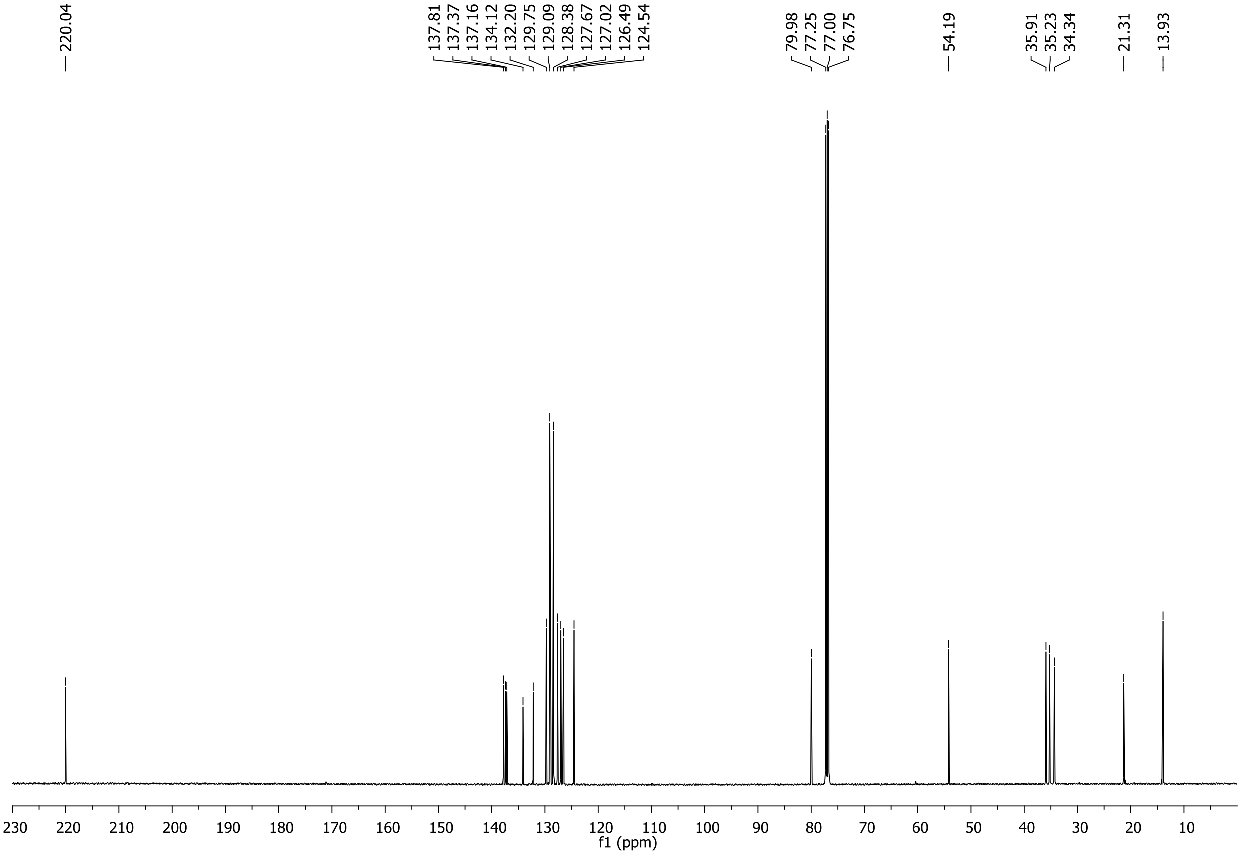
220.04
137.81
137.37
137.16
134.12
132.20
129.75
129.09
128.38
127.67
127.02
126.49
124.54
79.98
77.25
77.00
76.75
54.19
35.91
35.23
34.34
21.31
13.93
230 220 210 200 190 180 170 160 150 140 130 120 110 100 90 80 70 60 50 40 30 20 10
f1 (ppm)

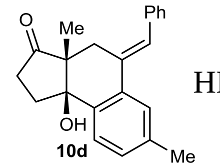


**10d**

HMBC

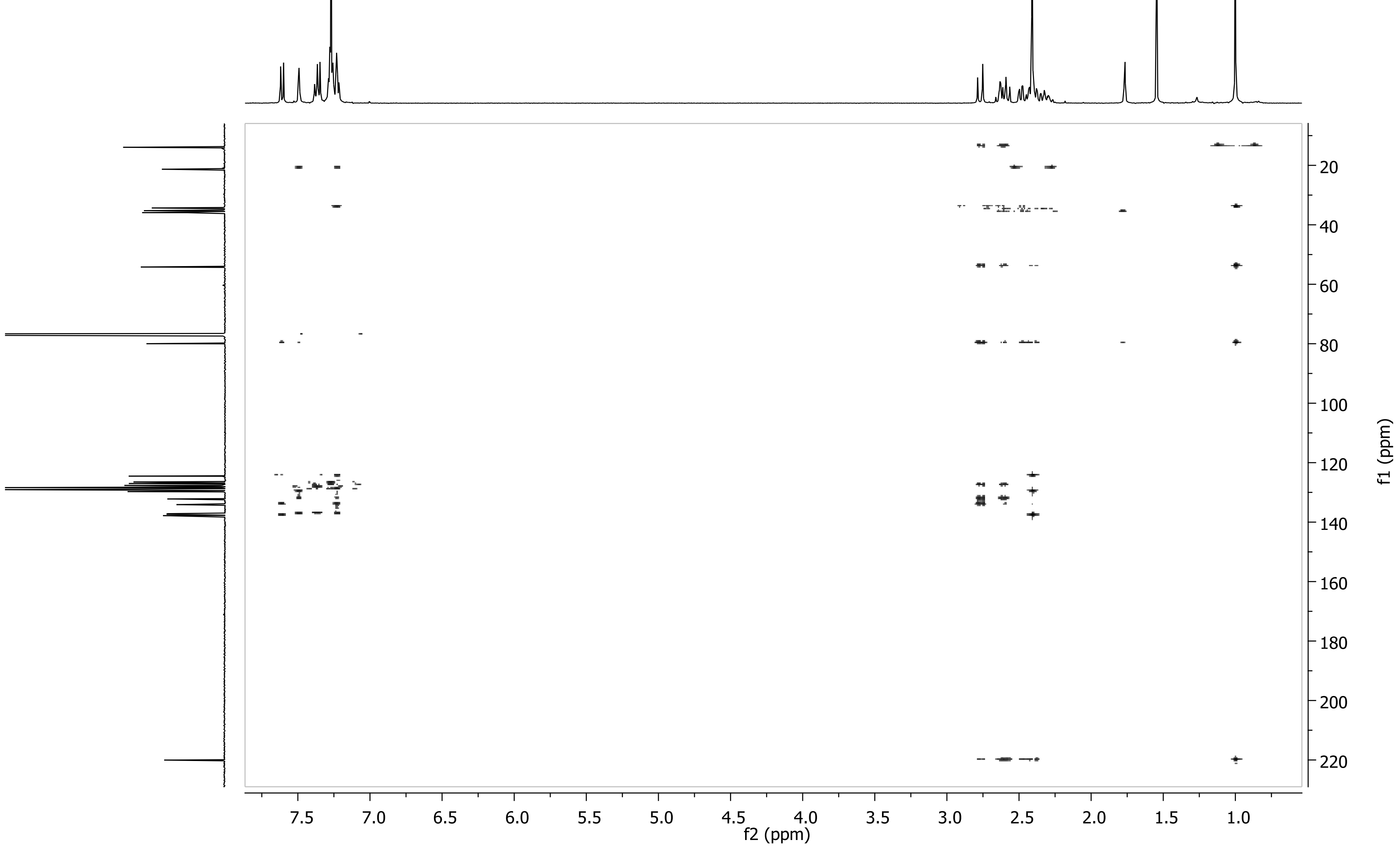

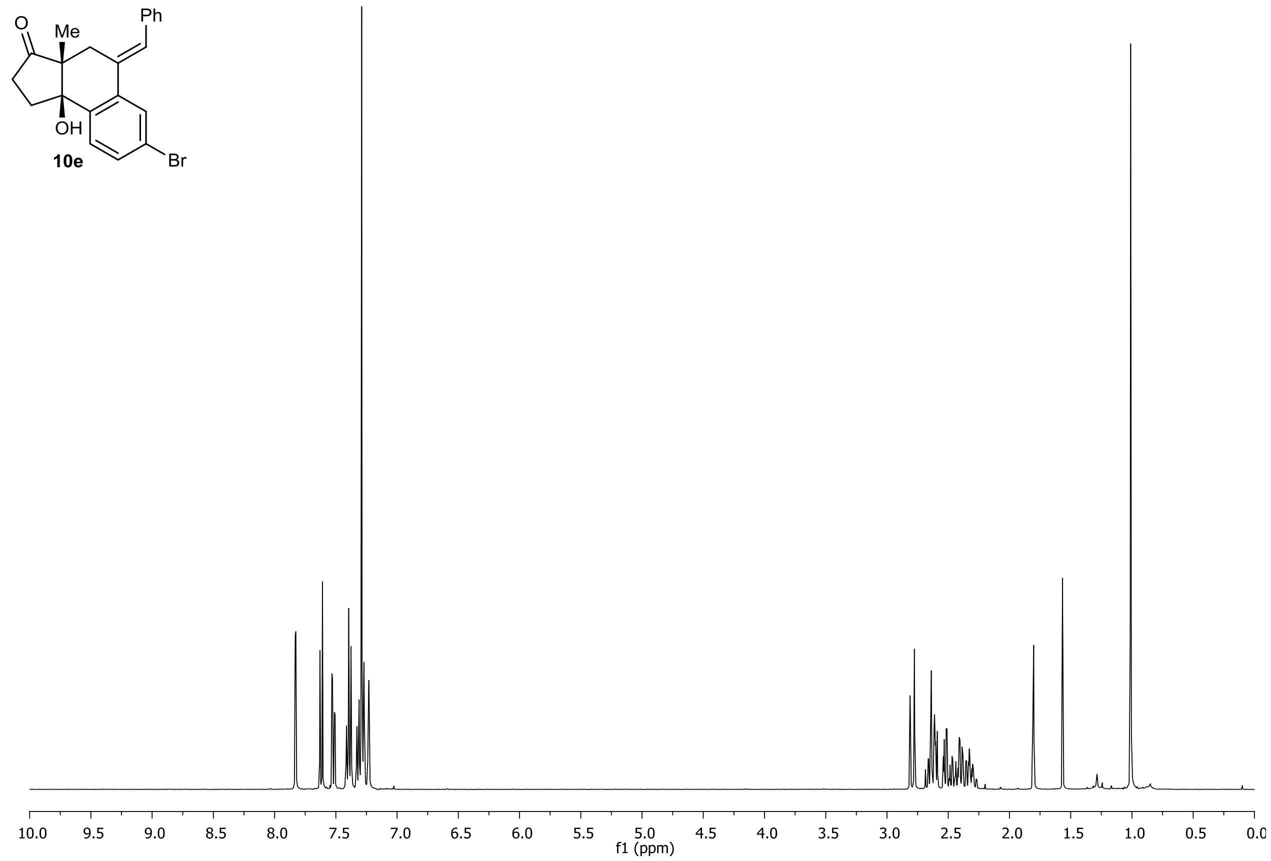
O
Me
Ph
OH
10e
Br
10.0 9.5 9.0 8.5 8.0 7.5 7.0 6.5 6.0 5.5 5.0 4.5 4.0 3.5 3.0 2.5 2.0 1.5 1.0 0.5 0.0
f1 (ppm)

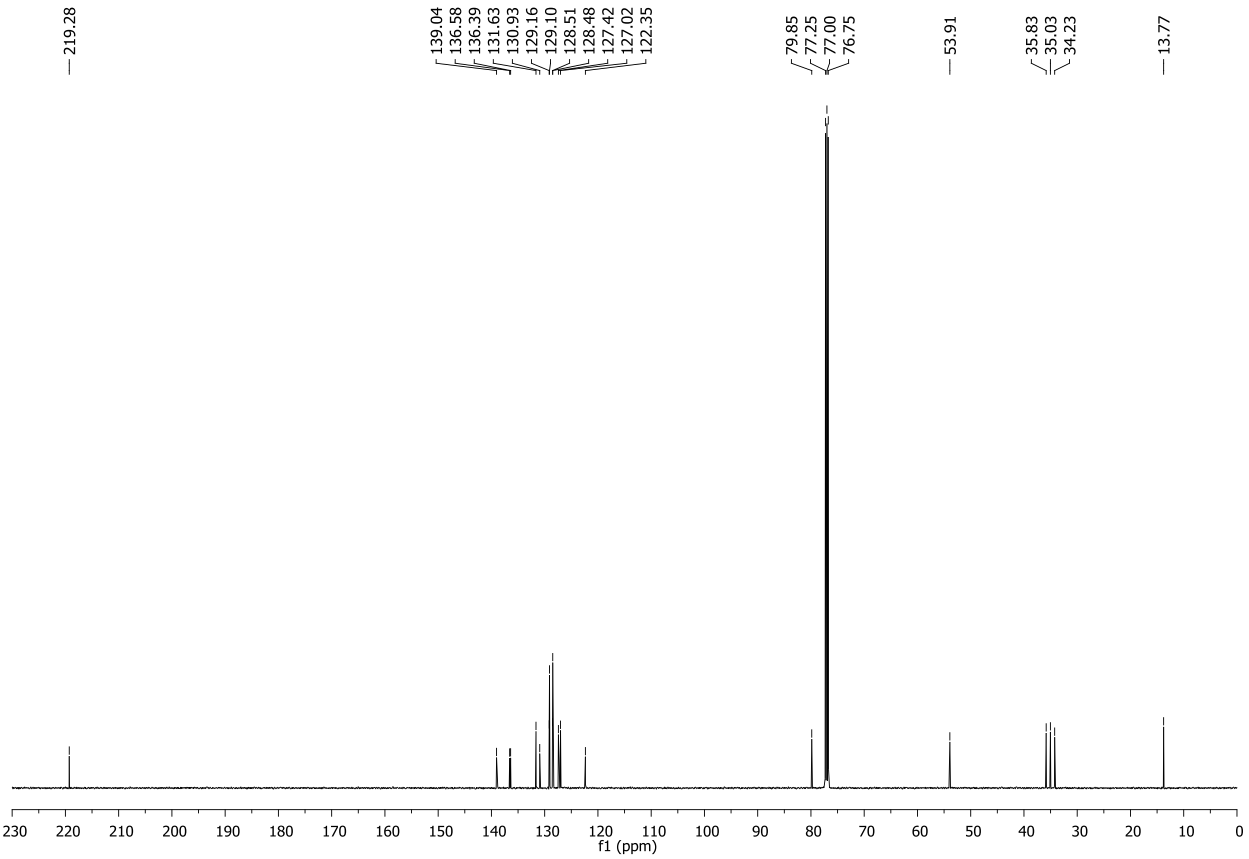
219.28
139.04
136.58
136.39
131.63
130.93
129.16
129.10
128.51
128.48
127.42
127.02
122.35
79.85
77.25
77.00
76.75
53.91
35.83
35.03
34.23
13.77
230 220 210 200 190 180 170 160 150 140 130 120 110 100 90 80 70 60 50 40 30 20 10 0
f1 (ppm)

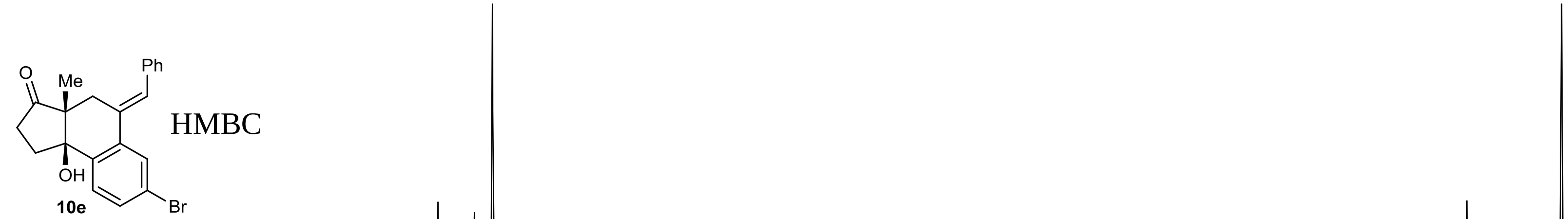
O
Me
Ph
OH
Br
**10e**

HMBC

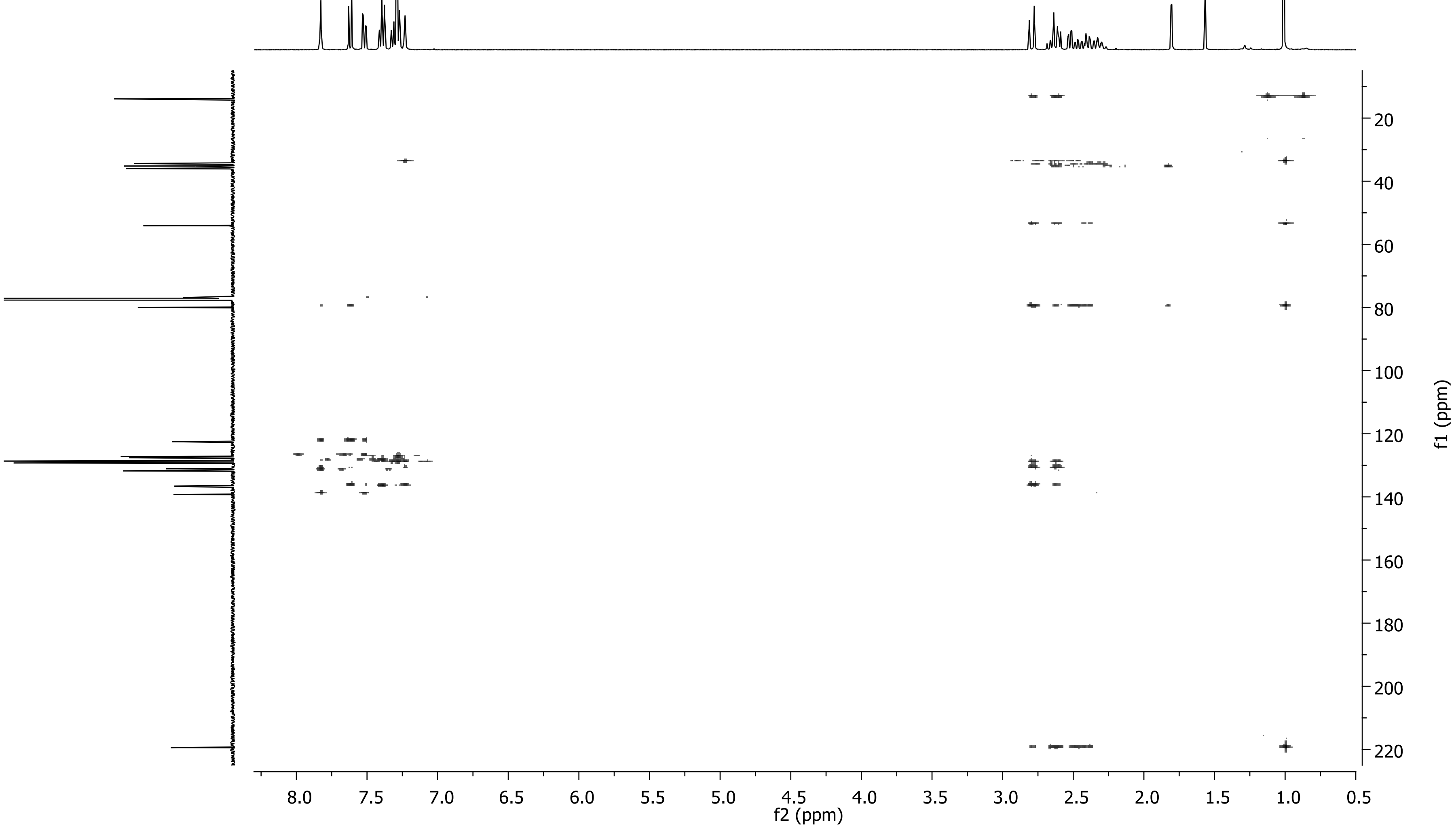
8.0
7.5
7.0
6.5
6.0
5.5
5.0
4.5
4.0
3.5
3.0
2.5
2.0
1.5
1.0
0.5
f2 (ppm)
20
40
60
80
100
120
140
160
180
200
220
f1 (ppm)

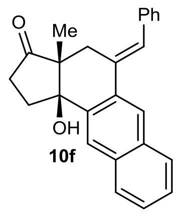
O
Me
Ph
OH

**10f**

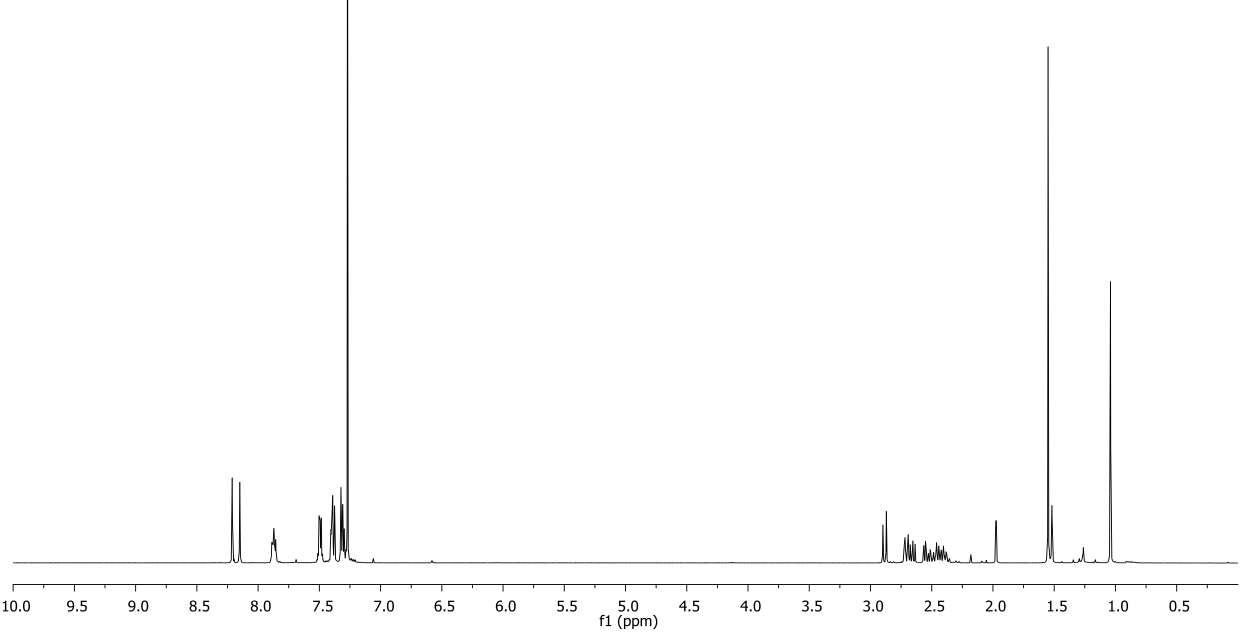
10.0 9.5 9.0 8.5 8.0 7.5 7.0 6.5 6.0 5.5 5.0 4.5 4.0 3.5 3.0 2.5 2.0 1.5 1.0 0.5
f1 (ppm)

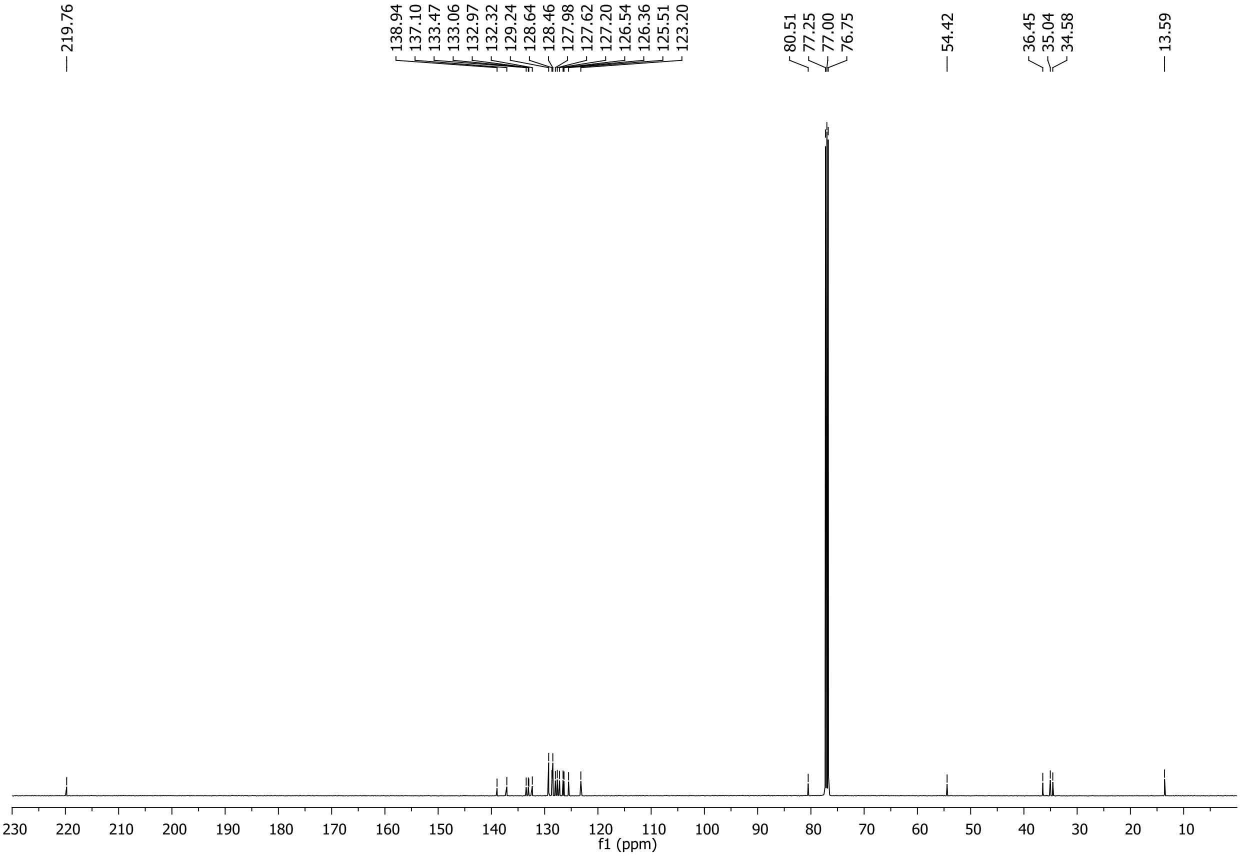
219.76
138.94
137.10
133.47
133.06
132.97
132.32
129.24
128.64
128.46
127.98
127.62
127.20
126.54
126.36
125.51
123.20
80.51
77.25
77.00
76.75
54.42
36.45
35.04
34.58
13.59
230 220 210 200 190 180 170 160 150 140 130 120 110 100 90 80 70 60 50 40 30 20 10
f1 (ppm)

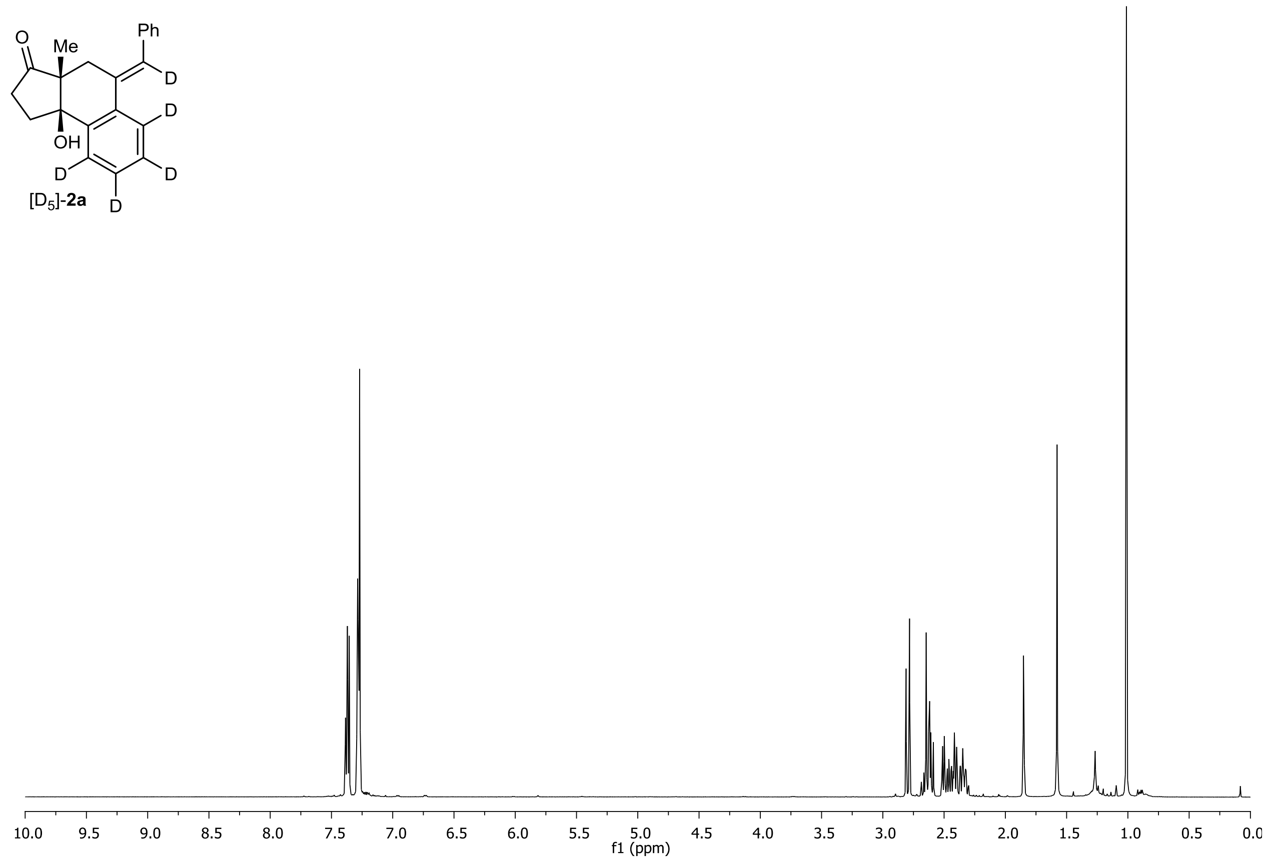
O
Me
Ph
D
D
OH
D
D
D
[D5]-2a
10.0 9.5 9.0 8.5 8.0 7.5 7.0 6.5 6.0 5.5 5.0 4.5 4.0 3.5 3.0 2.5 2.0 1.5 1.0 0.5 0.0
f1 (ppm)

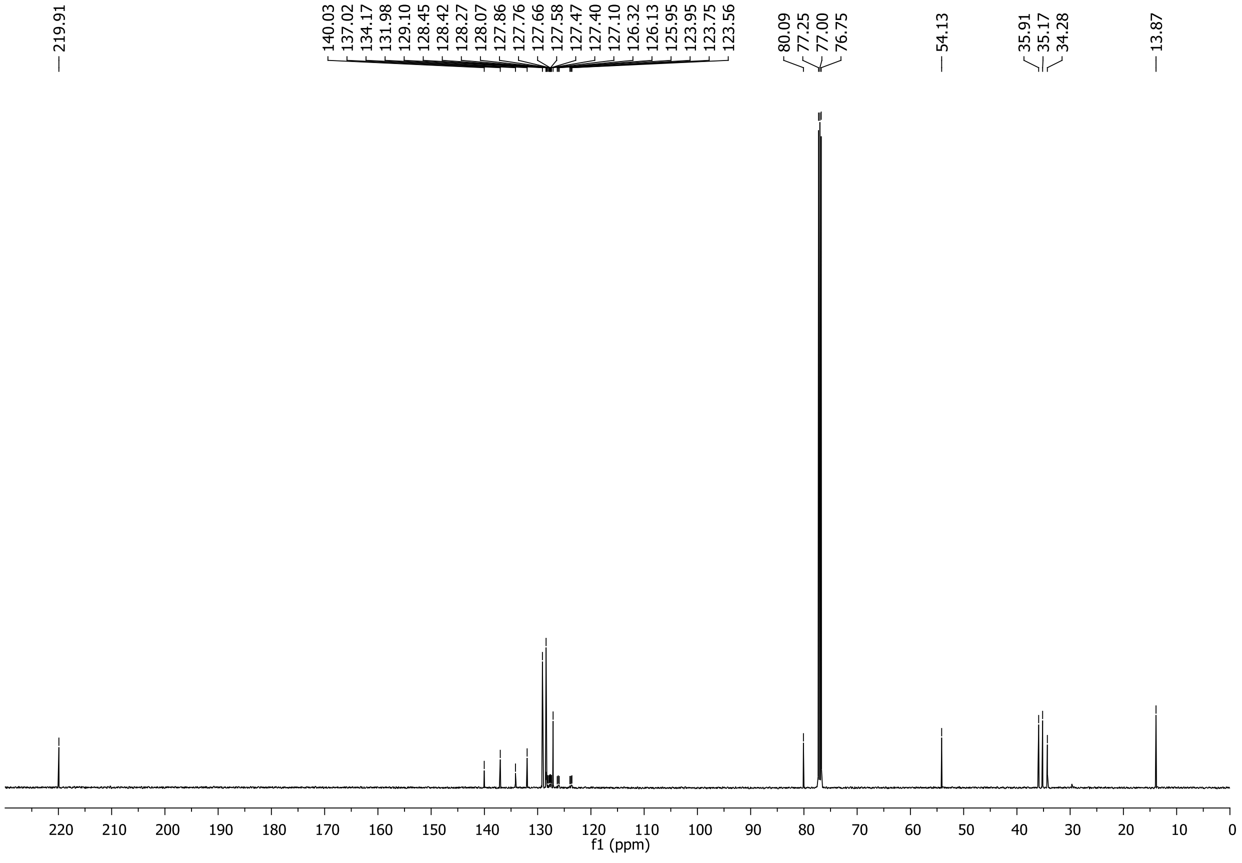
219.91
140.03
137.02
134.17
131.98
129.10
128.45
128.42
128.27
128.07
127.86
127.76
127.66
127.58
127.47
127.40
127.10
126.32
126.13
125.95
123.95
123.75
123.56
80.09
77.25
77.00
76.75
54.13
35.91
35.17
34.28
13.87
220 210 200 190 180 170 160 150 140 130 120 110 100 90 80 70 60 50 40 30 20 10 0
f1 (ppm)
